# Suicide mortality and suicide method trends in the Region of the Americas, 2015–2021: a population-based time-series analysis of progress toward a one-third reduction target

**DOI:** 10.1016/j.lana.2026.101671

**Published:** 2026-10-15

**Authors:** Shannon Lange, Ramon Martinez, Katherine Guo, Anselm J.M. Hennis, Renato Oliveria e Souza

**Affiliations:** a Population & Global Health Institute, Centre for Addiction and Mental Health, 250 College St., Toronto, ON, M5S 2S1, Canada; b Campbell Family Mental Health Research Institute, Centre for Addiction and Mental Health, 250 College St., Toronto, ON, M5T 1R8, Canada; c Department of Psychiatry, University of Toronto, 250 College St., Toronto, ON, M5T 1R8, Canada; d Institute of Medical Science, University of Toronto, 1 King's College Circle, Toronto, ON, M5S 1A8, Canada; e Pan American Health Organization, 525 23rd St. NW, Washington, DC, 20037, USA

**Keywords:** Suicide, Suicide method, Trends, One-third reduction target

## Abstract

**Background:**

The suicide mortality rate is a key global indicator for monitoring progress toward improved mental health and well-being under the Sustainable Development Goals, and the World Health Organization Comprehensive Mental Health Action Plan has set an explicit suicide-specific target of reducing the age-standardized suicide mortality rate by one-third by 2030 (with a baseline year of 2015). This study assessed temporal trends in suicide mortality and suicide methods in the Region of the Americas using average annual percent change (AAPC) for the period 2015–2021.

**Methods:**

Age-standardized and age group-specific suicide mortality rates (per 100,000 population) for the region, subregions, and by country for 2000–2021, and proportion of suicides by method by country, overall and by sex for 2015–2021, were obtained from the World Health Organization Global Health Estimates and the Pan American Health Organization Regional Mortality Database, respectively. In this population-based time-series analysis, AAPC was estimated using joinpoint regression analyses. The AAPCs for suicide mortality rates between 2015 and 2021 were compared with the compound annual reduction required to achieve a one-third reduction by 2030 (−2·67%).

**Findings:**

The regional age-standardized suicide mortality rate increased by 1·05% (95% CI: 0·85%, 1·26%; p ≤ 0·0001) annually on average between 2015 and 2021, driven by males (AAPC: 0·99%, 95% CI: 0·81%, 1·17%; p ≤ 0·0001), as the rate among females remained relatively stable during this period (AAPC: 0·02%, 95% CI: −1·38%, 1·45%; p = 0·97). Only 10 countries (30·3%; out of 33) showed significant declines exceeding the 2·67% annual reduction needed to achieve the one-third reduction target by 2030. Across countries, the distribution of recorded suicide methods shifted over time, with increases in the proportion of suicides involving hanging and decreases in the proportions involving firearms and poisoning.

**Interpretation:**

Suicide mortality in the region is rising, and the target of a one-third reduction by 2030 is unlikely to be met regionally; however, progress varies considerably across countries, with 10 (30·3%) making substantial progress toward the target. Urgent, coordinated action is needed in the countries with high and increasing suicide mortality rates, combining evidence-based means restriction strategies with broader upstream interventions that address social, economic and health-system determinants of suicide risk.

**Funding:**

None.

**Research in context**

**Evidence before this study**

When the World Health Organization (WHO) released Global Health Estimates of suicide mortality for 2000–2019, the Region of the Americas was the only region to experience an increase in age standardized suicide mortality, whereas all other regions experienced declining rates. This increase occurred in the context of international calls for global action and the establishment of the Sustainable Development Goals (SDG) target 3·4—“By 2030, reduce by one third premature mortality from non communicable diseases through prevention and treatment and promote mental health and well being”—for which suicide mortality rate serves as one of two indicators (3·4·2). The WHO subsequently set a suicide-specific target of reducing the age-standardized suicide mortality rate by one-third by 2030 with their Comprehensive Mental Health Action Plan, extended from 2013–2020 to 2013–2030 and setting the baseline year to 2015 to align with the 2030 Agenda for Sustainable Development. We searched PubMed, Scopus, and Google Scholar from database inception through December 2025 using combinations of the terms suicide, mortality, trends, Sustainable Development Goals, SDG 3·4, mental health, and Americas. Previous studies have assessed global and regional trends in premature mortality from major noncommunicable diseases in relation to SDG target 3·4; however, suicide mortality was only examined as part of broader noncommunicable disease groupings or not analysed in detail. A limited number of studies have described suicide trends in select countries or subregions of the Americas, often relying on national data sources and heterogeneous methods, which limits cross-country comparability.

**Added value of this study**

This study provides the first comprehensive, region-wide assessment of progress toward the one-third reduction target in suicide mortality rates by 2030 within the Region of the Americas, using the most recent available data from WHO Global Health Estimates (2000–2021). By benchmarking observed trends against the annual rate of decline required to achieve a one-third reduction in suicide mortality by 2030, we identified countries that are on track, those making insufficient progress, and those experiencing worsening trends. By integrating data from the Pan American Health Organization Regional Mortality Database, we concurrently examined patterns in the proportion of suicides by method across a subset of countries. Our findings reveal substantial heterogeneity across countries in progress towards the one third reduction target, an overall increase in regional suicide mortality, and broadly consistent shifts in the distribution of suicide methods, with increasing proportions involving hanging and declining proportions involving firearms and poisoning, regardless of overall suicide mortality trends. Together, these analyses generate actionable evidence to evaluate progress toward the internationally agreed suicide reduction target, guide public health prioritization, and inform the implementation and refinement of suicide prevention strategies.

**Implications of all the available evidence**

Available evidence indicates that, as of 2021, the Region of the Americas as a whole is not on track to achieve the one-third suicide mortality rate reduction target by 2030, with many countries experiencing relatively stable or increasing suicide mortality. Although reductions in the proportion of suicides involving firearms and poisoning are consistent with the potential benefits of means-specific interventions, the concurrent rise in the proportion of suicides involving hanging highlights the limitations of relying on means restriction alone. Achieving meaningful progress will require comprehensive national suicide prevention strategies that combine evidence-based means restriction with strengthened mental health systems responsive to the underlying social and structural determinants of suicide and capable of identifying and providing timely care to those at risk. Given the marked heterogeneity observed across countries and between sexes and age-groups, regional efforts to accelerate suicide prevention across the Americas must also be adaptable to local contexts.

## Introduction

Approximately 101,000 deaths by suicide occurred in the Region of the Americas in 2021, corresponding to an age-standardized suicide mortality rate of 9·2 (95% uncertainty interval [UI]: 8·2, 10·2) per 100,000 population.^1^ The rate was substantially higher among males (14·7, 95% UI: 13·2, 16·2 per 100,000 population) than females (4·0, 95% UI: 3·5, 4·4 per 100,000 population), with approximately 3·7 male suicide deaths for every female suicide death.^1^

Suicide is a critical public health challenge arising from the interplay of biological, social, psychological, cultural, and environmental factors. Despite its complexity, suicide is largely preventable through a combination of individual- and population-level interventions.^2^^,^^3^ The need for strengthened suicide prevention has been recognized by international public health agencies. In 2015, the United Nations (UN) adopted the Sustainable Development Goals (SDGs), including Goal 3, which aims to “ensure healthy lives and promote well-being for all at all ages”.^4^ Within this goal, target 3·4 calls for a one-third reduction in premature mortality from non-communicable diseases by 2030 and the promotion of mental health and well-being. The suicide mortality rate is designated as indicator 3·4·2.^4^ The World Health Organization (WHO) Comprehensive Mental Health Action Plan, extended from 2013 to 2020 to 2013–2030 and setting the baseline year to 2015 to align with the 2030 Agenda for Sustainable Development, then established a suicide-specific target of reducing the age-standardized suicide mortality rate by one-third by 2030.^5^ Together, these commitments provide a global benchmark for evaluating progress in suicide prevention.

The 2019 WHO Global Health Estimates (GHE) release^6^ indicated that the Region of the Americas was the only WHO region in which suicide mortality rates were increasing, despite long-standing recognition of suicide as a major public health concern in the region. The most recent data release, extending through 2021, enables an assessment of whether countries in the Region of the Americas are on track to achieve the one-third reduction target. The primary objective of this study was therefore to evaluate age-standardized suicide mortality rate trends in the Region of the Americas between 2015 and 2021 and assess progress towards achieving the one-third reduction target by 2030. In addition, given the central role of means availability in shaping suicide risk, we aimed to evaluate trends in the distribution of suicide methods to explore associations with observed suicide mortality trends.

Two studies have previously assessed global trends in premature mortality from major noncommunicable diseases and progress towards the SDG target 3·4^7^^,^^8^; however, suicide mortality was not examined in detail nor as a standalone outcome. To date, no study has comprehensively assessed suicide mortality rate trends in relation to SDG indicator 3·4·2 across all countries of the Region of the Americas, nor examined how changes in suicide method may relate to observed suicide mortality trends.

This study integrates complementary data sources to provide a more comprehensive assessment of suicide mortality trends and prevention progress than would be possible using a single data source alone. WHO GHE provide harmonized, comparable estimates of suicide mortality across countries, while the PAHO mortality database enables an examination of changes in the distribution of suicide methods using country-reported mortality data. By utilizing both of these sources, this study simultaneously evaluates progress towards the internationally set reduction target and characterizes changes in suicide method distributions across the Region of the Americas. This integrated approach provides important insights into the complexity of suicide prevention efforts, highlighting that changes in overall suicide mortality and method distributions may or may not occur in parallel and should be considered together when evaluating regional progress. Distinct from our previous analysis evaluating the impact of implementing national suicide prevention strategies on suicide mortality in nine countries within the Region of the Americas,^9^ the present study assesses country-level progress toward the international suicide mortality reduction target alongside changes in the distribution of suicide methods across the region.

## Methods

We conducted a population-based time-series analysis of suicide mortality rate and distribution of suicide methods in the Americas to estimate the trends during the period 2015–2021. This longitudinal time-series analysis is reported in line with the GATHER statement.^10^ The Region includes 35 countries grouped into five geographic subregions: Andean Area (Bolivia, Colombia, Ecuador, Peru, and Venezuela), Central America, Mexico and Latin Caribbean (Costa Rica, Cuba, Dominican Republic, El Salvador, Guatemala, Haiti, Honduras, Mexico, Nicaragua, and Panama), Non-Latin Caribbean (Antigua and Barbuda, Bahamas, Barbados, Belize, Dominica, Grenada, Guyana, Jamaica, Saint Lucia, Saint Kitts and Nevis, Saint Vincent and the Grenadines, Suriname, and Trinidad and Tobago), North America (Canada and United States of America), and the Southern Cone and Brazil (Argentina, Brazil, Chile, Paraguay, and Uruguay). Additionally, suicide mortality rate estimates are produced for Puerto Rico, as it is one of the largest populations in non-Member State territories; however, these data are only included in regional population-weighted totals and not available on the country level.

### Data sources and definitions

The most recent available data, at the time of analysis, on the age-standardized and age-group specific suicide mortality rates (≤24 years, 25–49 years, 50–64 years, 65–74 years, ≥75 years) per 100,000 population and their corresponding 95% UI were obtained from the WHO GHE^11^ for 2000–2021. Estimates were available for the region, subregions, and each of the 35 countries, both overall and stratified by sex. More recent GHE releases were not available at the time of this assessment. Suicide is a cause of death category included in the GHE cause list, defined according to the following International Classification of Diseases tenth revision (ICD-10) codes: X65-X83. The data sources and methodological details of the GHE are described in detail elsewhere.^11^ In brief, the GHE produce comparable measures of causes of death and disease burden globally, and across countries and WHO regions (as well as subregions for the Americas) by age, sex, and year from 2000 to 2021. Estimates are based on data from national vital registration, WHO technical programs, UN partners and inter-agency groups, and the Global Burden of Diseases, Injuries, and Risk Factors Study. WHO uses the completeness of death registration, the quality of the cause of death information, and the timeliness and frequency of data provided to WHO to classify countries into the following four data quality categories: i) high (≥5 years of reporting since 2010 and usability ≥80%); ii) medium (≥5 years of reporting and usability 60–79%, or ≥80% for countries reporting only shortlist causes); iii) low (at least 1 year of reporting and usability 40–59%); and iv) very low (usability <40% or insufficient reporting to meet higher categories). Based on the data quality assessment, standard methods are applied to produce precise and internationally comparable estimates. Total deaths by five-year age group and sex are estimated for each country by applying the WHO life table death rates^12^ to the estimated resident populations prepared by the UN Population Division in its 2024 revision.^13^ In order to facilitate international comparability, age-standardized suicide mortality rates are computed by the direct method using the WHO standard population,^14^ which assumes one standard age distribution for all countries. The corresponding UIs reflect uncertainty arising from data availability, completeness, and modelling assumptions within the GHE framework. It should be noted that the subregional and regional suicide mortality rates are population-weighted estimates, reflecting the contribution of all 35 countries (and Puerto Rico) according to their population size.

Data on the proportion of total suicides by method was obtained by country, overall and by sex from the Pan American Health Organization (PAHO) Regional Mortality Database for 2015–2021.^15^ This database comprises cause-of-death data from national civil registration and vital statistics reported by Member States to PAHO and serves as a primary input source for the GHE. However, the PAHO mortality data themselves are not subjected to the full GHE estimation and adjustment procedures. The following cause categories, defined via their respective ICD-10 codes, were examined: hanging (X70), firearms (X72–X74), jumping (X80), poisoning (X60–X69), and a residual category comprising other specified and unspecified methods (X71, X75–X79, X81–X84). Data on the proportion of suicides by method were available for 20 countries; 18 countries (Argentina, Belize, Brazil, Canada, Chile, Colombia, Costa Rica, Cuba, Ecuador, Dominican Republic, Guatemala, Mexico, Nicaragua, Panama, Paraguay, Peru, United States of America, and Uruguay) for the period 2015–2021 and two countries (Guyana and Trinidad and Tobago) for 2015–2020.

The two data sources were analysed separately and were not combined: WHO GHE were used for analyses of suicide mortality rate trends, whereas PAHO mortality data were used for analyses of the distribution of suicide methods, with both the numerator and denominator of the method-specific proportions derived exclusively from the latter.

### Ethical statement

Institutional review board approval and informed consent were not required, as the current study utilized aggregated data obtained from public domain databases.

### Statistical analysis

Joinpoint regression analyses^16^ were performed on age-standardized and age group-specific suicide mortality rates and proportions of suicides by method over the full time-series (2000–2021 and 2015–2021, respectively), overall and by sex, using the Joinpoint Trends Analysis software.^17^ Joinpoint regression analysis is a data-driven statistical technique that identifies points of inflection and divides a time-series into linear segments. Using a permutation-based model selection method, the most parsimonious model was selected, such that the addition of another joinpoint did not significantly improve model fit. The model for suicide mortality rates was configured to detect a maximum of two joinpoints, whereas the model for the proportion of suicides by method was configured to detect a maximum of one joinpoint, and required linear segments of at least two data points. These settings were chosen to reduce the risk of overfitting while allowing sufficient flexibility to identify major changes in trend. Both outcome measures (suicide mortality rates and proportion of suicides by method) were log transformed. For the method-specific analyses, the log-linear specification was selected to align with the study objective. A logit specification would instead estimate temporal changes in the odds of a suicide involving a given method relative to all other methods, representing a different estimand. Constant variance in the time series was assumed. Inspection of residuals and residual autocorrelation functions for suicide mortality rate models did not indicate substantial departures from the assumptions of constant variance or uncorrelated errors (Supplementary Figures S1 and S2). Given the short time series for the proportion of suicides by method, an uncorrelated error structure was specified a priori. The slope coefficient for each segment was transformed to an annual percentage change (APC). The average annual percent change (AAPC) was then calculated for 2015–2021 as a weighted mean of the APCs for the model segments spanning this interval, with weights proportional to the length of each segment. Fitting the joinpoint models for suicide mortality rate over the full 2000–2021 time-series allowed underlying changes in trend to be identified using all available information, providing more stable estimation of trend segments than would be possible using only the seven annual observations from 2015 to 2021. The analysis of suicide methods was restricted to the 2015–2021 period to maximize the number of countries with complete and useable data. Ninety-five percent confidence intervals (CIs) were obtained via the parametric method, and statistical significance (alpha = 0·05) was evaluated using a two-sided t-test of the null hypothesis that the true AAPC is zero. AAPC was estimated for the age-standardized suicide mortality rates overall and by sex for each country, subregion, and the region overall. Regional and subregional AAPCs were estimated independently from population-weighted annual rates for the respective geographic level and were not derived by averaging or aggregating country-specific AAPCs. Consequently, country-, subregional-, and regional-level AAPCs are not expected to reconcile directly. Similarly, overall and sex-specific AAPCs were estimated independently and therefore are not constrained to reconcile as weighted averages of one another. Puerto Rico was included only in these population-weighted regional totals and therefore contributed proportionally to the regional trend estimate through its population size and mortality rates. AAPCs were not calculated for any time series containing a value of zero for either the suicide mortality rate or the proportion of suicides by method. Based on this criterion, Antigua and Barbuda (2009–2013 and 2017–2019) and Saint Vincent and the Grenadines (2017–2018) were excluded from the overall age-standardized suicide trend analyses. Both countries were also excluded from the sex-specific analyses because of zero-valued rates among females (Antigua and Barbuda: 2000–2013 and 2017–2019; Saint Vincent and the Grenadines: 2017–2021) and males (Antigua and Barbuda: 2009–2019; Saint Vincent and the Grenadines: 2017–2018). Grenada was excluded from the sex-specific analyses because of zero-valued rates among females (2009–2011) and males (2001, 2014, and 2018), but was retained in the overall analysis because the sex-specific zero values occurred in different years and the overall mortality rate was therefore non-zero throughout the study period. Overall, 33 countries were included in the country-level analysis of overall suicide mortality rates, 32 in the sex-specific mortality trend analyses, and 20 in the analyses of the proportion of suicides by method, overall and by sex. Joinpoint trend analyses were conducted on point estimates only.

We conducted an exploratory assessment of potential bias related to variation in country-level data quality. WHO data-quality classifications were compared with progress classifications using Fisher's exact test, and an ordinal logistic regression model was used to evaluate whether progress classification differed by data-quality category. The two lowest data-quality categories (‘low’ and ‘very low’) were combined due to sparse cell counts.

A statistically significant AAPC above zero indicates an increasing trend and a statistically significant AAPC below zero indicates a decreasing trend, while an AAPC that is not statistically significant indicates that the trend is relatively stable. To assess the progress toward achieving the target of a one-third reduction over 15 years (2015–2030), the observed AAPC in the age-standardized suicide mortality rate was compared with the compound annual reduction required to reach this target (that is, (1-(0·667)^1/15^)∗100 = −2·67% per year). For this comparison, four categories were defined: i) Substantial progress towards the target or on track (statistically significant AAPC ≤ −2·67%); ii) Fair progress towards the target, but additional efforts needed (statistically significant AAPC > −2·67% and <0%), iii) No progress (AAPC not statistically significant), and iv) Worsening situation (statistically significant AAPC ≥0%).

The absolute change in the number of suicides between 2015 and 2021 was calculated by subtracting the GHE-estimated number of deaths in 2015 from that in 2021.

### Sensitivity analyses

Three sensitivity analyses were performed. First, to evaluate the robustness of the CI estimation method used, empirical 95% CIs for the AAPCs were estimated using the empirical quantile method. The joinpoint model identified in the primary analysis was refitted to 5000 resampled datasets generated by resampling residuals from their empirical distribution, and the 2·5th and 97·5th percentiles of the resulting AAPC estimates were used to define the empirical CI. Second, to assess the potential influence of the COVID-19 pandemic period on estimated suicide mortality rate trends, we conducted a sensitivity analysis in which the joinpoint regression analyses were repeated excluding the years 2020 and 2021. Third, a simple log-linear model with no joinpoint was fitted to the proportion of suicides by method for the country–method combinations where model selection indicated that one joinpoint provided the best fit, given that the relatively short time series can limit the stability of more complex joinpoint models.

### Role of the funding source

This research received no specific funding.

## Results

The age-standardized suicide mortality rate in 2021 and trend over time across countries in the Region of the Americas are presented in Fig. 1. The age-standardized suicide mortality rate in the region increased on average by 1·05% (95% CI: 0·85%, 1·26%) annually between 2015 and 2021, reaching 9·16 (95% UI: 8·20, 10·18) per 100,000 population in 2021. This upward trend indicates that, at the regional level, suicide mortality is not declining at the pace required to achieve the one-third reduction target by 2030, but rather increasing significantly. For the suicide mortality rate trends over time see Supplementary Figure S3.

**Fig. 1:**
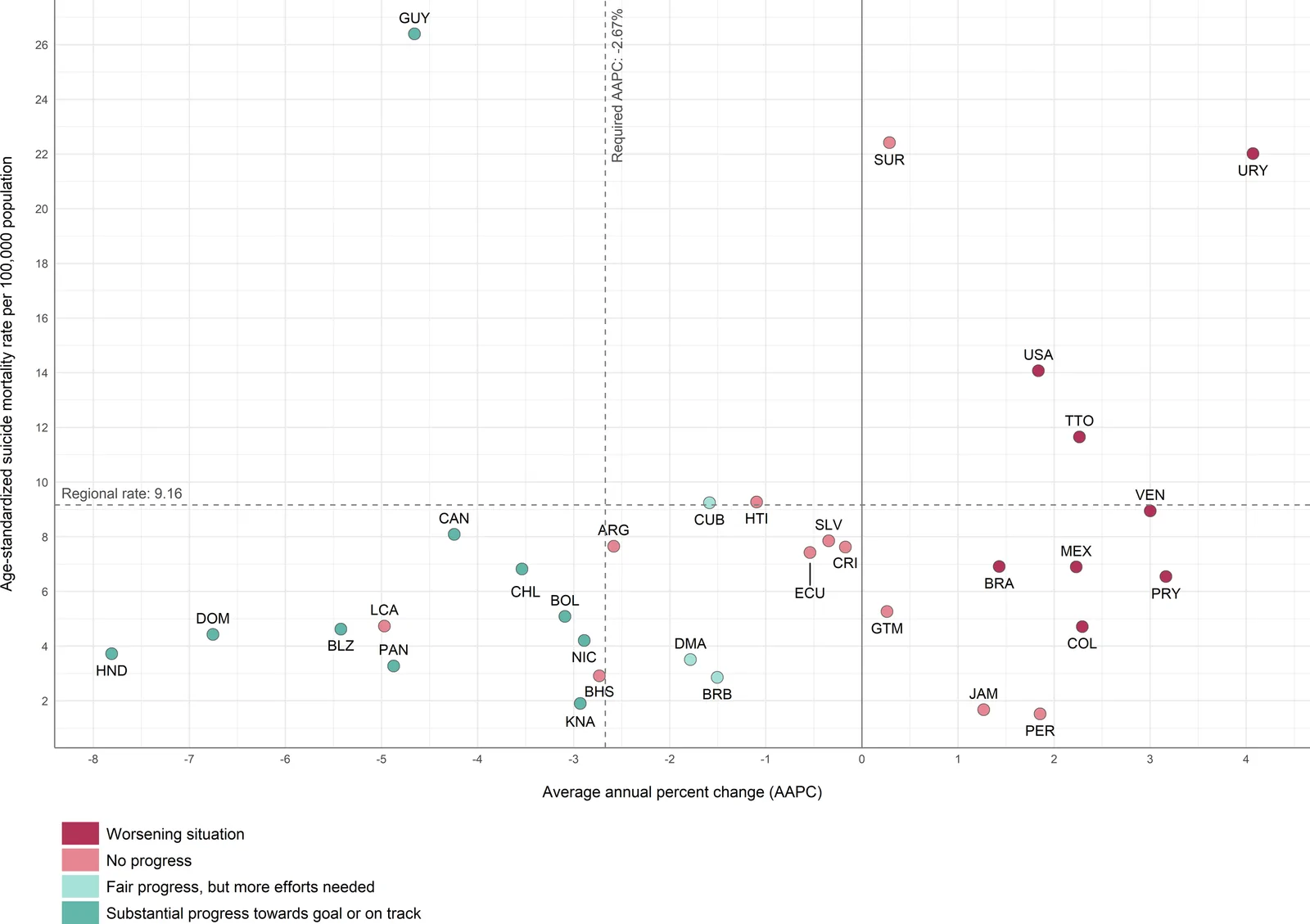
Age-standardized suicide mortality rate per 100,000 population in 2021 and the average annual percentage change between 2015 and 2021 in 32 countries in the Region of the Americas by country (indicated by ISO3 country code). AAPC Average annual percentage change. *Note.* The vertical dotted line indicates an AAPC of −2·67%, representing the compound annual reduction required to achieve the one-third reduction target by 2030. The horizontal dotted line indicates the suicide mortality rate in the region in 2021. Grenada was excluded from this figure because its AAPC was an extreme outlier relative to the other countries. Inclusion of Grenada would have substantially expanded the x-axis scale and reduced the readability of the remaining country estimates.

As depicted in Fig. 1, there is a tendency for countries with higher suicide mortality rates in 2021 to exhibit near stagnant or worsening trends in their suicide mortality rate between 2015 and 2021 (i.e., an AAPC that is not statistically significant, or a statistically significant AAPC ≥0). In particular, among the seven countries (out of 33, 21·2%) with suicide mortality rates above the regional rate in 2021, Haiti, Suriname, Trinidad and Tobago, Uruguay, and the United States all had either relatively stable or worsening suicide mortality rate trends between 2015 and 2021. Conversely, nine out of 26 countries with suicide mortality rates below the regional rate in 2021 achieved significant average annual declines, with a statistically significant AAPC at or below the −2·67% threshold. Although Guyana had the highest rate in 2021 (26·39, 95% UI: 18·84, 35·96 per 100,000 population), it experienced a statistically significant decline between 2015 and 2021 (AAPC: −4·66, 95% CI: −6·80, −2·46), nearly twice the annual rate of decline required to meet the one-third reduction target (Table 1).

**Table 1:** Age-standardized suicide mortality rate in 2015 and 2021 and average annual percent change from 2015 to 2021 in the Region of the Americas by country and sub-region for both sexes combined.

Subregion and Country	ISO3 country code	WHO data-quality category	Age-standardized suicide mortality rate per 100,000 population		Estimated change in number of deaths, 2015–2021	AAPC		No. of joinpoints	Location of joinpoint
			2015	2021		2015–2021	p-value		
Andean area			4·53 (3·71–5·50)	5·05 (3·69–6·70)	1234	0·65 (−3·25, 4·71)	0·75	2	2014, 2017
Bolivia	BOL	Very low	6·28 (3·53–10·05)	5·08 (2·89–8·06)	−115	−3·09 (−4·41, −1·75)	<0·0001	2	2013, 2019
Colombia	COL	High	4·08 (3·60–4·58)	4·71 (3·71–5·84)	600	2·30 (0·34, 4·29)	0·021	2	2013, 2018
Ecuador	ECU	Medium	8·51 (7·21–9·91)	7·42 (5·68–9·54)	−29	−0·54 (−1·91, 0·85)	0·42	1	2006
Peru	PER	Low	0·96 (0·63–1·31)	1·52 (0·91–2·29)	215	1·85 (−0·34, 4·10)	0·094	0	NA
Venezuela	VEN	High	6·27 (5·25–7·45)	8·94 (6·39–12·04)	562	3·00 (0·97, 5·07)	0·0061	1	2009
Central America, Mexico and Latin Caribbean			6·67 (5·71–7·81)	6·80 (5·45–8·34)	1445	0·83 (0·52, 1·14)	<0·0001	1	2007
Costa Rica	CRI	High	6·16 (5·36–7·02)	7·62 (6·32–9·07)	101	−0·17 (−1·13, 0·80)	0·71	0	NA
Cuba	CUB	High	10·37 (9·22–11·61)	9·24 (7·45–11·26)	−53	−1·59 (−2·22, −0·95)	0·00011	2	2007, 2010
Dominican Republic	DOM	Low	6·83 (4·50–9·88)	4·42 (2·73–6·70)	−206	−6·75 (−9·46, −3·96)	<0·0001	2	2011, 2017
El Salvador	SLV	Low	11·92 (8·85–15·46)	7·85 (5·23–11·20)	−256	−0·34 (−1·26, 0·58)	0·44	0	NA
Guatemala	GTM	Medium	5·98 (5·17–6·86)	5·26 (4·16–6·52)	−2	0·26 (−1·14, 1·68)	0·70	2	2005, 2011
Haiti	HTI	Very low	9·89 (5·01–15·78)	9·27 (4·85–14·79)	33	−1·09 (−2·37, 0·19)	0·10	2	2006, 2019
Honduras	HND	Very low	7·16 (4·01–11·85)	3·72 (2·14–6·00)	−295	−7·81 (−11·33, −4·15)	0·00052	2	2010, 2013
Mexico	MEX	High	5·69 (5·43–5·95)	6·89 (5·95–7·88)	2099	2·23 (1·93, 2·53)	<0·0001	0	NA
Nicaragua	NIC	High	4·86 (3·68–6·16)	4·20 (2·87–5·86)	−8	−2·89 (−3·43, −2·34)	<0·0001	0	NA
Panama	PAN	High	2·86 (2·43–3·33)	3·27 (2·44–4·18)	33	−4·87 (−5·81, −3·92)	<0·0001	1	2003
Non-Latin Caribbean			9·31 (7·31–11·63)	8·12 (5·75–11·07)	−43	−1·58 (−4·22, 1·13)	0·25	2	2006, 2019
Antigua and Barbuda	ATG	Medium	0·42 (0·33–0·52)	1·40 (1·11–1·72)	1	–	–	–	–
Bahamas	BHS	Medium	3·16 (2·51–3·93)	2·91 (2·13–3·88)	0	−2·73 (−5·95, 0·60)	0·11	1	2017
Barbados	BRB	High	3·49 (2·79–4·31)	2·85 (2·04–3·82)	−1	−1·51 (−2·06, −0·95)	<0·0001	0	NA
Belize	BLZ	High	6·53 (5·41–7·83)	4·62 (3·66–5·74)	−4	−5·42 (−7·14, −3·67)	<0·0001	2	2008, 2011
Dominica	DMA	Medium	3·81 (2·51–5·37)	3·50 (2·24–5·22)	0	−1·78 (−3·23, −0·32)	0·020	1	2014
Grenada	GRD	High	0·50 (0·41–0·60)	1·17 (0·93–1·46)	1	18·48 (−21·26, 78·26)	0·42	2	2004, 2018
Guyana	GUY	Medium	35·63 (27·71–44·86)	26·39 (18·84–35·96)	−50	−4·66 (−6·80, −2·46)	0·00050	2	2008, 2015
Jamaica	JAM	High	1·69 (1·33–2·10)	1·67 (1·17–2·31)	3	1·27 (−0·77, 3·35)	0·21	0	NA
Saint Kitts and Nevis	KNA	Medium	2·36 (1·82–3·02)	1·90 (1·33–2·64)	0	−2·93 (−3·70, −2·16)	<0·0001	2	2006, 2012
Saint Lucia	LCA	High	7·22 (5·90–8·73)	4·73 (3·62–6·07)	−4	−4·97 (−10·05, 0·40)	0·069	1	2019
Saint Vincent and the Grenadines	VCT	High	2·65 (2·12–3·24)	0·29 (0·23–0·36)	−2	–	–	–	–
Suriname	SUR	Medium	27·27 (19·98–35·94)	22·41 (14·90–31·79)	−14	0·29 (−0·06, 0·64)	0·10	0	NA
Trinidad and Tobago	TTO	High	10·69 (8·95–12·68)	11·65 (8·41–15·58)	29	2·27 (0·96, 3·59)	0·0022	2	2005, 2012
North America			12·59 (12·11–13·03)	13·46 (12·68–14·19)	4801	0·95 (0·21, 1·70)	0·012	2	2006, 2018
Canada	CAN	High	11·59 (10·54–12·67)	8·08 (7·08–9·16)	−1198	−4·24 (−7·98, −0·35)	0·033	1	2019
United States of America	USA	High	12·71 (12·29–13·08)	14·07 (13·32–14·75)	5998	1·84 (1·64, 2·04)	<0·0001	1	2006
Southern Cone and Brazil			6·64 (6·11–7·17)	7·20 (6·51–7·92)	3210	1·17 (0·65, 1·69)	0·00017	1	2010
Argentina	ARG	Medium	8·56 (7·55–9·62)	7·65 (6·53–8·81)	−223	−2·58 (−7·17, 2·23)	0·29	2	2007, 2019
Brazil	BRA	High	5·81 (5·49–6·12)	6·91 (6·42–7·39)	3562	1·43 (1·20, 1·66)	<0·0001	0	NA
Chile	CHL	High	9·40 (8·48–10·38)	6·82 (5·89–7·79)	−363	−3·54 (−4·36, −2·71)	<0·0001	2	2005, 2008
Paraguay	PRY	High	6·60 (4·43–8·69)	6·54 (4·11–9·57)	22	3·16 (2·49, 3·84)	<0·0001	0	NA
Uruguay	URY	Medium	16·76 (14·83–18·84)	22·01 (18·97–25·32)	211	4·07 (2·64, 5·52)	<0·0001	1	2009
Region of the Americas			8·75 (8·09–9·44)	9·16 (8·20–10·18)	10,646	1·05 (0·85, 1·26)	<0·0001	1	2006

AAPC Average annual percent change; – Zeros present in the time-series (AAPC not estimated); NA Not applicable.

*Note:* AAPCs represent model-based estimates of temporal change derived from the fitted log-linear joinpoint regression using all annual observations over the specified interval and therefore may differ from changes calculated directly from the observed values at the beginning and end of the interval. Country-specific trends are presented in the Supplementary Material, Figure S3.

A total of 10 countries (out of 33, 30·3%) in the region experienced average annual declines in their suicide mortality rate exceeding −2·67% and were classified as making substantial progress towards the one-third reduction target. These countries are primarily located in the bottom-left quadrant of Fig. 1, characterized by both low suicide mortality rates in 2021 and steep declines between 2015 and 2021. Three countries (out of 33, 9·1%), namely Barbados, Cuba, and Dominica showed significant declines at a lower level than required and were classified as making fair progress towards the reduction target. Twelve countries (out of 33, 36·4%) did not demonstrate statistically significant AAPCs in suicide mortality between 2015 and 2021 and were therefore classified as making no progress according to the predefined classification criteria. Eight countries (out of 33, 24·2%) experienced significant increases in suicide mortality, indicating a worsening situation (Brazil, Colombia, Mexico, Paraguay, Trinidad and Tobago, United States of America, Uruguay, and Venezuela).

At the regional level, the suicide mortality rate increased among males by an average of 0·99% per year (95% CI: 0·81%, 1·17%) between 2015 and 2021, while rates among females were relatively stable (AAPC: 0·02, 95% CI: −1·38%, 1·45%; Supplementary Tables S1 and S2). Although the overall pattern of higher-than-average regional rates accompanied by stable or increasing trends and lower-than-average regional rates accompanied by decreasing trends, was evident among both males and females, some countries exhibited sex-specific differences (Supplementary Figures S4 and S5). Specifically, 17 (out of 32, 53·1%) countries had different sex-specific trends between 2015 and 2021 (Table 2). Among these countries, progress classifications indicated greater progress among males in seven countries and among females in 10 countries. Notably, substantial progress toward the one-third reduction target among females was observed in Bolivia, Canada, Ecuador, and Saint Lucia, whereas no progress was observed among males in these countries.

**Table 2:** Average annual percent change in the age-standardized suicide mortality rate from 2015 to 2021 in the Region of the Americas by country and sub-region for males and females.

Subregion and Country	Males		Females	
	AAPC		AAPC	
	2015–2021	p-value	2015–2021	p-value
Andean area	1·12 (−2·67, 5·05)	0·57	−1·52 (−6·19, 3·37)	0·54
Bolivia	−0·10 (−0·70, 0·50)	0·72	−8·38 (−9·44, −7·32)	<0·0001
Colombia	1·79 (−0·49, 4·12)	0·12	4·69 (2·85, 6·56)	<0·0001
Ecuador	0·36 (−0·95, 1·69)	0·57	−3·06 (−4·34, −1·75)	0·00014
Peru	2·17 (−0·08, 4·48)	0·058	1·17 (−1·26, 3·67)	0·33
Venezuela	3·19 (1·07, 5·35)	0·0053	2·05 (0·36, 3·77)	0·020
Central America, Mexico and Latin Caribbean	0·92 (0·52, 1·32)	0·00013	0·75 (0·36, 1·15)	0·00085
Costa Rica	−0·55 (−1·53, 0·44)	0·26	6·18 (0·86, 11·78)	0·025
Cuba	−0·95 (−1·64, −0·26)	0·011	−6·08 (−10·05, −1·95)	0·0043
Dominican Republic	−6·99 (−9·99, −3·88)	<0·0001	−4·65 (−7·39, −1·82)	0·0014
El Salvador	−0·04 (−1·07, 1·00)	0·93	−0·98 (−1·55, −0·40)	0·0020
Guatemala	−0·11 (−1·64, 1·45)	0·88	1·30 (0·10, 2·52)	0·036
Haiti	−0·58 (−1·85, 0·71)	0·38	−1·86 (−3·78, 0·10)	0·063
Honduras	−8·28 (−12·28, −4·09)	0·0010	−4·56 (−8·39, −0·58)	0·025
Mexico	2·54 (2·04, 3·04)	<0·0001	3·32 (2·82, 3·82)	<0·0001
Nicaragua	−2·55 (−3·19, −1·91)	<0·0001	−5·12 (−6·57, −3·65)	<0·0001
Panama	−4·88 (−5·86, −3·89)	<0·0001	−3·90 (−5·85, −1·91)	0·00063
Non-Latin Caribbean	−1·70 (−4·80, 1·51)	0·30	−2·18 (−3·95, −0·38)	0·02
Antigua and Barbuda	–	–	–	–
Bahamas	−2·74 (−5·81, 0·42)	0·089	−0·85 (−1·44, −0·25)	0·0076
Barbados	−1·70 (−2·29, −1·11)	<0·0001	−0·40 (−0·88, 0·08)	0·095
Belize	−5·67 (−7·67, −3·62)	<0·0001	−3·56 (−4·76, −2·35)	<0·0001
Dominica	−0·40 (−0·70, −0·10)	0·012	−0·51 (−0·92, −0·11)	0·015
Grenada	–	–	–	–
Guyana	−4·12 (−6·34, −1·86)	0·0017	−5·96 (−9·23, −2·57)	0·00066
Jamaica	1·41 (−0·75, 3·62)	0·19	1·04 (0·18, 1·91)	0·020
Saint Kitts and Nevis	−3·28 (−4·24, −2·32)	<0·0001	−3·53 (−4·68, −2·37)	<0·0001
Saint Lucia	−5·26 (−10·27, 0·03)	0·051	−3·08 (−4·32, −1·82)	0·00013
Saint Vincent and the Grenadines	–	–	–	–
Suriname	0·08 (−0·41, 0·57)	0·74	1·05 (0·80, 1·30)	<0·0001
Trinidad and Tobago	2·97 (1·31, 4·65)	0·0014	−1·28 (−3·09, 0·57)	0·17
North America	1·49 (1·32, 1·66)	<0·0001	−0·56 (−1·69, 0·57)	0·33
Canada	−4·07 (−7·97, 0·01)	0·050	−4·89 (−9·06, −0·53)	0·028
United States of America	1·69 (1·49, 1·88)	<0·0001	−0·22 (−1·47, 1·04)	0·73
Southern Cone and Brazil	0·69 (0·39, 0·99)	0·00016	3·21 (1·39, 5·07)	0·00052
Argentina	−3·00 (−7·25, 1·44)	0·18	0·29 (−0·48, 1·07)	0·43
Brazil	1·25 (1·01, 1·49)	<0·0001	4·73 (3·18, 6·31)	<0·0001
Chile	−3·88 (−4·86, −2·89)	<0·0001	−3·46 (−4·56, −2·35)	<0·0001
Paraguay	−1·85 (−10·21, 7·28)	0·68	2·26 (1·25, 3·29)	0·00014
Uruguay	4·14 (2·63, 5·68)	<0·0001	1·61 (0·81, 2·41)	0·00042
Region of the Americas	0·99 (0·81, 1·17)	<0·0001	0·02 (−1·38, 1·45)	0·97

AAPC Average annual percent change; – Zeros present in the time-series (AAPC not estimated).

Age group-specific analyses produced findings that were broadly consistent with those based on age-standardized suicide mortality rates. At the regional level, suicide mortality increased significantly among individuals aged ≤24 years (AAPC: 2·66%, 95% CI: 2·23%, 3·09%), 25–49 years (1·19%, 95% CI: 0·76%, 1·63%), and ≥75 years (0·75%, 95% CI: 0·47%, 1·03%), decreased among those aged 50–64 years (−1·52%, 95% CI: −2·99%, −0·04%), and remained stable among those aged 65–74 years (−0·06%, 95% CI: −1·38%, 1·28%). Although the magnitude of the AAPCs varied across age groups, and some countries exhibited age-specific differences in trend direction or statistical significance, the overall pattern of progress toward the suicide mortality reduction target remained largely unchanged, indicating that the primary findings were robust across age groups. At the country-level, however, declines in suicide mortality were generally greatest among individuals aged ≤24 years, whereas trends were less favourable in older age groups, particularly among those ≥75 years, highlighting heterogeneity between regional and country-specific age patterns and suggesting important age-related heterogeneity in progress toward the target (Supplementary Tables S3–S5).

In the exploratory analysis assessing potential bias related to variation in country-level data quality, there was no evidence of an association between WHO data quality category and progress classification in the ordinal logistic regression analyses (Supplementary Table S6). Similarly, Fisher's exact tests did not identify statistically significant associations between data quality and progress categories (both sexes: p = 0·30; males: p = 0·65; females: p = 0·22). However, these findings should be interpreted cautiously given the relatively small number of countries distributed across multiple data-quality and progress categories, which limited the ability to detect potential associations. Accordingly, progress classifications for countries with low or very low data quality should be considered provisional and interpreted with particular caution. The data quality category for each country is presented in Table 1.

In general, among the subset of countries with available suicide method data, the proportion of both poisoning- and firearm-involved suicides declined between 2015 and 2021, whereas the proportion of suicides involving hanging increased during this period, regardless of overall progress towards the one-third reduction target (Table 3). For example, the proportion of suicides involving poisoning declined in several countries, including Canada (AAPC: −3·42%, 95% CI: −4·88%, −1·94%), Ecuador (−6·79%, 95% CI: −9·52%, −3·98%), Guatemala (−6·08%, 95% CI: −9·41%, −2·63%), Mexico (−8·16%, 95% CI: −12·20%, −3·92%), Nicaragua (−2·32%, 95% CI: −3·62%, −0·99%), and the United States of America (−4·79%, 95% CI: −5·61%, −3·96%). Similarly, the proportion of suicides involving a firearm significantly decreased in nine countries (out of 20, 45·0%): Argentina (AAPC: −6·65%, 95% CI: −10·35%, −2·78%), Brazil (−3·27%, 95% CI: −4·39%, −2·15%), Canada (−1·65%, 95% CI: −3·09%, −0·17%), Chile (−9·12%, 95% CI: −13·83%, −4·15%), Colombia (−8·29, 95% CI: −9·53%, −7·02%), Ecuador (−7·12%, 95% CI: −11·44%, −2·60), Guatemala (−11·42%, 95% CI: −17·79%, −4·55%), Mexico (−5·58%, 95% CI: −7·53%, −3·59%), and Nicaragua (−13·87%, 95% CI: −24·91%, −1·21%). However, the USA was a notable exception, where the proportion of suicides involving a firearm increased significantly between 2015 and 2021 (AAPC: 1·40%, 95% CI: 0·16%, 2·66%), rising from 49·88% in 2015 to 54·68% in 2021 despite the concurrent decline in the proportion of poisoning-related suicides. There was also a significant increase in the proportion of suicide involving a firearm in Peru (AAPC: 18·99, 95% CI: 5·19%, 34·60%), although the proportion remained relatively low in 2021 (3·79%; from 1·52% in 2015). For the trends over time for the proportion of suicide by method see Supplementary Figure S6.

**Table 3:** Average annual percent change in the proportion of suicides by method for 20 countries in the Region of the Americas for both sexes combined.

Country; suicide method	Progress toward the one-third reduction target by 2030	Proportion (%)		AAPC (95% CI)		No. of joinpoints	Location of joinpoint
		2015	2021	2015–2021	p-value		
Argentina	No progress						
Firearms		19·12	13·86	−6·65 (−10·35, −2·78)	0·0073	0	NA
Hanging		72·95	77·69	1·51 (0·34, 2·70)	0·021	0	NA
Jumping		0·81	1·19	2·89 (−16·14, 26·23)	0·73	0	NA
Poisoning		1·64	2·09	−0·58 (−9·30, 8·98)	0·88	0	NA
Other		5·48	5·17	−2·08 (−6·64, 2·71)	0·31	0	NA
Belize	Substantial progress						
Firearms		11·54	8·33	0·85 (−18·81, 25·27)	0·92	0	NA
Hanging		65·38	77·78	3·75 (−1·42, 9·19)	0·12	0	NA
Jumping		0·00	0·00	–	–	–	–
Poisoning		23·08	11·11	−15·04 (−30·50, 3·86)	0·091	0	NA
Other		0·00	2·78	–	–	–	–
Brazil	Worsening situation						
Firearms		8·10	6·80	−3·27 (−4·39, −2·15)	0·00071	0	NA
Hanging		68·24	71·51	1·01 (0·56, 1·46)	0·0021	0	NA
Jumping		4·22	4·06	−1·04 (−5·41, 3·53)	0·65	1	2019
Poisoning		11·69	10·85	−1·66 (−3·49, 0·21)	0·072	0	NA
Other		7·75	6·78	−2·71 (−4·10, −1·29)	0·0046	0	NA
Canada	Substantial progress						
Firearms		14·38	13·52	−1·65 (−3·09, −0·17)	0·035	0	NA
Hanging		48·68	51·91	1·41 (0·38, 2·44)	0·017	0	NA
Jumping		5·41	6·24	1·63 (0·21, 3·08)	0·032	0	NA
Poisoning		22·08	18·14	−3·42 (−4·88, −1·94)	0·0020	0	NA
Other		9·45	10·20	1·21 (0·30, 2·13)	0·019	0	NA
Chile	Substantial progress						
Firearms		5·17	2·99	−9·12 (−13·83, −4·15)	0·0057	0	NA
Hanging		83·72	88·80	1·05 (0·46, 1·65)	0·00046	1	2018
Jumping		2·12	1·27	−7·93 (−17·44, 2·68)	0·11	0	NA
Poisoning		5·61	3·76	−7·29 (−17·73, 4·48)	0·21	1	2018
Other		3·38	3·18	−2·74 (−10·75, 5·98)	0·44	0	NA
Colombia	Worsening situation						
Firearms		13·77	8·17	−8·29 (−9·53, −7·02)	<0·0001	0	NA
Hanging		56·18	64·10	2·12 (0·68, 3·58)	0·0039	1	2018
Jumping		4·94	5·25	1·86 (−2·80, 6·73)	0·36	0	NA
Poisoning		20·97	16·25	−3·49 (−7·39, 0·58)	0·078	0	NA
Other		4·13	6·24	3·98 (−3·70, 12·27)	0·25	0	NA
Costa Rica	No progress						
Firearms		10·79	9·95	−1·76 (−7·71, 4·58)	0·50	0	NA
Hanging		63·17	69·63	0·68 (−1·13, 2·52)	0·38	0	NA
Jumping		3·49	1·83	−9·18 (−22·52, 6·47)	0·24	1	2019
Poisoning		20·00	14·66	−0·80 (−8·29, 7·30)	0·80	0	NA
Other		2·54	3·93	1·62 (−10·36, 15·19)	0·76	0	NA
Cuba	Fair progress						
Firearms		1·06	1·11	−2·12 (−8·62, 4·85)	0·46	0	NA
Hanging		76·47	81·87	1·01 (0·73, 1·29)	0·00023	0	NA
Jumping		2·98	3·56	1·26 (−9·26, 12·99)	0·78	0	NA
Poisoning		9·01	6·67	−3·97 (−8·88, 1·20)	0·10	0	NA
Other		10·47	6·79	−5·69 (−11·21, 0·18)	0·055	0	NA
Dominican Republic	Substantial progress						
Firearms		15·32	9·22	−9·93 (−26·32, 10·11)	0·24	0	NA
Hanging		59·04	62·59	1·62 (−2·34, 5·74)	0·35	0	NA
Jumping		0·34	1·77	–	–	–	–
Poisoning		15·83	19·33	2·91 (−6·50, 13·27)	0·48	0	NA
Other		9·47	7·09	−5·05 (−23·10, 17·25)	0·56	0	NA
Ecuador	No progress						
Firearms		5·59	3·41	−7·12 (−11·44, −2·60)	0·010	0	NA
Hanging		69·25	78·52	2·53 (1·52, 3·55)	0·0013	0	NA
Jumping		1·03	1·25	−1·58 (−20·69, 22·13)	0·86	0	NA
Poisoning		21·34	14·89	−6·79 (−9·52, −3·98)	0·0017	0	NA
Other		2·80	1·93	−7·28 (−15·44, 1·66)	0·088	0	NA
Guatemala	No progress						
Firearms		8·95	3·64	−11·42 (−17·79, −4·55)	0·0087	0	NA
Hanging		40·27	56·08	5·45 (3·16, 7·80)	0·0016	0	NA
Jumping		0·19	0·00	–	–	–	–
Poisoning		46·69	34·27	−6·08 (−9·41, −2·63)	0·0066	0	NA
Other		3·89	6·01	12·09 (−6·59, 34·51)	0·17	0	NA
Guyana^a^	Substantial progress						
Firearms		0·90	1·03	–	–	–	–
Hanging		32·58	39·18	5·22 (0·62, 10·03)	0·034	0	NA
Jumping		0·90	0·00	–	–	–	–
Poisoning		64·71	58·76	−2·61 (−5·28, 0·13)	0·057	0	NA
Other		0·90	1·03	10·54 (−15·53, 44·64)	0·36	0	NA
Mexico	Worsening situation						
Firearms		8·94	6·05	−5·58 (−7·53, −3·59)	0·00087	0	NA
Hanging		79·27	85·34	1·22 (0·72, 1·72)	0·0015	0	NA
Jumping		0·81	0·71	−2·31 (−8·23, 4·00)	0·38	0	NA
Poisoning		7·91	5·18	−8·16 (−12·20, −3·92)	0·0047	0	NA
Other		3·07	2·72	−0·21 (−6·15, 6·10)	0·93	0	NA
Nicaragua	Substantial progress						
Firearms		5·71	2·36	−13·87 (−24·91, −1·21)	0·038	0	NA
Hanging		33·97	45·86	5·06 (2·97, 7·19)	0·0015	0	NA
Jumping		0·32	0·24	–	–	–	–
Poisoning		57·46	49·17	−2·32 (−3·62, −0·99)	0·0065	0	NA
Other		2·54	2·36	−6·87 (−18·76, 6·77)	0·24	0	NA
Panama	Substantial progress						
Firearms		4·55	0·77	−21·13 (−40·90, 5·26)	0·088	0	NA
Hanging		67·27	76·15	1·17 (−2·65, 5·15)	0·47	0	NA
Jumping		2·73	3·08	–	–	–	–
Poisoning		25·45	18·46	−2·40 (−20·15, 19·31)	0·77	0	NA
Other		0·00	1·54	–	–	–	–
Paraguay	Worsening situation						
Firearms		16·23	11·67	−6·91 (−14·62, 1·50)	0·087	0	NA
Hanging		72·77	80·31	2·20 (0·08, 4·36)	0·044	0	NA
Jumping		0·79	1·39	6·18 (−19·13, 39·40)	0·67	1	2017
Poisoning		5·24	3·83	−5·66 (−10·83, −0·19)	0·043	1	2017
Other		4·97	2·79	−7·67 (−20·07, 6·67)	0·21	0	NA
Peru	No progress						
Firearms		1·52	3·79	18·99 (5·19, 34·60)	0·015	0	NA
Hanging		47·73	53·73	4·08 (−4·99, 14·01)	0·31	0	NA
Jumping		0·76	1·52	–	–	–	–
Poisoning		45·83	32·62	−6·87 (−18·92, 6·99)	0·24	0	NA
Other		4·17	8·34	3·19 (−21·79, 36·15)	0·78	0	NA
Trinidad and Tobago^a^	Worsening situation						
Firearms		1·45	1·65	0·93 (−29·65, 44·80)	0·95	0	NA
Hanging		47·83	46·28	−2·01 (−5·80, 1·92)	0·22	0	NA
Jumping		0·00	1·65	–	–	–	–
Poisoning		50·00	49·59	1·04 (−2·19, 4·37)	0·43	0	NA
Other		0·72	0·83	12·66 (−33·83, 91·81)	0·57	0	NA
United States of America	Worsening situation						
Firearms		49·88	54·68	1·40 (0·16, 2·66)	0·027	1	2019
Hanging		26·85	25·82	−0·35 (−3·77, 3·20)	0·85	1	2019
Jumping		2·28	2·46	0·57 (−0·99, 2·14)	0·39	0	NA
Poisoning		15·44	11·56	−4·79 (−5·61, −3·96)	<0·0001	0	NA
Other		5·55	5·48	0·09 (−0·86, 1·05)	0·83	0	NA
Uruguay	Worsening situation						
Firearms		23·64	18·82	−2·98 (−6·28, 0·45)	0·075	0	NA
Hanging		63·45	67·84	1·19 (−0·26, 2·66)	0·088	0	NA
Jumping		1·71	2·22	1·73 (−6·85, 11·11)	0·64	0	NA
Poisoning		2·49	4·71	5·70 (−4·43, 16·90)	0·22	0	NA
Other		8·71	6·41	−5·07 (−10·52, 0·71)	0·073	0	NA

AAPC Average annual percent change; – Zeros present in the time-series (AAPC not estimated); NA Not applicable (no joinpoints identified).

*Note:* AAPCs represent model-based estimates of temporal change derived from the fitted log-linear joinpoint regression using all annual observations over the specified interval and therefore may differ from changes calculated directly from the observed values at the beginning and end of the interval. Country-specific trends are presented in the Supplementary Material, Figure S6.

a Time-series: 2015–2020; data for 2021 not available.

It should be noted that, by 2021, hanging was the predominant method of suicide in 16 countries (out of 20, 80·0%). Hanging accounted for more than 80% of suicides in countries such as Chile (88·80%), Cuba (81·87%), Mexico (85·34%), and Paraguay (80·31%). The proportion of suicides involving hanging increased significantly in 12 countries (out of 20, 60·0%). In some countries, the increase was particularly pronounced among females, such as Cuba (AAPC: 2·65%, 95% CI: 0·05%, 5·32%) and Nicaragua (8·82%, 95% CI: 3·24%, 14·69%), compared with males (Cuba: 0·50%, 95% CI: 0·11%, 0·88%; Nicaragua: 4·20%, 95% CI: 1·63%, 6·84%; Supplementary Tables S7 and S8). Although increases in the proportion of suicides involving hanging were observed for both males and females across the subset of countries with available data, the magnitude of the increase was often greater among females (Fig. 2).

**Fig. 2:**
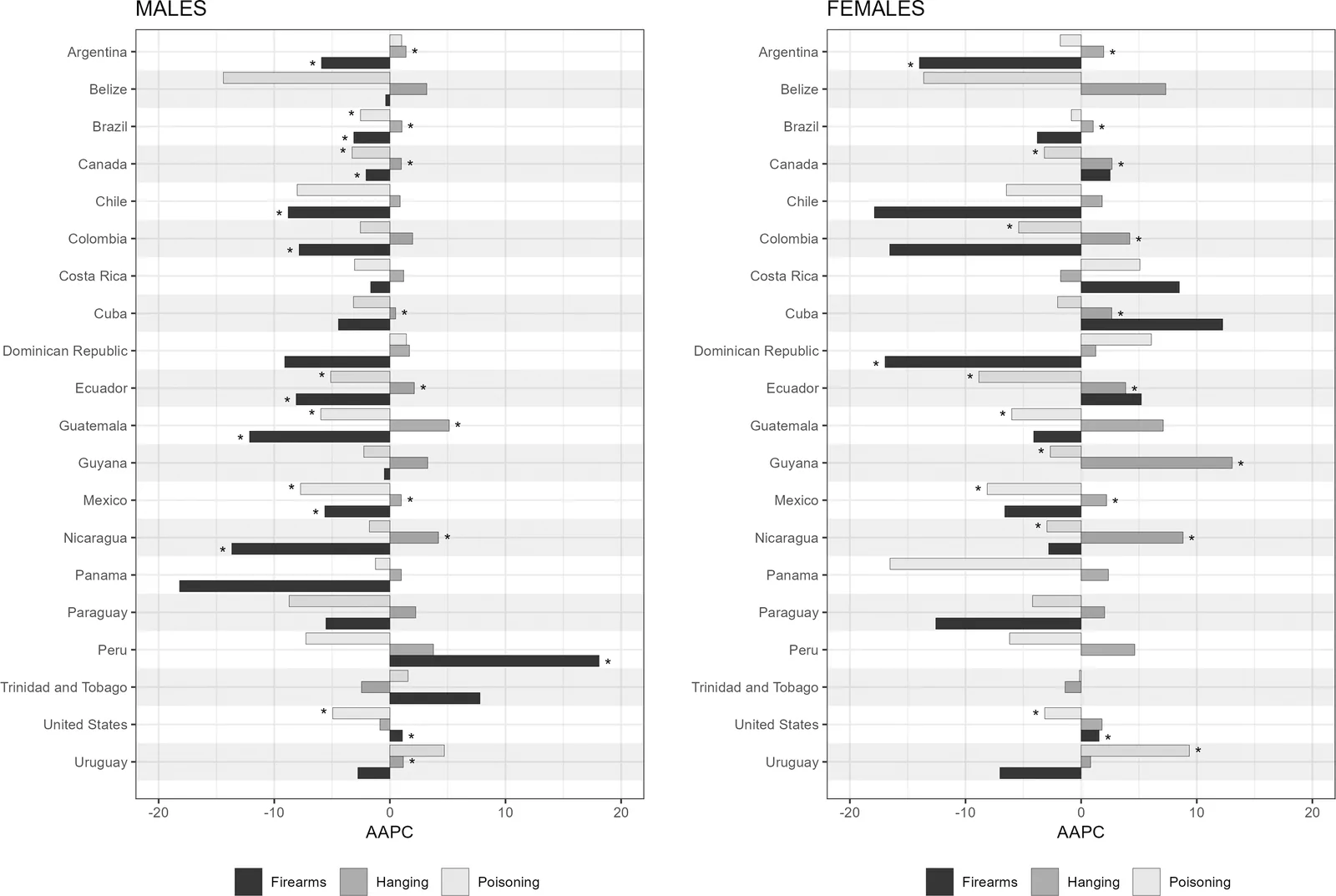
Average annual percentage change in the proportions of suicides involving firearms, hanging and poisoning among males and females in 20 countries in the Region of the Americas by country, 2015–2021. AAPC Average annual percentage change. *Note.* Asterisks (∗) indicate statistically significant AAPCs (p < 0·05; i.e., AAPCs for which the 95% confidence interval does not include zero). Estimates for Guyana and Trinidad and Tobago are based on data from 2015 to 2020 (data for 2021 were unavailable).

Importantly, these method-specific trends were broadly consistent regardless of countries’ progress toward the one-third reduction target. Both countries making substantial progress (e.g., Canada and Chile) and those with worsening suicide mortality (e.g., Brazil and Mexico) showed increasing proportions of suicide involving hanging and declining proportions involving poisoning and firearms. Changes in jumping and other methods were smaller, less consistent, and frequently not statistically significant.

### Sensitivity analyses

Compared with the model-based parametric CIs, empirically obtained CIs were generally narrower, with only minor changes in statistical significance for a small number of country-specific estimates (Supplementary Material, Tables S9–S11). Under the empirical-quantile CIs, the number of countries classified as not on track decreased from 23 to 21. The progress classification changed from no progress to fair progress for Argentina and Haiti, and from no progress to substantial progress for the Bahamas and Saint Lucia (Supplementary Material, Table S12). Compared with the primary analysis, excluding the COVID-19 pandemic years generally resulted in less favourable (more positive) AAPC estimates, suggesting that suicide mortality patterns during 2020–2021 modestly enhanced the estimated rate of progress toward reductions in suicide mortality. The number of countries classified as not on track increased from 23 to 26 when 2020–2021 were excluded. Although the overall regional pattern of progress was broadly similar, progress classifications changed for 10 out of 33 countries (30·3%), indicating some sensitivity of country-level classifications to the inclusion of the pandemic years (Supplementary Material, Tables S13–S15). Progress classification became less favourable for eight countries: from no progress to worsening for Ecuador, Guatemala, and Suriname; from substantial to fair progress for Bolivia and Nicaragua; from substantial progress to no progress for Canada and the Dominican Republic; and from fair progress to no progress for Dominica. Conversely, classifications became more favourable for two countries, changing from no progress to fair for Saint Lucia and to substantial for Grenada. The results of the log-linear model for the proportion of suicides by method yielded results consistent with the primary analyses. Estimated AAPCs were nearly identical, with only minor differences in confidence intervals. Five of 21 country-specific trends changed statistical significance, but the direction and magnitude of the estimated trends remained largely unchanged, indicating that the overall conclusions were robust (Supplementary Material, Table S16).

## Discussion

This analysis reveals marked heterogeneity in suicide mortality rates and trends across countries in the Americas. Five countries (Haiti, Suriname, Trinidad and Tobago, United States of America, and Uruguay) had suicide mortality rates exceeding the regional rate of 9·16 per 100,000 population in 2021 and experienced stable or increasing trends between 2015 and 2021, representing a substantial and persistent public health burden. Conversely, five countries (Brazil, Colombia, Mexico, Paraguay, and Venezuela) with rates below the regional average experienced increasing suicide mortality trends. Together, these findings underscore the need for context-specific suicide prevention strategies that address both high-burden settings and countries experiencing worsening trends.

The observed declines in the proportion of suicides involving firearms and poisoning in many countries coincide with increases in the proportion involving hanging. Given that method-specific estimates in this analysis are compositional, with proportions constrained to sum to 100%, increases in the proportion of one method may occur as a consequence of declines in other methods and do not, on their own, demonstrate method substitution. These findings are therefore interpreted as changes in the distribution of suicide methods rather than definitive evidence of substitution. Nevertheless, similar shifts in method distribution have been reported in other regions and may reflect, among other factors, changes in method availability, accessibility, or preferences.^18^^,^^19^ These findings reinforce that suicide prevention requires comprehensive strategies, including improved access to mental health care, early identification of those at risk, and addressing social and structural determinants of suicide, and evidence-based means restriction approaches. Because the present analysis examined proportions rather than method-specific mortality rates, further research using population-based rates of suicide by method is needed to assess whether changes in method distribution reflect true changes in method-specific mortality or patterns consistent with method substitution.

The lack of alignment between trends in method distribution and progress toward a one-third reduction suggests that changes in overall suicide mortality reflect a broader set of determinants extending beyond changes in the distribution of suicide methods alone. Because method-specific findings are based on proportions, observed changes reflect shifts in the distribution of recorded suicide methods and should not be interpreted as changes in the absolute number of deaths by specific methods. This distinction is particularly important in countries experiencing increases in overall suicide mortality, where declines in the proportion of suicides involving a given method may occur despite stable or increasing numbers of deaths by that method. Countries achieving substantial reductions in suicide mortality did not necessarily experience more favourable shifts in method distribution, and vice versa, highlighting the importance of evaluating both overall mortality trends and changes in method distributions when assessing suicide prevention progress. Sex-specific analyses similarly demonstrated differences in method distributions, with increases in the proportion of suicides involving hanging among females occurring alongside declines in the proportion involving poisoning—a method historically more common among women. These findings suggest changing patterns in the distribution of suicide methods between males and females, which may have implications for targeted prevention strategies.^20^

In response to the concerning trend in the suicide mortality rate in the Region of the Americas, PAHO launched a new initiative in 2025 aimed at curbing the rising rate. The initiative aligns with the WHO's LIVE LIFE framework, which promotes strategies such as responsible media reporting, fostering emotional resilience in youth, ensuring early identification and care for those at risk, as well as means restriction.^21^ The initiative has three overarching objectives: 1) to improve the governance and coordination of suicide prevention efforts, 2) to strengthen the capacity of health systems to prevent and respond to suicidal behavior, and 3) to improve multisectoral collaboration in suicide prevention.^22^ The expected result of the initiative is to reduce the suicide burden across the Americas by strengthening national capacities to implement evidence-based, multi-sectorial, and person-centered suicide prevention interventions. In our previous comparative interrupted time-series analysis of data from nine countries within the Americas from 2000 to 2021,^9^ it was found that the implementation of a national strategy was associated with a gradual and sustained decrease in suicide mortality rates of 3·00% per year among males (95% CI: −5·28%, −0·66%) and 2·55% per year among females (95% CI: −4·62%, −0·44%; there was no significant difference between sexes). Thus, these findings highlight the importance of strengthening national suicide prevention strategies as part of comprehensive efforts to reduce suicide mortality, not only in the Region of the Americas but elsewhere as well.

Despite the availability of effective suicide prevention strategies, several key barriers continue to impede advancing suicide prevention in the Americas. These include limitations in self-harm and suicide surveillance systems, restricted availability and access to quality in-person and remote mental health services, insufficient multisectoral action to address the social and economic determinants of mental health and suicide, and lack of public awareness of mental health conditions and suicide, as well as the associated stigma. Addressing these barriers will be essential to translating national strategies into sustained reductions in suicide mortality. Suicide mortality is shaped by the interaction of population-level determinants with individual experiences of psychological distress, psychiatric morbidity, substance use disorders, trauma exposure, and acute suicidal crises. Gaps in timely access to mental health care, continuity of care following suicidal crises, and early identification of individuals at elevated risk represent important opportunities for prevention. Efforts to reduce suicide mortality should integrate population-level strategies with clinical approaches that support the identification, treatment, and ongoing care of individuals experiencing suicidal thoughts or behaviors. Finally, the heterogeneity observed in suicide mortality trends, suicide method distributions, and the magnitude of changes across countries highlight the need for context-specific interventions. The determinants contributing to suicide mortality may vary across countries and subregions of the Americas, including differences in social, economic, health-system, and environmental contexts, and may contribute to the variation in observed trends.

This study has several limitations that must be acknowledged. First, as an ecological study, the findings are subject to the ecological fallacy, and population-level associations should not be inferred to represent individual-level relationships or behaviors. Second, joinpoint regression analysis assumes log-linear trends and may not capture more complex temporal patterns, particularly over a relatively short period of observation, as was the case for the method-specific analyses. Because the models for suicide mortality rates were fitted over the full 2000–2021 time series, the 2015–2021 AAPCs were derived from trend segments identified using data outside this interval; in countries without a joinpoint after 2015, the AAPC therefore reflects the slope of a longer fitted segment beginning before 2015. Further, although the log-linear specification aligned with the objective of estimating relative changes in method proportions, it does not account for their bounded nature, which may be particularly relevant when a given method accounts for a high proportion of suicides. Third, the WHO GHE rely on nationally registered mortality data that vary in completeness, quality, collection, and reporting standards across countries, and suicide deaths may be under-reported or misclassified in some settings. Although we found no evidence that progress classification was associated with country-level data quality, differences in vital registration completeness, cause-of-death certification, and suicide classification remain important considerations when interpreting cross-country differences and temporal trends. Fourth, means-specific analyses were limited to 20 of 35 countries with available registered death data, disproportionately representing countries with better vital registration, with two countries contributing data only through 2020, and were based on proportions rather than method-specific mortality rates. Thus, observed shifts in method distributions, including the shift toward hanging, characterize this subset and may not be generalizable to the region as a whole. Fifth, the significance-based classification of country-level progress should also be interpreted with caution, as countries with wider CIs, reflecting greater uncertainty in the estimated AAPCs, may have been classified differently had additional data or more precise estimates been available. Accordingly, the classifications should be viewed as descriptive indicators of progress rather than definitive judgments of country performance. Sixth, analyses could not be stratified by race/ethnicity or socioeconomic characteristics because these data were unavailable in the GHE, potentially obscuring important disparities in suicide mortality trends. Additionally, the youngest age category (≤24 years) combines children, adolescents, and young adults, populations with distinct epidemiological profiles and risk factors for suicide, precluding assessment of potentially important differences within this broad age group. Seventh, no adjustment for multiple testing was applied because analyses were pre-specified and intended to characterize distinct, correlated temporal patterns rather than test a family of independent confirmatory hypotheses. Nevertheless, given the number of trends examined, some statistically significant findings may have occurred by chance and should be interpreted with caution. Finally, joinpoint analyses were based on GHE point estimates, and the resulting AAPC CIs therefore reflect uncertainty in the fitted temporal trends but do not incorporate the uncertainty associated with the underlying annual GHE mortality estimates. Consequently, particularly for countries with wide GHE UIs, the reported AAPC CIs may understate the total uncertainty surrounding the estimated trends. Despite these limitations, the data used represent the most comprehensive and comparable data available for assessing progress toward the one-third reduction target and evaluating trends in the distribution of suicide methods. Future research is needed to identify the factors driving the observed trends in suicide mortality and suicide methods.

In conclusion, suicide mortality in the Region of the Americas increased between 2015 and 2021, indicating that the region is not on track to achieve the one-third reduction target by 2030. Nevertheless, progress varied across countries, with 10 (30·3%) making substantial progress toward the target, while accelerated and sustained action will be required in the 23 countries that are not on track to achieve this target. Among the subset of countries with available data, the broadly consistent shift in the distribution of suicide methods, reflected by increasing proportions of suicides involving hanging and decreasing proportions involving firearms and poisoning, was observed irrespective of progress in overall suicide mortality. While these compositional findings cannot establish changes in method-specific mortality, they suggest that means restriction may represent one component of a broader, comprehensive approach to suicide prevention. In conjunction with the broader evidence base, strengthening national suicide prevention strategies, expanding access to mental health care, and addressing social and structural determinants may contribute to reducing suicide mortality across the Americas. Together, the findings underscore the importance of intensified, context-specific suicide prevention efforts, particularly in countries with high and increasing mortality.

## Contributors

SL, RM, and ROS conceived the idea of the study. RM reviewed the scientific literature and prepared a summary of evidence on the topic. RM prepared the datasets. RM and KG conducted the data analyses. SL and RM prepared the results, tables, and figures. SL and RM drafted the manuscript with contributions from KG, AJMH, and ROS. All authors contributed important intellectual content to the study and commented critically on the manuscript. All authors have full access to all the data in the study, accept accountability for the overall work, and the corresponding author has the final responsibility for submitting the manuscript for publication.

## Data sharing statement

All data used in this study are publicly available through the online ENLACE Results Tool (https://ais.paho.org/phip/viz/nmh_data_ghe_results_tool.asp) and the Burden of Suicide (https://www.paho.org/en/enlace/burden-suicide). Data on the proportion of suicides by method is available in the Means of suicide visualization (https://www.paho.org/en/enlace/means-suicide). All results generated by this study are included in this paper.

The analytical code used to analyze the data, together with the derived datasets required to reproduce the analyses, are openly available at https://doi.org/10.5683/SP4/GBUNXR. The original datasets are not redistributed; instead, citations and links to the original sources are provided.

## Declaration of interests

We declare no competing interest.
