# Supplementary material for: Suicide mortality and suicide method trends in the Region of the Americas, 2015–2021: a population-based time-series analysis of progress toward a one-third reduction target
Source: Lancet Reg Health Am. 2026 Oct 15;65:101671. doi: 10.1016/j.lana.2026.101671 (PMC13694981; doi:10.1016/j.lana.2026.101671)
Supplement: Supplementary file 1 [file mmc1.pdf]

Suicide mortality and suicide method trends in the Region of the Americas, 2015-2021: A population-based time-series analysis of progress toward a one-third reduction target

Supplementary Material

Table of Contents

Table S1. Age-standardized suicide mortality rate in 2015 and 2021 and average annual percent change from 2015 to 2021 in the Region of the Americas by country and sub-region for males 3

Table S2. Age-standardized suicide mortality rate in 2015 and 2021 and average annual percent change from 2015 to 2021 in the Region of the Americas by country and sub-region for females 5

Table S3. Suicide mortality rate by age-group in 2015 and 2021 and average annual percent change from 2015 to 2021 in the Region of the Americas by country and sub-region for both sexes combined 7

Table S4. Suicide mortality rate by age-group in 2015 and 2021 and average annual percent change from 2015 to 2021 in the Region of the Americas by country and sub-region for males..... 13

Table S5. Suicide mortality rate by age-group in 2015 and 2021 and average annual percent change from 2015 to 2021 in the Region of the Americas by country and sub-region for females 19

Table S6. Association between World Health Organization data quality category and progress classification 25

Table S7. Average annual percent change in the proportion of suicides by method for 20 countries in the Region of the Americas for males 26

Table S8. Average annual percent change in the proportion of suicides by method for 20 countries in the Region of the Americas for females 29

Table S9. Sensitivity analysis: Confidence intervals obtained after propagating uncertainty in the annual suicide mortality estimates through repeated simulation for both sexes combined 32

Table S10. Sensitivity analysis: Confidence intervals obtained after propagating uncertainty in the annual suicide mortality estimates through repeated simulation for males 34

Table S11. Sensitivity analysis: Confidence intervals obtained after propagating uncertainty in the annual suicide mortality estimates through repeated simulation for females 36

Table S12. Country-specific progress classifications for the primary analyses and sensitivity analyses..... 38

Table S13. Sensitivity analysis (excluding 2020 and 2021): Age-standardized suicide mortality rate in 2015 and 2019, and the average annual percent change from 2015 to 2019 in the Region of the Americas by country and sub-region for both sexes combined 40

Table S14. Sensitivity analysis (excluding 2020 and 2021): Age-standardized suicide mortality rate in 2015 and 2019, and the average annual percent change from 2015 to 2019 in the Region of the Americas by country and sub-region for males 42

Table S15. Sensitivity analysis (excluding 2020 and 2021): Age-standardized suicide mortality rate in 2015 and 2019, and the average annual percent change from 2015 to 2019 in the Region of the Americas by country and sub-region for females 44

Table S16. Sensitivity analysis: Average annual percent change in the proportion of suicides by method for 20 countries in the Region of the Americas for both sexes combined, males, and females, using a log-linear model with no joinpoint 46

Figure S1. Residual plots for suicide mortality rate models for (A) the Andean Area, (B) Central America, Mexico and Latin Caribbean, (C) Non-Latin Caribbean, (D) North America, (E) Southern Cone and Brazil and (F) the Region of the Americas..... 47

Figure S2. Autocorrelation function (ACF) plots for suicide mortality rate models for (A) the Andean Area, (B) Central America, Mexico and Latin Caribbean, (C) Non-Latin Caribbean, (D) North America, (E) Southern Cone and Brazil and (F) the Region of the Americas..... 68

Figure S3. Suicide mortality rate trends for (A) the Andean Area, (B) Central America, Mexico and Latin Caribbean, (C) Non-Latin Caribbean, (D) North America, (E) Southern Cone and Brazil and (F) the Region of the Americas by country 89

Figure S4. Age-standardized suicide mortality rate per 100,000 population among males in 2021 and average annual percentage change between 2015 and 2021 in 32 countries in the Region of the Americas (indicated by ISO3 country code)........ 100

Figure S5. Age-standardized suicide mortality rate per 100,000 population among females in 2021 and average annual percentage change between 2015 and 2021 in 32 countries in the Region of the Americas (indicated by ISO3 country code)........ 101

Figure S6. Trends for the proportion of suicides by method for (A) Both sexes, (B) Males and (C) Females by country 102

Table S1. Age-standardized suicide mortality rate in 2015 and 2021 and average annual percent change from 2015 to 2021 in the Region of the Americas by country and sub-region for males

Subregion and Country	ISO3 country code	Age-standardized suicide mortality rate per 100,000 population		Estimated change in number of deaths, 2015–2021	AAPC		No. of joinpoints	Location of joinpoint(s)
		2015	2021		2015-2021	p-value		
Andean Area		7·20 (5·95, 8·65)	8·47 (6·20, 11·20)	1,289	1·12 (-2·67, 5·05)	0·57	2	2014, 2017
Bolivia	BOL	8·37 (4·77, 13·24)	7·80 (4·50, 12·13)	-26	-0·10 (-0·70, 0·50)	0·72	1	2012
Colombia	COL	6·55 (5·80, 7·34)	7·66 (6·01, 9·52)	510	1·79 (-0·49, 4·12)	0·12	2	2013, 2018
Ecuador	ECU	13·12 (11·13, 15·23)	12·40 (9·49, 15·93)	81	0·36 (-0·95, 1·69)	0·57	1	2005
Peru	PER	1·25 (0·85, 1·68)	2·28 (1·40, 3·39)	190	2·17 (-0·08, 4·48)	0·058	0	NA
Venezuela	VEN	11·15 (9·33, 13·26)	16·22 (11·62, 21·82)	535	3·19 (1·07, 5·35)	0·0053	1	2009
Central America, Mexico and Latin Caribbean		10·95 (9·45, 12·77)	11·28 (9·14, 13·75)	1,258	0·92 (0·52, 1·32)	0·00013	1	2008
Costa Rica	CRI	10·20 (8·95, 11·56)	12·54 (10·44, 14·86)	84	-0·55 (-1·53, 0·44)	0·26	0	NA
Cuba	CUB	17·02 (15·20, 18·94)	16·18 (13·05, 19·69)	26	-0·95 (-1·64, -0·26)	0·011	2	2007, 2010
Dominican Republic	DOM	11·74 (7·78, 16·87)	7·44 (4·62, 11·24)	-187	-6·99 (-9·99, -3·88)	<0·0001	2	2010, 2017
El Salvador	SLV	22·16 (16·58, 28·69)	13·95 (9·35, 19·83)	-251	-0·04 (-1·07, 1·00)	0·93	0	NA
Guatemala	GTM	9·28 (8·04, 10·61)	7·95 (6·27, 9·86)	-10	-0·11 (-1·64, 1·45)	0·88	2	2005, 2011
Haiti	HTI	14·10 (7·83, 22·11)	13·56 (7·68, 21·23)	46	-0·58 (-1·85, 0·71)	0·38	2	2006, 2019
Honduras	HND	12·99 (7·37, 21·32)	6·62 (3·89, 10·47)	-286	-8·28 (-12·28, -4·09)	0·0010	2	2010, 2013
Mexico	MEX	9·37 (8·97, 9·78)	11·54 (9·99, 13·13)	1,791	2·54 (2·04, 3·04)	<0·0001	1	2007
Nicaragua	NIC	7·64 (5·90, 9·61)	6·92 (4·79, 9·56)	12	-2·55 (-3·19, -1·91)	<0·0001	0	NA
Panama	PAN	4·70 (4·02, 5·45)	5·64 (4·19, 7·23)	34	-4·88 (-5·86, -3·89)	<0·0001	1	2003
Non-Latin Caribbean		15·14 (11·98, 18·76)	13·15 (9·38, 17·82)	-34	-1·70 (-4·80, 1·51)	0·30	2	2011, 2019
Antigua and Barbuda	ATG	0·00 (0·00, 0·00)	1·64 (1·27, 2·09)	1	---	---	---	---
Bahamas	BHS	5·92 (4·69, 7·35)	5·37 (3·95, 7·13)	0	-2·74 (-5·81, 0·42)	0·089	1	2017
Barbados	BRB	6·32 (5·07, 7·80)	5·14 (3·67, 6·91)	-1	-1·70 (-2·29, -1·11)	<0·0001	0	NA
Belize	BLZ	11·45 (9·50, 13·71)	7·68 (6·07, 9·55)	-4	-5·67 (-7·67, -3·62)	<0·0001	2	2008, 2011
Dominica	DMA	6·92 (4·63, 9·71)	6·30 (3·96, 9·19)	0	-0·40 (-0·70, -0·10)	0·012	0	NA
Grenada	GRD	0·70 (0·58, 0·82)	1·74 (1·35, 2·17)	1	---	---	---	---
Guyana	GUY	57·31 (44·78, 71·95)	43·94 (31·46, 59·88)	-33	-4·12 (-6·34, -1·86)	0·0017	2	2008, 2015
Jamaica	JAM	3·13 (2·46, 3·92)	3·10 (2·17, 4·29)	2	1·41 (-0·75, 3·62)	0·19	0	NA
Saint Kitts and Nevis	KNA	4·32 (3·35, 5·45)	3·32 (2·41, 4·82)	0	-3·28 (-4·24, -2·32)	<0·0001	2	2006, 2013
Saint Lucia	LCA	13·68 (11·15, 16·48)	8·65 (6·61, 11·02)	-4	-5·26 (-10·27, 0·03)	0·051	1	2019
Saint Vincent and the Grenadines	VCT	4·66 (3·75, 5·73)	0·55 (0·45, 0·70)	-2	---	---	---	---
Suriname	SUR	41·89 (31·22, 53·76)	31·40 (21·30, 43·69)	-20	0·08 (-0·41, 0·57)	0·74	0	NA
Trinidad and Tobago	TTO	17·68 (14·85, 20·91)	19·60 (14·20, 26·12)	28	2·97 (1·31, 4·65)	0·0014	1	2012
North America		19·28 (18·56, 19·92)	21·27 (20·07, 22·38)	5,488	1·49 (1·32, 1·66)	<0·0001	1	2006
Canada	CAN	16·94 (15·44, 18·45)	12·10 (10·62, 13·69)	-819	-4·07 (-7·97, 0·01)	0·050	1	2019
United States of America	USA	19·54 (18·91, 20·09)	22·31 (21·13, 23·36)	6,307	1·69 (1·49, 1·88)	<0·0001	1	2006
Southern Cone and Brazil		10·72 (9·88, 11·56)	11·15 (10·09, 12·23)	1,916	0·69 (0·39, 0·99)	0·00016	1	2005
Argentina	ARG	14·07 (12·42, 15·81)	12·17 (10·41, 14·02)	-242	-3·00 (-7·25, 1·44)	0·18	2	2006, 2019
Brazil	BRA	9·30 (8·80, 9·78)	10·62 (9·88, 11·34)	2,310	1·25 (1·01, 1·49)	<0·0001	0	NA
Chile	CHL	15·04 (13·65, 16·50)	10·84 (9·41, 12·34)	-287	-3·88 (-4·86, -2·89)	<0·0001	2	2005, 2009
Paraguay	PRY	9·63 (6·55, 12·49)	8·45 (5·35, 12·32)	-25	-1·85 (-10·21, 7·28)	0·68	1	2019
Uruguay	URY	27·80 (24·77, 31·06)	35·99 (31·15, 41·29)	159	4·14 (2·63, 5·68)	<0·0001	1	2009
REGION OF THE AMERICAS		13·79 (12·78, 14·84)	14·66 (13·17, 16·24)	9,916	0·99 (0·81, 1·17)	<0·0001	1	2006

AAPC Average annual percent change; --- Zeros present in the time-series (AAPC not estimated); NA Not applicable
Note: AAPCs represent model-based estimates of temporal change derived from the fitted log-linear joinpoint regression using all annual observations over the specified interval and therefore may differ from changes calculated directly from the observed values at the beginning and end of the interval.

Table S2. Age-standardized suicide mortality rate in 2015 and 2021 and average annual percent change from 2015 to 2021 in the Region of the Americas by country and sub-region for females

Subregion and Country	ISO3 country code	Age-standardized suicide mortality rate per 100,000 population		Estimated change in number of deaths, 2015–2021	AAPC		No. of joinpoints	Location of joinpoint(s)
		2015	2021		2015-2021	p-value		
Andean Area		2·01 (1·58, 2·52)	1·82 (1·32, 2·45)	-50	-1·52 (-6·19, 3·37)	0·54	2	2014, 2019
Bolivia	BOL	4·35 (2·40, 7·09)	2·64 (1·43, 4·38)	-89	-8·38 (-9·44, -7·32)	<0·0001	2	2008, 2019
Colombia	COL	1·85 (1·61, 2·09)	2·02 (1·61, 2·49)	89	4·69 (2·85, 6·56)	<0·0001	1	2012
Ecuador	ECU	4·11 (3·48, 4·84)	2·56 (1·95, 3·31)	-108	-3·06 (-4·34, -1·75)	0·00014	1	2006
Peru	PER	0·69 (0·43, 0·97)	0·80 (0·46, 1·24)	27	1·17 (-1·26, 3·67)	0·33	0	NA
Venezuela	VEN	1·69 (1·43, 1·99)	2·02 (1·44, 2·75)	30	2·05 (0·36, 3·77)	0·020	1	2011
Central America, Mexico and Latin Caribbean		2·68 (2·22, 3·19)	2·63 (2·02, 3·32)	164	0·75 (0·36, 1·15)	0·00085	1	2006
Costa Rica	CRI	2·16 (1·82, 2·54)	2·73 (2·21, 3·30)	17	6·18 (0·86, 11·78)	0·025	2	2003, 2013
Cuba	CUB	4·07 (3·54, 4·66)	2·84 (2·27, 3·48)	-79	-6·08 (-10·05, -1·95)	0·0043	2	2006, 2017
Dominican Republic	DOM	1·98 (1·25, 2·96)	1·50 (0·92, 2·34)	-17	-4·65 (-7·39, -1·82)	0·0014	2	2010, 2018
El Salvador	SLV	3·26 (2·36, 4·22)	3·07 (2·01, 4·43)	-5	-0·98 (-1·55, -0·40)	0·0020	0	NA
Guatemala	GTM	2·95 (2·53, 3·42)	2·80 (2·23, 3·46)	7	1·30 (0·10, 2·52)	0·036	2	2002, 2011
Haiti	HTI	6·17 (2·48, 10·19)	5·47 (2·32, 9·08)	-13	-1·86 (-3·78, 0·10)	0·063	2	2016, 2019
Honduras	HND	1·45 (0·75, 2·53)	1·05 (0·54, 1·85)	-8	-4·56 (-8·39, -0·58)	0·025	1	2019
Mexico	MEX	2·27 (2·15, 2·39)	2·56 (2·17, 2·98)	284	3·32 (2·82, 3·82)	<0·0001	0	NA
Nicaragua	NIC	2·35 (1·68, 3·05)	1·62 (1·05, 2·36)	-20	-5·12 (-6·57, -3·65)	<0·0001	2	2006, 2009
Panama	PAN	1·13 (0·95, 1·35)	0·98 (0·75, 1·24)	0	-3·90 (-5·85, -1·91)	0·00063	0	NA
Non-Latin Caribbean		3·86 (2·95, 4·97)	3·41 (2·34, 4·76)	-9	-2·18 (-3·95, -0·38)	0·020	2	2006, 2015
Antigua and Barbuda	ATG	0·77 (0·60, 0·95)	0·97 (0·82, 1·14)	0	---	---	---	---
Bahamas	BHS	0·70 (0·55, 0·87)	0·75 (0·53, 1·01)	0	-0·85 (-1·44, -0·25)	0·0076	0	NA
Barbados	BRB	1·04 (0·82, 1·29)	0·86 (0·63, 1·15)	0	-0·40 (-0·88, 0·08)	0·095	0	NA
Belize	BLZ	1·64 (1·34, 1·97)	1·61 (1·30, 2·01)	0	-3·56 (-4·76, -2·35)	<0·0001	2	2007, 2010
Dominica	DMA	0·78 (0·45, 1·17)	0·92 (0·51, 1·49)	0	-0·51 (-0·92, -0·11)	0·015	0	NA
Grenada	GRD	0·25 (0·20, 0·29)	0·54 (0·45, 0·68)	0	---	---	---	---
Guyana	GUY	15·66 (12·00, 19·91)	10·56 (7·43, 14·38)	-17	-5·96 (-9·23, -2·57)	0·00066	1	2016
Jamaica	JAM	0·38 (0·31, 0·47)	0·39 (0·28, 0·53)	1	1·04 (0·18, 1·91)	0·020	1	2004
Saint Kitts and Nevis	KNA	0·68 (0·56, 0·83)	0·62 (0·49, 0·72)	0	-3·53 (-4·68, -2·37)	<0·0001	2	2003, 2011
Saint Lucia	LCA	1·10 (0·88, 1·34)	1·13 (0·86, 1·46)	0	-3·08 (-4·32, -1·82)	0·00013	2	2007, 2010
Saint Vincent and the Grenadines	VCT	0·50 (0·40, 0·65)	0·00 (0·00, 0·00)	0	---	---	---	---
Suriname	SUR	13·40 (9·25, 19·13)	14·16 (8·95, 20·99)	6	1·05 (0·80, 1·30)	<0·0001	0	NA
Trinidad and Tobago	TTO	4·30 (3·54, 5·16)	4·13 (2·93, 5·62)	1	-1·28 (-3·09, 0·57)	0·17	2	2006, 2016
North America		6·10 (5·83, 6·34)	5·75 (5·39, 6·11)	-526	-0·56 (-1·69, 0·57)	0·33	1	2017
Canada	CAN	6·31 (5·69, 6·97)	4·07 (3·55, 4·64)	-379	-4·89 (-9·06, -0·53)	0·028	1	2019
United States of America	USA	6·08 (5·85, 6·28)	5·95 (5·61, 6·28)	-147	-0·22 (-1·47, 1·04)	0·73	1	2017
Southern Cone and Brazil		2·89 (2·64, 3·14)	3·58 (3·21, 3·95)	1,327	3·21 (1·39, 5·07)	0·00052	1	2016
Argentina	ARG	3·37 (2·96, 3·82)	3·36 (2·86, 3·90)	24	0·29 (-0·48, 1·07)	0·43	2	2002, 2006
Brazil	BRA	2·63 (2·46, 2·78)	3·56 (3·28, 3·81)	1,284	4·73 (3·18, 6·31)	<0·0001	2	2002, 2016
Chile	CHL	3·98 (3·49, 4·50)	2·91 (2·48, 3·37)	-79	-3·46 (-4·56, -2·35)	<0·0001	2	2005, 2008
Paraguay	PRY	3·57 (2·32, 4·90)	4·73 (2·94, 6·97)	47	2·26 (1·25, 3·29)	0·00014	0	NA
Uruguay	URY	6·77 (5·81, 7·83)	8·90 (7·51, 10·38)	51	1·61 (0·81, 2·41)	0·00042	0	NA
REGION OF THE AMERICAS		4·00 (3·66, 4·35)	3·95 (3·50, 4·43)	906	0·02 (-1·38, 1·45)	0·97	1	2018

AAPC Average annual percent change; --- Zeros present in the time-series (AAPC not estimated); NA Not applicable

Note: AAPCs represent model-based estimates of temporal change derived from the fitted log-linear joinpoint regression using all annual observations over the specified interval and therefore may differ from changes calculated directly from the observed values at the beginning and end of the interval.

Table S3. Suicide mortality rate by age-group in 2015 and 2021 and average annual percent change from 2015 to 2021 in the Region of the Americas by country and sub-region for both sexes combined

Subregion and Country; Age group	ISO3 country code	Age-standardized suicide mortality rate per 100,000 population		Estimated change in number of deaths, 2015–2021	AAPC		No. of joinpoints	Location of joinpoint(s)
		2015	2021		2015-2021	p-value		
Andean Area								
≤24 years		3·19 (2·58, 3·90)	3·62 (2·68, 4·77)	203·59	0·50 (-4·16, 5·38)	0·84	2	2014, 2017
25-49 years		5·44 (4·49, 6·56)	5·62 (4·17, 7·38)	280·40	-0·20 (-0·75, 0·36)	0·47	0	NA
50-64 years		4·97 (4·09, 6·02)	6·61 (4·59, 9·14)	468·56	4·85 (3·10, 6·64)	<0·0001	1	2014
65-74 years		6·85 (5·59, 8·31)	7·64 (5·48, 10·27)	166·20	3·33 (1·21, 5·50)	0·0039	1	2013
≥75 years		8·27 (6·67, 9·97)	9·42 (7·05, 12·09)	109·62	2·53 (0·56, 4·54)	0·015	1	2013
Bolivia	BOL							
≤24 years		3·29 (1·84, 5·32)	1·11 (0·62, 1·82)	-123·84	-15·87 (-16·91, -14·81)	<0·0001	1	2019
25-49 years		6·37 (3·51, 10·40)	3·56 (2·00, 5·77)	-81·55	-10·11 (-13·96, -6·08)	<0·0001	1	2019
50-64 years		10·65 (6·14, 16·77)	15·51 (8·90, 24·53)	80·22	7·62 (6·74, 8·51)	<0·0001	1	2019
65-74 years		13·63 (7·72, 21·25)	15·36 (8·66, 23·73)	11·22	0·06 (-0·42, 0·54)	0·81	0	NA
≥75 years		13·29 (7·74, 20·13)	11·73 (6·87, 17·57)	-1·63	-0·06 (-0·31, 0·18)	0·61	0	NA
Colombia	COL							
≤24 years		2·82 (2·46, 3·19)	3·48 (2·76, 4·27)	116·31	3·35 (0·78, 5·99)	0·010	2	2013, 2019
25-49 years		4·71 (4·20, 5·25)	4·83 (3·85, 5·93)	130·24	0·27 (-4·37, 5·14)	0·91	2	2014, 2017
50-64 years		4·47 (4·00, 4·99)	6·07 (4·67, 7·73)	185·14	5·82 (3·89, 7·79)	<0·0001	1	2012
65-74 years		7·57 (6·72, 8·55)	8·50 (6·63, 10·70)	85·85	3·89 (0·43, 7·46)	0·029	1	2013
≥75 years		9·78 (8·25, 11·27)	12·98 (10·22, 15·95)	82·59	3·72 (0·07, 7·51)	0·046	1	2013
Ecuador	ECU							
≤24 years		6·65 (5·63, 7·71)	5·65 (4·43, 7·05)	-77·99	0·03 (-1·15, 1·23)	0·95	1	2005
25-49 years		10·40 (8·90, 12·08)	9·96 (7·67, 12·74)	55·43	0·97 (-0·13, 2·08)	0·082	0	NA
50-64 years		7·58 (6·35, 8·93)	7·43 (5·33, 10·21)	22·90	0·84 (-0·46, 2·15)	0·20	0	NA
65-74 years		10·33 (8·61, 12·21)	6·88 (5·03, 9·24)	-11·97	-0·21 (-1·85, 1·46)	0·79	1	2004
≥75 years		15·06 (12·55, 17·74)	8·46 (6·47, 10·79)	-17·02	0·07 (-2·09, 2·28)	0·94	1	2005
Peru	PER							
≤24 years		0·75 (0·48, 1·03)	0·92 (0·54, 1·42)	25·76	1·57 (-0·78, 3·98)	0·18	0	NA
25-49 years		1·20 (0·81, 1·64)	1·65 (1·02, 2·46)	70·59	1·41 (-0·84, 3·72)	0·21	0	NA
50-64 years		0·89 (0·60, 1·21)	2·53 (1·52, 3·79)	76·55	2·33 (-0·24, 4·96)	0·073	0	NA
65-74 years		1·31 (0·83, 1·78)	2·77 (1·65, 4·13)	28·76	3·75 (0·53, 7·06)	0·024	0	NA
≥75 years		1·02 (0·64, 1·37)	2·07 (1·21, 3·02)	13·73	1·70 (-3·11, 6·75)	0·48	0	NA
Venezuela	VEN							
≤24 years		4·13 (3·39, 5·02)	6·82 (4·86, 9·29)	267·06	6·04 (2·56, 9·65)	0·0018	1	2010
25-49 years		7·90 (6·63, 9·40)	10·40 (7·51, 13·86)	107·71	0·19 (-0·74, 1·13)	0·68	0	NA
50-64 years		6·95 (5·95, 8·04)	8·76 (5·92, 12·18)	103·39	1·42 (0·33, 2·53)	0·014	1	2008
65-74 years		7·94 (6·78, 9·21)	9·68 (6·89, 13·08)	52·27	2·52 (1·54, 3·51)	<0·0001	1	2009
≥75 years		11·17 (9·38, 13·03)	13·23 (9·93, 17·08)	31·89	2·95 (1·45, 4·47)	0·00063	1	2013
Central America, Mexico and Latin Caribbean								
≤24 years		4·00 (3·37, 4·78)	3·81 (3·16, 4·53)	-216·27	-0·41 (-2·57, 1·80)	0·69	2	2008, 2015
25-49 years		8·33 (7·19, 9·71)	9·27 (7·49, 11·32)	1187·65	1·71 (1·09, 2·34)	<0·0001	1	2010
50-64 years		7·91 (6·79, 9·24)	7·96 (6·08, 10·19)	358·67	-0·44 (-0·66, -0·21)	0·00055	0	NA

65-74 years		9·49 (8·14, 11·04)	8·44 (6·51, 10·70)	65·10	-1·99 (-2·76, -1·22)	<0·0001	2	2003, 2013
≥75 years		14·96 (12·84, 17·12)	13·75 (11·05, 16·76)	52·04	-0·61 (-0·91, -0·30)	0·00053	0	NA
Costa Rica	CRI							
≤24 years		3·39 (2·95, 3·87)	3·90 (3·27, 4·59)	5·43	0·55 (-0·86, 1·97)	0·43	0	NA
25-49 years		8·08 (7·09, 9·16)	12·20 (10·17, 14·44)	87·22	0·01 (-1·05, 1·09)	0·98	0	NA
50-64 years		10·34 (8·96, 11·84)	9·93 (8·08, 12·00)	7·47	-0·73 (-2·36, 0·93)	0·37	1	2003
65-74 years		7·07 (6·07, 8·19)	4·81 (3·89, 5·85)	-1·98	-2·35 (-3·88, -0·80)	0·0050	0	NA
≥75 years		5·81 (4·85, 6·91)	6·10 (4·93, 7·34)	3·51	-2·63 (-5·01, -0·20)	0·035	0	NA
Cuba	CUB							
≤24 years		2·38 (2·06, 2·71)	2·16 (1·72, 2·64)	-13·05	-3·40 (-6·02, -0·70)	0·017	2	2007, 2011
25-49 years		11·55 (10·36, 12·87)	8·08 (6·56, 9·81)	-179·16	-3·73 (-4·53, -2·93)	<0·0001	2	2007, 2010
50-64 years		20·02 (17·96, 22·23)	20·31 (16·20, 24·92)	86·84	-0·33 (-0·74, 0·08)	0·11	0	NA
65-74 years		28·21 (25·05, 31·49)	30·45 (24·45, 37·16)	40·15	0·13 (-0·61, 0·87)	0·71	1	2003
≥75 years		49·39 (42·70, 56·02)	45·42 (36·98, 54·75)	12·20	0·36 (-0·29, 1·01)	0·26	1	2005
Dominican Republic	DOM							
≤24 years		3·33 (2·06, 4·89)	1·43 (0·80, 2·18)	-95·28	-13·99 (-21·18, -6·14)	0·00072	2	2010, 2017
25-49 years		9·21 (6·09, 13·36)	5·38 (3·35, 8·14)	-120·30	-9·01 (-13·42, -4·37)	0·00020	2	2011, 2017
50-64 years		9·22 (6·24, 13·23)	7·88 (4·98, 11·90)	-0·28	0·09 (-0·34, 0·52)	0·67	0	NA
65-74 years		9·88 (6·72, 13·85)	8·23 (5·17, 12·67)	6·16	-0·20 (-0·62, 0·22)	0·32	0	NA
≥75 years		13·88 (9·51, 19·81)	13·51 (8·65, 20·62)	3·78	-1·11 (-1·57, -0·64)	0·00010	1	2005
El Salvador	SLV							
≤24 years		8·86 (6·26, 11·95)	3·85 (2·48, 5·64)	-165·33	-0·87 (-2·52, 0·82)	0·29	0	NA
25-49 years		17·03 (12·99, 21·71)	11·33 (7·67, 16·04)	-90·89	-1·53 (-3·20, 0·15)	0·072	1	2006
50-64 years		10·68 (8·03, 13·59)	10·75 (7·05, 15·51)	9·86	0·18 (-0·03, 0·40)	0·093	0	NA
65-74 years		11·05 (8·16, 14·26)	8·18 (5·49, 11·42)	-5·23	-4·14 (-6·75, -1·45)	0·0027	1	2019
≥75 years		14·32 (10·36, 18·55)	10·71 (7·23, 14·67)	-4·68	-3·85 (-5·73, -1·94)	0·00078	2	2007, 2015
Guatemala	GTM							
≤24 years		4·04 (3·46, 4·66)	3·41 (2·72, 4·19)	-49·73	-2·82 (-9·36, 4·20)	0·42	2	2012, 2017
25-49 years		7·70 (6·71, 8·80)	7·01 (5·57, 8·64)	39·05	0·37 (-1·27, 2·05)	0·64	2	2005, 2012
50-64 years		6·37 (5·48, 7·30)	6·18 (4·76, 7·85)	10·05	1·07 (-0·82, 3·00)	0·25	2	2004, 2012
65-74 years		6·68 (5·73, 7·73)	5·05 (3·92, 6·41)	-1·30	-0·40 (-2·64, 1·90)	0·71	2	2004, 2011
≥75 years		8·40 (7·06, 9·79)	7·00 (5·60, 8·57)	-1·19	0·09 (-1·35, 1·55)	0·90	2	2004, 2011
Haiti	HTI							
≤24 years		3·35 (1·44, 5·72)	2·59 (1·14, 4·39)	-40·75	-4·15 (-5·86, -2·41)	<0·0001	2	2007, 2019
25-49 years		12·50 (6·23, 19·83)	11·89 (6·11, 18·89)	38·59	-0·04 (-0·52, 0·45)	0·87	1	2006
50-64 years		16·21 (8·51, 25·75)	16·54 (8·96, 26·41)	28·66	0·89 (0·12, 1·66)	0·024	1	2017
65-74 years		20·15 (10·94, 31·24)	18·38 (10·30, 28·26)	4·52	-0·34 (-0·51, -0·17)	0·00044	0	NA
≥75 years		20·86 (12·42, 31·29)	19·14 (11·57, 29·55)	2·30	-0·29 (-0·43, -0·15)	0·00031	0	NA
Honduras	HND							
≤24 years		5·39 (2·97, 9·13)	1·16 (0·62, 2·03)	-216·82	-19·06 (-26·59, -10·74)	0·00038	2	2010, 2013
25-49 years		7·07 (3·59, 12·49)	3·89 (1·97, 6·83)	-71·88	-2·52 (-3·50, -1·53)	<0·0001	0	NA
50-64 years		9·75 (5·94, 14·95)	6·73 (4·18, 10·14)	-11·79	-3·87 (-5·48, -2·24)	0·00018	2	2010, 2013
65-74 years		12·03 (7·57, 17·99)	10·07 (6·59, 14·53)	4·02	-1·92 (-3·41, -0·42)	0·016	2	2010, 2013
≥75 years		13·05 (8·24, 19·21)	11·98 (7·73, 17·11)	1·36	-1·14 (-1·90, -0·37)	0·0068	2	2010, 2013
Mexico	MEX							
≤24 years		3·96 (3·76, 4·18)	4·70 (4·17, 5·22)	366·84	4·23 (3·38, 5·10)	<0·0001	1	2007
25-49 years		7·61 (7·32, 7·91)	10·30 (8·89, 11·76)	1468·11	4·00 (3·19, 4·83)	<0·0001	1	2010
50-64 years		5·49 (5·28, 5·71)	5·87 (4·80, 7·04)	219·95	0·84 (0·43, 1·25)	0·00037	0	NA

65-74 years		5·93 (5·65, 6·21)	5·08 (4·19, 6·02)	14·35	-2·80 (-3·95, -1·64)	<0·0001	1	2011
≥75 years		8·93 (8·26, 9·41)	8·58 (7·27, 9·83)	33·63	0·17 (-0·68, 1·04)	0·68	0	NA
Nicaragua	NIC							
≤24 years		3·45 (2·52, 4·35)	2·61 (1·74, 3·66)	-26·75	-3·07 (-3·74, -2·39)	<0·0001	0	NA
25-49 years		6·84 (5·35, 8·55)	6·88 (4·81, 9·46)	21·38	-2·03 (-2·65, -1·40)	<0·0001	0	NA
50-64 years		4·58 (3·44, 5·96)	3·64 (2·43, 5·26)	-0·55	-4·10 (-5·48, -2·70)	<0·0001	0	NA
65-74 years		3·89 (2·86, 5·14)	2·97 (1·99, 4·23)	-0·28	-3·26 (-5·32, -1·15)	0·0045	0	NA
≥75 years		4·33 (3·17, 5·86)	2·22 (1·41, 3·24)	-2·11	-6·26 (-9·94, -2·44)	0·0033	1	2004
Panama	PAN							
≤24 years		1·33 (1·13, 1·56)	2·15 (1·65, 2·74)	15·97	-3·66 (-5·33, -1·96)	0·00025	0	NA
25-49 years		3·58 (3·08, 4·13)	3·22 (2·42, 4·08)	-1·18	-6·16 (-7·30, -5·00)	<0·0001	1	2004
50-64 years		4·27 (3·64, 4·98)	5·07 (3·64, 6·62)	9·77	-3·62 (-5·01, -2·20)	<0·0001	0	NA
65-74 years		2·69 (2·27, 3·16)	4·41 (3·18, 5·73)	5·19	-5·22 (-7·78, -2·59)	0·00058	0	NA
≥75 years		9·17 (7·61, 10·79)	9·27 (6·93, 11·72)	3·42	1·85 (-2·00, 5·85)	0·33	1	2008
Non-Latin Caribbean								
≤24 years		4·53 (3·50, 5·71)	4·07 (2·88, 5·53)	-21·64	-1·88 (-3·95, 0·23)	0·080	2	2011, 2016
25-49 years		12·66 (10·02, 15·66)	10·66 (7·62, 14·39)	-34·10	-2·20 (-4·75, 0·41)	0·10	2	2006, 2019
50-64 years		13·12 (10·31, 16·51)	11·91 (8·21, 16·64)	4·03	0·53 (-0·12, 1·18)	0·11	1	2005
65-74 years		14·26 (11·21, 18·11)	13·03 (9·14, 17·94)	6·93	0·80 (0·09, 1·51)	0·031	2	2004, 2009
≥75 years		17·12 (13·50, 21·52)	15·45 (11·21, 20·62)	2·22	-0·19 (-0·71, 0·32)	0·44	1	2004
Antigua and Barbuda	ATG							
≤24 years		0·00 (0·00, 0·00)	1·85 (1·43, 2·37)	0·57	---	---	---	---
25-49 years		0·00 (0·00, 0·00)	0·44 (0·35, 0·56)	0·15	---	---	---	---
50-64 years		2·81 (2·21, 3·48)	0·00 (0·00, 0·00)	-0·42	---	---	---	---
65-74 years		0·00 (0·00, 0·00)	0·00 (0·00, 0·00)	0·00	---	---	---	---
≥75 years		0·00 (0·00, 0·00)	18·12 (14·85, 21·09)	0·61	---	---	---	---
Bahamas	BHS							
≤24 years		1·95 (1·46, 2·54)	1·23 (0·84, 1·74)	-1·20	-9·32 (-12·87, -5·62)	<0·0001	2	2005, 2016
25-49 years		4·54 (3·65, 5·58)	4·53 (3·36, 5·99)	0·24	-0·65 (-1·16, -0·14)	0·016	0	NA
50-64 years		3·32 (2·64, 4·07)	3·63 (2·64, 4·80)	0·64	-0·75 (-1·10, -0·39)	0·00028	0	NA
65-74 years		4·22 (3·48, 5·14)	3·92 (2·95, 5·10)	-0·05	-0·96 (-1·41, -0·51)	0·00044	2	2007, 2010
≥75 years		5·33 (4·41, 6·38)	4·88 (3·79, 6·18)	0·13	-1·04 (-1·92, -0·14)	0·026	2	2007, 2010
Barbados	BRB							
≤24 years		1·21 (0·95, 1·53)	1·04 (0·71, 1·46)	-0·19	-2·20 (-3·13, -1·26)	<0·0001	0	NA
25-49 years		4·71 (3·79, 5·83)	3·48 (2·53, 4·70)	-1·28	-1·70 (-2·51, -0·89)	0·00030	0	NA
50-64 years		4·49 (3·56, 5·54)	3·60 (2·51, 4·84)	-0·42	-3·05 (-5·57, -0·46)	0·021	2	2008, 2019
65-74 years		6·57 (5·35, 8·12)	6·16 (4·39, 8·12)	0·27	-1·25 (-3·61, 1·17)	0·31	2	2012, 2019
≥75 years		11·85 (9·69, 14·36)	12·52 (9·27, 16·03)	0·25	0·52 (-0·15, 1·19)	0·12	2	2004, 2009
Belize	BLZ							
≤24 years		1·78 (1·45, 2·16)	1·03 (0·81, 1·31)	-1·37	-12·99 (-20·93, -4·26)	0·0043	1	2017
25-49 years		9·92 (8·18, 11·92)	6·68 (5·31, 8·27)	-2·36	-6·02 (-8·91, -3·04)	0·00078	2	2008, 2011
50-64 years		8·88 (7·40, 10·60)	7·03 (5·51, 8·85)	0·02	-4·12 (-5·15, -3·07)	<0·0001	2	2007, 2010
65-74 years		11·56 (9·71, 13·74)	8·48 (6·79, 10·42)	-0·01	-4·94 (-7·91, -1·87)	0·0018	2	2007, 2019
≥75 years		17·52 (14·73, 20·69)	13·95 (11·19, 17·05)	-0·13	-1·83 (-2·20, -1·46)	<0·0001	1	2003
Dominica	DMA							
≤24 years		1·29 (0·82, 1·97)	1·24 (0·78, 1·98)	-0·09	0·00 (-0·83, 0·84)	1·00	0	NA
25-49 years		4·42 (2·93, 6·22)	4·25 (2·64, 6·35)	-0·07	-1·81 (-3·51, -0·08)	0·041	1	2013
50-64 years		6·65 (4·44, 9·33)	5·80 (3·79, 8·67)	0·03	-2·06 (-3·78, -0·30)	0·022	1	2019

65-74 years		9·03 (5·93, 12·64)	7·54 (4·89, 10·59)	0·02	-2·81 (-4·43, -1·15)	0·00094	2	2005, 2019
≥75 years		13·68 (9·23, 18·13)	11·05 (7·56, 15·99)	-0·02	-3·22 (-4·64, -1·79)	<0·0001	1	2019
Grenada	GRD							
≤24 years		0·00 (0·00, 0·00)	0·00 (0·00, 0·00)	0·00	---	---	---	---
25-49 years		0·00 (0·00, 0·00)	1·84 (1·45, 2·30)	0·80	---	---	---	---
50-64 years		0·00 (0·00, 0·00)	1·65 (1·28, 2·07)	0·31	---	---	---	---
65-74 years		6·50 (5·34, 7·84)	5·38 (4·44, 6·59)	0·01	---	---	---	---
≥75 years		6·12 (5·04, 7·02)	0·00 (0·00, 0·00)	-0·34	---	---	---	---
Guyana	GUY							
≤24 years		17·36 (13·52, 21·74)	12·66 (9·12, 17·23)	-17·01	-6·37 (-9·79, -2·82)	0·00052	2	2008, 2016
25-49 years		49·17 (38·46, 61·47)	31·82 (22·96, 42·90)	-33·92	-6·00 (-7·45, -4·53)	<0·0001	2	2010, 2014
50-64 years		50·79 (38·93, 65·08)	45·79 (31·75, 63·71)	1·77	0·67 (-0·57, 1·93)	0·27	0	NA
65-74 years		51·03 (39·38, 65·29)	41·63 (29·53, 57·14)	0·40	0·71 (-0·27, 1·70)	0·15	0	NA
≥75 years		46·05 (36·07, 58·10)	32·15 (23·54, 42·21)	-1·15	2·19 (0·62, 3·77)	0·0083	0	NA
Jamaica	JAM							
≤24 years		0·63 (0·49, 0·81)	0·47 (0·32, 0·65)	-2·64	4·15 (-0·53, 9·05)	0·080	0	NA
25-49 years		1·85 (1·45, 2·32)	1·71 (1·16, 2·42)	0·11	1·71 (-1·14, 4·63)	0·23	0	NA
50-64 years		2·33 (1·84, 2·92)	2·33 (1·59, 3·29)	1·52	0·52 (-0·66, 1·71)	0·37	0	NA
65-74 years		4·03 (3·22, 4·98)	4·72 (3·34, 6·47)	1·69	0·28 (-0·87, 1·45)	0·62	0	NA
≥75 years		8·84 (7·10, 10·74)	10·36 (7·78, 13·57)	1·91	1·66 (1·09, 2·23)	<0·0001	0	NA
Saint Kitts and Nevis	KNA							
≤24 years		0·64 (0·52, 0·87)	0·58 (0·39, 0·91)	-0·02	-1·20 (-1·78, -0·61)	0·00038	0	NA
25-49 years		1·99 (1·33, 2·66)	1·62 (1·06, 2·46)	-0·07	-0·47 (-1·12, 0·19)	0·15	1	2005
50-64 years		5·14 (4·16, 6·24)	3·85 (2·83, 4·98)	-0·08	-4·25 (-5·58, -2·89)	<0·0001	2	2002, 2013
65-74 years		7·83 (6·36, 9·79)	5·92 (4·27, 7·89)	0·02	-5·05 (-6·92, -3·13)	<0·0001	1	2013
≥75 years		10·31 (8·38, 12·89)	9·49 (6·96, 11·39)	-0·01	-2·13 (-2·73, -1·53)	<0·0001	2	2007, 2010
Saint Lucia	LCA							
≤24 years		3·20 (2·56, 3·96)	2·26 (1·72, 2·95)	-0·81	-3·32 (-5·21, -1·39)	0·0025	2	2009, 2012
25-49 years		11·08 (9·10, 13·35)	6·28 (4·86, 7·98)	-2·95	-6·58 (-12·51, -0·24)	0·042	1	2019
50-64 years		8·52 (6·94, 10·18)	6·28 (4·64, 8·26)	-0·25	-4·42 (-6·10, -2·70)	<0·0001	2	2007, 2019
65-74 years		10·38 (8·46, 12·52)	8·28 (6·31, 10·75)	-0·08	-2·98 (-5·82, -0·05)	0·046	2	2014, 2019
≥75 years		15·64 (13·10, 18·97)	14·01 (11·00, 17·39)	-0·01	-2·23 (-6·34, 2·06)	0·30	2	2012, 2019
Saint Vincent and the Grenadines	VCT							
≤24 years		3·77 (2·99, 4·65)	0·00 (0·00, 0·00)	-1·63	---	---	---	---
25-49 years		2·40 (1·93, 2·91)	0·00 (0·00, 0·00)	-0·88	---	---	---	---
50-64 years		0·00 (0·00, 0·00)	2·26 (1·83, 2·85)	0·42	---	---	---	---
65-74 years		0·00 (0·00, 0·00)	0·00 (0·00, 0·00)	0·00	---	---	---	---
≥75 years		6·58 (5·48, 7·68)	0·00 (0·00, 0·00)	-0·30	---	---	---	---
Suriname	SUR							
≤24 years		11·27 (8·10, 14·84)	12·60 (8·60, 17·29)	4·26	3·46 (1·60, 5·35)	0·0012	2	2010, 2014
25-49 years		38·64 (28·92, 49·47)	31·50 (21·33, 43·79)	-11·87	0·84 (0·42, 1·25)	0·00041	0	NA
50-64 years		35·95 (25·86, 48·80)	24·39 (15·46, 36·37)	-5·37	-4·81 (-7·55, -1·99)	0·00093	2	2011, 2019
65-74 years		41·09 (29·14, 57·97)	31·37 (19·35, 47·98)	-0·37	-3·82 (-7·45, -0·04)	0·048	2	2012, 2019
≥75 years		50·71 (36·20, 71·00)	36·28 (22·74, 55·65)	-0·86	-1·22 (-1·66, -0·77)	<0·0001	0	NA
Trinidad and Tobago	TTO							
≤24 years		4·16 (3·41, 4·98)	3·94 (2·85, 5·24)	-1·70	-0·23 (-2·71, 2·31)	0·85	1	2013
25-49 years		14·87 (12·48, 17·58)	17·46 (12·72, 23·22)	17·89	4·39 (2·22, 6·61)	0·00047	1	2012

50-64 years		16·24 (13·74, 19·17)	17·84 (12·50, 24·60)	5·85	1·14 (0·64, 1·64)	0·00015	1	2006
65-74 years		17·91 (14·97, 21·45)	16·71 (12·02, 22·32)	5·02	-0·25 (-0·65, 0·16)	0·22	1	2006
≥75 years		20·96 (17·41, 24·94)	20·12 (14·86, 25·89)	2·15	0·42 (-0·50, 1·35)	0·35	1	2011
North America								
≤24 years		5·60 (5·42, 5·78)	7·22 (6·83, 7·62)	1879·36	3·48 (3·03, 3·93)	<0·0001	1	2008
25-49 years		17·52 (16·99, 18·07)	19·09 (18·06, 20·11)	2885·25	1·18 (1·07, 1·29)	<0·0001	0	NA
50-64 years		20·33 (19·62, 21·03)	17·72 (16·87, 18·60)	-1705·74	-2·41 (-4·32, -0·46)	0·016	2	2012, 2018
65-74 years		15·94 (15·04, 16·59)	15·36 (14·35, 16·19)	884·68	-0·94 (-2·58, 0·73)	0·24	2	2006, 2015
≥75 years		19·80 (17·01, 21·35)	20·16 (17·31, 21·82)	846·19	1·25 (0·91, 1·59)	<0·0001	1	2009
Canada	CAN							
≤24 years		5·49 (5·04, 5·97)	3·86 (3·43, 4·30)	-159·7	-4·49 (-13·30, 5·22)	0·35	2	2016, 2019
25-49 years		16·61 (15·17, 18·09)	11·13 (9·78, 12·64)	-577·43	-4·44 (-8·94, 0·27)	0·065	1	2019
50-64 years		18·96 (17·20, 20·79)	13·79 (12·08, 15·69)	-382·81	-4·14 (-9·44, 1·47)	0·15	1	2019
65-74 years		11·97 (10·70, 13·26)	9·47 (8·15, 10·90)	-4·05	-2·85 (-7·08, 1·58)	0·20	1	2019
≥75 years		15·78 (13·41, 17·89)	10·55 (8·63, 12·32)	-74·39	-4·51 (-8·87, 0·05)	0·052	1	2019
United States of America	USA							
≤24 years		5·61 (5·45, 5·76)	7·56 (7·17, 7·95)	2044·54	3·99 (3·42, 4·56)	<0·0001	1	2009
25-49 years		17·62 (17·19, 18·07)	20·00 (19·00, 20·97)	3463·86	1·41 (1·27, 1·54)	<0·0001	0	NA
50-64 years		20·49 (19·91, 21·06)	18·20 (17·45, 18·95)	-1315·5	-2·23 (-3·97, -0·47)	0·013	2	2011, 2018
65-74 years		16·42 (15·56, 16·99)	16·09 (15·11, 16·84)	890·34	-0·73 (-2·41, 0·99)	0·40	2	2005, 2016
≥75 years		20·31 (17·47, 21·80)	21·44 (18·46, 23·09)	921·86	1·50 (1·21, 1·80)	<0·0001	1	2009
Southern Cone and Brazil								
≤24 years		3·44 (3·11, 3·78)	3·80 (3·39, 4·25)	220·66	1·74 (1·40, 2·09)	<0·0001	0	NA
25-49 years		8·40 (7·83, 9·00)	9·07 (8·29, 9·90)	1156·55	0·92 (0·72, 1·11)	<0·0001	0	NA
50-64 years		9·53 (8·86, 10·24)	10·17 (9·29, 11·11)	750·73	1·90 (1·09, 2·72)	0·00012	1	2011
65-74 years		10·06 (9·21, 10·90)	11·74 (10·63, 12·85)	705·05	2·27 (1·28, 3·27)	0·00014	1	2012
≥75 years		15·84 (13·80, 17·42)	16·29 (13·89, 18·17)	367·73	2·11 (-0·40, 4·69)	0·10	1	2017
Argentina	ARG							
≤24 years		6·12 (5·43, 6·83)	5·25 (4·51, 6·01)	-174·16	-3·43 (-6·33, -0·44)	0·027	1	2014
25-49 years		10·25 (9·06, 11·51)	10·24 (8·77, 11·78)	127·25	0·36 (-0·06, 0·79)	0·092	0	NA
50-64 years		9·80 (8·60, 11·12)	7·37 (6·27, 8·54)	-124·31	-5·04 (-10·34, 0·58)	0·078	2	2010, 2019
65-74 years		10·31 (9·02, 11·73)	8·96 (7·54, 10·52)	-6·72	-2·79 (-7·91, 2·60)	0·30	2	2010, 2019
≥75 years		15·92 (13·76, 18·14)	12·12 (10·08, 14·28)	-45·20	-1·03 (-2·42, 0·38)	0·14	1	2009
Brazil	BRA							
≤24 years		2·57 (2·41, 2·74)	3·34 (3·09, 3·61)	480·89	3·83 (2·27, 5·42)	<0·0001	1	2011
25-49 years		7·52 (7·18, 7·87)	8·53 (8·02, 9·06)	1026·65	1·31 (1·12, 1·51)	<0·0001	0	NA
50-64 years		8·72 (8·33, 9·13)	10·52 (9·83, 11·23)	959·04	3·71 (2·31, 5·12)	<0·0001	1	2014
65-74 years		9·31 (8·75, 9·81)	12·29 (11·40, 13·11)	694·06	4·58 (2·54, 6·66)	0·00017	1	2015
≥75 years		15·32 (13·42, 16·47)	17·52 (15·13, 19·09)	400·00	3·79 (0·37, 7·33)	0·029	2	2008, 2018
Chile	CHL							
≤24 years		4·84 (4·35, 5·33)	3·54 (3·05, 4·04)	-90·57	-5·36 (-6·96, -3·73)	<0·0001	1	2009
25-49 years		12·71 (11·55, 13·94)	9·76 (8·52, 11·06)	-123·74	-3·23 (-4·25, -2·20)	<0·0001	2	2005, 2008
50-64 years		14·12 (12·72, 15·70)	8·94 (7·70, 10·33)	-120·56	-6·49 (-12·46, -0·10)	0·046	1	2019
65-74 years		11·82 (10·54, 13·19)	8·53 (7·31, 9·88)	-8·77	-4·95 (-9·03, -0·68)	0·026	1	2014
≥75 years		16·34 (14·20, 18·55)	12·08 (10·02, 14·11)	-19·39	-1·25 (-2·12, -0·37)	0·0076	0	NA
Paraguay	PRY							

≤24 years		5·70 (3·42, 7·50)	5·14 (3·06, 7·58)	-15·43	4·00 (2·74, 5·28)	<0·0001	0	NA
25-49 years		7·01 (4·91, 9·11)	6·74 (4·33, 9·79)	14·21	3·24 (2·09, 4·40)	<0·0001	0	NA
50-64 years		9·15 (6·64, 12·22)	8·95 (5·73, 13·28)	9·78	3·03 (1·73, 4·34)	<0·0001	0	NA
65-74 years		8·75 (6·29, 11·69)	8·99 (5·95, 12·83)	4·51	3·05 (1·20, 4·93)	0·0025	0	NA
≥75 years		1·66 (1·18, 2·25)	7·66 (5·14, 10·95)	9·27	2·64 (-1·31, 6·74)	0·18	0	NA
Uruguay	URY							
≤24 years		7·44 (6·61, 8·36)	10·11 (8·76, 11·59)	25·23	6·48 (4·16, 8·85)	<0·0001	1	2009
25-49 years		21·65 (19·36, 24·09)	30·74 (26·80, 34·95)	113·53	4·76 (3·06, 6·50)	<0·0001	1	2009
50-64 years		26·11 (22·91, 29·65)	29·66 (25·18, 34·66)	27·34	0·28 (-6·50, 7·56)	0·94	2	2013, 2016
65-74 years		30·34 (26·44, 34·70)	36·66 (30·85, 43·22)	22·53	-0·17 (-1·49, 1·17)	0·79	0	NA
≥75 years		33·15 (28·06, 38·19)	39·71 (32·84, 46·96)	22·06	-0·08 (-0·92, 0·77)	0·85	0	NA
REGION OF THE AMERICAS								
≤24 years		4·21 (3·80, 4·67)	4·84 (4·29, 5·46)	2054·59	2·66 (2·23, 3·09)	<0·0001	1	2008
25-49 years		11·25 (10·51, 12·08)	12·15 (10·96, 13·45)	5489·24	1·19 (0·76, 1·63)	<0·0001	1	2012
50-64 years		13·81 (13·02, 14·67)	12·66 (11·47, 14·01)	-119·10	-1·52 (-2·99, -0·04)	0·045	2	2011, 2018
65-74 years		12·70 (11·70, 13·62)	12·73 (11·43, 14·02)	1831·41	-0·06 (-1·38, 1·28)	0·93	2	2006, 2018
≥75 years		17·24 (14·81, 18·90)	17·46 (14·77, 19·45)	1378·82	0·75 (0·47, 1·03)	<0·0001	1	2009

AAPC Average annual percent change; --- Zeros present in the time-series (AAPC not estimated); NA Not applicable
Note: AAPCs represent model-based estimates of temporal change derived from the fitted log-linear joinpoint regression using all annual observations over the specified interval and therefore may differ from changes calculated directly from the observed values at the beginning and end of the interval.

Table S4. Suicide mortality rate by age-group in 2015 and 2021 and average annual percent change from 2015 to 2021 in the Region of the Americas by country and sub-region for males

Subregion and Country; Age group	ISO3 country code	Age-standardized suicide mortality rate per 100,000 population		Estimated change in number of deaths, 2015–2021	AAPC		No. of joinpoints	Location of joinpoint(s)
		2015	2021		2015-2021	p-value		
Andean Area								
≤24 years		4·30 (3·52, 5·21)	5·45 (4·02, 7·18)	316·20	1·10 (-3·83, 6·27)	0·67	2	2014, 2017
25-49 years		8·77 (7·30, 10·49)	9·42 (7·05, 12·26)	314·34	-0·09 (-0·69, 0·51)	0·75	0	NA
50-64 years		8·28 (6·88, 9·92)	11·41 (7·92, 15·76)	416·78	5·55 (3·49, 7·66)	<0·0001	1	2014
65-74 years		12·68 (10·44, 15·20)	14·43 (10·35, 19·35)	148·41	4·03 (1·71, 6·40)	0·0018	1	2013
≥75 years		16·56 (13·46, 19·79)	19·38 (14·57, 24·80)	94·66	3·21 (1·03, 5·44)	0·0062	1	2013
Bolivia	BOL							
≤24 years		2·94 (1·67, 4·69)	1·03 (0·58, 1·63)	-55·02	-15·44 (-16·48, -14·40)	<0·0001	2	2007, 2019
25-49 years		8·44 (4·65, 13·88)	4·84 (2·74, 7·80)	-52·7	-9·20 (-14·38, -3·71)	0·0013	2	2013, 2018
50-64 years		16·16 (9·52, 25·09)	25·02 (14·60, 39·13)	68·54	8·61 (4·89, 12·46)	<0·0001	1	2018
65-74 years		21·70 (12·46, 33·07)	26·68 (15·22, 40·19)	11·48	5·41 (0·56, 10·49)	0·028	1	2018
≥75 years		22·98 (13·62, 33·97)	23·79 (14·13, 34·90)	1·41	0·45 (0·11, 0·78)	0·012	0	NA
Colombia	COL							
≤24 years		3·66 (3·20, 4·14)	4·71 (3·70, 5·83)	95·94	3·50 (2·32, 4·69)	<0·0001	1	2011
25-49 years		7·54 (6·75, 8·37)	7·74 (6·20, 9·47)	107·60	-0·38 (-6·32, 5·94)	0·90	2	2014, 2017
50-64 years		7·47 (6·73, 8·31)	10·54 (8·06, 13·49)	164·09	7·59 (5·10, 10·14)	<0·0001	1	2013
65-74 years		14·48 (12·90, 16·27)	16·41 (12·75, 20·73)	77·57	3·36 (1·13, 5·65)	0·0059	2	2007, 2011
≥75 years		20·85 (17·67, 23·99)	26·71 (21·10, 32·82)	64·67	3·31 (-0·69, 7·47)	0·10	1	2013
Ecuador	ECU							
≤24 years		8·65 (7·27, 10·04)	8·23 (6·41, 10·32)	-16·90	2·74 (1·68, 3·80)	<0·0001	0	NA
25-49 years		16·54 (14·25, 19·04)	17·24 (13·41, 21·83)	89·87	0·38 (-1·24, 2·03)	0·63	1	2005
50-64 years		11·95 (10·03, 14·00)	12·97 (9·29, 17·90)	31·06	1·02 (-0·48, 2·55)	0·17	0	NA
65-74 years		19·67 (16·40, 23·22)	12·68 (9·23, 17·12)	-12·85	0·06 (-1·62, 1·76)	0·94	1	2003
≥75 years		29·72 (24·76, 34·97)	18·25 (13·93, 23·32)	-10·31	1·75 (0·12, 3·41)	0·037	0	NA
Peru	PER							
≤24 years		0·71 (0·46, 0·96)	0·98 (0·58, 1·51)	20·37	2·47 (-0·27, 5·27)	0·075	0	NA
25-49 years		1·64 (1·14, 2·20)	2·64 (1·66, 3·88)	69·94	1·42 (-0·77, 3·67)	0·19	0	NA
50-64 years		1·34 (0·95, 1·81)	4·17 (2·55, 6·21)	65·25	2·44 (-0·22, 5·16)	0·071	0	NA
65-74 years		2·33 (1·49, 3·13)	4·97 (2·98, 7·36)	24·40	4·12 (0·02, 8·39)	0·049	0	NA
≥75 years		1·92 (1·23, 2·56)	3·53 (2·09, 5·09)	9·21	1·78 (-2·61, 6·37)	0·41	0	NA
Venezuela	VEN							
≤24 years		6·81 (5·57, 8·34)	11·87 (8·44, 16·21)	269·47	6·67 (2·91, 10·56)	0·0014	1	2010
25-49 years		13·92 (11·65, 16·57)	18·50 (13·43, 24·52)	99·62	0·26 (-0·72, 1·26)	0·58	0	NA
50-64 years		12·47 (10·70, 14·40)	15·77 (10·58, 21·98)	87·66	1·52 (0·36, 2·69)	0·013	1	2008
65-74 years		15·23 (13·03, 17·64)	19·14 (13·55, 25·99)	47·91	3·16 (2·11, 4·22)	<0·0001	1	2009
≥75 years		24·62 (20·77, 28·66)	30·31 (22·80, 39·12)	29·72	3·33 (1·75, 4·93)	0·00032	1	2013
Central America, Mexico and Latin Caribbean								
≤24 years		5·69 (4·81, 6·79)	5·21 (4·40, 6·12)	-251·03	-1·18 (-3·44, 1·13)	0·29	2	2009, 2015
25-49 years		14·05 (12·22, 16·32)	15·95 (13·06, 19·24)	1099·71	1·94 (1·17, 2·72)	<0·0001	1	2011
50-64 years		13·74 (11·93, 15·93)	14·05 (10·79, 17·94)	299·92	-0·19 (-0·40, 0·02)	0·080	0	NA

65-74 years		16·73 (14·46, 19·30)	15·24 (11·79, 19·26)	55·31	-0·92 (-1·17, -0·67)	<0·0001	0	NA
≥75 years		26·63 (22·97, 30·42)	25·64 (20·63, 31·26)	55·91	-0·76 (-1·07, -0·45)	<0·0001	0	NA
Costa Rica	CRI							
≤24 years		5·11 (4·48, 5·80)	5·80 (4·88, 6·83)	3·45	0·54 (-0·82, 1·92)	0·42	0	NA
25-49 years		13·26 (11·73, 14·91)	19·70 (16·56, 23·15)	69·46	-0·54 (-1·66, 0·59)	0·33	0	NA
50-64 years		17·39 (15·20, 19·72)	17·79 (14·50, 21·48)	10·06	0·04 (-1·33, 1·43)	0·95	0	NA
65-74 years		13·59 (11·69, 15·71)	9·86 (7·96, 12·00)	-0·98	-2·21 (-4·11, -0·27)	0·028	0	NA
≥75 years		13·35 (11·13, 15·84)	12·62 (10·20, 15·17)	2·18	-2·25 (-4·90, 0·48)	0·10	0	NA
Cuba	CUB							
≤24 years		3·57 (3·12, 4·07)	3·25 (2·58, 3·98)	-9·80	-1·79 (-4·13, 0·59)	0·13	2	2007, 2010
25-49 years		18·42 (16·64, 20·37)	14·10 (11·50, 17·07)	-117·85	-3·38 (-4·03, -2·73)	<0·0001	2	2007, 2010
50-64 years		33·19 (29·92, 36·65)	34·22 (27·33, 41·99)	75·87	0·30 (-0·09, 0·70)	0·13	0	NA
65-74 years		45·78 (40·77, 50·82)	52·79 (42·24, 64·53)	40·86	1·23 (0·10, 2·37)	0·035	1	2005
≥75 years		88·62 (76·82, 100·15)	92·68 (75·51, 111·76)	36·49	0·98 (0·18, 1·78)	0·020	1	2007
Dominican Republic	DOM							
≤24 years		5·28 (3·25, 7·73)	2·18 (1·18, 3·29)	-79·11	-15·13 (-23·31, -6·06)	0·0015	2	2010, 2017
25-49 years		15·93 (10·59, 22·98)	8·89 (5·55, 13·38)	-111·56	-9·76 (-14·50, -4·75)	0·00020	2	2010, 2017
50-64 years		16·28 (11·13, 23·20)	13·70 (8·71, 20·64)	-2·07	0·35 (-0·15, 0·85)	0·16	0	NA
65-74 years		16·62 (11·43, 23·06)	13·56 (8·61, 20·88)	3·83	-0·45 (-0·91, 0·02)	0·059	0	NA
≥75 years		26·70 (18·57, 37·74)	25·65 (16·67, 38·94)	2·03	-0·50 (-0·83, -0·17)	0·0050	0	NA
El Salvador	SLV							
≤24 years		14·38 (10·04, 19·61)	4·87 (3·09, 7·29)	-157·71	-0·89 (-2·94, 1·21)	0·38	0	NA
25-49 years		33·37 (25·53, 42·50)	20·27 (13·81, 28·52)	-93·02	-1·84 (-3·68, 0·03)	0·053	1	2006
50-64 years		20·38 (15·67, 25·62)	21·28 (13·99, 30·64)	8·57	1·39 (0·37, 2·42)	0·010	1	2012
65-74 years		20·91 (15·84, 26·63)	15·75 (10·75, 21·82)	-4·77	-3·79 (-6·61, -0·87)	0·011	1	2019
≥75 years		27·92 (20·85, 35·81)	21·27 (14·68, 29·04)	-4·15	-3·32 (-5·36, -1·24)	0·0044	2	2007, 2015
Guatemala	GTM							
≤24 years		5·04 (4·31, 5·82)	4·20 (3·32, 5·20)	-33·82	-3·57 (-11·15, 4·66)	0·38	2	2012, 2017
25-49 years		12·25 (10·73, 13·94)	10·79 (8·63, 13·24)	22·78	0·05 (-1·75, 1·88)	0·96	2	2005, 2012
50-64 years		11·23 (9·71, 12·79)	10·57 (8·10, 13·50)	5·04	1·32 (-0·93, 3·63)	0·23	2	2006, 2012
65-74 years		11·85 (10·20, 13·64)	8·58 (6·60, 10·98)	-2·84	-0·86 (-3·58, 1·93)	0·52	1	2011
≥75 years		15·92 (13·40, 18·53)	13·21 (10·53, 16·21)	-1·41	-0·27 (-1·74, 1·23)	0·71	2	2004, 2011
Haiti	HTI							
≤24 years		2·48 (1·28, 4·22)	2·01 (1·04, 3·34)	-12·41	-3·63 (-5·24, -1·99)	<0·0001	1	2019
25-49 years		17·84 (9·74, 27·98)	17·23 (9·60, 26·95)	31·57	-1·22 (-3·13, 0·73)	0·22	2	2006, 2019
50-64 years		25·08 (14·07, 39·46)	25·80 (14·70, 40·81)	22·47	0·55 (0·42, 0·69)	<0·0001	0	NA
65-74 years		33·62 (18·96, 51·71)	30·86 (17·90, 47·18)	2·85	-0·09 (-0·29, 0·11)	0·35	0	NA
≥75 years		40·67 (25·09, 60·18)	37·33 (23·31, 56·71)	1·70	-0·30 (-0·47, -0·13)	0·0016	0	NA
Honduras	HND							
≤24 years		9·77 (5·46, 16·36)	1·74 (0·97, 2·92)	-210·78	-22·02 (-30·84, -12·07)	0·00056	2	2010, 2013
25-49 years		12·55 (6·37, 22·14)	6·46 (3·28, 11·31)	-71·37	-2·51 (-3·68, -1·34)	0·00026	0	NA
50-64 years		17·03 (10·60, 25·96)	12·07 (7·65, 18·07)	-9·37	-3·72 (-5·55, -1·86)	0·00082	2	2010, 2013
65-74 years		22·28 (14·12, 33·15)	19·38 (12·76, 27·75)	3·80	-1·57 (-3·23, 0·12)	0·066	2	2010, 2013
≥75 years		28·45 (18·13, 41·69)	26·49 (17·24, 37·47)	1·41	-1·06 (-1·95, -0·16)	0·024	2	2010, 2013
Mexico	MEX							
≤24 years		5·66 (5·37, 5·96)	6·66 (5·94, 7·37)	253·49	3·01 (2·02, 4·00)	<0·0001	2	2008, 2011
25-49 years		13·04 (12·56, 13·56)	18·26 (15·91, 20·64)	1333·83	5·66 (2·98, 8·40)	<0·0001	2	2010, 2018
50-64 years		9·81 (9·44, 10·18)	10·57 (8·63, 12·69)	176·53	0·83 (0·44, 1·22)	0·00025	0	NA

65-74 years		11·09 (10·57, 11·61)	9·58 (7·88, 11·41)	9·24	-2·86 (-4·26, -1·43)	0·00059	1	2011
≥75 years		14·72 (13·72, 15·47)	14·18 (12·01, 16·29)	20·40	-0·73 (-1·50, 0·04)	0·062	0	NA
Nicaragua	NIC							
≤24 years		4·23 (3·11, 5·31)	3·14 (2·12, 4·31)	-17·45	-2·18 (-2·90, -1·45)	<0·0001	0	NA
25-49 years		11·15 (8·90, 13·75)	12·68 (8·92, 17·32)	34·62	-1·74 (-2·46, -1·02)	<0·0001	0	NA
50-64 years		8·19 (6·32, 10·55)	6·44 (4·33, 9·28)	-0·38	-3·83 (-5·26, -2·39)	<0·0001	0	NA
65-74 years		7·36 (5·48, 9·60)	5·09 (3·44, 7·20)	-0·91	-3·19 (-5·72, -0·60)	0·019	0	NA
≥75 years		11·25 (8·21, 15·20)	2·56 (1·75, 3·65)	-3·73	-8·35 (-12·66, -3·83)	0·0014	1	2004
Panama	PAN							
≤24 years		1·63 (1·38, 1·91)	2·86 (2·16, 3·66)	12·15	-3·91 (-5·78, -1·99)	0·00042	0	NA
25-49 years		6·08 (5·26, 6·98)	6·01 (4·53, 7·60)	3·20	-6·42 (-7·99, -4·83)	<0·0001	1	2006
50-64 years		6·82 (5·84, 7·91)	9·98 (7·15, 13·04)	13·34	-3·63 (-5·05, -2·19)	<0·0001	0	NA
65-74 years		4·47 (3·77, 5·20)	7·60 (5·44, 9·91)	4·38	-5·09 (-7·63, -2·48)	0·00068	0	NA
≥75 years		20·52 (17·07, 24·22)	17·22 (12·90, 21·80)	1·05	-1·60 (-4·33, 1·22)	0·25	0	NA
Non-Latin Caribbean								
≤24 years		5·94 (4·58, 7·49)	5·73 (4·06, 7·80)	-8·69	0·97 (0·44, 1·50)	0·0010	0	NA
25-49 years		21·12 (16·89, 25·82)	17·19 (12·51, 22·92)	-35·47	-2·58 (-6·04, 0·99)	0·15	2	2012, 2019
50-64 years		21·75 (17·18, 27·17)	20·08 (13·86, 28·02)	4·81	0·80 (0·10, 1·50)	0·028	1	2006
65-74 years		25·13 (19·81, 31·71)	22·50 (15·69, 31·08)	3·70	1·41 (0·52, 2·30)	0·0041	2	2005, 2009
≥75 years		34·46 (27·19, 43·01)	30·58 (22·08, 40·70)	1·36	-0·62 (-1·11, -0·12)	0·019	1	2004
Antigua and Barbuda	ATG							
≤24 years		0·00 (0·00, 0·00)	3·65 (2·81, 4·67)	0·57	---	---	---	---
25-49 years		0·00 (0·00, 0·00)	0·92 (0·73, 1·16)	0·15	---	---	---	---
50-64 years		0·00 (0·00, 0·00)	0·00 (0·00, 0·00)	0·00	---	---	---	---
65-74 years		0·00 (0·00, 0·00)	0·00 (0·00, 0·00)	0·00	---	---	---	---
≥75 years		0·00 (0·00, 0·00)	0·00 (0·00, 0·00)	0·00	---	---	---	---
Bahamas	BHS							
≤24 years		3·46 (2·59, 4·54)	2·11 (1·43, 3·04)	-1·11	-9·67 (-13·06, -6·15)	<0·0001	2	2005, 2016
25-49 years		8·43 (6·78, 10·34)	8·22 (6·14, 10·79)	0·07	-0·58 (-1·09, -0·06)	0·030	0	NA
50-64 years		6·24 (4·94, 7·62)	6·80 (4·94, 8·99)	0·55	-0·61 (-0·96, -0·27)	0·0013	0	NA
65-74 years		8·35 (6·90, 10·16)	7·69 (5·77, 9·97)	-0·08	-0·69 (-1·16, -0·22)	0·0071	2	2007, 2010
≥75 years		11·61 (9·45, 13·94)	10·92 (8·38, 13·72)	0·11	-0·94 (-1·29, -0·59)	<0·0001	0	NA
Barbados	BRB							
≤24 years		1·53 (1·18, 1·94)	1·31 (0·83, 1·85)	-0·12	-3·32 (-4·51, -2·11)	<0·0001	0	NA
25-49 years		8·93 (7·16, 10·99)	6·43 (4·68, 8·69)	-1·23	-1·76 (-2·63, -0·89)	0·00046	0	NA
50-64 years		8·11 (6·48, 10·05)	6·55 (4·58, 8·83)	-0·33	-2·99 (-5·44, -0·48)	0·020	2	2008, 2019
65-74 years		11·74 (9·54, 14·67)	11·37 (8·07, 15·16)	0·26	-0·76 (-3·82, 2·40)	0·63	2	2013, 2019
≥75 years		25·34 (20·80, 30·58)	26·75 (19·75, 34·71)	0·23	0·83 (-0·14, 1·81)	0·089	2	2003, 2011
Belize	BLZ							
≤24 years		2·76 (2·25, 3·36)	1·96 (1·53, 2·47)	-0·72	-8·36 (-13·63, -2·77)	0·0064	1	2013
25-49 years		18·00 (14·87, 21·57)	10·77 (8·59, 13·29)	-3·02	-10·61 (-18·96, -1·41)	0·025	1	2017
50-64 years		14·81 (12·29, 17·68)	10·77 (8·31, 13·70)	-0·25	-4·98 (-6·15, -3·80)	<0·0001	2	2008, 2011
65-74 years		19·54 (16·57, 23·36)	14·63 (11·67, 18·08)	-0·03	-4·44 (-6·73, -2·10)	0·00024	2	2008, 2019
≥75 years		32·83 (27·55, 38·87)	26·30 (20·97, 32·34)	-0·13	-1·81 (-2·17, -1·45)	<0·0001	1	2003
Dominica	DMA							
≤24 years		2·09 (1·37, 3·17)	2·13 (1·20, 3·24)	-0·06	-0·01 (-0·87, 0·85)	0·98	0	NA
25-49 years		7·96 (5·33, 11·15)	7·15 (4·47, 10·56)	-0·12	-2·45 (-4·57, -0·27)	0·030	1	2014
50-64 years		11·75 (8·01, 16·38)	10·51 (6·75, 15·31)	0·04	-1·58 (-3·11, -0·02)	0·047	2	2006, 2019

65-74 years		16·80 (10·84, 23·31)	13·99 (9·19, 19·98)	0·04	-2·81 (-4·19, -1·41)	<0·0001	2	2006, 2019
≥75 years		29·46 (20·20, 39·56)	26·28 (17·29, 35·96)	0·03	-2·09 (-3·43, -0·73)	0·0028	2	2004, 2019
Grenada	GRD							
≤24 years		0·00 (0·00, 0·00)	0·00 (0·00, 0·00)	0·00	---	---	---	---
25-49 years		0·00 (0·00, 0·00)	3·66 (2·85, 4·57)	0·81	---	---	---	---
50-64 years		0·00 (0·00, 0·00)	3·35 (2·62, 4·19)	0·32	---	---	---	---
65-74 years		13·78 (11·37, 16·20)	0·00 (0·00, 0·00)	-0·4	---	---	---	---
≥75 years		0·00 (0·00, 0·00)	0·00 (0·00, 0·00)	0·00	---	---	---	---
Guyana	GUY							
≤24 years		21·94 (16·95, 27·67)	16·81 (12·02, 23·00)	-9·04	-6·98 (-10·74, -3·06)	0·00059	2	2009, 2016
25-49 years		85·31 (67·52, 105·67)	55·53 (40·73, 74·32)	-28·53	-5·43 (-6·90, -3·93)	<0·0001	2	2008, 2014
50-64 years		76·00 (58·06, 97·75)	79·06 (54·68, 110·66)	5·81	2·80 (1·70, 3·91)	<0·0001	2	2002, 2005
65-74 years		90·78 (69·80, 116·45)	71·78 (50·30, 99·53)	-0·49	0·80 (-0·82, 2·44)	0·32	0	NA
≥75 years		90·13 (70·20, 114·54)	62·71 (45·32, 82·50)	-1·09	2·30 (1·01, 3·61)	0·0013	0	NA
Jamaica	JAM							
≤24 years		1·08 (0·84, 1·37)	0·77 (0·53, 1·08)	-2·49	4·86 (-0·54, 10·56)	0·076	0	NA
25-49 years		3·31 (2·58, 4·15)	3·01 (2·04, 4·25)	-0·01	1·87 (-1·29, 5·12)	0·23	0	NA
50-64 years		4·35 (3·41, 5·46)	4·31 (2·92, 6·10)	1·23	0·96 (-0·34, 2·26)	0·14	0	NA
65-74 years		7·48 (5·98, 9·34)	9·17 (6·44, 12·65)	1·44	0·63 (-0·67, 1·95)	0·33	0	NA
≥75 years		18·29 (14·66, 22·34)	20·88 (15·67, 27·44)	1·63	1·07 (0·47, 1·68)	0·0014	0	NA
Saint Kitts and Nevis	KNA							
≤24 years		0·94 (0·71, 1·29)	0·93 (0·66, 1·59)	-0·01	0·00 (-0·64, 0·64)	0·99	0	NA
25-49 years		3·51 (2·38, 4·87)	2·80 (1·87, 4·43)	-0·07	-2·67 (-6·69, 1·52)	0·21	2	2006, 2019
50-64 years		9·17 (7·19, 10·65)	6·55 (4·91, 8·66)	-0·09	-4·30 (-5·87, -2·71)	<0·0001	2	2002, 2013
65-74 years		14·18 (12·16, 18·24)	10·04 (6·69, 14·06)	0·01	-5·38 (-7·42, -3·30)	<0·0001	1	2012
≥75 years		24·67 (21·38, 27·96)	19·26 (16·05, 24·08)	-0·03	-3·73 (-4·74, -2·71)	<0·0001	2	2006, 2012
Saint Lucia	LCA							
≤24 years		5·84 (4·64, 7·24)	4·03 (3·09, 5·28)	-0·79	-5·05 (-9·36, -0·54)	0·031	2	2009, 2014
25-49 years		20·70 (16·97, 24·81)	11·15 (8·63, 14·09)	-2·95	-6·95 (-12·97, -0·52)	0·035	1	2019
50-64 years		15·39 (12·58, 18·44)	10·99 (8·03, 14·34)	-0·30	-4·92 (-7·07, -2·73)	<0·0001	2	2007, 2019
65-74 years		20·60 (16·72, 24·48)	15·14 (11·46, 19·24)	-0·15	-4·19 (-6·53, -1·78)	0·00072	2	2013, 2019
≥75 years		35·75 (29·29, 43·20)	31·34 (24·07, 38·15)	-0·03	-2·52 (-7·12, 2·30)	0·30	2	2012, 2019
Saint Vincent and the Grenadines	VCT							
≤24 years		6·28 (4·98, 7·77)	0·00 (0·00, 0·00)	-1·4	---	---	---	---
25-49 years		4·66 (3·81, 5·71)	0·00 (0·00, 0·00)	-0·88	---	---	---	---
50-64 years		0·00 (0·00, 0·00)	4·30 (3·48, 5·43)	0·42	---	---	---	---
65-74 years		0·00 (0·00, 0·00)	0·00 (0·00, 0·00)	0·00	---	---	---	---
≥75 years		13·74 (11·37, 16·58)	0·00 (0·00, 0·00)	-0·29	---	---	---	---
Suriname	SUR							
≤24 years		13·22 (9·58, 17·17)	17·96 (12·44, 24·47)	6·91	5·85 (3·96, 7·77)	<0·0001	2	2011, 2014
25-49 years		59·41 (45·46, 73·84)	41·16 (28·85, 55·44)	-17·61	0·31 (-0·33, 0·95)	0·33	0	NA
50-64 years		59·10 (43·20, 77·71)	32·59 (21·03, 47·28)	-7·34	-7·41 (-11·31, -3·34)	0·00046	1	2019
65-74 years		71·58 (51·37, 97·62)	48·65 (29·91, 73·98)	-1·07	-5·29 (-8·80, -1·64)	0·0048	2	2012, 2019
≥75 years		101·05 (73·40, 136·13)	77·59 (48·82, 116·30)	-0·85	-0·50 (-0·97, -0·04)	0·034	0	NA
Trinidad and Tobago	TTO							
≤24 years		5·62 (4·61, 6·74)	5·62 (4·04, 7·48)	-0·43	0·00 (-3·03, 3·11)	1·00	1	2013
25-49 years		24·13 (20·36, 28·37)	29·29 (21·57, 38·58)	17·98	4·75 (2·50, 7·05)	0·00031	1	2012

50-64 years		28·03 (23·78, 33·05)	30·57 (21·31, 42·34)	4·75	1·10 (0·54, 1·66)	0·00066	1	2006
65-74 years		31·60 (26·42, 37·87)	29·82 (21·29, 40·13)	4·16	-0·26 (-0·76, 0·25)	0·29	1	2007
≥75 years		45·28 (37·49, 53·73)	41·77 (30·67, 53·80)	1·78	-0·21 (-1·27, 0·86)	0·68	1	2012
North America								
≤24 years		8·47 (8·21, 8·73)	11·12 (10·52, 11·72)	1580·57	3·33 (2·76, 3·89)	<0·0001	1	2009
25-49 years		26·33 (25·56, 27·13)	30·11 (28·51, 31·63)	3219·11	2·06 (1·44, 2·67)	<0·0001	1	2014
50-64 years		30·26 (29·28, 31·25)	27·18 (25·92, 28·50)	-875·60	-2·02 (-3·96, -0·04)	0·045	2	2011, 2018
65-74 years		26·91 (25·56, 27·92)	25·89 (24·34, 27·21)	715·20	-1·03 (-2·96, 0·93)	0·28	2	2006, 2015
≥75 years		37·51 (32·82, 40·10)	38·55 (33·60, 41·43)	849·89	1·19 (0·86, 1·51)	<0·0001	1	2009
Canada	CAN							
≤24 years		7·36 (6·77, 7·97)	5·32 (4·72, 5·92)	-102·40	-4·46 (-13·60, 5·65)	0·37	2	2016, 2019
25-49 years		24·56 (22·48, 26·63)	17·08 (15·02, 19·36)	-378·42	-1·13 (-1·61, -0·66)	<0·0001	0	NA
50-64 years		28·04 (25·50, 30·67)	20·74 (18·23, 23·52)	-270·25	-3·71 (-9·25, 2·18)	0·21	1	2019
65-74 years		19·23 (17·28, 21·23)	14·92 (12·89, 17·14)	-9·77	-0·29 (-0·82, 0·25)	0·27	0	NA
≥75 years		26·28 (22·79, 29·53)	16·35 (13·64, 18·94)	-58·44	-5·46 (-10·88, 0·29)	0·062	1	2019
United States of America	USA							
≤24 years		8·58 (8·35, 8·80)	11·70 (11·10, 12·30)	1683·06	4·95 (2·33, 7·63)	0·00018	2	2008, 2019
25-49 years		26·53 (25·89, 27·18)	31·57 (30·02, 33·00)	3594·25	2·68 (1·75, 3·61)	<0·0001	1	2015
50-64 years		30·53 (29·73, 31·32)	27·95 (26·83, 29·09)	-604·72	-1·90 (-3·80, 0·03)	0·054	2	2011, 2018
65-74 years		27·85 (26·57, 28·73)	27·25 (25·76, 28·45)	724·82	-0·85 (-2·79, 1·12)	0·37	2	2005, 2015
≥75 years		38·97 (34·12, 41·48)	41·55 (36·30, 44·48)	907·83	1·44 (1·16, 1·72)	<0·0001	1	2009
Southern Cone and Brazil								
≤24 years		5·27 (4·77, 5·78)	5·21 (4·65, 5·82)	-146·12	-1·26 (-3·53, 1·05)	0·28	1	2017
25-49 years		13·23 (12·34, 14·14)	13·84 (12·66, 15·06)	685·09	0·72 (0·48, 0·96)	<0·0001	0	NA
50-64 years		15·22 (14·19, 16·33)	16·17 (14·78, 17·70)	563·53	2·26 (1·42, 3·10)	<0·0001	1	2011
65-74 years		17·07 (15·72, 18·46)	20·02 (18·21, 21·90)	549·64	2·39 (1·15, 3·64)	0·00075	1	2012
≥75 years		31·58 (27·88, 34·63)	31·06 (26·93, 34·54)	263·89	1·04 (-0·51, 2·60)	0·18	1	2014
Argentina	ARG							
≤24 years		9·63 (8·55, 10·74)	7·37 (6·34, 8·44)	-220·05	-4·72 (-7·61, -1·75)	0·0041	1	2014
25-49 years		16·15 (14·30, 18·10)	16·13 (13·84, 18·48)	105·38	0·33 (-0·15, 0·82)	0·16	0	NA
50-64 years		15·64 (13·75, 17·73)	12·10 (10·29, 14·00)	-84·83	-4·54 (-9·53, 0·72)	0·090	2	2011, 2019
65-74 years		18·28 (16·04, 20·78)	16·92 (14·21, 19·89)	11·86	-0·98 (-2·14, 0·20)	0·10	1	2007
≥75 years		40·03 (34·72, 45·49)	28·34 (23·68, 33·43)	-53·41	-1·17 (-2·70, 0·40)	0·13	1	2009
Brazil	BRA							
≤24 years		3·93 (3·69, 4·19)	4·54 (4·20, 4·92)	159·47	2·25 (1·70, 2·81)	<0·0001	0	NA
25-49 years		11·84 (11·31, 12·38)	12·87 (12·11, 13·65)	587·44	1·09 (0·83, 1·34)	<0·0001	0	NA
50-64 years		13·91 (13·30, 14·54)	16·62 (15·55, 17·78)	706·79	3·86 (2·63, 5·10)	<0·0001	1	2013
65-74 years		15·37 (14·55, 16·14)	20·57 (19·21, 21·88)	531·00	4·62 (2·62, 6·66)	0·00013	1	2014
≥75 years		27·55 (24·62, 29·38)	31·66 (27·93, 34·24)	321·30	3·58 (0·05, 7·23)	0·046	2	2008, 2017
Chile	CHL							
≤24 years		7·05 (6·38, 7·72)	4·96 (4·30, 5·65)	-73·82	-6·32 (-8·04, -4·56)	<0·0001	1	2010
25-49 years		20·03 (18·34, 21·80)	15·20 (13·32, 17·13)	-103·68	-6·30 (-12·32, 0·14)	0·055	1	2019
50-64 years		22·63 (20·53, 24·99)	15·20 (13·12, 17·51)	-79·19	-6·21 (-12·77, 0·84)	0·083	1	2019
65-74 years		21·41 (19·19, 23·80)	15·83 (13·59, 18·29)	-4·36	-4·94 (-8·25, -1·51)	0·0078	1	2013
≥75 years		35·05 (30·69, 39·58)	23·58 (19·78, 27·44)	-25·71	-1·65 (-2·55, -0·73)	0·0013	0	NA
Paraguay	PRY							

≤24 years		7·88 (4·70, 10·30)	5·46 (3·19, 8·25)	-37·12	-3·79 (-20·00, 15·72)	0·68	1	2019
25-49 years		10·30 (7·27, 13·08)	8·77 (5·69, 12·40)	-3·03	3·48 (2·02, 4·95)	<0·0001	0	NA
50-64 years		14·69 (10·84, 19·37)	13·72 (8·83, 20·23)	4·99	3·31 (1·86, 4·79)	0·00011	0	NA
65-74 years		12·95 (9·59, 16·94)	11·93 (8·04, 16·94)	1·23	3·05 (-0·06, 6·25)	0·054	0	NA
≥75 years		2·04 (1·51, 2·68)	16·20 (10·90, 23·14)	8·82	2·10 (-2·55, 6·96)	0·36	0	NA
Uruguay	URY							
≤24 years		11·79 (10·50, 13·18)	16·26 (14·09, 18·66)	21·93	4·48 (3·45, 5·52)	<0·0001	0	NA
25-49 years		34·23 (30·95, 37·74)	49·80 (43·70, 56·32)	97·96	4·61 (3·24, 6·01)	<0·0001	1	2006
50-64 years		41·99 (37·12, 47·33)	45·42 (38·73, 52·88)	16·16	0·33 (-5·87, 6·94)	0·92	2	2013, 2016
65-74 years		58·16 (50·98, 66·16)	62·61 (52·93, 73·72)	10·5	5·16 (0·74, 9·77)	0·024	1	2013
≥75 years		74·01 (63·62, 84·68)	84·92 (71·06, 100·25)	12·71	-0·85 (-1·67, -0·03)	0·044	0	NA
REGION OF THE AMERICAS								
≤24 years		6·21 (5·64, 6·86)	7·11 (6·33, 7·97)	1503·05	1·51 (0·17, 2·87)	0·027	2	2009, 2017
25-49 years		17·60 (16·47, 18·88)	19·51 (17·65, 21·52)	5280·95	1·46 (0·95, 1·97)	<0·0001	1	2013
50-64 years		21·39 (20·20, 22·68)	20·20 (18·28, 22·39)	409·50	-1·18 (-2·68, 0·34)	0·13	1	2018
65-74 years		21·75 (20·16, 23·24)	21·89 (19·74, 24·10)	1472·06	0·03 (-1·51, 1·59)	0·97	2	2006, 2018
≥75 years		32·79 (28·59, 35·73)	33·45 (28·68, 37·08)	1264·60	0·70 (0·46, 0·94)	<0·0001	1	2009

AAPC Average annual percent change; --- Zeros present in the time-series (AAPC not estimated); NA Not applicable

Note: AAPCs represent model-based estimates of temporal change derived from the fitted log-linear joinpoint regression using all annual observations over the specified interval and therefore may differ from changes calculated directly from the observed values at the beginning and end of the interval.

Table S5. Suicide mortality rate by age-group in 2015 and 2021 and average annual percent change from 2015 to 2021 in the Region of the Americas by country and sub-region for females

Subregion and Country; Age group	ISO3 country code	Age-standardized suicide mortality rate per 100,000 population		Estimated change in number of deaths, 2015–2021	AAPC		No. of joinpoints	Location of joinpoint(s)
		2015	2021		2015-2021	p-value		
Andean Area								
≤24 years		2·03 (1·60, 2·54)	1·73 (1·29, 2·26)	-105·60	-2·41 (-8·10, 3·63)	0·43	2	2014, 2019
25-49 years		2·14 (1·70, 2·67)	1·86 (1·32, 2·55)	-33·87	-0·71 (-1·21, -0·20)	0·0088	0	NA
50-64 years		1·89 (1·49, 2·37)	2·15 (1·49, 2·98)	54·24	-0·04 (-0·65, 0·58)	0·89	0	NA
65-74 years		1·75 (1·34, 2·28)	1·88 (1·34, 2·56)	20·46	-0·16 (-1·04, 0·72)	0·70	0	NA
≥75 years		2·10 (1·61, 2·67)	2·38 (1·72, 3·11)	16·70	-0·62 (-1·67, 0·45)	0·24	0	NA
Bolivia	BOL							
≤24 years		3·64 (2·02, 5·97)	1·19 (0·65, 2·02)	-68·59	-16·25 (-17·65, -14·83)	<0·0001	2	2007, 2019
25-49 years		4·27 (2·34, 6·85)	2·27 (1·24, 3·71)	-29·09	-11·03 (-14·79, -7·11)	<0·0001	1	2018
50-64 years		5·27 (2·86, 8·68)	6·34 (3·39, 10·44)	11·90	2·51 (1·46, 3·58)	<0·0001	1	2019
65-74 years		6·69 (3·65, 11·11)	6·04 (3·25, 10·17)	-0·05	-1·66 (-2·00, -1·31)	<0·0001	1	2011
≥75 years		6·65 (3·71, 10·64)	3·89 (2·14, 6·31)	-2·95	-9·14 (-9·45, -8·83)	<0·0001	2	2011, 2019
Colombia	COL							
≤24 years		1·94 (1·69, 2·20)	2·21 (1·78, 2·66)	21·71	4·49 (-3·42, 13·06)	0·27	2	2013, 2019
25-49 years		1·95 (1·72, 2·21)	1·96 (1·54, 2·45)	22·46	3·30 (1·31, 5·33)	0·0026	1	2011
50-64 years		1·72 (1·50, 1·96)	1·98 (1·56, 2·46)	22·06	-0·10 (-1·28, 1·09)	0·86	0	NA
65-74 years		1·68 (1·45, 1·95)	1·76 (1·42, 2·16)	8·29	0·27 (-2·71, 3·34)	0·86	0	NA
≥75 years		1·96 (1·60, 2·30)	3·29 (2·53, 4·05)	16·10	-1·82 (-4·68, 1·12)	0·21	0	NA
Ecuador	ECU							
≤24 years		4·57 (3·93, 5·29)	2·97 (2·37, 3·65)	-60·96	-2·85 (-4·13, -1·55)	0·00026	1	2005
25-49 years		4·26 (3·56, 5·11)	2·66 (1·92, 3·61)	-34·16	-4·31 (-7·27, -1·26)	0·0087	1	2009
50-64 years		3·44 (2·86, 4·11)	2·15 (1·56, 2·88)	-8·51	0·20 (-2·06, 2·51)	0·86	0	NA
65-74 years		1·95 (1·62, 2·32)	1·76 (1·33, 2·28)	0·94	-1·59 (-5·42, 2·39)	0·41	1	2007
≥75 years		3·72 (3·09, 4·40)	1·22 (0·95, 1·54)	-5·05	2·27 (-1·61, 6·31)	0·24	0	NA
Peru	PER							
≤24 years		0·79 (0·49, 1·09)	0·85 (0·49, 1·32)	4·83	0·87 (-1·61, 3·41)	0·48	0	NA
25-49 years		0·77 (0·48, 1·09)	0·70 (0·40, 1·09)	1·52	1·45 (-1·34, 4·32)	0·29	0	NA
50-64 years		0·46 (0·27, 0·65)	0·93 (0·53, 1·44)	11·62	2·54 (-1·52, 6·76)	0·21	0	NA
65-74 years		0·37 (0·21, 0·52)	0·81 (0·47, 1·24)	4·55	---	---	---	---
≥75 years		0·25 (0·15, 0·35)	0·90 (0·50, 1·36)	4·49	---	---	---	---
Venezuela	VEN							
≤24 years		1·30 (1·09, 1·53)	1·44 (1·05, 1·91)	-2·40	-1·06 (-5·35, 3·42)	0·64	2	2013, 2016
25-49 years		1·99 (1·69, 2·34)	2·44 (1·70, 3·39)	5·72	1·16 (-0·05, 2·38)	0·058	1	2007
50-64 years		1·88 (1·59, 2·19)	2·47 (1·73, 3·38)	17·14	4·76 (-0·56, 10·38)	0·081	2	2008, 2019
65-74 years		1·81 (1·52, 2·11)	2·20 (1·62, 2·85)	6·65	3·24 (1·12, 5·40)	0·0048	1	2015
≥75 years		2·57 (2·10, 3·04)	2·91 (2·17, 3·72)	3·94	1·26 (0·41, 2·13)	0·0062	1	2008
Central America, Mexico and Latin Caribbean								
≤24 years		2·28 (1·89, 2·73)	2·38 (1·91, 2·91)	33·29	1·42 (0·98, 1·86)	<0·0001	0	NA
25-49 years		2·91 (2·42, 3·45)	2·89 (2·15, 3·75)	76·65	0·91 (0·34, 1·49)	0·0037	1	2006
50-64 years		2·62 (2·13, 3·18)	2·53 (1·88, 3·29)	51·32	-1·06 (-1·52, -0·59)	0·00012	0	NA

65-74 years		3·08 (2·53, 3·71)	2·64 (2·01, 3·38)	8·60	-2·67 (-4·38, -0·93)	0·0055	2	2002, 2011
≥75 years		5·64 (4·78, 6·48)	4·59 (3·68, 5·56)	-5·65	0·53 (-0·43, 1·49)	0·27	0	NA
Costa Rica	CRI							
≤24 years		1·59 (1·36, 1·85)	1·91 (1·58, 2·25)	1·96	0·71 (-1·93, 3·42)	0·58	0	NA
25-49 years		2·82 (2·37, 3·32)	4·53 (3·64, 5·54)	17·6	8·42 (3·15, 13·95)	0·0032	1	2014
50-64 years		3·52 (2·92, 4·21)	2·35 (1·89, 2·86)	-3·10	5·07 (0·30, 10·08)	0·039	2	2004, 2007
65-74 years		0·93 (0·78, 1·11)	0·21 (0·17, 0·25)	-0·87	---	---	---	---
≥75 years		0·00 (0·00, 0·00)	1·01 (0·81, 1·22)	1·26	---	---	---	---
Cuba	CUB							
≤24 years		1·10 (0·94, 1·27)	0·99 (0·80, 1·21)	-3·09	-5·48 (-7·57, -3·35)	<0·0001	0	NA
25-49 years		4·58 (3·98, 5·24)	1·83 (1·44, 2·28)	-61·23	-11·86 (-17·37, -5·99)	0·00013	2	2007, 2017
50-64 years		7·53 (6·63, 8·55)	7·16 (5·69, 8·79)	10·61	-2·04 (-3·06, -1·00)	0·00059	0	NA
65-74 years		12·18 (10·70, 13·85)	10·93 (8·90, 13·25)	-1·18	-1·63 (-2·64, -0·61)	0·0037	1	2002
≥75 years		17·51 (14·96, 20·18)	9·48 (7·69, 11·41)	-24·24	-1·74 (-2·89, -0·58)	0·0053	0	NA
Dominican Republic	DOM							
≤24 years		1·29 (0·81, 1·92)	0·64 (0·39, 1·02)	-16·02	-10·49 (-21·21, 1·68)	0·089	2	2007, 2017
25-49 years		2·35 (1·49, 3·54)	1·80 (1·10, 2·80)	-7·16	-4·81 (-7·96, -1·56)	0·0040	2	2012, 2018
50-64 years		2·30 (1·44, 3·47)	2·23 (1·35, 3·42)	2·02	-0·08 (-0·63, 0·48)	0·77	2	2007, 2010
65-74 years		3·35 (2·16, 4·92)	3·28 (1·98, 5·06)	2·54	0·52 (0·35, 0·68)	<0·0001	0	NA
≥75 years		3·92 (2·48, 5·82)	4·05 (2·41, 6·24)	1·10	-0·14 (-1·26, 0·99)	0·81	2	2005, 2016
El Salvador	SLV							
≤24 years		3·03 (2·27, 3·85)	2·76 (1·82, 3·90)	-7·49	-1·17 (-2·15, -0·17)	0·024	0	NA
25-49 years		3·55 (2·63, 4·56)	3·55 (2·33, 5·18)	2·63	-0·84 (-1·56, -0·11)	0·027	0	NA
50-64 years		3·52 (2·38, 4·69)	3·29 (2·13, 4·79)	1·06	-2·85 (-4·55, -1·12)	0·0035	2	2004, 2013
65-74 years		3·62 (2·36, 4·93)	2·89 (1·81, 4·15)	-0·46	-3·36 (-4·64, -2·07)	<0·0001	2	2011, 2014
≥75 years		4·78 (3·00, 6·42)	3·76 (2·35, 5·28)	-0·59	-3·43 (-5·24, -1·58)	0·0014	2	2009, 2014
Guatemala	GTM							
≤24 years		3·01 (2·59, 3·47)	2·60 (2·11, 3·16)	-15·62	-2·72 (-6·51, 1·22)	0·16	2	2012, 2015
25-49 years		3·33 (2·85, 3·86)	3·31 (2·58, 4·14)	16·02	1·24 (0·10, 2·39)	0·035	2	2009, 2012
50-64 years		2·27 (1·91, 2·67)	2·59 (2·03, 3·24)	5·20	2·17 (0·89, 3·46)	0·0023	1	2012
65-74 years		2·10 (1·76, 2·49)	2·10 (1·69, 2·60)	1·42	1·08 (-0·05, 2·22)	0·059	2	2007, 2011
≥75 years		2·05 (1·73, 2·40)	2·00 (1·61, 2·39)	0·31	1·63 (0·61, 2·65)	0·0035	1	2010
Haiti	HTI							
≤24 years		4·22 (1·60, 7·23)	3·17 (1·23, 5·46)	-28·26	-4·51 (-6·48, -2·51)	<0·0001	2	2007, 2019
25-49 years		7·19 (2·74, 11·72)	6·62 (2·66, 10·92)	7·23	-1·44 (-2·02, -0·86)	<0·0001	1	2009
50-64 years		8·05 (3·39, 13·13)	7·99 (3·66, 13·12)	6·11	-0·07 (-0·63, 0·50)	0·81	1	2017
65-74 years		9·10 (4·36, 14·44)	8·45 (4·24, 13·19)	1·65	-1·08 (-1·16, -1·01)	<0·0001	0	NA
≥75 years		7·55 (3·91, 11·92)	7·06 (3·77, 11·48)	0·64	-0·97 (-1·04, -0·90)	<0·0001	1	2004
Honduras	HND							
≤24 years		0·79 (0·37, 1·56)	0·55 (0·25, 1·09)	-5·58	-2·09 (-2·87, -1·30)	<0·0001	0	NA
25-49 years		1·50 (0·76, 2·69)	1·24 (0·61, 2·23)	-0·30	-2·99 (-3·37, -2·60)	<0·0001	0	NA
50-64 years		2·69 (1·43, 4·26)	1·73 (0·93, 2·74)	-2·16	-5·57 (-11·07, 0·28)	0·062	1	2018
65-74 years		2·38 (1·41, 3·70)	1·61 (0·98, 2·52)	-0·12	-5·81 (-9·13, -2·37)	0·0011	2	2015, 2019
≥75 years		2·29 (1·32, 3·54)	1·71 (0·99, 2·71)	-0·13	-4·91 (-6·81, -2·97)	<0·0001	2	2014, 2019
Mexico	MEX							
≤24 years		2·25 (2·12, 2·38)	2·71 (2·38, 3·04)	111·98	3·99 (3·29, 4·68)	<0·0001	0	NA
25-49 years		2·56 (2·44, 2·67)	2·89 (2·36, 3·47)	120·37	3·55 (2·96, 4·15)	<0·0001	0	NA
50-64 years		1·60 (1·53, 1·68)	1·69 (1·39, 2·03)	34·43	1·39 (0·52, 2·26)	0·0032	0	NA

65-74 years		1·36 (1·28, 1·42)	1·22 (1·04, 1·41)	4·25	0·72 (-0·69, 2·15)	0·30	0	NA
≥75 years		4·27 (3·87, 4·54)	4·18 (3·56, 4·74)	11·89	-0·50 (-2·87, 1·93)	0·66	2	2005, 2008
Nicaragua	NIC							
≤24 years		2·66 (1·91, 3·36)	2·07 (1·34, 2·99)	-9·36	-4·39 (-5·38, -3·39)	<0·0001	0	NA
25-49 years		2·74 (1·97, 3·60)	1·33 (0·87, 1·93)	-13·09	-3·29 (-4·75, -1·80)	0·00019	0	NA
50-64 years		1·42 (0·93, 1·94)	1·15 (0·74, 1·69)	-0·06	-4·81 (-8·54, -0·92)	0·018	0	NA
65-74 years		1·05 (0·69, 1·44)	1·30 (0·83, 1·88)	0·62	---	---	---	---
≥75 years		0·00 (0·00, 0·00)	1·99 (1·18, 2·97)	1·42	---	---	---	---
Panama	PAN							
≤24 years		1·02 (0·86, 1·20)	1·41 (1·11, 1·78)	3·83	-3·77 (-7·28, -0·12)	0·044	0	NA
25-49 years		1·06 (0·88, 1·26)	0·40 (0·29, 0·53)	-4·37	-6·10 (-8·46, -3·69)	<0·0001	0	NA
50-64 years		1·81 (1·51, 2·16)	0·36 (0·27, 0·45)	-3·51	---	---	---	---
65-74 years		1·08 (0·91, 1·31)	1·51 (1·13, 1·94)	0·79	---	---	---	---
≥75 years		0·00 (0·00, 0·00)	3·31 (2·46, 4·16)	2·80	---	---	---	---
Non-Latin Caribbean								
≤24 years		3·09 (2·38, 3·89)	2·35 (1·66, 3·19)	-13·21	0·46 (-0·22, 1·14)	0·17	0	NA
25-49 years		4·28 (3·21, 5·60)	4·11 (2·72, 5·86)	1·09	-1·64 (-2·90, -0·37)	0·015	2	2009, 2013
50-64 years		5·28 (4·08, 6·83)	4·49 (3·08, 6·32)	-0·97	-0·42 (-1·22, 0·38)	0·28	0	NA
65-74 years		4·91 (3·82, 6·41)	5·24 (3·74, 7·14)	3·22	-0·43 (-1·23, 0·37)	0·28	0	NA
≥75 years		5·05 (3·94, 6·53)	4·73 (3·54, 6·39)	0·61	0·71 (-0·15, 1·58)	0·10	0	NA
Antigua and Barbuda	ATG							
≤24 years		0·00 (0·00, 0·00)	0·00 (0·00, 0·00)	0·00	---	---	---	---
25-49 years		0·00 (0·00, 0·00)	0·00 (0·00, 0·00)	0·00	---	---	---	---
50-64 years		5·15 (4·04, 6·37)	0·00 (0·00, 0·00)	-0·42	---	---	---	---
65-74 years		0·00 (0·00, 0·00)	0·00 (0·00, 0·00)	0·00	---	---	---	---
≥75 years		0·00 (0·00, 0·00)	28·48 (23·89, 33·53)	0·62	---	---	---	---
Bahamas	BHS							
≤24 years		0·43 (0·33, 0·55)	0·36 (0·26, 0·47)	-0·07	-5·21 (-7·59, -2·76)	0·00038	1	2011
25-49 years		0·98 (0·76, 1·22)	1·16 (0·79, 1·61)	0·17	-0·49 (-1·25, 0·28)	0·20	0	NA
50-64 years		0·76 (0·63, 0·92)	0·89 (0·64, 1·15)	0·1	-1·38 (-2·12, -0·64)	0·00092	0	NA
65-74 years		0·99 (0·76, 1·14)	0·94 (0·73, 1·23)	0·00	-2·26 (-3·38, -1·13)	0·00080	2	2006, 2011
≥75 years		1·26 (1·14, 1·60)	1·05 (0·88, 1·40)	0·01	-2·93 (-6·30, 0·57)	0·10	2	2006, 2017
Barbados	BRB							
≤24 years		0·87 (0·71, 1·11)	0·79 (0·58, 1·05)	-0·05	-0·07 (-0·79, 0·66)	0·84	1	2002
25-49 years		0·84 (0·64, 1·04)	0·69 (0·46, 0·96)	-0·09	-1·21 (-1·74, -0·69)	0·00012	0	NA
50-64 years		1·34 (1·04, 1·64)	1·03 (0·74, 1·36)	-0·08	-3·84 (-7·94, 0·45)	0·079	2	2007, 2019
65-74 years		2·38 (1·87, 2·81)	1·80 (1·40, 2·34)	-0·01	-3·89 (-7·98, 0·39)	0·075	2	2007, 2019
≥75 years		3·09 (2·54, 3·65)	2·68 (2·17, 3·30)	-0·02	-2·23 (-5·28, 0·91)	0·16	2	2007, 2018
Belize	BLZ							
≤24 years		0·76 (0·62, 0·92)	0·09 (0·08, 0·11)	-0·62	-30·05 (-34·96, -24·76)	<0·0001	2	2011, 2019
25-49 years		2·01 (1·63, 2·45)	2·58 (2·04, 3·26)	0·64	-1·75 (-3·76, 0·31)	0·090	2	2007, 2010
50-64 years		2·83 (2·29, 3·31)	3·42 (2·73, 4·16)	0·27	3·02 (-2·84, 9·23)	0·32	2	2005, 2018
65-74 years		2·91 (2·46, 3·36)	2·22 (1·90, 2·86)	0·01	-3·53 (-4·02, -3·04)	<0·0001	0	NA
≥75 years		2·94 (2·21, 3·68)	2·33 (2·33, 3·00)	-0·01	-3·30 (-3·78, -2·83)	<0·0001	0	NA
Dominica	DMA							
≤24 years		0·43 (0·29, 0·71)	0·46 (0·18, 0·83)	-0·01	0·63 (-0·12, 1·38)	0·10	0	NA
25-49 years		0·81 (0·49, 1·21)	1·34 (0·75, 2·09)	0·06	0·43 (-0·41, 1·28)	0·30	0	NA
50-64 years		1·15 (0·58, 1·73)	0·96 (0·64, 1·76)	0·00	-2·73 (-3·01, -2·45)	<0·0001	0	NA

65-74 years		1·97 (0·98, 2·95)	1·66 (0·83, 2·07)	0·00	-2·86 (-3·12, -2·60)	<0·0001	2	2004, 2015
≥75 years		2·30 (1·72, 2·87)	2·00 (1·50, 3·00)	0·00	-2·17 (-4·39, 0·09)	0·058	2	2010, 2014
Grenada	GRD							
≤24 years		0·00 (0·00, 0·00)	0·00 (0·00, 0·00)	0·00	---	---	---	---
25-49 years		0·00 (0·00, 0·00)	0·00 (0·00, 0·00)	0·00	---	---	---	---
50-64 years		0·00 (0·00, 0·00)	0·00 (0·00, 0·00)	0·00	---	---	---	---
65-74 years		0·00 (0·00, 0·00)	10·45 (8·58, 13·13)	0·39	---	---	---	---
≥75 years		10·34 (8·51, 11·86)	0·00 (0·00, 0·00)	-0·34	---	---	---	---
Guyana	GUY							
≤24 years		12·68 (10·02, 15·68)	8·39 (6·12, 11·27)	-7·92	-0·10 (-1·26, 1·07)	0·86	0	NA
25-49 years		15·43 (11·33, 20·22)	9·98 (6·61, 13·99)	-5·42	-6·36 (-9·44, -3·17)	0·00069	1	2014
50-64 years		27·00 (20·90, 34·31)	15·57 (10·93, 21·08)	-4·18	0·96 (-1·38, 3·36)	0·40	0	NA
65-74 years		16·60 (12·99, 20·91)	17·07 (12·55, 22·49)	0·82	1·33 (-1·19, 3·92)	0·29	0	NA
≥75 years		15·18 (12·17, 18·56)	11·63 (8·80, 15·30)	-0·10	NA (NA, NA)	NA	NA	NA
Jamaica	JAM							
≤24 years		0·17 (0·14, 0·22)	0·15 (0·11, 0·21)	-0·23	2·21 (0·93, 3·50)	0·0017	0	NA
25-49 years		0·39 (0·31, 0·49)	0·39 (0·26, 0·56)	0·16	-0·01 (-0·80, 0·79)	0·98	0	NA
50-64 years		0·58 (0·47, 0·70)	0·59 (0·42, 0·81)	0·23	1·65 (0·80, 2·50)	0·00070	1	2006
65-74 years		0·93 (0·77, 1·13)	1·13 (0·84, 1·46)	0·27	0·06 (-0·91, 1·03)	0·90	1	2006
≥75 years		1·64 (1·36, 1·94)	1·86 (1·40, 2·35)	0·17	1·25 (0·75, 1·75)	<0·0001	0	NA
Saint Kitts and Nevis	KNA							
≤24 years		0·34 (0·23, 0·46)	0·25 (0·25, 0·25)	-0·01	-3·86 (-12·08, 5·13)	0·39	2	2013, 2016
25-49 years		0·54 (0·32, 0·65)	0·43 (0·43, 0·64)	-0·01	-2·88 (-3·59, -2·15)	<0·0001	1	2003
50-64 years		1·21 (1·21, 1·69)	1·31 (0·88, 1·53)	0·01	-1·13 (-1·73, -0·52)	0·00094	0	NA
65-74 years		1·89 (1·89, 1·89)	1·93 (1·29, 1·93)	0·01	-1·55 (-2·75, -0·34)	0·015	1	2004
≥75 years		3·15 (3·15, 3·15)	3·11 (2·07, 3·11)	0·00	0·29 (0·12, 0·45)	0·0018	1	2002
Saint Lucia	LCA							
≤24 years		0·48 (0·36, 0·57)	0·47 (0·30, 0·57)	-0·02	-2·14 (-3·02, -1·26)	<0·0001	0	NA
25-49 years		1·56 (1·26, 1·92)	1·37 (1·05, 1·82)	-0·04	-4·67 (-5·87, -3·46)	<0·0001	2	2006, 2010
50-64 years		1·67 (1·45, 2·05)	1·87 (1·50, 2·43)	0·08	0·34 (-0·24, 0·93)	0·23	1	2008
65-74 years		1·69 (1·27, 2·11)	2·72 (1·99, 3·26)	0·07	6·41 (1·03, 12·08)	0·019	2	2007, 2019
≥75 years		2·26 (1·61, 2·26)	2·91 (2·62, 3·78)	0·03	2·03 (0·75, 3·32)	0·0036	1	2009
Saint Vincent and the Grenadines	VCT							
≤24 years		1·15 (0·91, 1·48)	0·00 (0·00, 0·00)	-0·24	---	---	---	---
25-49 years		0·00 (0·00, 0·00)	0·00 (0·00, 0·00)	0·00	---	---	---	---
50-64 years		0·00 (0·00, 0·00)	0·00 (0·00, 0·00)	0·00	---	---	---	---
65-74 years		0·00 (0·00, 0·00)	0·00 (0·00, 0·00)	0·00	---	---	---	---
≥75 years		0·00 (0·00, 0·00)	0·00 (0·00, 0·00)	0·00	---	---	---	---
Suriname	SUR							
≤24 years		9·31 (6·61, 12·50)	7·16 (4·69, 10·00)	-2·62	-2·77 (-7·66, 2·38)	0·29	1	2019
25-49 years		16·72 (11·47, 23·74)	21·58 (13·62, 31·81)	5·74	2·80 (2·23, 3·38)	<0·0001	0	NA
50-64 years		14·24 (9·56, 21·68)	16·44 (10·07, 25·78)	1·91	1·44 (0·79, 2·09)	0·00020	1	2009
65-74 years		17·90 (12·25, 27·79)	18·20 (11·31, 28·14)	0·65	0·36 (-0·55, 1·27)	0·42	1	2013
≥75 years		16·97 (11·14, 27·34)	11·99 (7·49, 19·98)	-0·11	-5·26 (-6·72, -3·77)	<0·0001	2	2011, 2019
Trinidad and Tobago	TTO							
≤24 years		2·65 (2·18, 3·16)	2·20 (1·61, 2·91)	-1·26	-4·00 (-8·13, 0·31)	0·069	2	2005, 2017
25-49 years		5·61 (4·59, 6·79)	5·43 (3·72, 7·58)	-0·14	0·54 (-0·27, 1·36)	0·18	2	2007, 2010

50-64 years		5·40 (4·51, 6·43)	6·01 (4·31, 8·15)	1·08	1·18 (0·76, 1·61)	<0·0001	1	2006
65-74 years		6·47 (5·40, 7·71)	6·06 (4·49, 7·91)	1·01	-0·63 (-1·10, -0·16)	0·012	2	2007, 2010
≥75 years		6·33 (5·23, 7·47)	6·07 (4·64, 7·76)	0·36	-0·34 (-0·67, -0·01)	0·045	1	2007
North America								
≤24 years		2·63 (2·53, 2·73)	3·19 (3·01, 3·38)	315·25	4·24 (3·82, 4·66)	<0·0001	0	NA
25-49 years		8·63 (8·34, 8·94)	7·91 (7·45, 8·42)	-248	-1·07 (-2·21, 0·08)	0·069	2	2002, 2017
50-64 years		10·83 (10·38, 11·25)	8·56 (8·10, 8·99)	-808·25	-3·96 (-5·75, -2·13)	0·00031	1	2015
65-74 years		6·27 (5·77, 6·61)	6·01 (5·48, 6·39)	173·42	-1·00 (-2·17, 0·19)	0·10	2	2005, 2017
≥75 years		6·89 (5·51, 7·68)	6·33 (5·08, 7·06)	44·32	-0·09 (-0·62, 0·45)	0·74	2	2003, 2009
Canada	CAN							
≤24 years		3·52 (3·21, 3·85)	2·34 (2·08, 2·60)	-56·60	-4·65 (-12·31, 3·67)	0·26	1	2019
25-49 years		8·65 (7·85, 9·54)	5·08 (4·45, 5·80)	-199·12	-4·51 (-7·43, -1·50)	0·0060	1	2014
50-64 years		9·99 (9·00, 11·04)	6·97 (6·03, 8·00)	-112·28	-4·41 (-7·08, -1·65)	0·0038	1	2014
65-74 years		5·18 (4·55, 5·81)	4·39 (3·75, 5·09)	5·51	-0·32 (-1·28, 0·65)	0·50	0	NA
≥75 years		8·29 (6·72, 9·60)	6·12 (4·79, 7·26)	-15·77	-1·73 (-2·39, -1·06)	<0·0001	0	NA
United States of America	USA							
≤24 years		2·54 (2·46, 2·62)	3·28 (3·10, 3·45)	376·07	4·61 (4·16, 5·07)	<0·0001	0	NA
25-49 years		8·63 (8·40, 8·88)	8·24 (7·80, 8·73)	-50·67	-0·73 (-1·95, 0·51)	0·25	2	2002, 2017
50-64 years		10·93 (10·55, 11·27)	8·75 (8·35, 9·11)	-696·93	-3·86 (-5·69, -1·98)	<0·0001	1	2016
65-74 years		6·40 (5·92, 6·71)	6·20 (5·69, 6·55)	166·55	-1·00 (-2·02, 0·04)	0·060	2	2005, 2016
≥75 years		6·71 (5·35, 7·44)	6·35 (5·11, 7·03)	59·35	0·02 (-0·47, 0·52)	0·93	2	2002, 2009
Southern Cone and Brazil								
≤24 years		1·54 (1·38, 1·70)	2·34 (2·08, 2·62)	370·13	6·50 (3·52, 9·57)	0·00022	1	2015
25-49 years		3·62 (3·36, 3·91)	4·32 (3·93, 4·76)	465·77	1·52 (1·21, 1·83)	<0·0001	0	NA
50-64 years		4·32 (4·00, 4·68)	4·67 (4·26, 5·08)	189·94	0·69 (0·27, 1·12)	0·0028	0	NA
65-74 years		4·23 (3·81, 4·62)	4·98 (4·44, 5·46)	166·72	3·34 (0·41, 6·36)	0·025	1	2016
≥75 years		6·25 (5·21, 6·95)	7·18 (5·84, 8·07)	130·20	0·56 (-0·18, 1·30)	0·13	0	NA
Argentina	ARG							
≤24 years		2·42 (2·13, 2·71)	3·01 (2·58, 3·46)	45·93	1·48 (0·72, 2·25)	0·00062	0	NA
25-49 years		4·22 (3·71, 4·78)	4·19 (3·55, 4·89)	22·72	0·29 (-0·15, 0·74)	0·19	0	NA
50-64 years		4·26 (3·73, 4·85)	2·87 (2·44, 3·34)	-39·01	-3·56 (-4·42, -2·68)	<0·0001	0	NA
65-74 years		3·80 (3·30, 4·35)	2·39 (2·03, 2·79)	-17·33	-3·94 (-5·09, -2·78)	<0·0001	0	NA
≥75 years		2·81 (2·35, 3·25)	3·36 (2·72, 3·93)	12·09	-1·13 (-3·37, 1·17)	0·31	2	2003, 2007
Brazil	BRA							
≤24 years		1·16 (1·10, 1·23)	2·09 (1·93, 2·25)	320·51	9·36 (6·91, 11·86)	<0·0001	1	2014
25-49 years		3·29 (3·14, 3·44)	4·25 (3·98, 4·53)	432·17	4·42 (2·11, 6·79)	0·00015	2	2002, 2018
50-64 years		4·05 (3·85, 4·26)	5·01 (4·66, 5·32)	253·95	2·37 (1·86, 2·87)	<0·0001	0	NA
65-74 years		4·32 (3·97, 4·59)	5·65 (5·13, 6·06)	173·76	2·71 (2·23, 3·19)	<0·0001	0	NA
≥75 years		7·63 (6·37, 8·35)	8·59 (7·02, 9·52)	105·65	1·14 (0·23, 2·07)	0·018	1	2002
Chile	CHL							
≤24 years		2·54 (2·24, 2·85)	2·07 (1·76, 2·38)	-16·72	-4·29 (-6·23, -2·30)	0·00043	2	2003, 2008
25-49 years		5·29 (4·66, 5·97)	4·23 (3·63, 4·88)	-19·78	-3·21 (-4·94, -1·44)	0·0014	1	2009
50-64 years		5·96 (5·23, 6·78)	2·90 (2·45, 3·38)	-41·34	-5·65 (-9·86, -1·25)	0·015	1	2012
65-74 years		3·35 (2·90, 3·83)	1·97 (1·65, 2·31)	-4·87	0·01 (-1·87, 1·91)	0·99	0	NA
≥75 years		4·09 (3·40, 4·78)	4·18 (3·31, 4·95)	3·87	-0·33 (-2·36, 1·75)	0·74	0	NA
Paraguay	PRY							

≤24 years		3·42 (2·08, 4·59)	4·80 (2·92, 6·88)	21·71	1·70 (0·35, 3·08)	0·016	0	NA
25-49 years		3·67 (2·50, 5·08)	4·66 (2·93, 7·12)	16·94	2·83 (1·12, 4·56)	0·0024	0	NA
50-64 years		3·94 (2·69, 5·50)	4·49 (2·84, 6·80)	4·76	3·94 (0·77, 7·20)	0·017	0	NA
65-74 years		4·79 (3·18, 6·73)	6·36 (4·08, 9·17)	3·42	---	---	---	---
≥75 years		1·36 (0·93, 1·90)	1·09 (0·71, 1·56)	-0·01	---	---	---	---
Uruguay	URY							
≤24 years		2·92 (2·55, 3·34)	3·68 (3·18, 4·20)	3·2	2·89 (0·77, 5·05)	0·010	0	NA
25-49 years		9·08 (7·79, 10·45)	11·54 (9·78, 13·43)	15·73	2·11 (1·11, 3·13)	0·00027	0	NA
50-64 years		11·58 (9·92, 13·47)	14·99 (12·56, 17·68)	11·35	0·55 (-0·98, 2·11)	0·46	0	NA
65-74 years		8·69 (7·34, 10·21)	16·06 (13·31, 19·00)	11·92	0·71 (-1·55, 3·02)	0·52	0	NA
≥75 years		13·10 (10·60, 15·39)	17·16 (13·77, 20·38)	8·38	2·25 (0·63, 3·90)	0·0087	0	NA
REGION OF THE AMERICAS								
≤24 years		2·14 (1·90, 2·42)	2·51 (2·19, 2·87)	598·52	2·96 (2·44, 3·48)	<0·0001	1	2009
25-49 years		5·01 (4·66, 5·40)	4·89 (4·37, 5·49)	261·33	0·81 (0·62, 0·99)	<0·0001	0	NA
50-64 years		6·76 (6·34, 7·20)	5·63 (5·13, 6·18)	-519·76	-3·35 (-4·82, -1·87)	0·00020	1	2015
65-74 years		4·86 (4·38, 5·28)	4·85 (4·30, 5·35)	370·31	-0·22 (-1·29, 0·87)	0·69	2	2005, 2017
≥75 years		6·16 (5·00, 6·92)	5·95 (4·78, 6·76)	185·18	0·11 (-0·50, 0·72)	0·71	2	2002, 2009

AAPC Average annual percent change; --- Zeros present in the time-series (AAPC not estimated); NA Not applicable
Note: AAPCs represent model-based estimates of temporal change derived from the fitted log-linear joinpoint regression using all annual observations over the specified interval and therefore may differ from changes calculated directly from the observed values at the beginning and end of the interval.

Table S6. Association between World Health Organization data quality category and progress classification

	Both sexes		Males		Females	
	Odds Ratio (95% CI)	p-value	Odds Ratio (95% CI)	p-value	Odds Ratio (95% CI)	p-value
Data quality category	0·41 (0·12–1·39)	0·16	0·57 (0·17–1·92)	0·36	0·49 (0·15–1·64)	0·25

Table S7. Average annual percent change in the proportion of suicides by method for 20 countries in the Region of the Americas for males

Country; suicide method	Progress toward the one-third reduction target by 2030	Proportion (%)		AAPC (95% CI)		No. of joinpoints	Location of joinpoint(s)
		2015	2021	2015-2021	p-value		
Argentina	No progress						
Firearms		21·23	15·88	-5·91 (-9·73, -1·93)	0·013	0	NA
Hanging		72·87	77·37	1·40 (0·26, 2·55)	0·025	0	NA
Jumping		0·60	1·14	5·53 (-18·41, 36·49)	0·61	0	NA
Poisoning		0·96	1·14	1·02 (-4·12, 6·42)	0·64	0	NA
Other		4·33	4·47	-1·23 (-6·04, 3·83)	0·55	0	NA
Belize	Substantial progress						
Firearms		13·64	9·38	-0·36 (-15·93, 18·08)	0·96	0	NA
Hanging		63·64	75·00	3·19 (-3·95, 10·86)	0·31	0	NA
Jumping		0·00	0·00	---	---	---	---
Poisoning		22·73	12·50	-14·40 (-37·53, 17·28)	0·26	0	NA
Other		0·00	3·13	---	---	---	---
Brazil	Worsening situation						
Firearms		9·32	7·93	-3·12 (-4·44, -1·78)	0·0019	0	NA
Hanging		71·38	75·22	1·04 (0·72, 1·37)	0·00042	0	NA
Jumping		3·35	3·27	-2·06 (-6·61, 2·71)	0·31	0	NA
Poisoning		8·92	7·54	-2·54 (-4·46, -0·59)	0·021	0	NA
Other		7·04	6·03	-3·20 (-5·22, -1·15)	0·011	0	NA
Canada	No progress						
Firearms		18·49	17·16	-2·06 (-3·73, -0·36)	0·026	0	NA
Hanging		50·40	52·22	0·99 (0·04, 1·95)	0·043	0	NA
Jumping		5·33	6·13	1·12 (-1·17, 3·46)	0·27	0	NA
Poisoning		16·44	13·72	-3·27 (-4·42, -2·11)	0·00082	0	NA
Other		9·34	10·78	2·89 (1·76, 4·04)	0·0012	0	NA
Chile	Substantial progress						
Firearms		6·03	3·63	-8·80 (-15·62, -1·42)	0·020	1	2018
Hanging		86·09	89·90	0·88 (-0·16, 1·93)	0·10	1	2018
Jumping		1·58	1·03	-7·94 (-17·95, 3·29)	0·12	0	NA
Poisoning		3·50	2·21	-8·01 (-16·02, 0·77)	0·065	0	NA
Other		2·81	3·24	-0·77 (-11·77, 11·60)	0·87	0	NA
Colombia	No progress						
Firearms		16·21	9·78	-7·85 (-9·32, -6·36)	<0·0001	0	NA
Hanging		58·19	64·91	1·97 (-0·34, 4·34)	0·10	1	2017
Jumping		4·13	4·79	3·36 (-1·12, 8·05)	0·11	0	NA
Poisoning		17·12	13·83	-2·55 (-6·80, 1·88)	0·20	0	NA
Other		4·35	6·69	4·18 (-3·80, 12·83)	0·24	0	NA
Costa Rica	No progress						
Firearms		12·17	11·88	-1·66 (-7·26, 4·28)	0·50	0	NA
Hanging		63·50	70·63	1·20 (-0·15, 2·57)	0·071	0	NA
Jumping		3·42	1·56	-10·22 (-16·13, -3·89)	0·0019	1	2019
Poisoning		18·25	11·88	-3·05 (-11·46, 6·16)	0·42	0	NA
Other		2·66	4·06	1·27 (-9·76, 13·65)	0·79	0	NA
Cuba	Fair progress						
Firearms		1·24	1·22	-4·45 (-11·92, 3·66)	0·21	0	NA
Hanging		85·63	89·23	0·50 (0·11, 0·88)	0·021	0	NA
Jumping		2·15	2·85	2·89 (-6·62, 13·37)	0·48	0	NA
Poisoning		5·12	3·18	-3·15 (-12·14, 6·76)	0·44	0	NA
Other		5·86	3·52	-6·32 (-11·00, -1·39)	0·022	0	NA
Dominican Republic	Substantial progress						
Firearms		16·87	10·53	-9·09 (-26·05, 11·75)	0·29	0	NA
Hanging		58·73	62·11	1·69 (-1·64, 5·13)	0·25	0	NA
Jumping		0·40	1·89	---	---	---	---
Poisoning		15·28	19·16	1·42 (-10·36, 14·76)	0·78	0	NA
Other		8·73	6·32	-3·87 (-22·20, 18·78)	0·65	0	NA
Ecuador	No progress						
Firearms		7·06	3·98	-8·10 (-12·11, -3·91)	0·0046	0	NA
Hanging		72·00	80·94	2·11 (1·05, 3·18)	0·0036	0	NA
Jumping		1·12	1·00	0·73 (-26·77, 38·55)	0·96	1	2017
Poisoning		16·73	12·52	-5·12 (-6·68, -3·53)	0·00045	0	NA
Other		3·10	1·56	-10·90 (-21·65, 1·34)	0·069	0	NA

Guatemala	No progress						
Firearms		12·15	4·32	-12·13 (-19·52, -4·07)	0·013	0	NA
Hanging		42·54	59·59	5·13 (2·62, 7·70)	0·0031	0	NA
Jumping		0·28	0·00	---	---	---	---
Poisoning		40·88	30·26	-5·97 (-9·91, -1·87)	0·014	0	NA
Other		4·14	5·83	9·17 (-7·96, 29·49)	0·24	0	NA
Guyana*	Substantial progress						
Firearms		1·26	1·31	---	---	---	---
Hanging		38·36	41·18	3·27 (-3·56, 10·58)	0·26	0	NA
Jumping		0·00	0·00	---	---	---	---
Poisoning		59·75	56·21	-2·25 (-6·54, 2·23)	0·23	0	NA
Other		0·63	1·31	20·35 (1·32, 42·94)	0·040	0	NA
Mexico	Worsening situation						
Firearms		10·14	6·90	-5·63 (-7·75, -3·46)	0·0012	0	NA
Hanging		80·96	85·85	0·99 (0·46, 1·52)	0·0048	0	NA
Jumping		0·66	0·69	0·49 (-5·26, 6·60)	0·84	0	NA
Poisoning		5·53	3·85	-7·73 (-12·22, -3·00)	0·0090	0	NA
Other		2·72	2·70	1·27 (-4·98, 7·93)	0·63	0	NA
Nicaragua	Fair progress						
Firearms		7·39	3·00	-13·67 (-24·63, -1·12)	0·039	0	NA
Hanging		39·13	50·15	4·20 (1·63, 6·84)	0·0082	0	NA
Jumping		0·00	0·30	---	---	---	---
Poisoning		50·43	44·14	-1·77 (-3·96, 0·46)	0·10	0	NA
Other		3·04	2·40	-12·93 (-29·29, 7·21)	0·15	0	NA
Panama	Substantial progress						
Firearms		4·40	0·88	-18·19 (-38·18, 8·26)	0·12	0	NA
Hanging		67·03	75·22	0·99 (-2·81, 4·93)	0·54	0	NA
Jumping		3·30	1·77	---	---	---	---
Poisoning		25·27	21·24	-1·25 (-20·72, 23·00)	0·89	0	NA
Other		0·00	0·88	---	---	---	---
Paraguay	No progress						
Firearms		18·06	15·15	-5·53 (-12·96, 2·53)	0·13	0	NA
Hanging		73·96	79·55	2·23 (-0·07, 4·59)	0·055	0	NA
Jumping		0·69	1·52	-2·72 (-36·27, 48·48)	0·87	0	NA
Poisoning		3·13	1·77	-8·71 (-18·73, 2·54)	0·10	0	NA
Other		4·17	2·02	-10·08 (-25·35, 8·30)	0·20	0	NA
Peru	No progress						
Firearms		2·37	5·32	18·10 (4·19, 33·87)	0·019	0	NA
Hanging		52·07	58·17	3·76 (-4·37, 12·59)	0·30	0	NA
Jumping		1·18	1·71	---	---	---	---
Poisoning		40·24	27·38	-7·26 (-19·42, 6·73)	0·23	0	NA
Other		4·14	7·41	0·91 (-23·92, 33·85)	0·94	0	NA
Trinidad and Tobago*	Worsening situation						
Firearms		1·80	2·00	---	---	---	---
Hanging		48·65	46·00	-2·45 (-6·42, 1·68)	0·17	0	NA
Jumping		0·00	2·00	---	---	---	---
Poisoning		48·65	49·00	1·57 (-2·04, 5·32)	0·30	0	NA
Other		0·90	1·00	11·56 (-22·57, 60·73)	0·45	0	NA
United States of America	Worsening situation						
Firearms		55·68	59·84	1·08 (0·05, 2·12)	0·040	1	2019
Hanging		26·90	25·15	-0·86 (-3·72, 2·09)	0·56	1	2019
Jumping		2·15	2·28	0·42 (-1·14, 2·00)	0·52	0	NA
Poisoning		10·03	7·41	-4·94 (-5·63, -4·25)	<0·0001	0	NA
Other		5·24	5·32	0·53 (-0·68, 1·75)	0·32	0	NA
Uruguay	Worsening situation						
Firearms		25·29	20·94	-2·76 (-5·83, 0·40)	0·074	0	NA
Hanging		64·71	69·92	1·16 (0·25, 2·08)	0·022	0	NA
Jumping		1·57	1·57	1·94 (-20·89, 31·35)	0·88	1	2017
Poisoning		1·37	2·83	4·71 (-10·18, 22·06)	0·48	0	NA
Other		7·06	4·72	-4·84 (-9·54, 0·10)	0·053	0	NA

AAPC Average annual percent change; --- Zeros present in the time-series (AAPC not estimated); NA Not applicable

*Time-series: 2015-2020; data for 2021 not available

Note: AAPCs represent model-based estimates of temporal change derived from the fitted log-linear joinpoint regression using all annual observations over the specified interval and therefore may differ from changes calculated directly from the observed values at the beginning and end of the interval.

Table S8. Average annual percent change in the proportion of suicides by method for 20 countries in the Region of the Americas for females

Country; suicide method	Progress toward the one-third reduction target by 2030	Proportion (%)		AAPC (95% CI)		No. of joinpoints	Location of joinpoint(s)
		2015	2021	2015-2021	p-value		
Argentina	No progress						
Firearms		10·20	5·80	-13·99 (-22·77, -4·20)	0·016	0	NA
Hanging		73·52	79·09	1·95 (0·51, 3·40)	0·017	0	NA
Jumping		1·64	1·41	-4·85 (-27·19, 24·34)	0·65	0	NA
Poisoning		4·44	5·98	-1·80 (-16·27, 15·16)	0·78	0	NA
Other		10·20	7·73	-3·74 (-10·12, 3·10)	0·21	0	NA
Belize	Substantial progress						
Firearms		0·00	0·00	---	---	---	---
Hanging		75·00	100·00	7·32 (-3·63, 19·50)	0·15	0	NA
Jumping		0·00	0·00	---	---	---	---
Poisoning		25·00	0·00	---	---	---	---
Other		0·00	0·00	---	---	---	---
Brazil	Worsening situation						
Firearms		3·63	2·83	-3·79 (-8·54, 1·20)	0·11	0	NA
Hanging		56·72	58·47	1·04 (0·02, 2·07)	0·046	0	NA
Jumping		7·43	6·82	-1·42 (-7·36, 4·90)	0·58	0	NA
Poisoning		21·87	22·50	-0·84 (-3·32, 1·69)	0·43	0	NA
Other		10·35	9·39	-1·85 (-4·17, 0·52)	0·10	0	NA
Canada	Substantial progress						
Firearms		2·55	2·65	2·51 (-6·66, 12·57)	0·53	0	NA
Hanging		43·75	51·01	2·67 (0·98, 4·40)	0·010	0	NA
Jumping		5·63	6·56	3·17 (-0·25, 6·71)	0·063	0	NA
Poisoning		38·29	31·32	-3·18 (-5·11, -1·22)	0·0090	0	NA
Other		9·77	8·47	-4·08 (-7·24, -0·80)	0·024	0	NA
Chile	Substantial progress						
Firearms		1·85	0·33	-17·88 (-35·90, 5·21)	0·10	0	NA
Hanging		74·60	84·21	1·82 (-0·19, 3·87)	0·076	1	2018
Jumping		4·23	2·30	-8·10 (-19·88, 5·41)	0·17	0	NA
Poisoning		13·76	10·20	-6·46 (-19·67, 8·91)	0·39	1	2018
Other		5·56	2·96	-7·99 (-21·07, 7·26)	0·22	0	NA
Colombia	Worsening situation						
Firearms		4·35	1·37	-16·55 (-31·74, 2·02)	0·068	0	NA
Hanging		48·45	60·65	4·20 (1·22, 7·28)	0·015	0	NA
Jumping		8·07	7·22	-1·67 (-8·75, 5·96)	0·59	0	NA
Poisoning		35·82	26·46	-5·40 (-9·08, -1·58)	0·015	0	NA
Other		3·31	4·30	2·71 (-5·89, 12·10)	0·47	0	NA
Costa Rica	Worsening situation						
Firearms		3·85	0·00	---	---	---	---
Hanging		61·54	64·52	-1·78 (-8·07, 4·95)	0·52	0	NA
Jumping		3·85	3·23	-2·60 (-26·99, 29·94)	0·82	0	NA
Poisoning		28·85	29·03	5·08 (-5·07, 16·33)	0·27	0	NA
Other		1·92	3·23	2·97 (-17·42, 28·39)	0·75	0	NA
Cuba	Substantial progress						
Firearms		0·34	0·62	---	---	---	---
Hanging		39·26	48·14	2·65 (0·05, 5·32)	0·047	0	NA
Jumping		6·38	6·83	0·20 (-13·54, 16·13)	0·97	0	NA
Poisoning		24·83	22·67	-2·03 (-6·95, 3·15)	0·35	0	NA
Other		29·19	21·74	-3·11 (-10·23, 4·56)	0·33	0	NA
Dominican Republic	Substantial progress						
Firearms		5·19	2·25	-16·96 (-27·28, -5·18)	0·016	0	NA
Hanging		61·04	65·17	1·27 (-6·18, 9·30)	0·69	0	NA
Jumping		0·00	1·12	---	---	---	---
Poisoning		19·48	20·22	6·07 (-2·95, 15·93)	0·15	0	NA
Other		14·29	11·24	-10·38 (-29·23, 13·50)	0·29	0	NA
Ecuador	Substantial progress						
Firearms		1·13	1·13	---	---	---	---
Hanging		60·90	68·93	3·85 (0·39, 7·44)	0·035	0	NA
Jumping		0·75	2·26	2·82 (-29·11, 49·13)	0·85	0	NA
Poisoning		35·34	24·29	-8·84 (-15·70, -1·42)	0·029	0	NA

Other		1·88	3·39	5·74 (-9·44, 23·46)	0·40	0	NA
Guatemala	Worsening situation						
Firearms		1·32	1·64	-4·10 (-22·94, 19·34)	0·64	0	NA
Hanging		34·87	45·90	7·10 (-1·16, 16·05)	0·080	0	NA
Jumping		0·00	0·00	---	---	---	---
Poisoning		60·53	45·90	-6·00 (-9·93, -1·91)	0·014	0	NA
Other		3·29	6·56	23·99 (-6·12, 63·76)	0·10	0	NA
Guyana*	Substantial progress						
Firearms		0·00	0·00	---	---	---	---
Hanging		17·74	31·71	13·07 (4·39, 22·46)	0·013	0	NA
Jumping		3·23	0·00	---	---	---	---
Poisoning		77·42	68·29	-2·67 (-4·69, -0·60)	0·023	0	NA
Other		1·61	0·00	---	---	---	---
Mexico	Worsening situation						
Firearms		4·16	2·32	-6·60 (-15·26, 2·94)	0·13	0	NA
Hanging		72·66	83·10	2·20 (1·23, 3·18)	0·0021	0	NA
Jumping		1·44	0·77	-9·31 (-16·28, -1·77)	0·025	0	NA
Poisoning		17·51	11·03	-8·12 (-12·74, -3·26)	0·0083	0	NA
Other		4·24	2·77	-4·62 (-10·02, 1·10)	0·091	0	NA
Nicaragua	Substantial progress						
Firearms		1·18	0·00	---	---	---	---
Hanging		20·00	30·00	8·82 (3·24, 14·69)	0·0091	0	NA
Jumping		1·18	0·00	---	---	---	---
Poisoning		76·47	67·78	-2·96 (-4·95, -0·92)	0·014	0	NA
Other		1·18	2·22	---	---	---	---
Panama	Substantial progress						
Firearms		5·26	0·00	---	---	---	---
Hanging		68·42	82·35	2·36 (-10·24, 16·72)	0·67	0	NA
Jumping		0·00	11·76	---	---	---	---
Poisoning		26·32	0·00	---	---	---	---
Other		0·00	5·88	---	---	---	---
Paraguay	Worsening situation						
Firearms		10·64	3·93	-12·57 (-26·35, 3·78)	0·10	0	NA
Hanging		69·15	82·02	2·03 (-1·77, 5·98)	0·23	0	NA
Jumping		1·06	1·12	-1·69 (-28·70, 35·57)	0·92	1	2018
Poisoning		11·70	8·43	-4·20 (-21·24, 16·52)	0·67	1	2017
Other		7·45	4·49	-4·59 (-23·75, 19·38)	0·61	0	NA
Peru	No progress						
Firearms		0·00	0·75	---	---	---	---
Hanging		40·00	44·91	4·63 (-7·15, 17·91)	0·37	0	NA
Jumping		0·00	1·13	---	---	---	---
Poisoning		55·79	43·02	-6·18 (-17·95, 7·27)	0·28	0	NA
Other		4·21	10·19	9·06 (-21·22, 50·98)	0·52	0	NA
Trinidad and Tobago*	No progress						
Firearms		0·00	0·00	---	---	---	---
Hanging		44·44	47·62	-1·39 (-18·43, 19·21)	0·85	0	NA
Jumping		0·00	0·00	---	---	---	---
Poisoning		55·56	52·38	-0·15 (-10·13, 10·94)	0·97	0	NA
Other		0·00	0·00	---	---	---	---
United States of America	No progress						
Firearms		30·51	34·53	1·55 (0·19, 2·92)	0·032	0	NA
Hanging		26·71	28·43	1·81 (-0·43, 4·10)	0·094	0	NA
Jumping		2·74	3·16	1·44 (-1·99, 4·99)	0·33	0	NA
Poisoning		33·47	27·76	-3·15 (-4·12, -2·16)	0·00047	0	NA
Other		6·57	6·12	-0·83 (-1·74, 0·08)	0·066	0	NA
Uruguay	Worsening situation						
Firearms		17·42	8·46	-7·03 (-19·31, 7·13)	0·24	0	NA
Hanging		58·33	57·69	0·82 (-3·64, 5·50)	0·66	0	NA
Jumping		2·27	5·38	-0·16 (-26·78, 36·13)	0·99	0	NA
Poisoning		6·82	13·85	9·36 (0·09, 19·48)	0·048	0	NA
Other		15·15	14·62	-3·85 (-12·81, 6·04)	0·35	0	NA

AAPC Average annual percent change; --- Zeros present in the time-series (AAPC not estimated); NA Not applicable

*Time-series: 2015-2020; data for 2021 not available

Note: AAPCs represent model-based estimates of temporal change derived from the fitted log-linear joinpoint regression using all annual observations over the specified interval and therefore may differ from changes calculated directly from the observed values at the beginning and end of the interval.

Table S9. Sensitivity analysis: Confidence intervals obtained after propagating uncertainty in the annual suicide mortality estimates through repeated simulation for both sexes combined

Subregion and Country	ISO3 country code	Age-standardized suicide mortality rate per 100,000 population		Estimated change in number of deaths, 2015–2021	AAPC		No. of joinpoints	Location of joinpoint(s)
		2015	2021		2015-2021	p-value		
Andean Area		4·53 (3·71, 5·50)	5·05 (3·69, 6·70)	1233·59	0·65 (-1·58, 2·28)	0·49	2	2014, 2017
Bolivia	BOL	6·28 (3·53, 10·05)	5·08 (2·89, 8·06)	-115·39	-3·09 (-3·92, -2·47)	<0·0001	2	2013, 2019
Colombia	COL	4·08 (3·60, 4·58)	4·71 (3·71, 5·84)	600·48	2·30 (0·68, 3·76)	0·011	2	2013, 2018
Ecuador	ECU	8·51 (7·21, 9·91)	7·42 (5·68, 9·54)	-28·64	-0·54 (-3·36, 0·67)	0·36	1	2006
Peru	PER	0·96 (0·63, 1·31)	1·52 (0·91, 2·29)	215·30	1·85 (-0·44, 4·32)	0·11	0	NA
Venezuela	VEN	6·27 (5·25, 7·45)	8·94 (6·39, 12·04)	561·84	3·00 (1·45, 7·08)	0·00040	1	2009
Central America, Mexico and Latin Caribbean		6·67 (5·71, 7·81)	6·80 (5·45, 8·34)	1445·38	0·83 (0·50, 1·46)	0·00040	1	2007
Costa Rica	CRI	6·16 (5·36, 7·02)	7·62 (6·32, 9·07)	101·41	-0·17 (-1·30, 1·05)	0·76	0	NA
Cuba	CUB	10·37 (9·22, 11·61)	9·24 (7·45, 11·26)	-53·05	-1·59 (-2·40, -1·11)	<0·0001	2	2007, 2010
Dominican Republic	DOM	6·83 (4·50, 9·88)	4·42 (2·73, 6·70)	-205·99	-6·75 (-9·10, -5·09)	<0·0001	2	2011, 2017
El Salvador	SLV	11·92 (8·85, 15·46)	7·85 (5·23, 11·20)	-256·48	-0·34 (-1·48, 0·88)	0·53	0	NA
Guatemala	GTM	5·98 (5·17, 6·86)	5·26 (4·16, 6·52)	-2·39	0·26 (-1·12, 2·23)	0·66	2	2005, 2011
Haiti	HTI	9·89 (5·01, 15·78)	9·27 (4·85, 14·79)	33·44	-1·09 (-1·64, -0·64)	<0·0001	2	2006, 2019
Honduras	HND	7·16 (4·01, 11·85)	3·72 (2·14, 6·00)	-295·04	-7·81 (-11·61, -5·05)	<0·0001	2	2010, 2013
Mexico	MEX	5·69 (5·43, 5·95)	6·89 (5·95, 7·88)	2098·65	2·23 (1·86, 2·62)	<0·0001	0	NA
Nicaragua	NIC	4·86 (3·68, 6·16)	4·20 (2·87, 5·86)	-8·41	-2·89 (-3·44, -2·31)	<0·0001	0	NA
Panama	PAN	2·86 (2·43, 3·33)	3·27 (2·44, 4·18)	33·24	-4·87 (-7·41, -4·03)	0·0040	1	2003
Non-Latin Caribbean		9·31 (7·31, 11·63)	8·12 (5·75, 11·07)	-42·90	-1·58 (-3·32, -0·12)	0·033	2	2006, 2019
Antigua and Barbuda	ATG	0·42 (0·33, 0·52)	1·40 (1·11, 1·72)	0·91	---	---	---	---
Bahamas	BHS	3·16 (2·51, 3·93)	2·91 (2·13, 3·88)	-0·26	-2·73 (-4·98, -0·54)	0·012	1	2017
Barbados	BRB	3·49 (2·79, 4·31)	2·85 (2·04, 3·82)	-1·37	-1·51 (-2·09, -0·89)	<0·0001	0	NA
Belize	BLZ	6·53 (5·41, 7·83)	4·62 (3·66, 5·74)	-3·85	-5·42 (-7·23, -4·12)	<0·0001	2	2008, 2011
Dominica	DMA	3·81 (2·51, 5·37)	3·50 (2·24, 5·22)	-0·12	-1·78 (-3·57, -0·56)	0·0016	1	2014
Grenada	GRD	0·50 (0·41, 0·60)	1·17 (0·93, 1·46)	0·78	18·48 (-7·68, 44·52)	0·17	2	2004, 2018
Guyana	GUY	35·63 (27·71, 44·86)	26·39 (18·84, 35·96)	-49·91	-4·66 (-6·26, -2·81)	<0·0001	2	2008, 2015
Jamaica	JAM	1·69 (1·33, 2·10)	1·67 (1·17, 2·31)	2·52	1·27 (-0·88, 3·51)	0·24	0	NA
Saint Kitts and Nevis	KNA	2·36 (1·82, 3·02)	1·90 (1·33, 2·64)	-0·17	-2·93 (-3·66, -2·36)	<0·0001	2	2006, 2012
Saint Lucia	LCA	7·22 (5·90, 8·73)	4·73 (3·62, 6·07)	-4·10	-4·97 (-8·50, -1·53)	0·0012	1	2019
Saint Vincent and the Grenadines	VCT	2·65 (2·12, 3·24)	0·29 (0·23, 0·36)	-2·38	---	---	---	---
Suriname	SUR	27·27 (19·98, 35·94)	22·41 (14·90, 31·79)	-14·20	0·29 (-0·15, 0·73)	0·19	0	NA
Trinidad and Tobago	TTO	10·69 (8·95, 12·68)	11·65 (8·41, 15·58)	29·25	2·27 (0·79, 4·08)	0·014	2	2005, 2012
North America		12·59 (12·11, 13·03)	13·46 (12·68, 14·19)	4800·52	0·95 (0·40, 1·34)	<0·0001	2	2006, 2018
Canada	CAN	11·59 (10·54, 12·67)	8·08 (7·08, 9·16)	-1197·81	-4·24 (-6·05, -2·86)	<0·0001	1	2019
United States of America	USA	12·71 (12·29, 13·08)	14·07 (13·32, 14·75)	5998·33	1·84 (1·67, 2·29)	<0·0001	1	2006
Southern Cone and Brazil		6·64 (6·11, 7·17)	7·20 (6·51, 7·92)	3209·63	1·17 (0·74, 2·09)	0·0092	1	2010
Argentina	ARG	8·56 (7·55, 9·62)	7·65 (6·53, 8·81)	-222·69	-2·58 (-4·62, -0·71)	0·014	2	2007, 2019
Brazil	BRA	5·81 (5·49, 6·12)	6·91 (6·42, 7·39)	3562·18	1·43 (1·17, 1·70)	<0·0001	0	NA
Chile	CHL	9·40 (8·48, 10·38)	6·82 (5·89, 7·79)	-362·93	-3·54 (-4·56, -2·61)	0·00080	2	2005, 2008
Paraguay	PRY	6·60 (4·43, 8·69)	6·54 (4·11, 9·57)	22·45	3·16 (2·32, 4·07)	<0·0001	0	NA

Uruguay	URY	16·76 (14·83, 18·84)	22·01 (18·97, 25·32)	210·62	4·07 (2·96, 6·21)	<0·0001	1	2009
REGION OF THE AMERICAS		8·75 (8·09, 9·44)	9·16 (8·20, 10·18)	10646·22	1·05 (0·88, 1·37)	<0·0001	1	2006

AAPC Average annual percent change; --- Zeros present in the time-series (AAPC not estimated); NA Not applicable

Note: AAPCs represent model-based estimates of temporal change derived from the fitted log-linear joinpoint regression using all annual observations over the specified interval and therefore may differ from changes calculated directly from the observed values at the beginning and end of the interval.

Table S10. Sensitivity analysis: Confidence intervals obtained after propagating uncertainty in the annual suicide mortality estimates through repeated simulation for males

Subregion and Country	ISO3 country code	Age-standardized suicide mortality rate per 100,000 population		Estimated change in number of deaths, 2015–2021	AAPC		No. of joinpoints	Location of joinpoint(s)
		2015	2021		2015-2021	p-value		
Andean Area		7·20 (5·95, 8·65)	8·47 (6·20, 11·20)	1289·03	1·12 (-1·26, 3·02)	0·25	2	2014, 2017
Bolivia	BOL	8·37 (4·77, 13·24)	7·80 (4·50, 12·13)	-26·46	-0·10 (-0·64, 0·71)	0·78	1	2012
Colombia	COL	6·55 (5·80, 7·34)	7·66 (6·01, 9·52)	510·00	1·79 (0·56, 2·90)	0·0060	2	2013, 2018
Ecuador	ECU	13·12 (11·13, 15·23)	12·40 (9·49, 15·93)	81·28	0·36 (-4·31, 1·54)	0·72	1	2005
Peru	PER	1·25 (0·85, 1·68)	2·28 (1·40, 3·39)	189·69	2·17 (-0·02, 4·52)	0·053	0	NA
Venezuela	VEN	11·15 (9·33, 13·26)	16·22 (11·62, 21·82)	534·52	3·19 (1·56, 7·70)	0·0020	1	2009
Central America, Mexico and Latin Caribbean		10·95 (9·45, 12·77)	11·28 (9·14, 13·75)	1258·16	0·92 (0·49, 2·02)	0·0032	1	2008
Costa Rica	CRI	10·20 (8·95, 11·56)	12·54 (10·44, 14·86)	84·12	-0·55 (-1·79, 0·79)	0·38	0	NA
Cuba	CUB	17·02 (15·20, 18·94)	16·18 (13·05, 19·69)	25·66	-0·95 (-1·81, -0·49)	0·0032	2	2007, 2010
Dominican Republic	DOM	11·74 (7·78, 16·87)	7·44 (4·62, 11·24)	-186·97	-6·99 (-9·59, -5·22)	<0·0001	2	2010, 2017
El Salvador	SLV	22·16 (16·58, 28·69)	13·95 (9·35, 19·83)	-251·09	-0·04 (-1·39, 1·38)	0·96	0	NA
Guatemala	GTM	9·28 (8·04, 10·61)	7·95 (6·27, 9·86)	-10·07	-0·11 (-1·57, 1·88)	0·94	2	2005, 2011
Haiti	HTI	14·10 (7·83, 22·11)	13·56 (7·68, 21·23)	46·06	-0·58 (-1·15, -0·21)	0·0084	2	2006, 2019
Honduras	HND	12·99 (7·37, 21·32)	6·62 (3·89, 10·47)	-286·45	-8·28 (-12·62, -5·22)	<0·0001	2	2010, 2013
Mexico	MEX	9·37 (8·97, 9·78)	11·54 (9·99, 13·13)	1790·72	2·54 (1·89, 4·21)	0·019	1	2007
Nicaragua	NIC	7·64 (5·90, 9·61)	6·92 (4·79, 9·56)	12·05	-2·55 (-3·30, -1·77)	<0·0001	0	NA
Panama	PAN	4·70 (4·02, 5·45)	5·64 (4·19, 7·23)	34·13	-4·88 (-8·49, -3·95)	0·0020	1	2003
Non-Latin Caribbean		15·14 (11·98, 18·76)	13·15 (9·38, 17·82)	-34·18	-1·70 (-3·26, -0·15)	0·038	2	2011, 2019
Antigua and Barbuda	ATG	0·00 (0·00, 0·00)	1·64 (1·27, 2·09)	0·72	---	---	---	---
Bahamas	BHS	5·92 (4·69, 7·35)	5·37 (3·95, 7·13)	-0·48	-2·74 (-5·21, -0·53)	0·010	1	2017
Barbados	BRB	6·32 (5·07, 7·80)	5·14 (3·67, 6·91)	-1·18	-1·70 (-2·31, -1·06)	<0·0001	0	NA
Belize	BLZ	11·45 (9·50, 13·71)	7·68 (6·07, 9·55)	-4·16	-5·67 (-7·90, -4·18)	<0·0001	2	2008, 2011
Dominica	DMA	6·92 (4·63, 9·71)	6·30 (3·96, 9·19)	-0·08	-0·40 (-0·71, -0·06)	0·020	0	NA
Grenada	GRD	0·70 (0·58, 0·82)	1·74 (1·35, 2·17)	0·72	---	---	---	---
Guyana	GUY	57·31 (44·78, 71·95)	43·94 (31·46, 59·88)	-33·32	-4·12 (-5·50, -2·32)	<0·0001	2	2008, 2015
Jamaica	JAM	3·13 (2·46, 3·92)	3·10 (2·17, 4·29)	1·76	1·41 (-0·90, 3·85)	0·23	0	NA
Saint Kitts and Nevis	KNA	4·32 (3·35, 5·45)	3·32 (2·41, 4·82)	-0·16	-3·28 (-4·32, -2·48)	<0·0001	2	2006, 2013
Saint Lucia	LCA	13·68 (11·15, 16·48)	8·65 (6·61, 11·02)	-4·21	-5·26 (-8·60, -1·91)	0·00040	1	2019
Saint Vincent and the Grenadines	VCT	4·66 (3·75, 5·73)	0·55 (0·45, 0·70)	-2·16	---	---	---	---
Suriname	SUR	41·89 (31·22, 53·76)	31·40 (21·30, 43·69)	-19·93	0·08 (-0·56, 0·72)	0·79	0	NA
Trinidad and Tobago	TTO	17·68 (14·85, 20·91)	19·60 (14·20, 26·12)	28·30	2·97 (1·37, 5·38)	0·00040	1	2012
North America		19·28 (18·56, 19·92)	21·27 (20·07, 22·38)	5487·78	1·49 (1·32, 1·84)	<0·0001	1	2006
Canada	CAN	16·94 (15·44, 18·45)	12·10 (10·62, 13·69)	-818·91	-4·07 (-6·72, -1·68)	0·00040	1	2019
United States of America	USA	19·54 (18·91, 20·09)	22·31 (21·13, 23·36)	6306·69	1·69 (1·47, 2·32)	<0·0001	1	2006
Southern Cone and Brazil		10·72 (9·88, 11·56)	11·15 (10·09, 12·23)	1915·57	0·69 (0·42, 1·50)	0·014	1	2005
Argentina	ARG	14·07 (12·42, 15·81)	12·17 (10·41, 14·02)	-241·67	-3·00 (-4·83, -1·45)	0·0016	2	2006, 2019
Brazil	BRA	9·30 (8·80, 9·78)	10·62 (9·88, 11·34)	2310·06	1·25 (0·99, 1·52)	<0·0001	0	NA
Chile	CHL	15·04 (13·65, 16·50)	10·84 (9·41, 12·34)	-287·13	-3·88 (-5·12, -2·73)	<0·0001	2	2005, 2009
Paraguay	PRY	9·63 (6·55, 12·49)	8·45 (5·35, 12·32)	-24·94	-1·85 (-5·81, 2·35)	0·28	1	2019

Uruguay	URY	27·80 (24·77, 31·06)	35·99 (31·15, 41·29)	159·25	4·14 (2·91, 6·45)	<0·0001	1	2009
REGION OF THE AMERICAS		13·79 (12·78, 14·84)	14·66 (13·17, 16·24)	9916·36	0·99 (0·83, 1·23)	<0·0001	1	2006

AAPC Average annual percent change; --- Zeros present in the time-series (AAPC not estimated); NA Not applicable

Note: AAPCs represent model-based estimates of temporal change derived from the fitted log-linear joinpoint regression using all annual observations over the specified interval and therefore may differ from changes calculated directly from the observed values at the beginning and end of the interval.

Table S11. Sensitivity analysis: Confidence intervals obtained after propagating uncertainty in the annual suicide mortality estimates through repeated simulation for females

Subregion and Country	ISO3 country code	Age-standardized suicide mortality rate per 100,000 population		Estimated change in number of deaths, 2015–2021	AAPC		No. of joinpoints	Location of joinpoint(s)
		2015	2021		2015-2021	p-value		
Andean Area		2·01 (1·58, 2·52)	1·82 (1·32, 2·45)	-50·07	-1·52 (-3·70, 0·41)	0·11	2	2014, 2019
Bolivia	BOL	4·35 (2·40, 7·09)	2·64 (1·43, 4·38)	-88·75	-8·38 (-9·71, -7·67)	<0·0001	2	2008, 2019
Colombia	COL	1·85 (1·61, 2·09)	2·02 (1·61, 2·49)	89·45	4·69 (3·06, 6·85)	<0·0001	1	2012
Ecuador	ECU	4·11 (3·48, 4·84)	2·56 (1·95, 3·31)	-108·05	-3·06 (-4·45, -1·94)	<0·0001	1	2006
Peru	PER	0·69 (0·43, 0·97)	0·80 (0·46, 1·24)	26·87	1·17 (-1·36, 3·85)	0·36	0	NA
Venezuela	VEN	1·69 (1·43, 1·99)	2·02 (1·44, 2·75)	30·41	2·05 (0·39, 5·30)	0·018	1	2011
Central America, Mexico and Latin Caribbean		2·68 (2·22, 3·19)	2·63 (2·02, 3·32)	164·36	0·75 (0·35, 1·58)	0·0016	1	2006
Costa Rica	CRI	2·16 (1·82, 2·54)	2·73 (2·21, 3·30)	16·80	6·18 (1·46, 12·79)	0·030	2	2003, 2013
Cuba	CUB	4·07 (3·54, 4·66)	2·84 (2·27, 3·48)	-79·20	-6·08 (-9·89, -3·18)	<0·0001	2	2006, 2017
Dominican Republic	DOM	1·98 (1·25, 2·96)	1·50 (0·92, 2·34)	-17·49	-4·65 (-6·04, -3·45)	<0·0001	2	2010, 2018
El Salvador	SLV	3·26 (2·36, 4·22)	3·07 (2·01, 4·43)	-4·87	-0·98 (-1·55, -0·36)	0·0016	0	NA
Guatemala	GTM	2·95 (2·53, 3·42)	2·80 (2·23, 3·46)	7·39	1·30 (0·01, 3·00)	0·048	2	2002, 2011
Haiti	HTI	6·17 (2·48, 10·19)	5·47 (2·32, 9·08)	-12·70	-1·86 (-2·69, -1·34)	<0·0001	2	2016, 2019
Honduras	HND	1·45 (0·75, 2·53)	1·05 (0·54, 1·85)	-8·31	-4·56 (-6·52, -3·04)	<0·0001	1	2019
Mexico	MEX	2·27 (2·15, 2·39)	2·56 (2·17, 2·98)	283·53	3·32 (2·78, 3·87)	<0·0001	0	NA
Nicaragua	NIC	2·35 (1·68, 3·05)	1·62 (1·05, 2·36)	-20·39	-5·12 (-7·11, -4·14)	<0·0001	2	2006, 2009
Panama	PAN	1·13 (0·95, 1·35)	0·98 (0·75, 1·24)	-0·40	-3·90 (-5·89, -1·76)	0·00040	0	NA
Non-Latin Caribbean		3·86 (2·95, 4·97)	3·41 (2·34, 4·76)	-9·21	-2·18 (-3·22, -0·87)	0·00080	2	2006, 2015
Antigua and Barbuda	ATG	0·77 (0·60, 0·95)	0·97 (0·82, 1·14)	0·20	---	---	---	---
Bahamas	BHS	0·70 (0·55, 0·87)	0·75 (0·53, 1·01)	0·20	-0·85 (-1·44, -0·22)	0·0084	0	NA
Barbados	BRB	1·04 (0·82, 1·29)	0·86 (0·63, 1·15)	-0·22	-0·40 (-0·87, 0·09)	0·12	0	NA
Belize	BLZ	1·64 (1·34, 1·97)	1·61 (1·30, 2·01)	0·30	-3·56 (-4·79, -2·80)	<0·0001	2	2007, 2010
Dominica	DMA	0·78 (0·45, 1·17)	0·92 (0·51, 1·49)	0·05	-0·51 (-0·94, -0·06)	0·026	0	NA
Grenada	GRD	0·25 (0·20, 0·29)	0·54 (0·45, 0·68)	0·05	---	---	---	---
Guyana	GUY	15·66 (12·00, 19·91)	10·56 (7·43, 14·38)	-16·83	-5·96 (-8·20, -3·37)	<0·0001	1	2016
Jamaica	JAM	0·38 (0·31, 0·47)	0·39 (0·28, 0·53)	0·61	1·04 (0·20, 3·37)	0·031	1	2004
Saint Kitts and Nevis	KNA	0·68 (0·56, 0·83)	0·62 (0·49, 0·72)	-0·01	-3·53 (-5·37, -2·66)	<0·0001	2	2003, 2011
Saint Lucia	LCA	1·10 (0·88, 1·34)	1·13 (0·86, 1·46)	0·11	-3·08 (-5·08, -2·14)	0·00040	2	2007, 2010
Saint Vincent and the Grenadines	VCT	0·50 (0·40, 0·65)	0·00 (0·00, 0·00)	-0·24	---	---	---	---
Suriname	SUR	13·40 (9·25, 19·13)	14·16 (8·95, 20·99)	5·54	1·05 (0·72, 1·39)	<0·0001	0	NA
Trinidad and Tobago	TTO	4·30 (3·54, 5·16)	4·13 (2·93, 5·62)	1·03	-1·28 (-2·41, -0·17)	0·030	2	2006, 2016
North America		6·10 (5·83, 6·34)	5·75 (5·39, 6·11)	-526·31	-0·56 (-1·19, 0·02)	0·059	1	2017
Canada	CAN	6·31 (5·69, 6·97)	4·07 (3·55, 4·64)	-379·05	-4·89 (-7·38, -3·11)	<0·0001	1	2019
United States of America	USA	6·08 (5·85, 6·28)	5·95 (5·61, 6·28)	-147·26	-0·22 (-0·96, 0·42)	0·47	1	2017
Southern Cone and Brazil		2·89 (2·64, 3·14)	3·58 (3·21, 3·95)	1327·20	3·21 (1·79, 4·33)	<0·0001	1	2016
Argentina	ARG	3·37 (2·96, 3·82)	3·36 (2·86, 3·90)	24·42	0·29 (-1·08, 1·77)	0·33	2	2002, 2006
Brazil	BRA	2·63 (2·46, 2·78)	3·56 (3·28, 3·81)	1284·20	4·73 (3·30, 5·83)	<0·0001	2	2002, 2016
Chile	CHL	3·98 (3·49, 4·50)	2·91 (2·48, 3·37)	-78·92	-3·46 (-4·55, -2·52)	0·0016	2	2005, 2008
Paraguay	PRY	3·57 (2·32, 4·90)	4·73 (2·94, 6·97)	46·87	2·26 (1·00, 3·62)	0·0016	0	NA

Uruguay	URY	6·77 (5·81, 7·83)	8·90 (7·51, 10·38)	50·63	1·61 (0·71, 2·56)	0·0016	0	NA
REGION OF THE AMERICAS		4·00 (3·66, 4·35)	3·95 (3·50, 4·43)	905·97	0·02 (-0·89, 0·89)	0·90	1	2018

AAPC Average annual percent change; --- Zeros present in the time-series (AAPC not estimated); NA Not applicable

Note: AAPCs represent model-based estimates of temporal change derived from the fitted log-linear joinpoint regression using all annual observations over the specified interval and therefore may differ from changes calculated directly from the observed values at the beginning and end of the interval.

Table S12. Country-specific progress classifications for the primary analyses and sensitivity analyses

Country and Subregion	Both sexes			Males			Females		
	Primary analysis	Sensitivity analyses		Primary analysis	Sensitivity analyses		Primary analysis	Sensitivity analyses	
		Empirical CI	Timeseries 2000-2019		Empirical CI	Timeseries 2000-2019		Empirical CI	Timeseries 2000-2019
Andean Area	No progress	No progress	Worsening situation	No progress	No progress	Worsening situation	No progress	No progress	No progress
Bolivia	Substantial progress	Substantial progress	Fair progress	No progress	No progress	No progress	Substantial progress	Substantial progress	Fair progress
Colombia	Worsening situation	Worsening situation	Worsening situation	No progress	Worsening situation	Worsening situation	Worsening situation	Worsening situation	Worsening situation
Ecuador	No progress	No progress	Worsening situation	No progress	No progress	Worsening situation	Substantial progress	Substantial progress	Fair progress
Peru	No progress	No progress	No progress	No progress	No progress	No progress	No progress	No progress	No progress
Venezuela	Worsening situation	Worsening situation	Worsening situation	Worsening situation	Worsening situation	Worsening situation	Worsening situation	Worsening situation	No progress
Central America, Mexico and Latin Caribbean	Worsening situation	Worsening situation	Worsening situation	Worsening situation	Worsening situation	Worsening situation	Worsening situation	Worsening situation	Worsening situation
Costa Rica	No progress	No progress	No progress	No progress	No progress	No progress	Worsening situation	Worsening situation	No progress
Cuba	Fair progress	Fair progress	Fair progress	Fair progress	Fair progress	Fair progress	Substantial progress	Substantial progress	Fair progress
Dominican Republic	Substantial progress	Substantial progress	No progress	Substantial progress	Substantial progress	No progress	Substantial progress	Substantial progress	No progress
El Salvador	No progress	No progress	No progress	No progress	No progress	No progress	Fair progress	Fair progress	Fair progress
Guatemala	No progress	No progress	Worsening situation	No progress	No progress	Worsening situation	Worsening situation	Worsening situation	Worsening situation
Haiti	No progress	Fair progress	No progress	No progress	Fair progress	Worsening situation	No progress	Fair progress	No progress
Honduras	Substantial progress	Substantial progress	Substantial progress	Substantial progress	Substantial progress	Substantial progress	Substantial progress	Substantial progress	No progress
Mexico	Worsening situation	Worsening situation	Worsening situation	Worsening situation	Worsening situation	Worsening situation	Worsening situation	Worsening situation	Worsening situation
Nicaragua	Substantial progress	Substantial progress	Fair progress	Fair progress	Fair progress	Fair progress	Substantial progress	Substantial progress	Substantial progress
Panama	Substantial progress	Substantial progress	Substantial progress	Substantial progress	Substantial progress	Substantial progress	Substantial progress	Substantial progress	Substantial progress
Non-Latin Caribbean	No progress	Fair progress	No progress	No progress	Fair progress	No progress	Fair progress	Fair progress	Substantial progress
Antigua and Barbuda	NA	NA	NA	NA	NA	NA	NA	NA	NA
Bahamas	No progress	Substantial progress	No progress	No progress	Substantial progress	No progress	Fair progress	Fair progress	Fair progress
Barbados	Fair progress	Fair progress	Fair progress	Fair progress	Fair progress	Fair progress	No progress	No progress	No progress
Belize	Substantial progress	Substantial progress	Substantial progress	Substantial progress	Substantial progress	Substantial progress	Substantial progress	Substantial progress	Substantial progress
Dominica	Fair progress	Fair progress	No progress	Fair progress	Fair progress	No progress	Fair progress	Fair progress	No progress
Grenada	No progress	No progress	Substantial progress	NA	NA	NA	NA	NA	NA

Guyana	Substantial progress	Substantial progress	Substantial progress	Substantial progress	Substantial progress	Substantial progress	Substantial progress	Substantial progress	Substantial progress
Jamaica	No progress	No progress	No progress	No progress	No progress	No progress	Worsening situation	Worsening situation	No progress
Saint Kitts and Nevis	Substantial progress	Substantial progress	Substantial progress	Substantial progress	Substantial progress	Fair progress	Substantial progress	Substantial progress	Substantial progress
Saint Lucia	No progress	Substantial progress	Fair progress	No progress	Substantial progress	Fair progress	Substantial progress	Substantial progress	Substantial progress
Saint Vincent and the Grenadines	NA	NA	NA	NA	NA	NA	NA	NA	NA
Suriname	No progress	No progress	Worsening situation	No progress	No progress	Worsening situation	Worsening situation	Worsening situation	No progress
Trinidad and Tobago	Worsening situation	Worsening situation	Worsening situation	Worsening situation	Worsening situation	Worsening situation	No progress	Fair progress	No progress
North America	Worsening situation	Worsening situation	Worsening situation	Worsening situation	Worsening situation	Worsening situation	No progress	No progress	No progress
Canada	Substantial progress	Substantial progress	No progress	No progress	Substantial progress	Fair progress	Substantial progress	Substantial progress	Worsening situation
United States	Worsening situation	Worsening situation	Worsening situation	Worsening situation	Worsening situation	Worsening situation	No progress	No progress	No progress
Southern Cone and Brazil	Worsening situation	Worsening situation	Worsening situation	Worsening situation	Worsening situation	Worsening situation	Worsening situation	Worsening situation	No progress
Argentina	No progress	Fair progress	No progress	No progress	Substantial progress	No progress	No progress	No progress	No progress
Brazil	Worsening situation	Worsening situation	Worsening situation	Worsening situation	Worsening situation	Worsening situation	Worsening situation	Worsening situation	Worsening situation
Chile	Substantial progress	Substantial progress	Substantial progress	Substantial progress	Substantial progress	Substantial progress	Substantial progress	Substantial progress	Substantial progress
Paraguay	Worsening situation	Worsening situation	Worsening situation	No progress	No progress	Worsening situation	Worsening situation	Worsening situation	Worsening situation
Uruguay	Worsening situation	Worsening situation	Worsening situation	Worsening situation	Worsening situation	Worsening situation	Worsening situation	Worsening situation	Worsening situation
REGION OF THE AMERICAS	Worsening situation	Worsening situation	Worsening situation	Worsening situation	Worsening situation	Worsening situation	No progress	No progress	Worsening situation

CI Confidence interval; NA Not applicable

Table S13. Sensitivity analysis (excluding 2020 and 2021): Age-standardized suicide mortality rate in 2015 and 2019, and the average annual percent change from 2015 to 2019 in the Region of the Americas by country and sub-region for both sexes combined

Subregion and Country	ISO3 country code	Age-standardized suicide mortality rate per 100,000 population		Estimated change in number of deaths, 2015–2021	AAPC		No. of joinpoints	Location of joinpoint(s)
		2015	2019		2015-2021	p-value		
Andean Area		4·53 (3·71, 5·50)	5·24 (4·08, 6·64)	1349·06	3·63 (0·99, 6·34)	0·010	1	2013
Bolivia	BOL	6·28 (3·53, 10·05)	5·92 (3·35, 9·49)	7·71	-0·56 (-1·06, -0·05)	0·033	2	2009, 2012
Colombia	COL	4·08 (3·60, 4·58)	4·88 (4·24, 5·57)	608·88	5·11 (3·93, 6·31)	<0·0001	1	2013
Ecuador	ECU	8·51 (7·21, 9·91)	8·45 (7·01, 10·07)	113·10	4·44 (0·46, 8·59)	0·031	2	2008, 2013
Peru	PER	0·96 (0·63, 1·31)	2·16 (1·37, 3·10)	416·03	2·12 (-0·56, 4·88)	0·11	0	NA
Venezuela	VEN	6·27 (5·25, 7·45)	7·42 (5·57, 9·78)	203·34	3·59 (0·32, 6·97)	0·033	1	2010
Central America, Mexico and Latin Caribbean		6·67 (5·71, 7·81)	6·58 (5·66, 7·66)	621·26	0·86 (0·47, 1·25)	0·00030	1	2007
Costa Rica	CRI	6·16 (5·36, 7·02)	6·71 (5·79, 7·72)	46·34	-0·45 (-1·55, 0·67)	0·41	0	NA
Cuba	CUB	10·37 (9·22, 11·61)	9·55 (8·25, 10·94)	-43·19	-1·65 (-2·54, -0·75)	0·0018	2	2007, 2010
Dominican Republic	DOM	6·83 (4·50, 9·88)	6·06 (3·77, 9·07)	-37·52	-2·97 (-6·26, 0·44)	0·081	2	2011, 2015
El Salvador	SLV	11·92 (8·85, 15·46)	7·99 (5·70, 10·78)	-248·92	-0·05 (-1·14, 1·04)	0·92	0	NA
Guatemala	GTM	5·98 (5·17, 6·86)	6·31 (5·40, 7·33)	134·03	2·58 (0·91, 4·28)	0·0054	2	2004, 2012
Haiti	HTI	9·89 (5·01, 15·78)	10·11 (5·19, 16·15)	99·07	0·88 (-0·86, 2·65)	0·32	2	2006, 2017
Honduras	HND	7·16 (4·01, 11·85)	3·91 (2·23, 6·31)	-297·88	-9·94 (-15·01, -4·56)	0·0020	2	2010, 2013
Mexico	MEX	5·69 (5·43, 5·95)	6·14 (5·85, 6·45)	959·67	2·08 (1·75, 2·41)	<0·0001	0	NA
Nicaragua	NIC	4·86 (3·68, 6·16)	4·71 (3·45, 6·20)	11·27	-2·54 (-3·05, -2·02)	<0·0001	0	NA
Panama	PAN	2·86 (2·43, 3·33)	2·57 (2·15, 3·04)	-1·61	-5·89 (-7·09, -4·67)	<0·0001	1	2006
Non-Latin Caribbean		9·31 (7·31, 11·63)	9·01 (6·70, 11·81)	15·01	0·26 (-0·20, 0·71)	0·25	1	2006
Antigua and Barbuda	ATG	0·42 (0·33, 0·52)	0·00 (0·00, 0·00)	-0·42	---	---	---	---
Bahamas	BHS	3·16 (2·51, 3·93)	3·29 (2·47, 4·27)	1·07	0·14 (-0·29, 0·58)	0·50	0	NA
Barbados	BRB	3·49 (2·79, 4·31)	4·16 (3·14, 5·37)	2·22	-1·29 (-1·89, -0·68)	0·00032	0	NA
Belize	BLZ	6·53 (5·41, 7·83)	5·55 (4·46, 6·83)	-1·00	-3·94 (-5·93, -1·91)	0·0013	2	2007, 2011
Dominica	DMA	3·81 (2·51, 5·37)	3·89 (2·56, 5·71)	0·22	-0·21 (-0·53, 0·11)	0·18	0	NA
Grenada	GRD	0·50 (0·41, 0·60)	0·85 (0·70, 1·02)	0·44	-10·76 (-16·57, -4·56)	0·0023	0	NA
Guyana	GUY	35·63 (27·71, 44·86)	29·42 (21·46, 39·07)	-29·00	-5·19 (-8·71, -1·54)	0·0057	2	2008, 2016
Jamaica	JAM	1·69 (1·33, 2·10)	1·68 (1·18, 2·31)	2·04	1·38 (-1·11, 3·92)	0·26	0	NA
Saint Kitts and Nevis	KNA	2·36 (1·82, 3·02)	2·21 (1·54, 3·03)	-0·04	-2·71 (-3·71, -1·70)	<0·0001	2	2006, 2012
Saint Lucia	LCA	7·22 (5·90, 8·73)	6·57 (5·17, 8·21)	-0·65	-2·20 (-3·64, -0·74)	0·0067	2	2008, 2012
Saint Vincent and the Grenadines	VCT	2·65 (2·12, 3·24)	0·23 (0·19, 0·29)	-2·47	---	---	---	---
Suriname	SUR	27·27 (19·98, 35·94)	26·83 (19·06, 36·46)	7·51	0·73 (0·45, 1·00)	<0·0001	2	2002, 2005
Trinidad and Tobago	TTO	10·69 (8·95, 12·68)	12·17 (9·58, 15·18)	35·09	3·03 (1·21, 4·89)	0·0033	2	2005, 2012
North America		12·59 (12·11, 13·03)	13·13 (12·54, 13·69)	3902·16	1·77 (1·58, 1·96)	<0·0001	1	2006
Canada	CAN	11·59 (10·54, 12·67)	11·05 (9·84, 12·31)	-34·04	-0·17 (-0·52, 0·17)	0·31	0	NA
United States of America	USA	12·71 (12·29, 13·08)	13·37 (12·85, 13·84)	3936·20	1·94 (1·72, 2·17)	<0·0001	1	2006
Southern Cone and Brazil		6·64 (6·11, 7·17)	7·04 (6·42, 7·67)	2228·80	1·49 (0·67, 2·31)	0·0014	1	2011
Argentina	ARG	8·56 (7·55, 9·62)	9·06 (7·85, 10·34)	356·16	0·55 (-0·01, 1·10)	0·052	2	2002, 2005
Brazil	BRA	5·81 (5·49, 6·12)	6·19 (5·83, 6·53)	1579·90	1·31 (1·07, 1·56)	<0·0001	0	NA
Chile	CHL	9·40 (8·48, 10·38)	8·71 (7·68, 9·81)	14·26	-2·90 (-3·79, -2·00)	<0·0001	2	2005, 2008
Paraguay	PRY	6·60 (4·43, 8·69)	8·08 (5·33, 11·14)	113·94	3·55 (2·82, 4·29)	<0·0001	0	NA

Uruguay	URY	16·76 (14·83, 18·84)	21·05 (18·34, 23·97)	164·54	4·65 (2·30, 7·06)	0·00068	1	2010
REGION OF THE AMERICAS		8·75 (8·09, 9·44)	9·07 (8·30, 9·89)	8116·29	1·25 (1·02, 1·47)	<0·0001	1	2007

AAPC Average annual percent change; --- Zeros present in the time-series (AAPC not estimated); NA Not applicable

Note: AAPCs represent model-based estimates of temporal change derived from the fitted log-linear joinpoint regression using all annual observations over the specified interval and therefore may differ from changes calculated directly from the observed values at the beginning and end of the interval.

Table S14. Sensitivity analysis (excluding 2020 and 2021): Age-standardized suicide mortality rate in 2015 and 2019, and the average annual percent change from 2015 to 2019 in the Region of the Americas by country and sub-region for males

Subregion and Country	ISO3 country code	Age-standardized suicide mortality rate per 100,000 population		Estimated change in number of deaths, 2015–2021	AAPC		No. of joinpoints	Location of joinpoint(s)
		2015	2019		2015-2021	p-value		
Andean Area		7·20 (5·95, 8·65)	8·42 (6·62, 10·59)	1144·14	4·10 (1·03, 7·25)	0·012	1	2013
Bolivia	BOL	8·37 (4·77, 13·24)	8·05 (4·65, 12·63)	16·54	-0·81 (-2·18, 0·58)	0·25	2	2013, 2016
Colombia	COL	6·55 (5·80, 7·34)	7·74 (6·74, 8·82)	473·08	4·46 (2·93, 6·00)	<0·0001	1	2013
Ecuador	ECU	13·12 (11·13, 15·23)	13·67 (11·35, 16·27)	153·69	5·94 (1·54, 10·54)	0·012	2	2008, 2013
Peru	PER	1·25 (0·85, 1·68)	2·99 (1·93, 4·24)	297·99	2·18 (-0·57, 5·01)	0·11	0	NA
Venezuela	VEN	11·15 (9·33, 13·26)	13·36 (10·04, 17·59)	202·84	3·79 (0·38, 7·32)	0·031	1	2010
Central America, Mexico and Latin Caribbean		10·95 (9·45, 12·77)	10·85 (9·43, 12·53)	546·02	0·92 (0·41, 1·44)	0·0017	1	2008
Costa Rica	CRI	10·20 (8·95, 11·56)	11·54 (9·99, 13·21)	49·74	-0·76 (-1·87, 0·36)	0·17	0	NA
Cuba	CUB	17·02 (15·20, 18·94)	16·36 (14·16, 18·71)	2·14	-1·22 (-2·18, -0·25)	0·020	2	2007, 2010
Dominican Republic	DOM	11·74 (7·78, 16·87)	10·21 (6·37, 15·16)	-42·90	-3·46 (-7·35, 0·59)	0·087	2	2011, 2015
El Salvador	SLV	22·16 (16·58, 28·69)	14·07 (10·12, 18·92)	-246·80	0·30 (-0·92, 1·53)	0·61	0	NA
Guatemala	GTM	9·28 (8·04, 10·61)	9·89 (8·49, 11·48)	111·68	2·59 (0·83, 4·37)	0·0073	2	2005, 2012
Haiti	HTI	14·10 (7·83, 22·11)	14·61 (8·19, 22·95)	81·78	0·71 (0·49, 0·94)	<0·0001	1	2006
Honduras	HND	12·99 (7·37, 21·32)	6·70 (3·92, 10·56)	-299·45	-11·08 (-16·62, -5·16)	0·0019	2	2010, 2013
Mexico	MEX	9·37 (8·97, 9·78)	10·26 (9·78, 10·76)	858·93	2·31 (1·72, 2·90)	<0·0001	1	2007
Nicaragua	NIC	7·64 (5·90, 9·61)	7·71 (5·73, 10·08)	19·54	-2·12 (-2·70, -1·53)	<0·0001	0	NA
Panama	PAN	4·70 (4·02, 5·45)	4·81 (4·03, 5·67)	11·36	-5·90 (-7·22, -4·57)	<0·0001	1	2006
Non-Latin Caribbean		15·14 (11·98, 18·76)	15·02 (11·26, 19·56)	28·37	0·99 (-0·14, 2·14)	0·082	1	2011
Antigua and Barbuda	ATG	0·00 (0·00, 0·00)	0·00 (0·00, 0·00)	0·00	---	---	---	---
Bahamas	BHS	5·92 (4·69, 7·35)	6·14 (4·62, 7·95)	0·87	0·29 (-0·13, 0·70)	0·16	0	NA
Barbados	BRB	6·32 (5·07, 7·80)	7·58 (5·72, 9·78)	2·00	-1·50 (-2·15, -0·84)	0·00015	0	NA
Belize	BLZ	11·45 (9·50, 13·71)	9·52 (7·69, 11·73)	-1·15	-4·05 (-6·26, -1·79)	0·0023	2	2008, 2011
Dominica	DMA	6·92 (4·63, 9·71)	7·14 (4·64, 10·35)	0·27	-0·21 (-0·52, 0·11)	0·19	0	NA
Grenada	GRD	0·70 (0·58, 0·82)	0·91 (0·75, 1·10)	0·27	---	---	---	---
Guyana	GUY	57·31 (44·78, 71·95)	49·48 (36·28, 65·54)	-14·99	-3·19 (-5·88, -0·43)	0·027	2	2009, 2014
Jamaica	JAM	3·13 (2·46, 3·92)	3·11 (2·19, 4·29)	1·45	1·56 (-1·08, 4·27)	0·23	0	NA
Saint Kitts and Nevis	KNA	4·32 (3·35, 5·45)	3·92 (2·74, 5·62)	-0·02	-2·44 (-3·39, -1·48)	0·00014	2	2006, 2012
Saint Lucia	LCA	13·68 (11·15, 16·48)	12·43 (9·82, 15·56)	-0·69	-1·77 (-3·27, -0·25)	0·026	2	2008, 2012
Saint Vincent and the Grenadines	VCT	4·66 (3·75, 5·73)	0·44 (0·36, 0·55)	-2·25	---	---	---	---
Suriname	SUR	41·89 (31·22, 53·76)	41·97 (30·36, 55·87)	7·59	0·74 (0·41, 1·08)	0·00042	2	2002, 2006
Trinidad and Tobago	TTO	17·68 (14·85, 20·91)	20·68 (16·32, 25·73)	35·02	3·90 (1·50, 6·35)	0·0033	1	2012
North America		19·28 (18·56, 19·92)	20·42 (19·53, 21·26)	3978·22	1·52 (1·30, 1·74)	<0·0001	1	2006
Canada	CAN	16·94 (15·44, 18·45)	16·48 (14·72, 18·33)	50·55	-0·45 (-0·82, -0·08)	0·021	0	NA
United States of America	USA	19·54 (18·91, 20·09)	20·87 (20·08, 21·59)	3927·67	1·67 (1·42, 1·92)	<0·0001	1	2006
Southern Cone and Brazil		10·72 (9·88, 11·56)	11·18 (10·21, 12·18)	1594·35	1·24 (0·56, 1·94)	0·0015	1	2010
Argentina	ARG	14·07 (12·42, 15·81)	14·88 (12·90, 16·98)	318·69	0·44 (-0·08, 0·96)	0·090	2	2002, 2005
Brazil	BRA	9·30 (8·80, 9·78)	9·68 (9·13, 10·20)	1016·98	1·19 (0·91, 1·47)	<0·0001	0	NA
Chile	CHL	15·04 (13·65, 16·50)	14·03 (12·44, 15·74)	29·00	-2·87 (-3·76, -1·97)	<0·0001	2	2005, 2008
Paraguay	PRY	9·63 (6·55, 12·49)	12·22 (8·15, 16·78)	90·68	4·23 (3·40, 5·06)	<0·0001	0	NA

Uruguay	URY	27·80 (24·77, 31·06)	35·45 (31·04, 40·21)	139·00	4·74 (2·24, 7·30)	0·0010	1	2010
REGION OF THE AMERICAS		13·79 (12·78, 14·84)	14·41 (13·24, 15·66)	7291·10	1·14 (0·91, 1·37)	<0·0001	1	2007

AAPC Average annual percent change; --- Zeros present in the time-series (AAPC not estimated); NA Not applicable

Note: AAPCs represent model-based estimates of temporal change derived from the fitted log-linear joinpoint regression using all annual observations over the specified interval and therefore may differ from changes calculated directly from the observed values at the beginning and end of the interval.

Table S15. Sensitivity analysis (excluding 2020 and 2021): Age-standardized suicide mortality rate in 2015 and 2019, and the average annual percent change from 2015 to 2019 in the Region of the Americas by country and sub-region for females

Subregion and Country	ISO3 country code	Age-standardized suicide mortality rate per 100,000 population		Estimated change in number of deaths, 2015–2021	AAPC		No. of joinpoints	Location of joinpoint(s)
		2015	2019		2015-2021	p-value		
Andean Area		2·01 (1·58, 2·52)	2·22 (1·68, 2·89)	209·00	2·80 (-0·21, 5·90)	0·070	1	2014
Bolivia	BOL	4·35 (2·40, 7·09)	3·95 (2·15, 6·59)	-8·51	-1·95 (-2·16, -1·74)	<0·0001	1	2007
Colombia	COL	1·85 (1·61, 2·09)	2·26 (1·94, 2·60)	136·06	7·31 (4·10, 10·61)	0·00017	1	2013
Ecuador	ECU	4·11 (3·48, 4·84)	3·38 (2·79, 4·04)	-39·65	-2·50 (-4·06, -0·91)	0·0044	1	2006
Peru	PER	0·69 (0·43, 0·97)	1·38 (0·83, 2·02)	119·76	2·10 (-0·74, 5·03)	0·14	0	NA
Venezuela	VEN	1·69 (1·43, 1·99)	1·80 (1·34, 2·36)	1·34	2·09 (-0·44, 4·68)	0·10	1	2011
Central America, Mexico and Latin Caribbean		2·68 (2·22, 3·19)	2·59 (2·14, 3·11)	58·59	0·91 (0·42, 1·41)	0·0014	1	2006
Costa Rica	CRI	2·16 (1·82, 2·54)	1·94 (1·62, 2·29)	-4·01	1·05 (-0·43, 2·55)	0·16	0	NA
Cuba	CUB	4·07 (3·54, 4·66)	3·12 (2·67, 3·63)	-45·42	-1·70 (-3·03, -0·34)	0·017	1	2006
Dominican Republic	DOM	1·98 (1·25, 2·96)	1·99 (1·22, 3·07)	6·62	0·69 (-1·60, 3·03)	0·53	2	2011, 2014
El Salvador	SLV	3·26 (2·36, 4·22)	3·18 (2·22, 4·29)	-1·49	-0·97 (-1·66, -0·27)	0·0095	0	NA
Guatemala	GTM	2·95 (2·53, 3·42)	3·01 (2·56, 3·51)	22·62	2·23 (0·90, 3·59)	0·0033	2	2002, 2011
Haiti	HTI	6·17 (2·48, 10·19)	6·10 (2·51, 10·10)	17·33	0·11 (-1·13, 1·36)	0·86	1	2016
Honduras	HND	1·45 (0·75, 2·53)	1·34 (0·68, 2·36)	2·05	0·24 (-0·80, 1·29)	0·63	2	2005, 2012
Mexico	MEX	2·27 (2·15, 2·39)	2·29 (2·17, 2·41)	81·77	3·42 (2·82, 4·02)	<0·0001	0	NA
Nicaragua	NIC	2·35 (1·68, 3·05)	1·97 (1·37, 2·67)	-8·30	-4·89 (-6·92, -2·81)	0·00029	2	2006, 2009
Panama	PAN	1·13 (0·95, 1·35)	0·42 (0·35, 0·51)	-12·58	-4·26 (-6·56, -1·90)	0·0015	0	NA
Non-Latin Caribbean		3·86 (2·95, 4·97)	3·38 (2·43, 4·55)	-13·36	-3·58 (-6·44, -0·63)	0·018	2	2006, 2016
Antigua and Barbuda	ATG	0·77 (0·60, 0·95)	0·00 (0·00, 0·00)	-0·42	---	---	---	---
Bahamas	BHS	0·70 (0·55, 0·87)	0·75 (0·56, 0·99)	0·19	-0·74 (-1·44, -0·03)	0·041	0	NA
Barbados	BRB	1·04 (0·82, 1·29)	1·15 (0·87, 1·47)	0·20	-0·12 (-0·58, 0·35)	0·60	0	NA
Belize	BLZ	1·64 (1·34, 1·97)	1·59 (1·25, 1·94)	0·16	-4·33 (-5·77, -2·88)	<0·0001	2	2007, 2010
Dominica	DMA	0·78 (0·45, 1·17)	0·82 (0·46, 1·28)	0·02	0·00 (-0·53, 0·53)	0·99	1	2007
Grenada	GRD	0·25 (0·20, 0·29)	0·74 (0·62, 0·89)	0·16	---	---	---	---
Guyana	GUY	15·66 (12·00, 19·91)	11·13 (7·95, 14·91)	-14·04	-8·38 (-14·27, -2·09)	0·010	1	2016
Jamaica	JAM	0·38 (0·31, 0·47)	0·40 (0·28, 0·53)	0·52	1·08 (-0·03, 2·20)	0·056	1	2004
Saint Kitts and Nevis	KNA	0·68 (0·56, 0·83)	0·64 (0·47, 0·76)	-0·01	-4·42 (-6·30, -2·51)	0·00033	2	2003, 2012
Saint Lucia	LCA	1·10 (0·88, 1·34)	1·11 (0·83, 1·39)	0·04	-4·05 (-5·45, -2·63)	<0·0001	2	2007, 2010
Saint Vincent and the Grenadines	VCT	0·50 (0·40, 0·65)	0·00 (0·00, 0·00)	-0·24	---	---	---	---
Suriname	SUR	13·40 (9·25, 19·13)	12·53 (8·31, 18·21)	-0·08	-1·30 (-3·72, 1·19)	0·30	2	2011, 2016
Trinidad and Tobago	TTO	4·30 (3·54, 5·16)	4·11 (3·19, 5·17)	0·14	-2·10 (-5·80, 1·74)	0·28	2	2006, 2016
North America		6·10 (5·83, 6·34)	5·97 (5·67, 6·26)	14·42	0·57 (-2·04, 3·26)	0·67	1	2017
Canada	CAN	6·31 (5·69, 6·97)	5·64 (4·98, 6·33)	-84·70	0·58 (0·18, 0·97)	0·0065	0	NA
United States of America	USA	6·08 (5·85, 6·28)	6·02 (5·76, 6·26)	99·12	0·75 (-1·86, 3·44)	0·58	1	2017
Southern Cone and Brazil		2·89 (2·64, 3·14)	3·21 (2·91, 3·51)	651·85	3·19 (-0·18, 6·67)	0·064	1	2016
Argentina	ARG	3·37 (2·96, 3·82)	3·50 (3·02, 4·02)	40·51	0·69 (-0·19, 1·57)	0·11	2	2002, 2006
Brazil	BRA	2·63 (2·46, 2·78)	3·02 (2·83, 3·19)	579·72	4·01 (1·36, 6·73)	0·0029	2	2002, 2016
Chile	CHL	3·98 (3·49, 4·50)	3·53 (3·04, 4·05)	-16·31	-3·22 (-4·69, -1·73)	0·00055	2	2005, 2008
Paraguay	PRY	3·57 (2·32, 4·90)	4·07 (2·59, 5·67)	22·86	2·00 (0·81, 3·20)	0·0022	0	NA

Uruguay	URY	6·77 (5·81, 7·83)	7·53 (6·39, 8·77)	25·07	1·47 (0·52, 2·42)	0·0043	0	NA
REGION OF THE AMERICAS		4·00 (3·66, 4·35)	4·01 (3·63, 4·42)	920·50	1·18 (1·03, 1·33)	<0·0001	0	NA

AAPC Average annual percent change; --- Zeros present in the time-series (AAPC not estimated); NA Not applicable

Note: AAPCs represent model-based estimates of temporal change derived from the fitted log-linear joinpoint regression using all annual observations over the specified interval and therefore may differ from changes calculated directly from the observed values at the beginning and end of the interval.

Table S16. Sensitivity analysis: Average annual percent change in the proportion of suicides by method for 20 countries in the Region of the Americas for both sexes combined, males, and females, using a log-linear model with no joinpoint

Country; suicide method	Both sexes				Males				Females			
	Proportion (%)		AAPC (95% CI)		Proportion (%)		AAPC (95% CI)		Proportion (%)		AAPC (95% CI)	
	2015	2021	2015-2021	p-value	2015	2021	2015-2021	p-value	2015	2021	2015-2021	p-value
Brazil												
Jumping	4·22	4·06	-1·76 (-5·76, 2·42)	0·32	NA	NA	NA	NA	NA	NA	NA	NA
Chile												
Firearms	NA	NA	NA	NA	6·03	3·63	-8·80 (-14·35, -2·87)	0·013	NA	NA	NA	NA
Hanging	83·72	88·80	1·05 (0·07, 2·05)	0·040	86·09	89·90	0·88 (0·09, 1·67)	0·035	74·60	84·21	1·82 (-0·40, 4·09)	0·090
Poisoning	5·61	3·76	-7·29 (-18·88, 5·95)	0·20	NA	NA	NA	NA	13·76	10·20	-6·46 (-21·89, 12·01)	0·38
Colombia												
Hanging	56·18	64·10	2·12 (0·27, 4·00)	0·032	58·19	64·91	1·69 (0·02, 3·39)	0·048	NA	NA	NA	NA
Costa Rica												
Jumping	3·49	1·83	-7·33 (-17·93, 4·64)	0·17	3·42	1·56	-8·32 (-18·87, 3·60)	0·13	NA	NA	NA	NA
Ecuador												
Jumping	NA	NA	NA	NA	1·12	1·00	-2·59 (-20·90, 19·96)	0·76	NA	NA	NA	NA
Paraguay												
Jumping	0·79	1·39	1·08 (-23·53, 33·61)	0·92	NA	NA	NA	NA	1·06	1·12	-1·69 (-35·16, 49·06)	0·92
Poisoning	5·24	3·83	-2·88 (-18·19, 15·30)	0·68	NA	NA	NA	NA	11·70	8·43	-0·13 (-21·45, 26·97)	0·99
United States of America												
Firearms	49·88	54·68	1·22 (0·17, 2·28)	0·030	55·68	59·84	0·91 (-0·08, 1·91)	0·065	NA	NA	NA	NA
Hanging	26·85	25·82	0·02 (-2·20, 2·28)	0·98	26·90	25·15	-0·47 (-2·73, 1·85)	0·62	NA	NA	NA	NA
Uruguay												
Jumping	NA	NA	NA	NA	1·57	1·57	5·03 (-12·10, 25·50)	0·51	NA	NA	NA	NA

AAPC Average annual percent change; --- Zeros present in the time-series (AAPC not estimated); NA Not applicable

Note: AAPCs represent model-based estimates of temporal change derived from the fitted log-linear joinpoint regression using all annual observations over the specified interval and therefore may differ from changes calculated directly from the observed values at the beginning and end of the interval.

Figure S1. Residual plots for suicide mortality rate models for (A) the Andean Area, (B) Central America, Mexico and Latin Caribbean, (C) Non-Latin Caribbean, (D) North America, (E) Southern Cone and Brazil and (F) the Region of the Americas

(A)

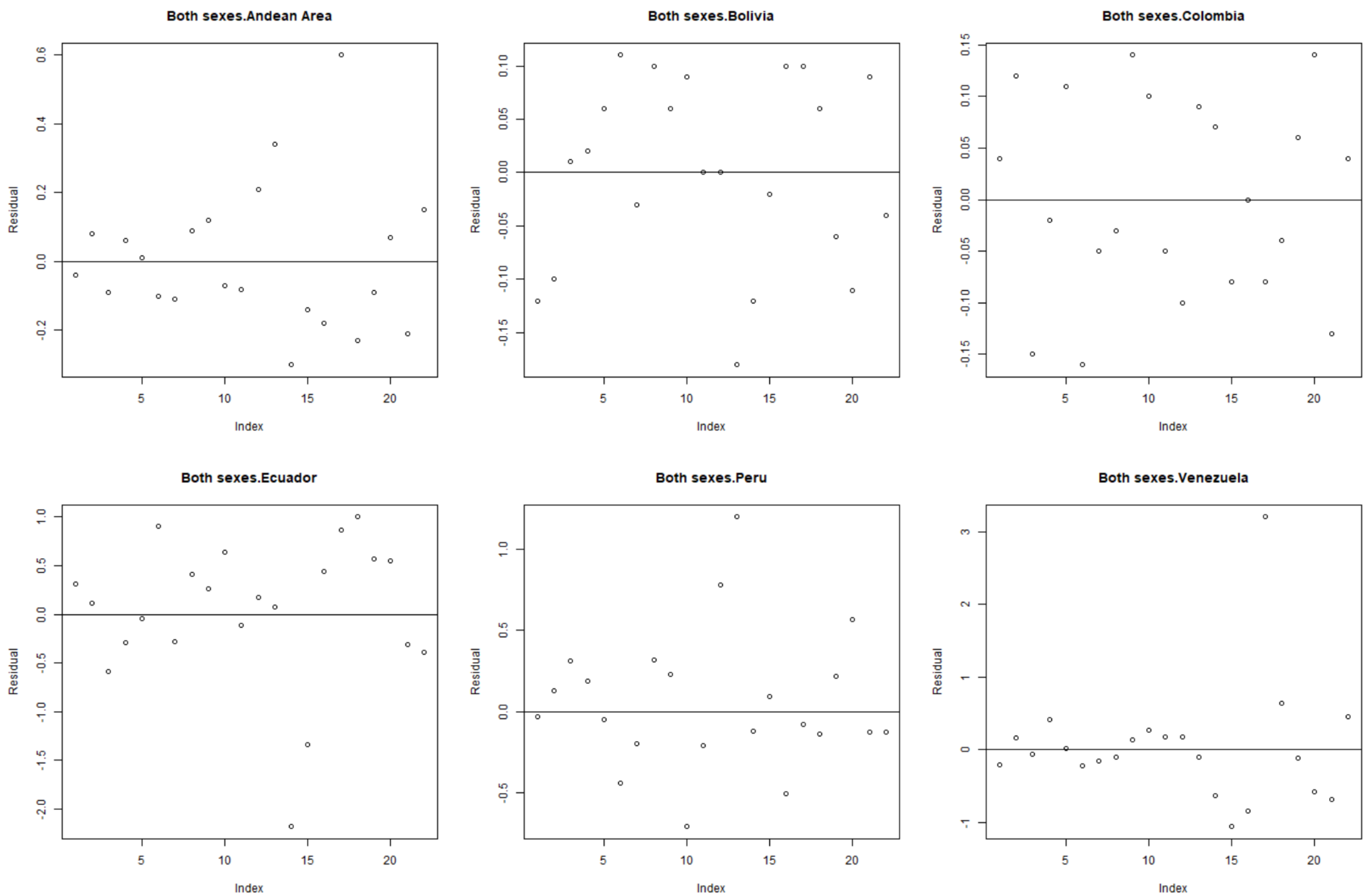

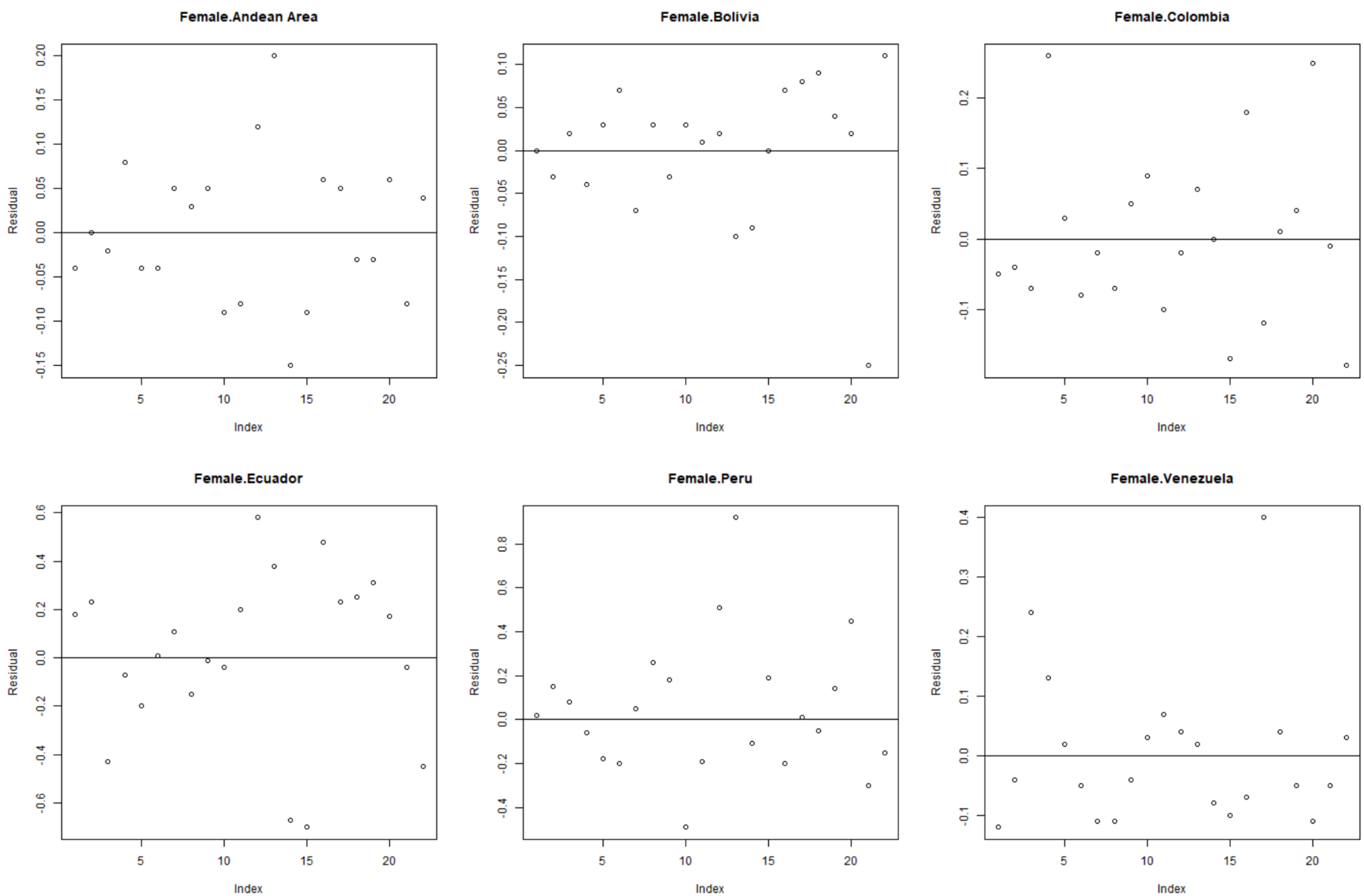
Female.Andean Area
Female.Bolivia
Female.Colombia
Female.Ecuador
Female.Peru
Female.Venezuela
Residual
Index

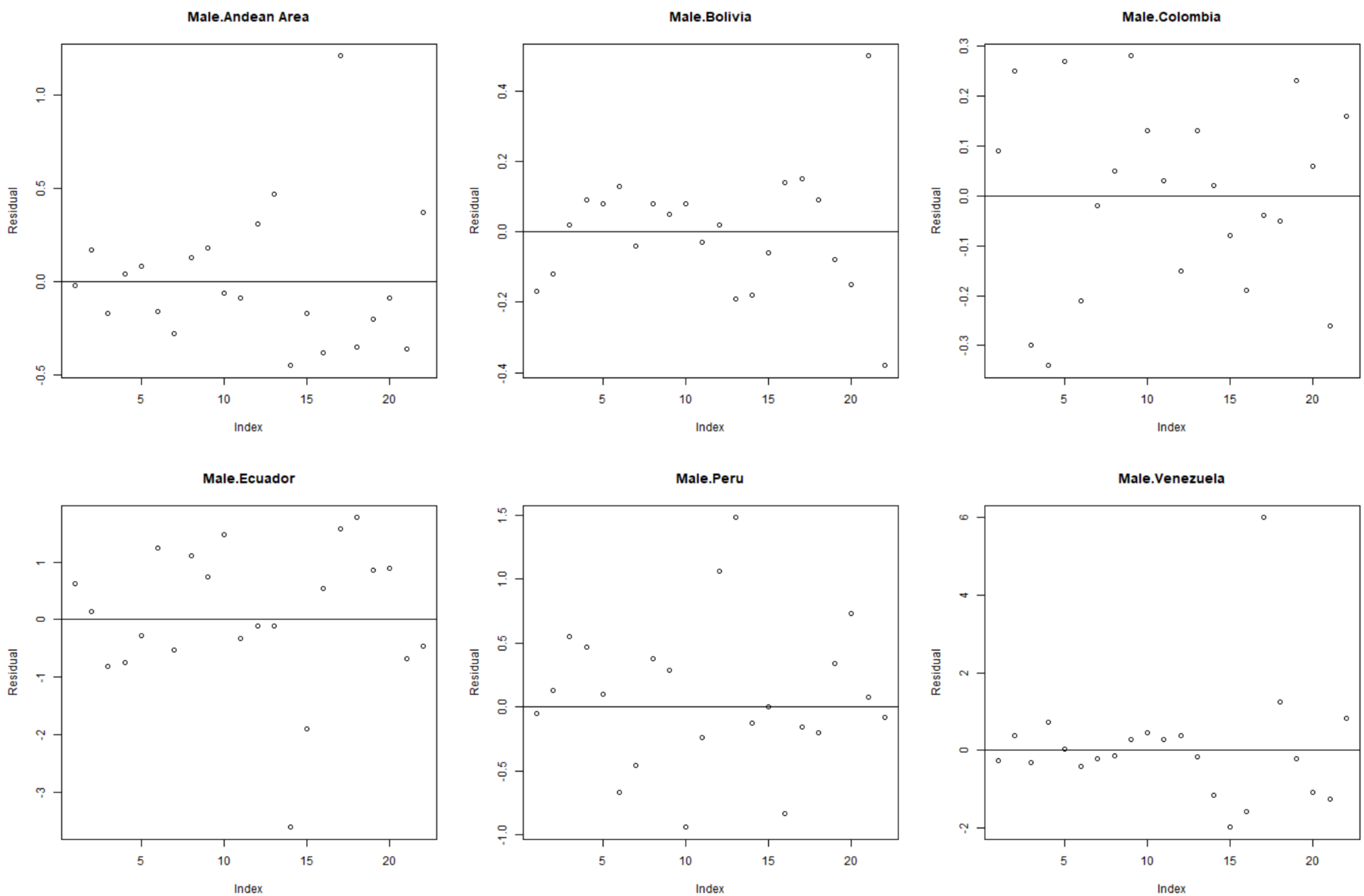
Male.Andean Area
Male.Bolivia
Male.Colombia
Male.Ecuador
Male.Peru
Male.Venezuela
Residual
Index

(B)

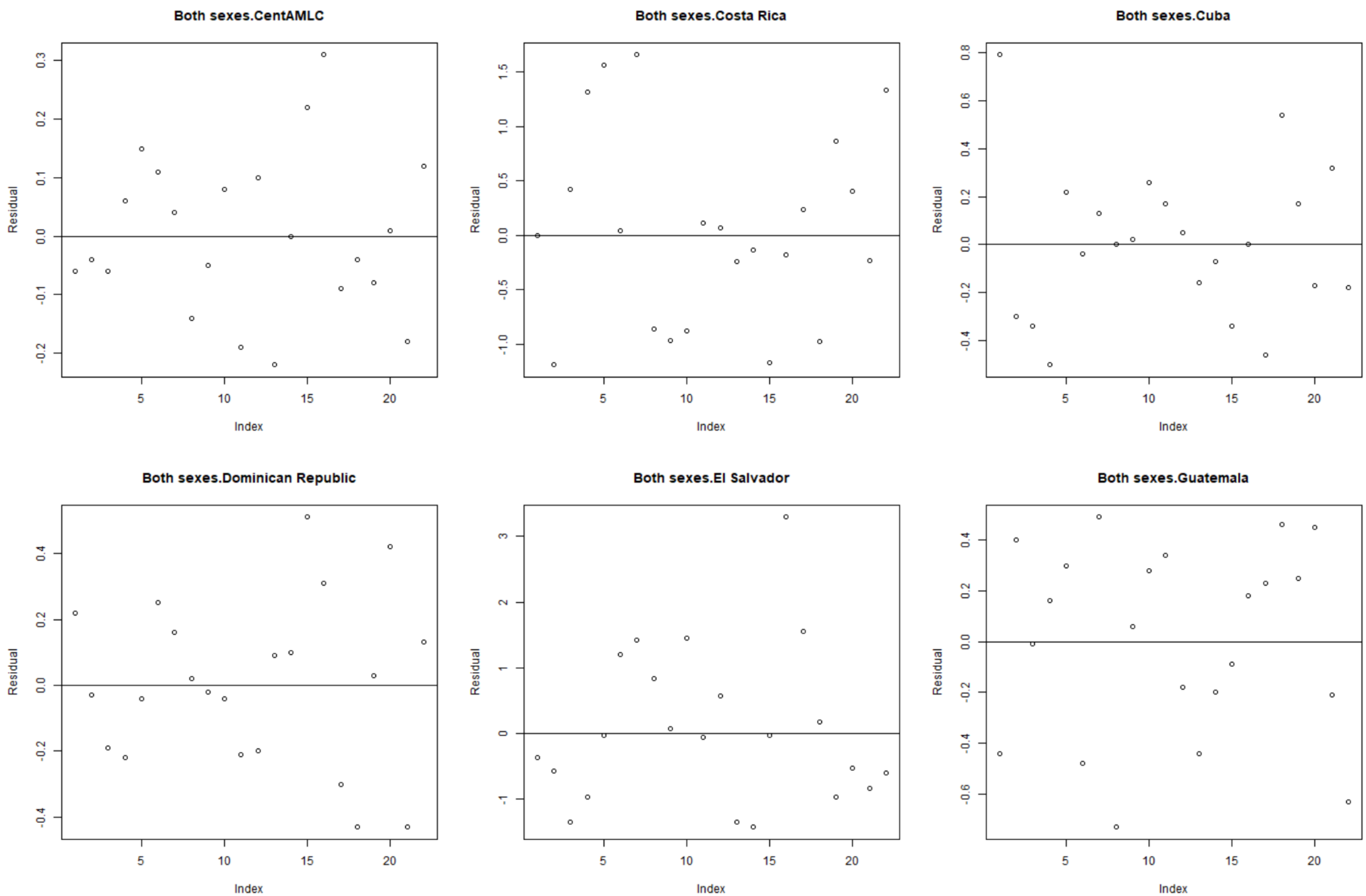

Both sexes.CentAMLC
Both sexes.Costa Rica
Both sexes.Cuba
Both sexes.Dominican Republic
Both sexes.El Salvador
Both sexes.Guatemala
Residual
Index

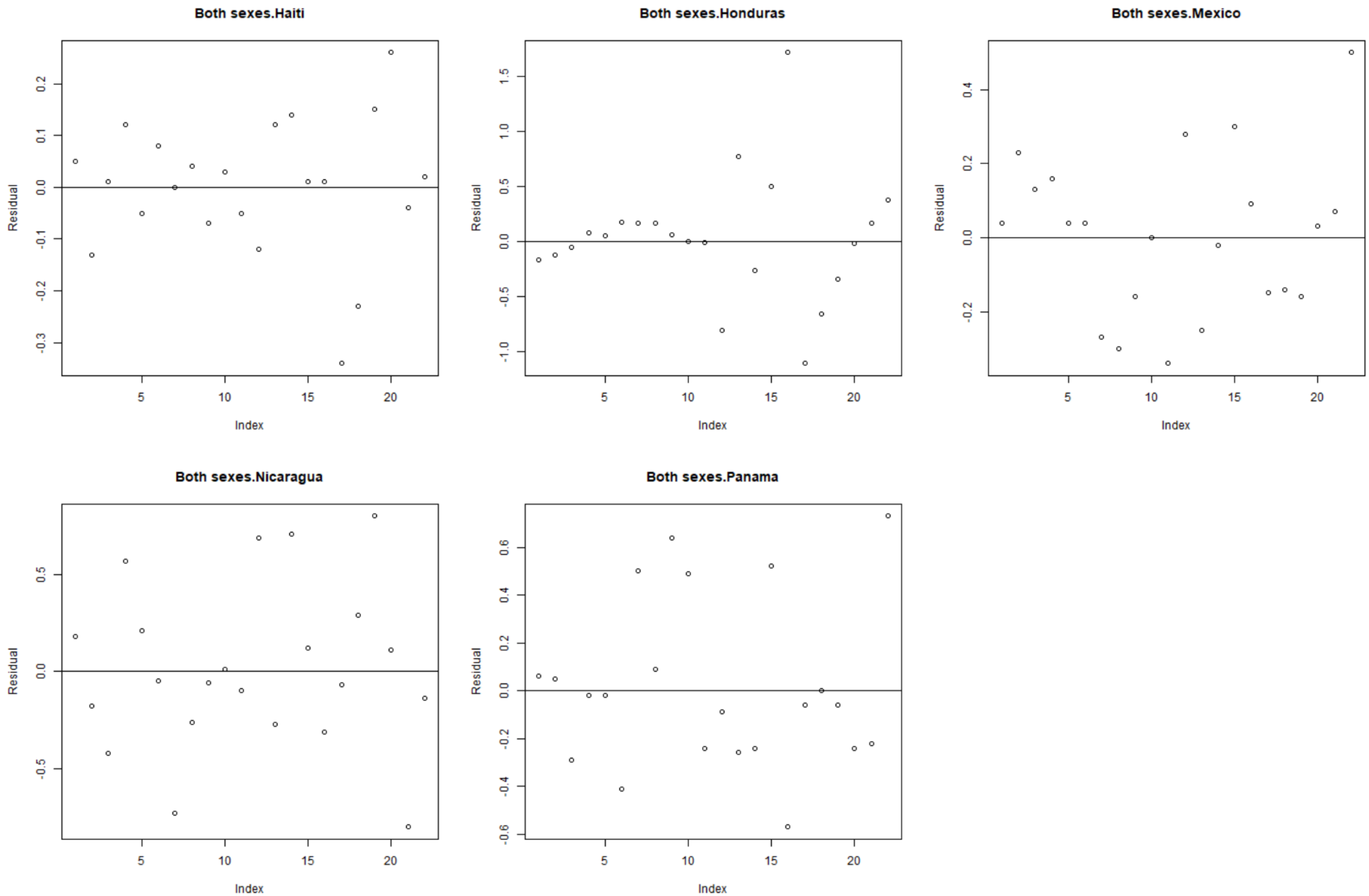
Both sexes.Haiti
Residual
Index
Both sexes.Honduras
Residual
Index
Both sexes.Mexico
Residual
Index
Both sexes.Nicaragua
Residual
Index
Both sexes.Panama
Residual
Index

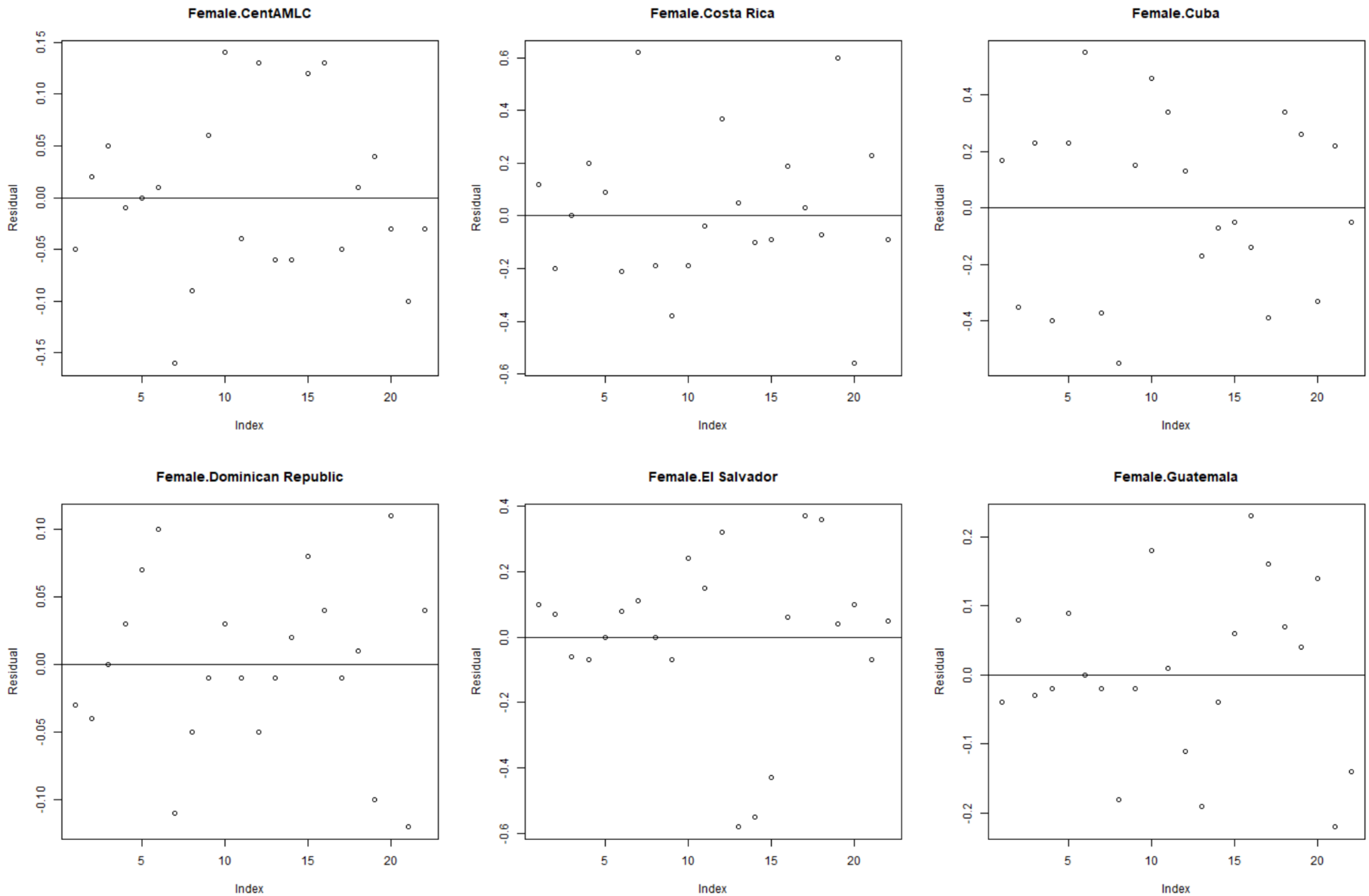
Female.CentAMLC
Female.Costa Rica
Female.Cuba
Female.Dominican Republic
Female.El Salvador
Female.Guatemala
Residual
Index

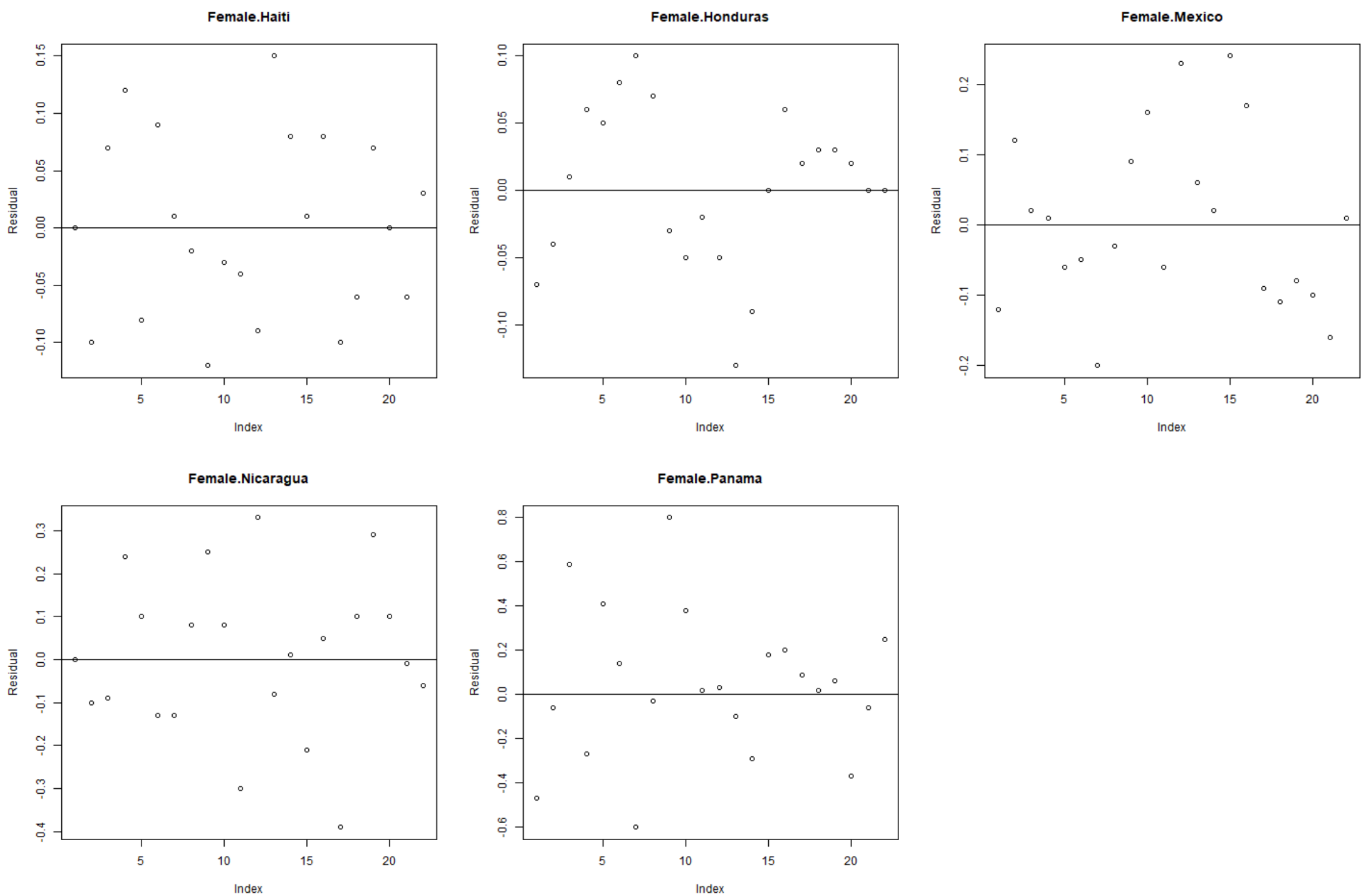
Female.Haiti
Female.Honduras
Female.Mexico
Female.Nicaragua
Female.Panama
Residual
Index

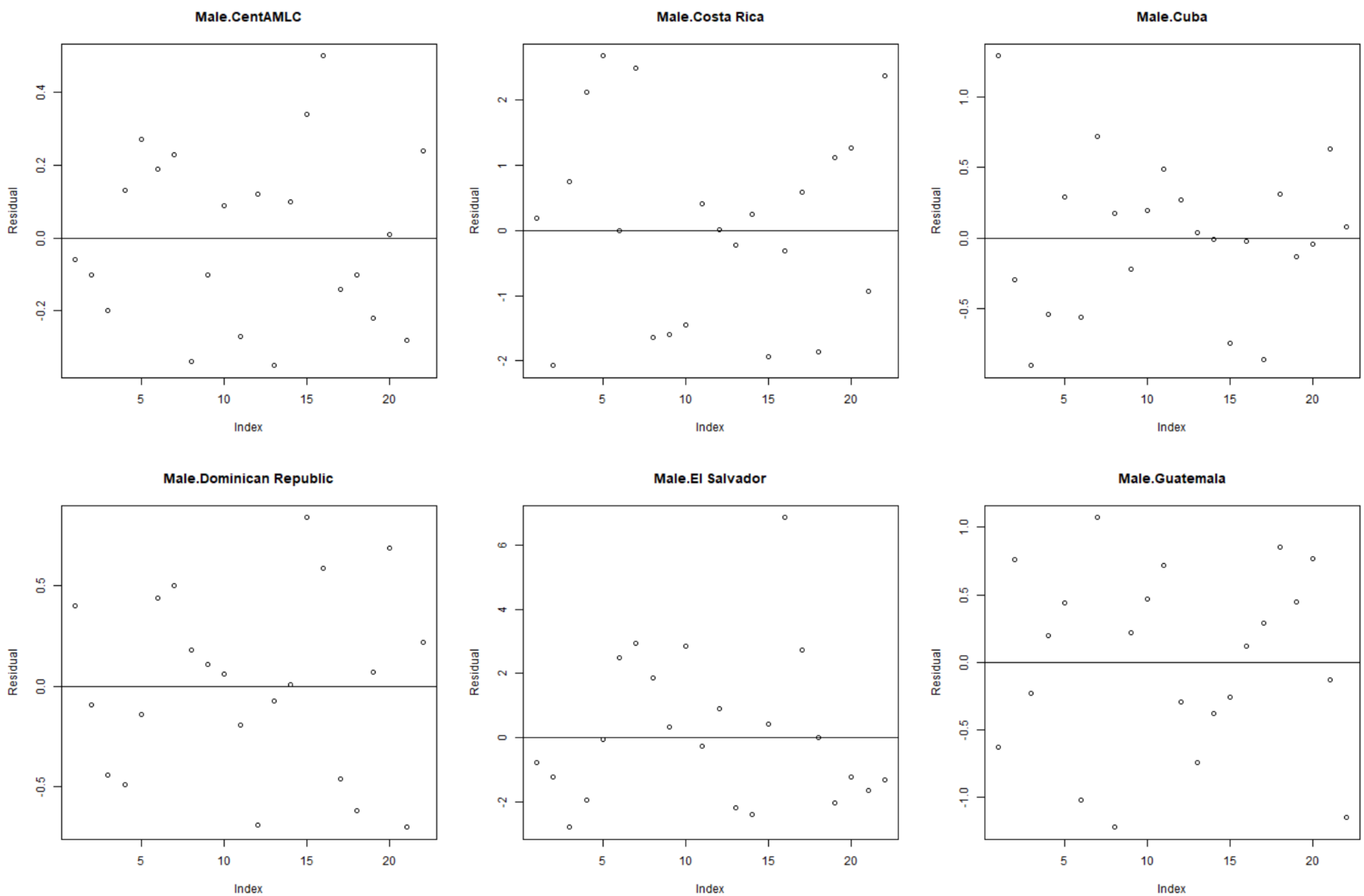
Male.CentAMLC
Male.Costa Rica
Male.Cuba
Male.Dominican Republic
Male.El Salvador
Male.Guatemala
Residual
Index
5
10
15
20

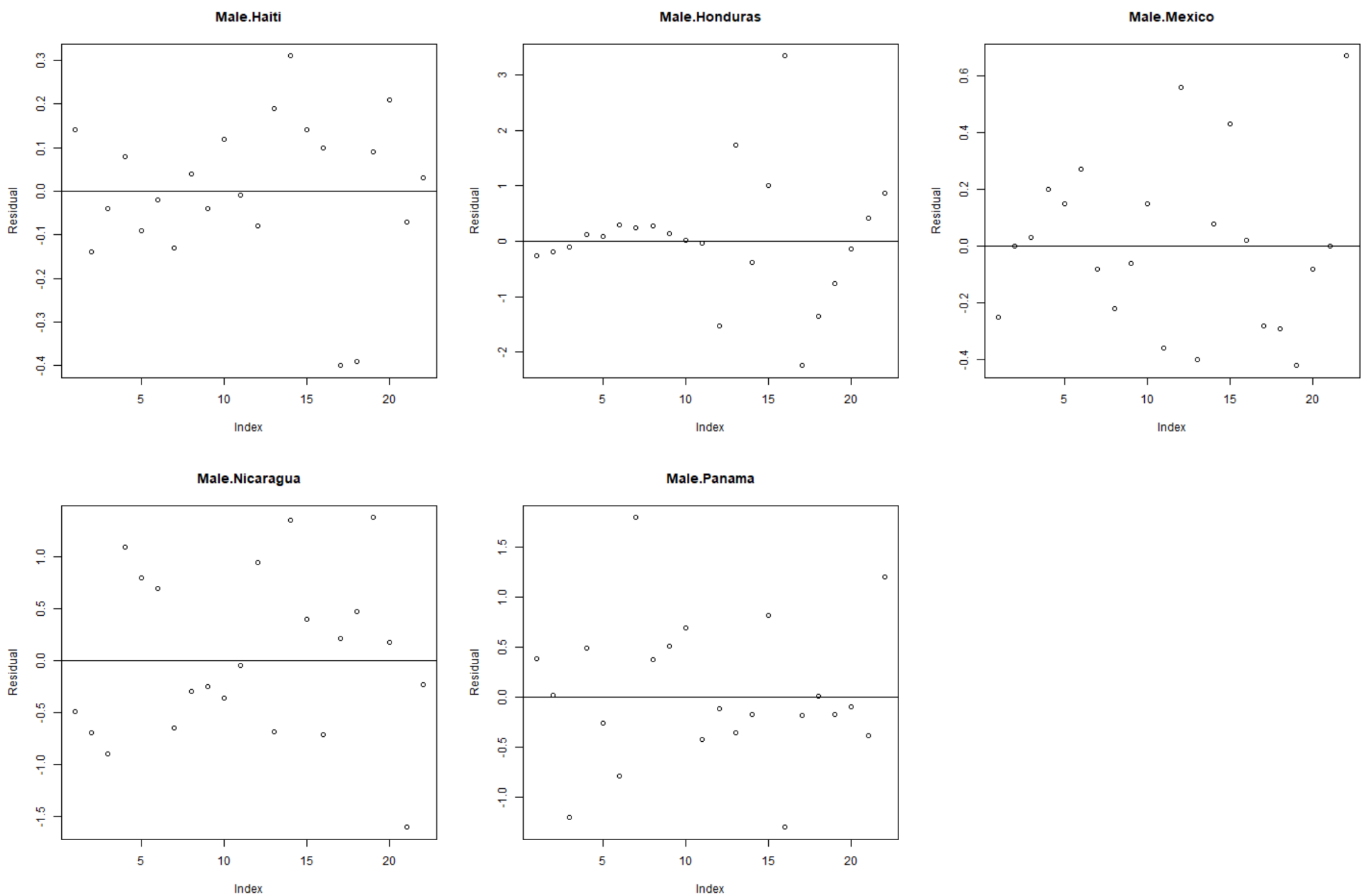
Male.Haiti
Male.Honduras
Male.Mexico
Male.Nicaragua
Male.Panama
Residual
Index

(C)

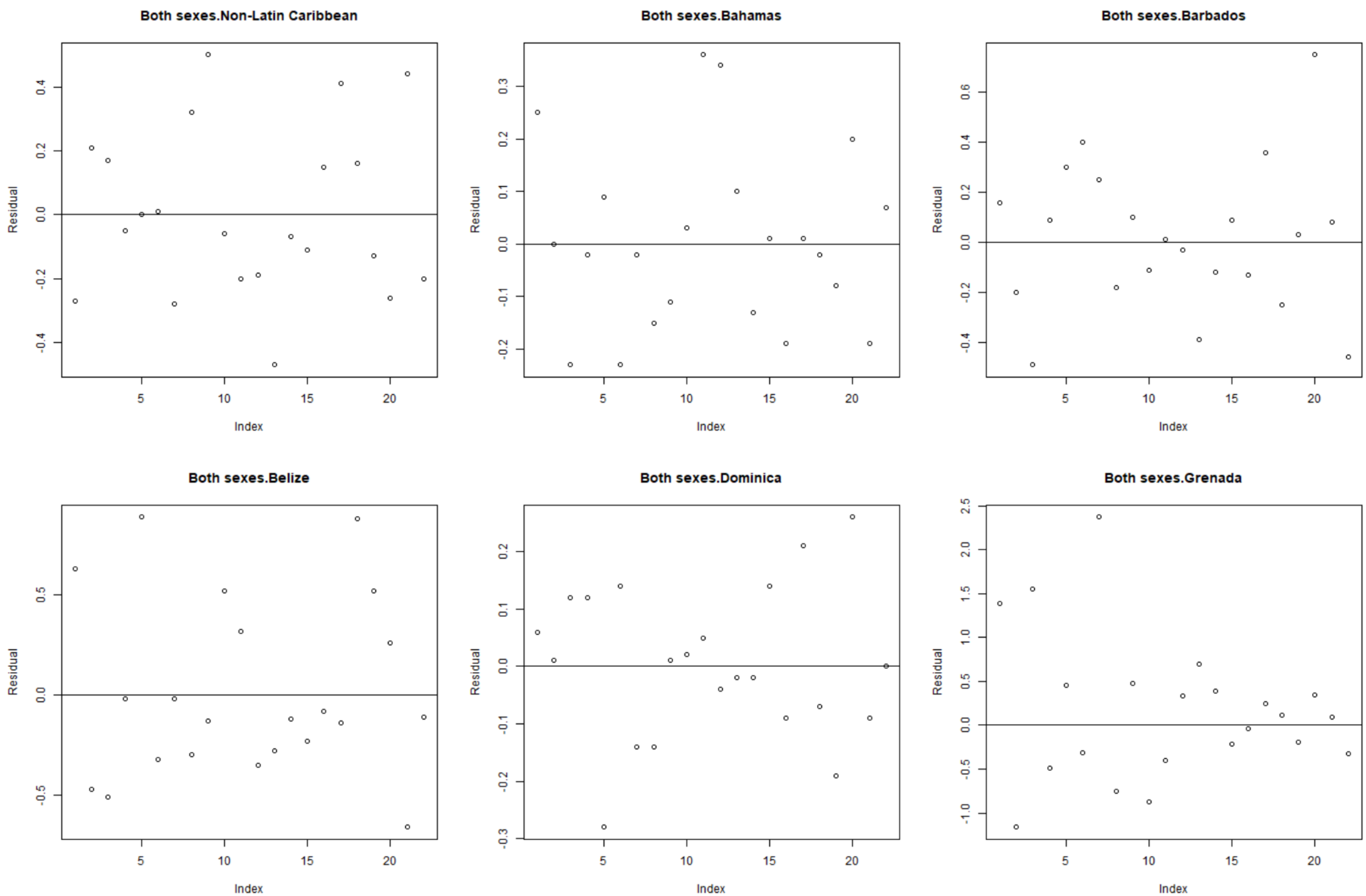
Both sexes.Non-Latin Caribbean
Both sexes.Bahamas
Both sexes.Barbados
Both sexes.Belize
Both sexes.Dominica
Both sexes.Grenada
Residual
Index

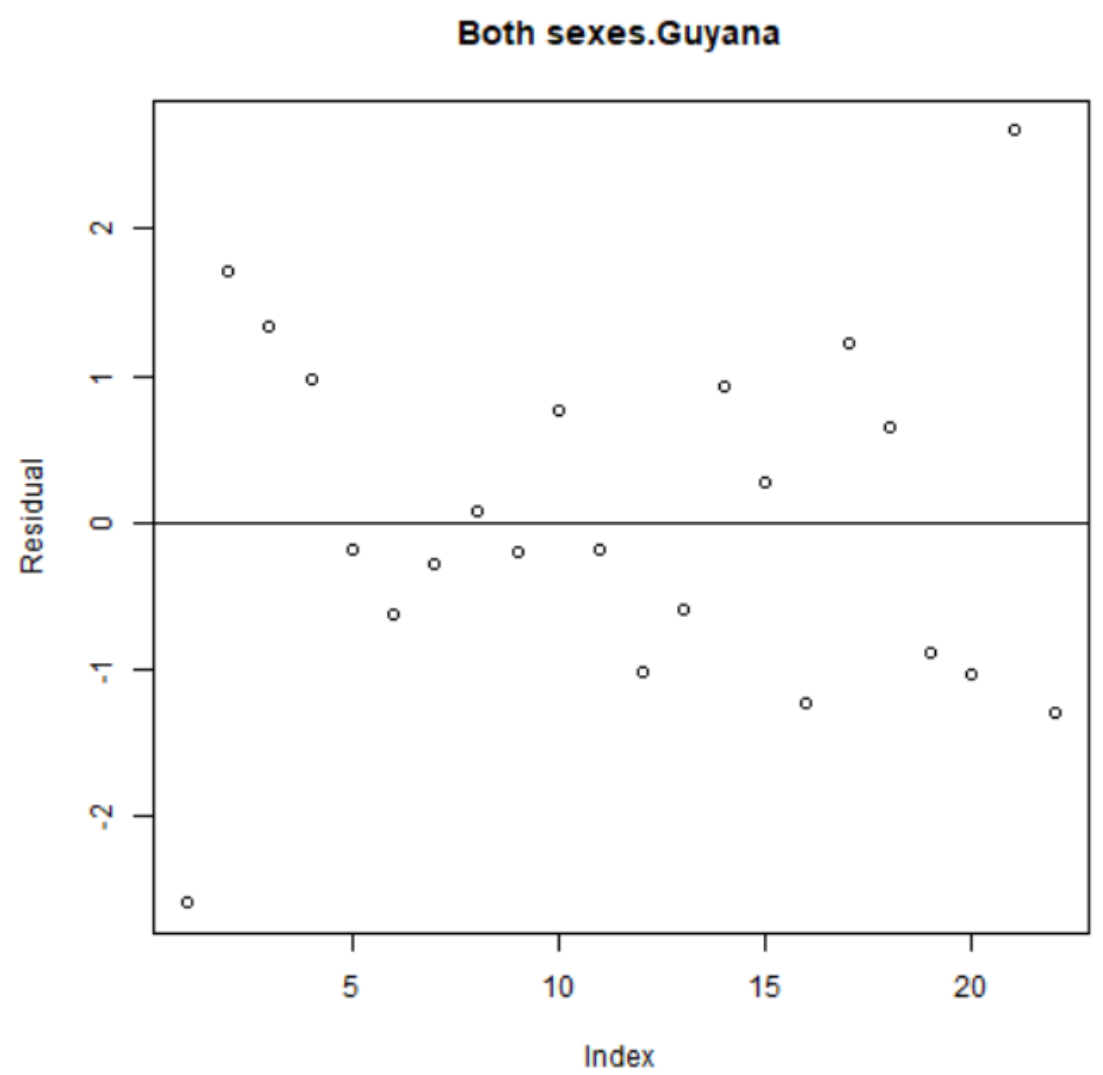
Both sexes.Guyana
Residual
-2
-1
0
1
2
Index
5
10
15
20

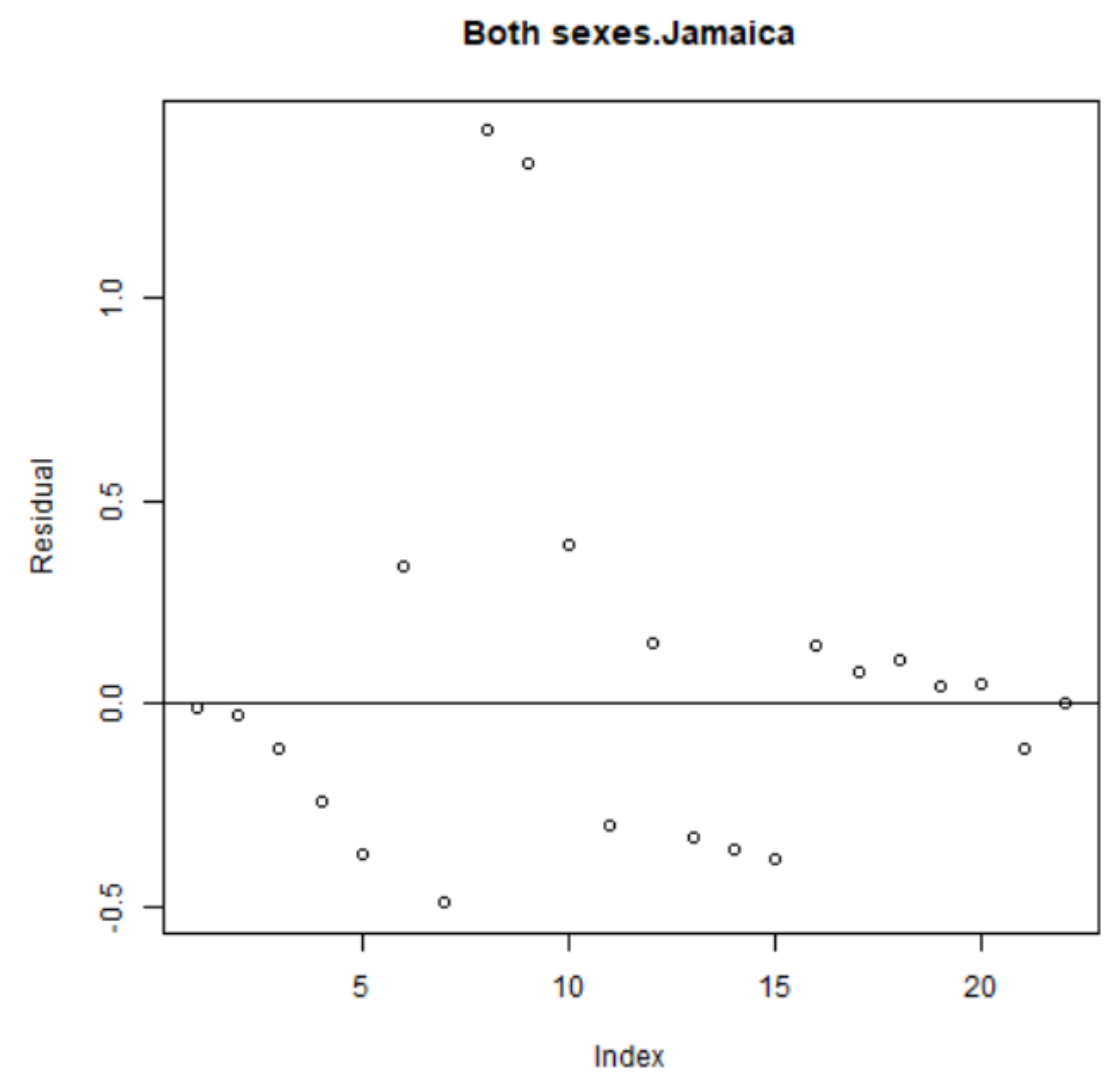
Both sexes.Jamaica
Residual
-0.5
0.0
0.5
1.0
Index
5
10
15
20

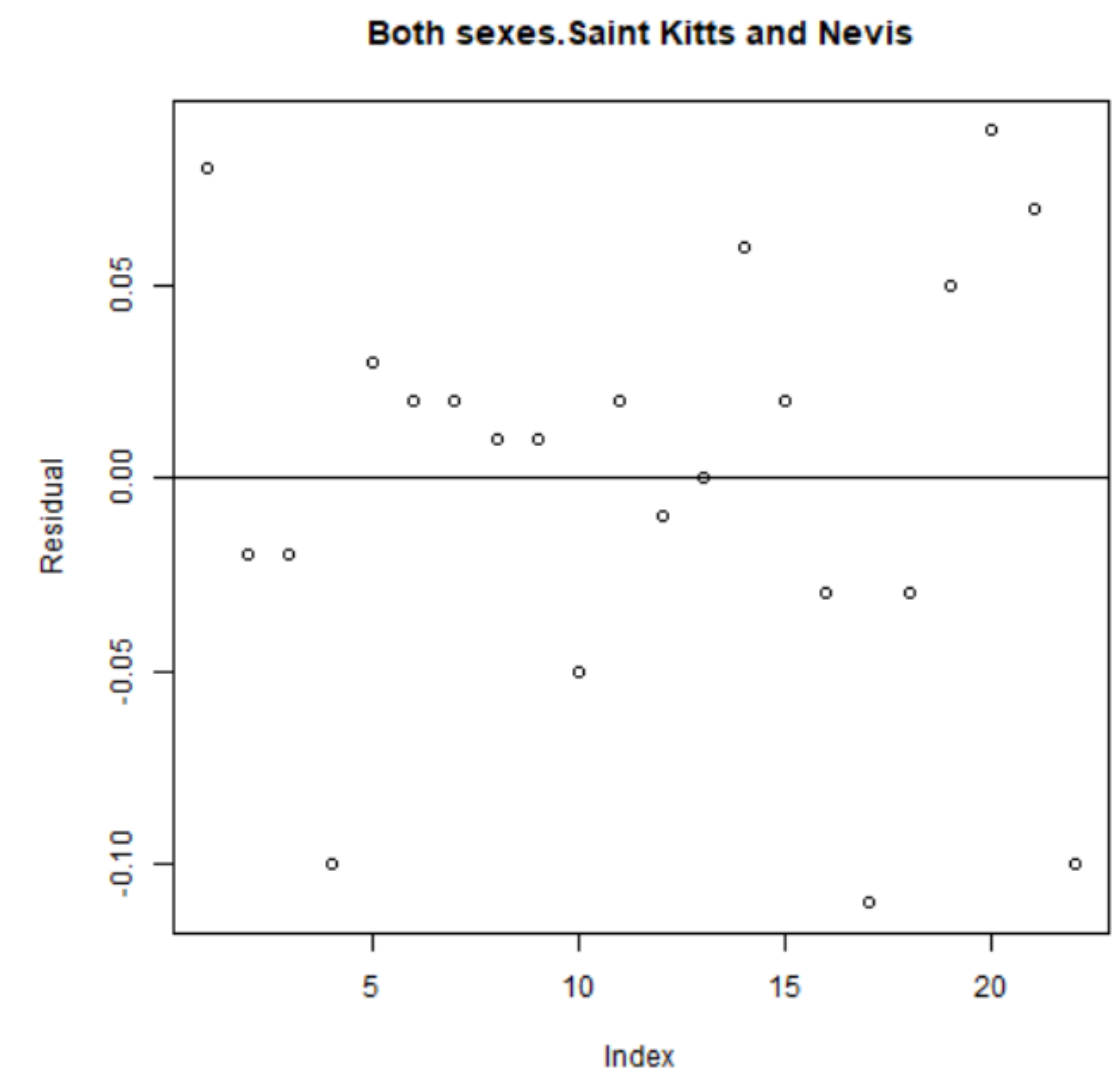
Both sexes.Saint Kitts and Nevis
Residual
-0.10
-0.05
0.00
0.05
Index
5
10
15
20

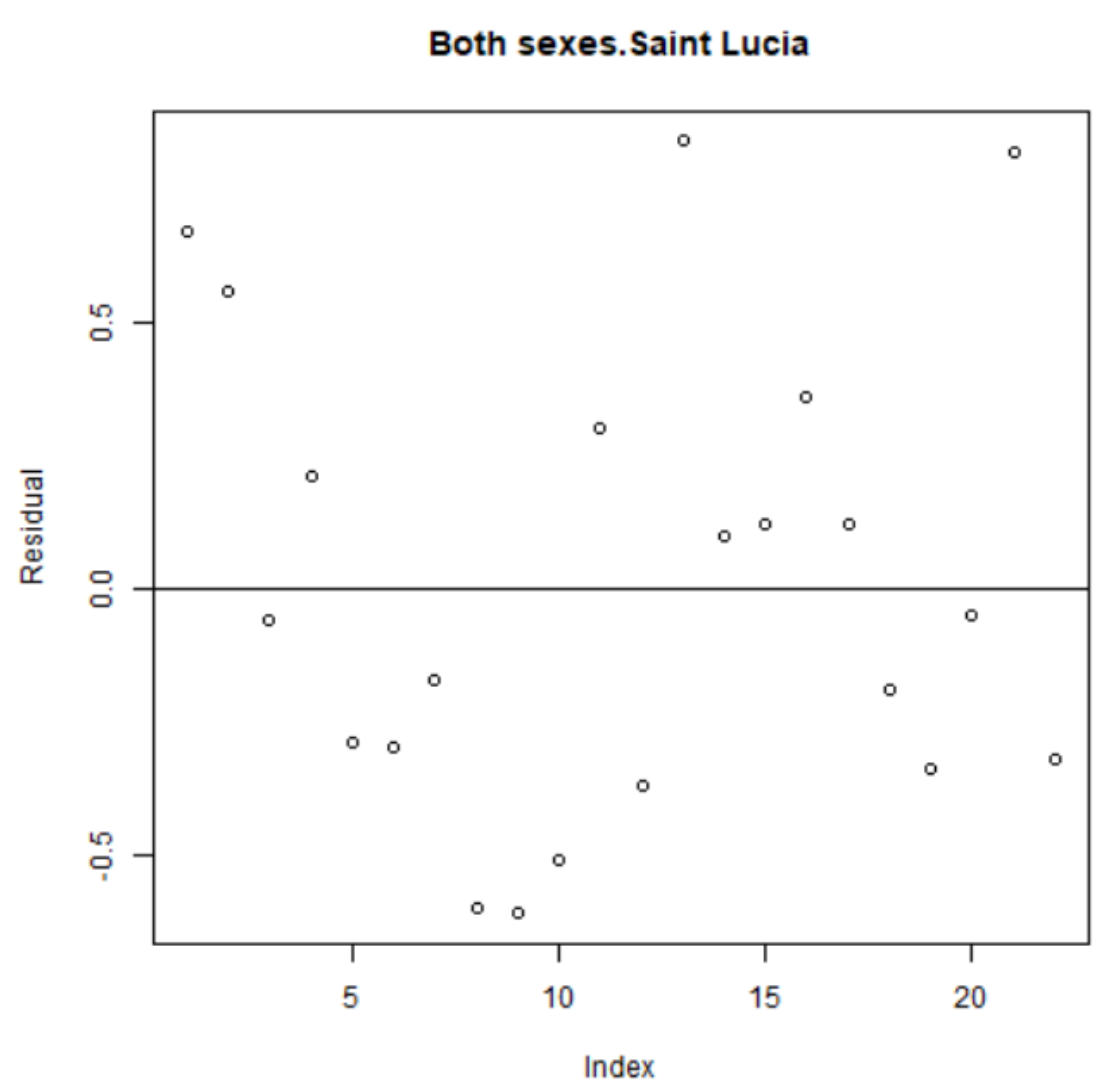
Both sexes.Saint Lucia
Residual
-0.5
0.0
0.5
Index
5
10
15
20

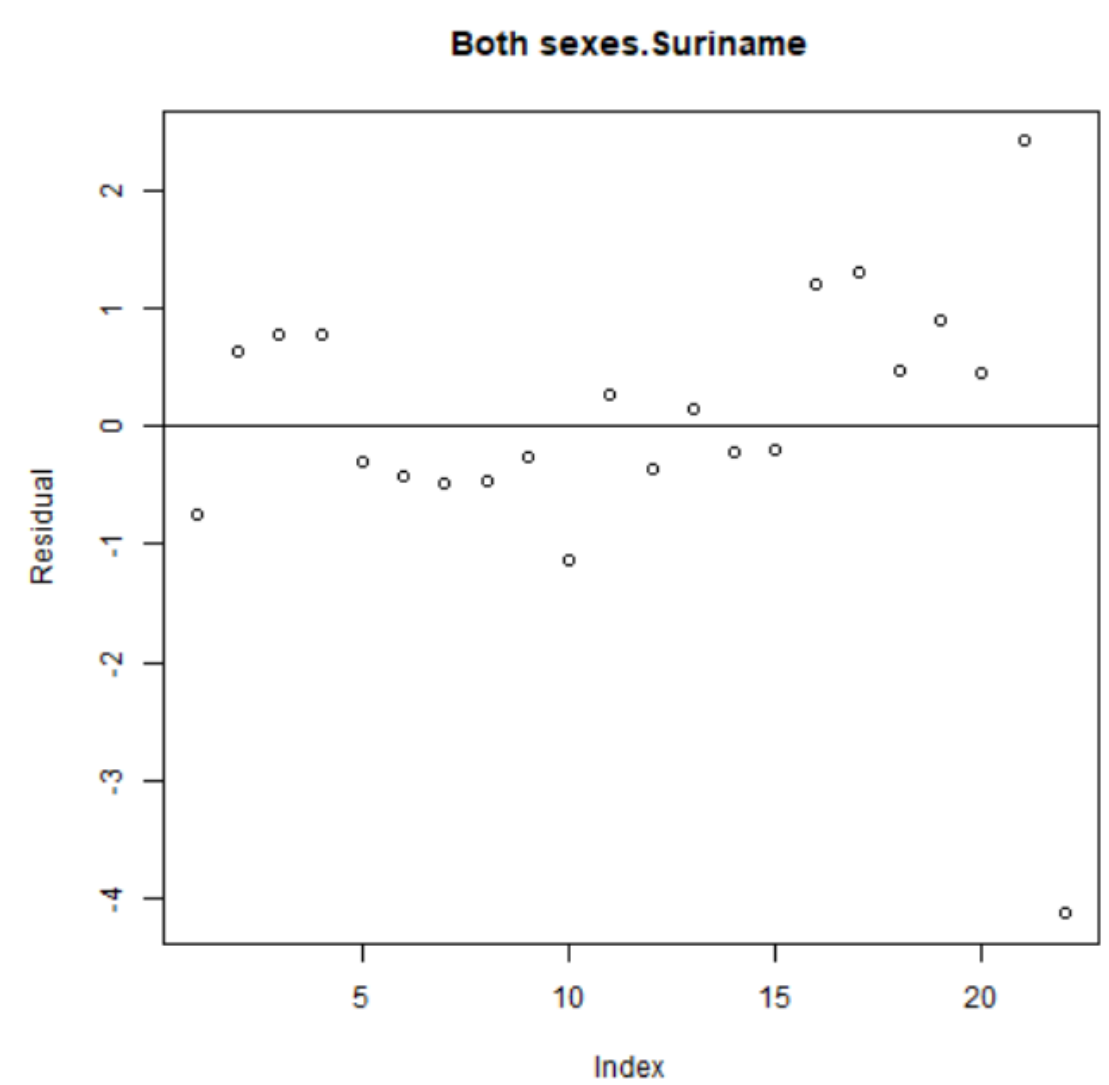
Both sexes.Suriname
Residual
-4
-3
-2
-1
0
1
2
Index
5
10
15
20

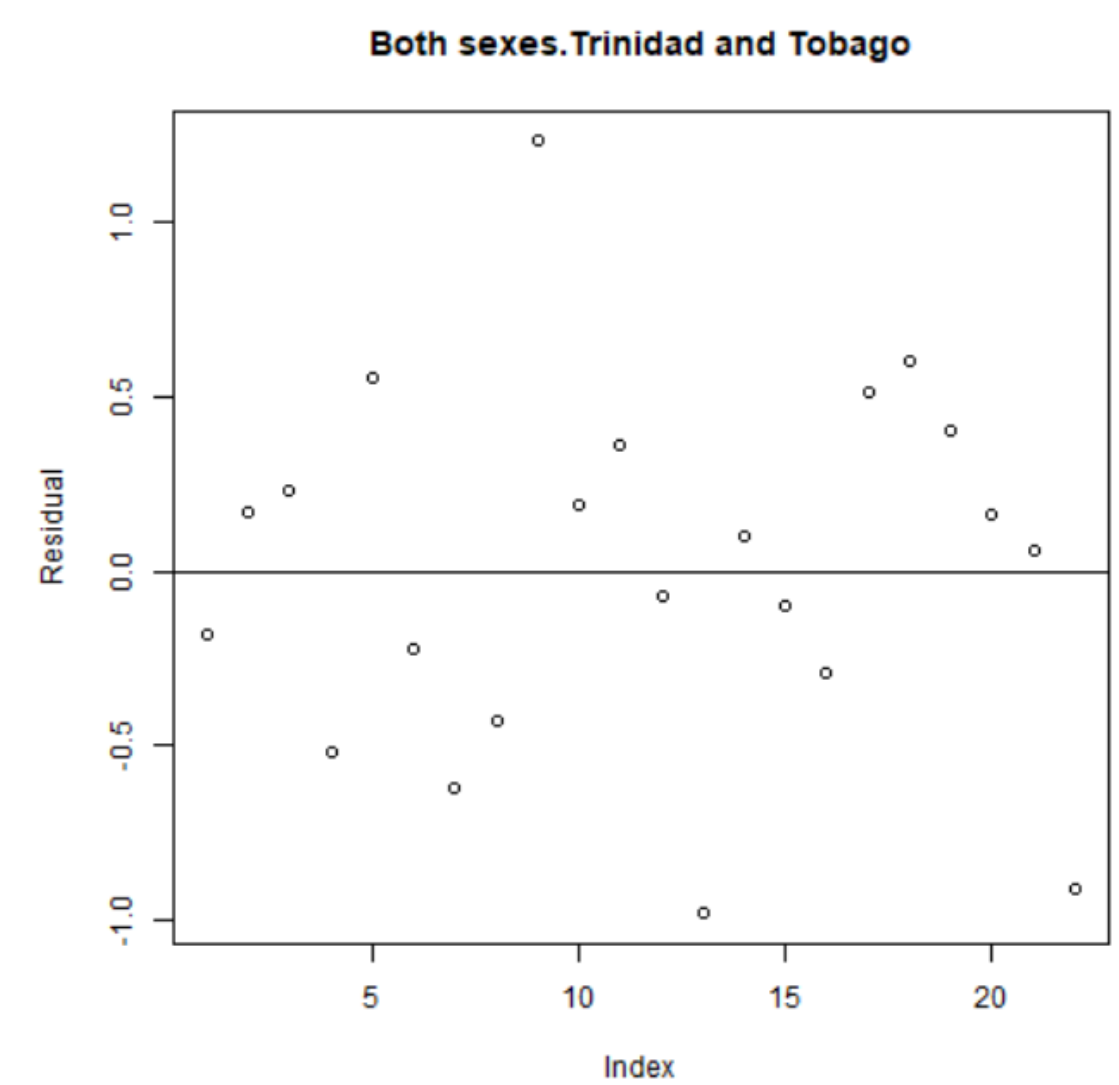
Both sexes.Trinidad and Tobago
Residual
-1.0
-0.5
0.0
0.5
1.0
Index
5
10
15
20

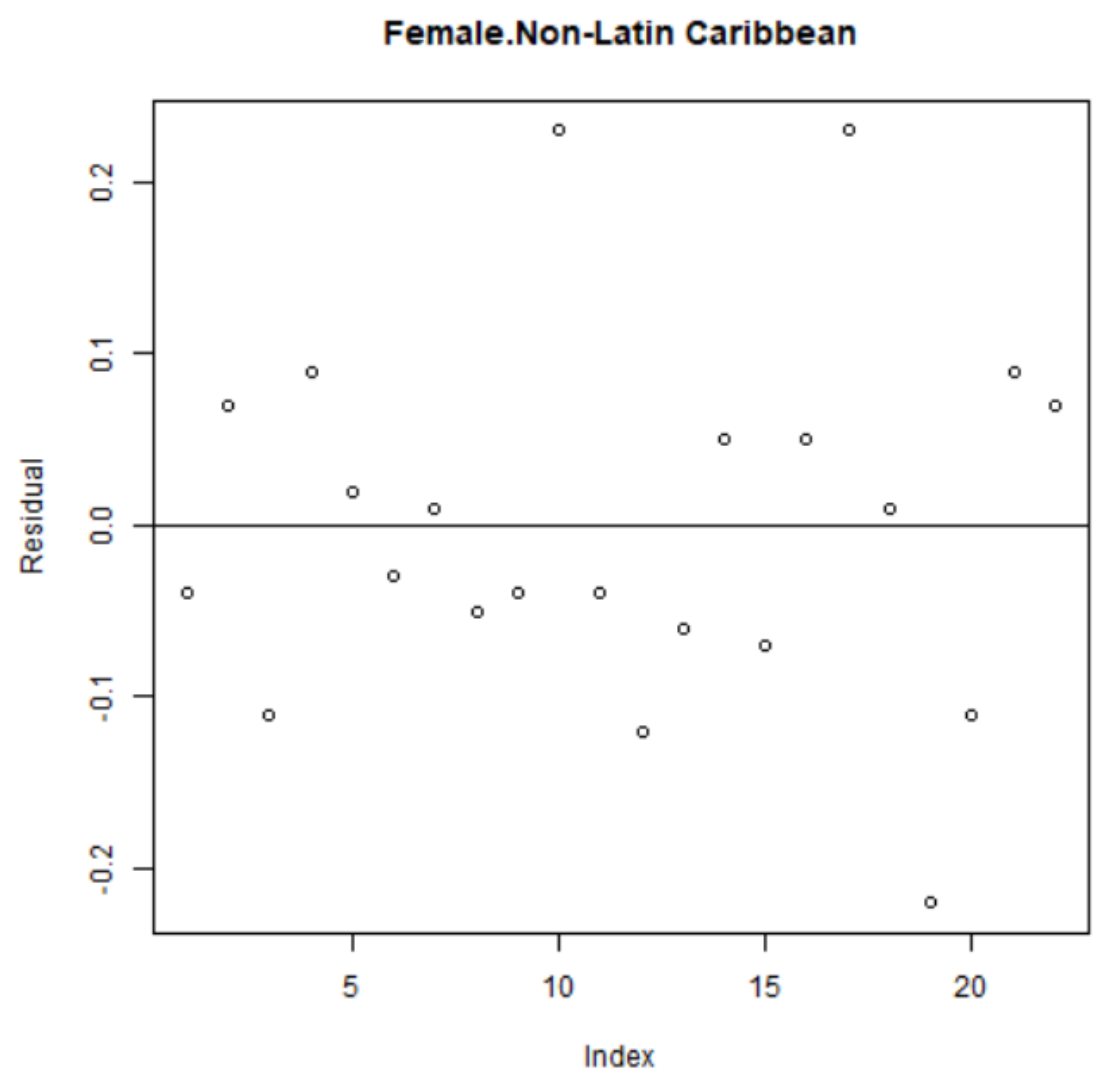
Female.Non-Latin Caribbean
Residual
Index

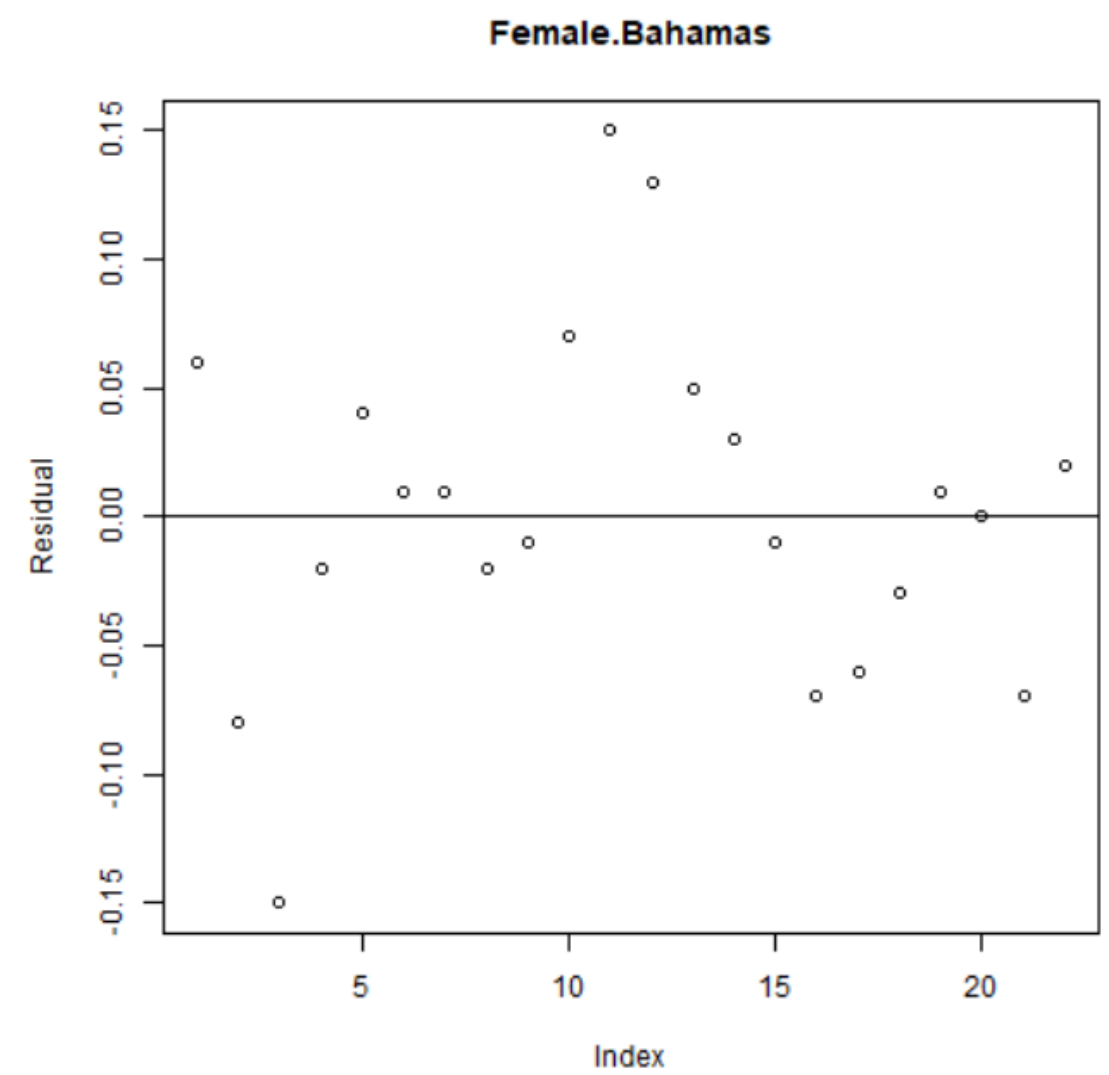
Female.Bahamas
Residual
Index

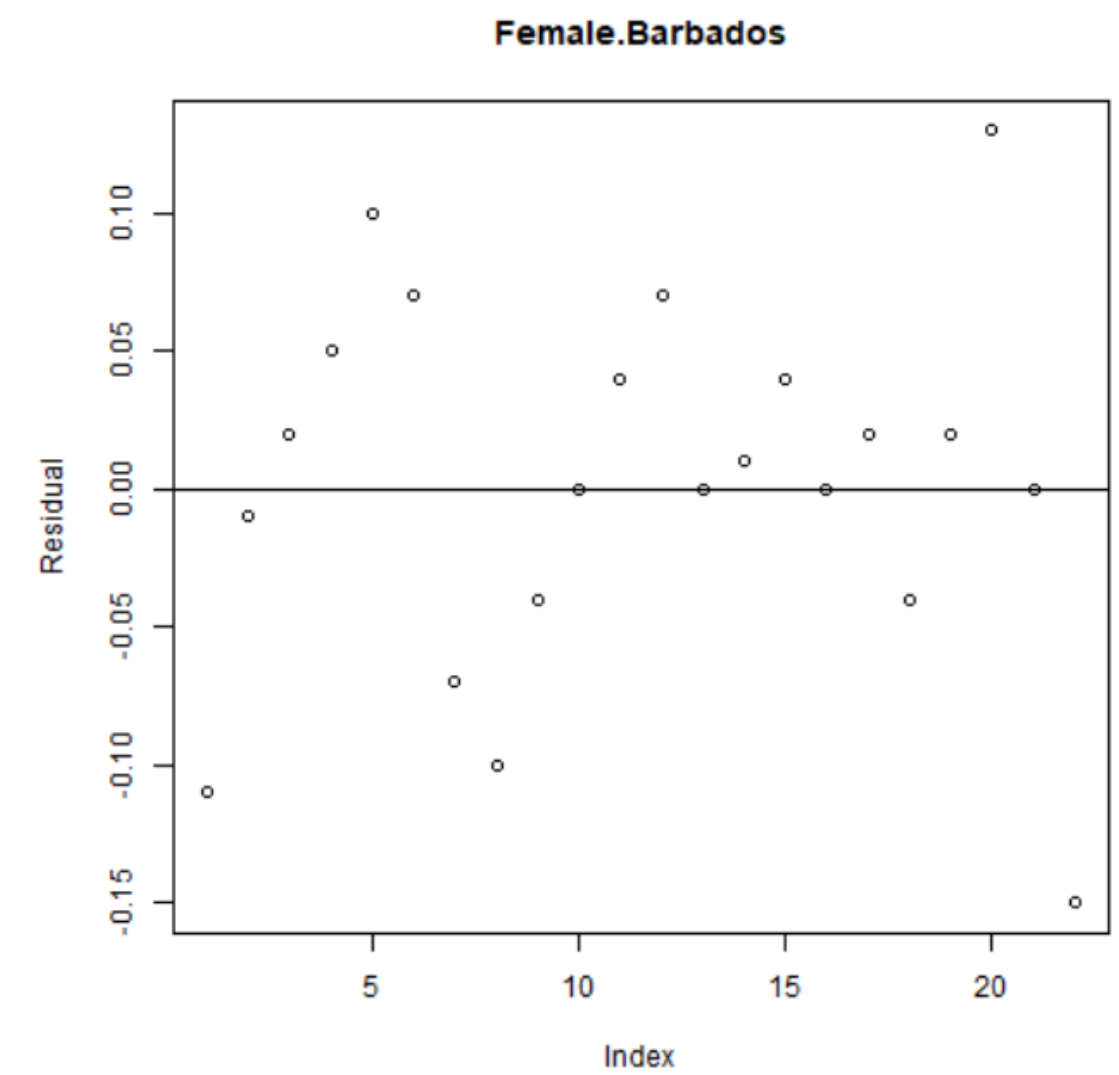
Female.Barbados
Residual
Index

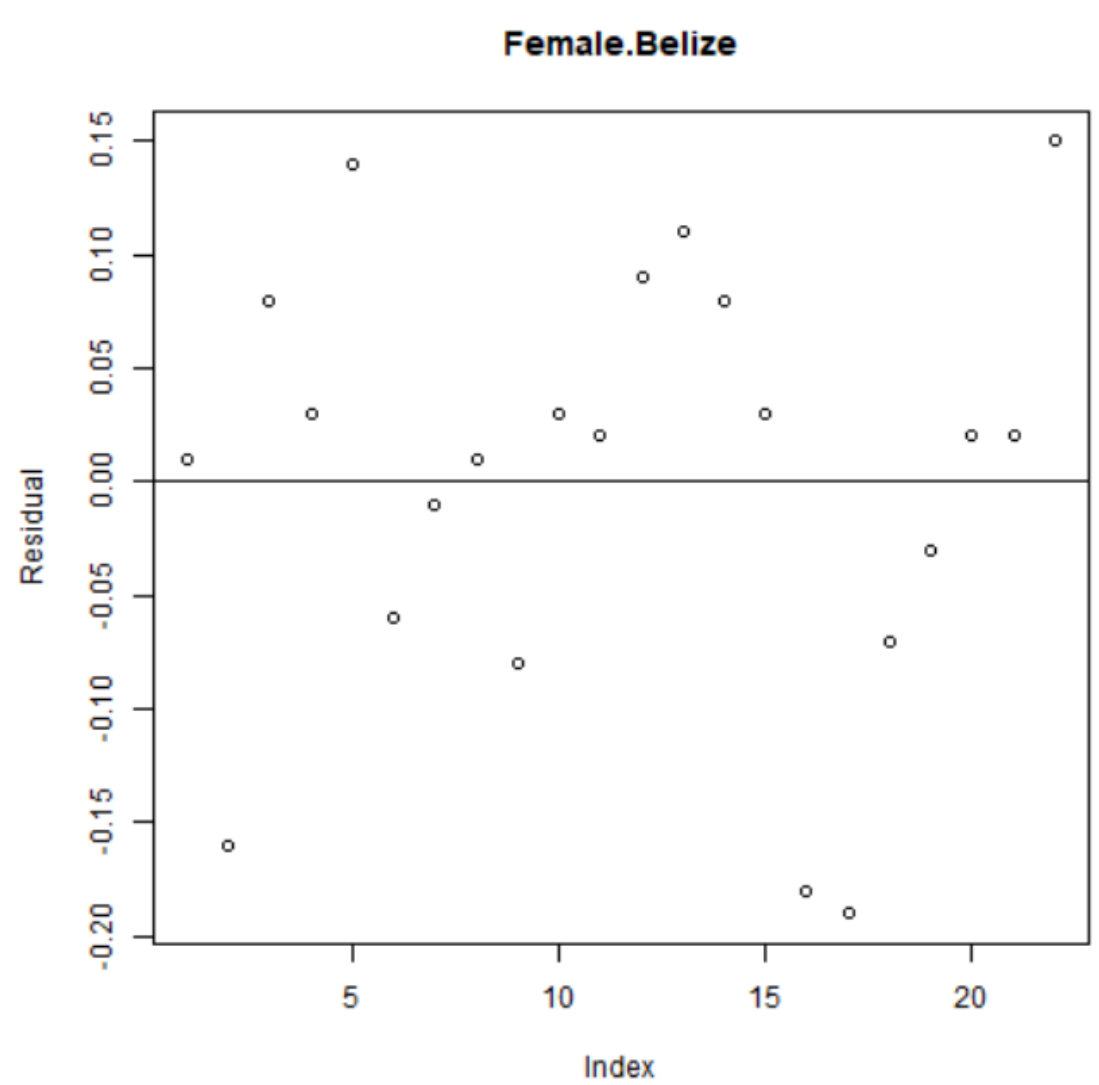
Female.Belize
Residual
Index

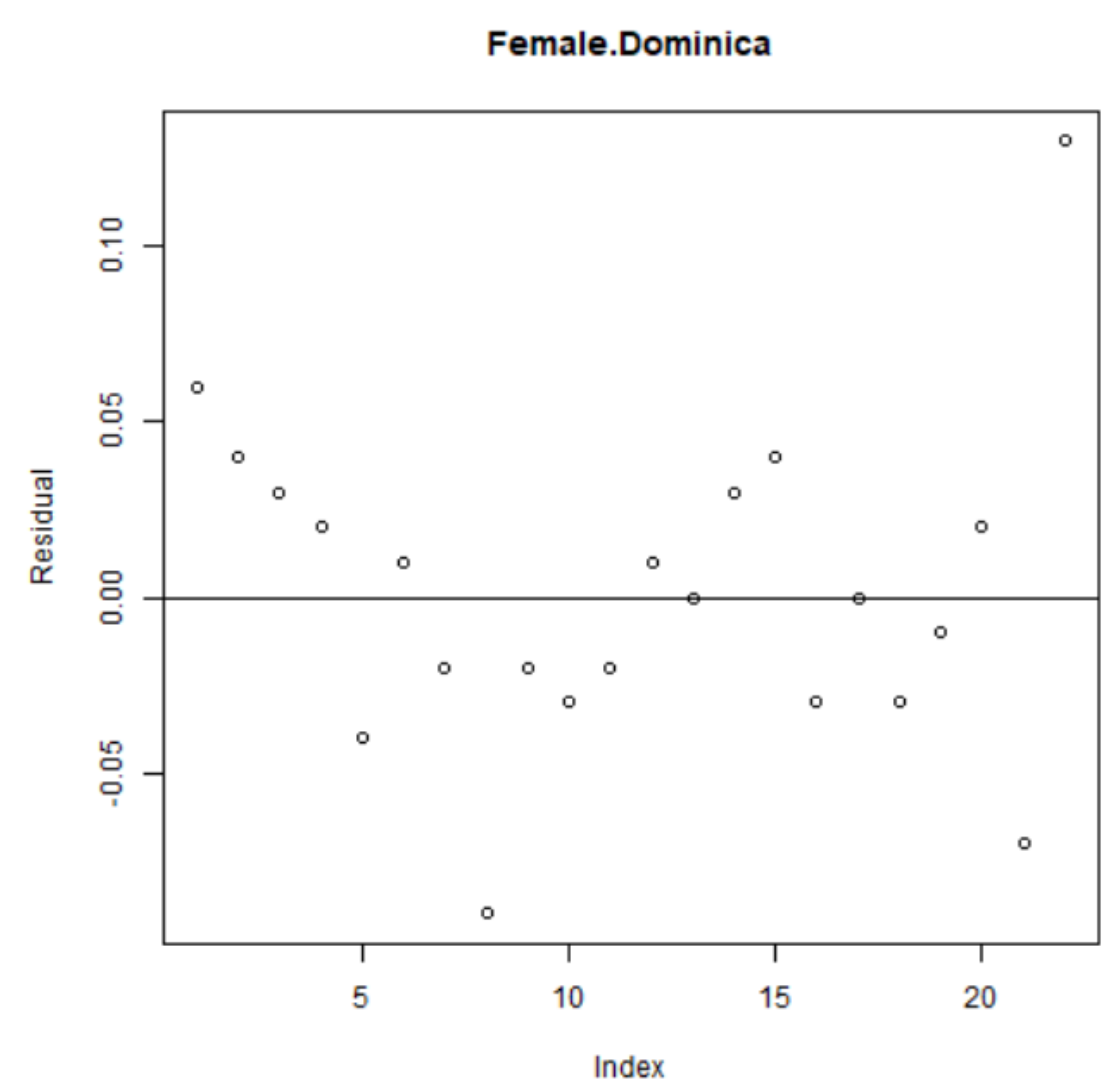
Female.Dominica
Residual
Index

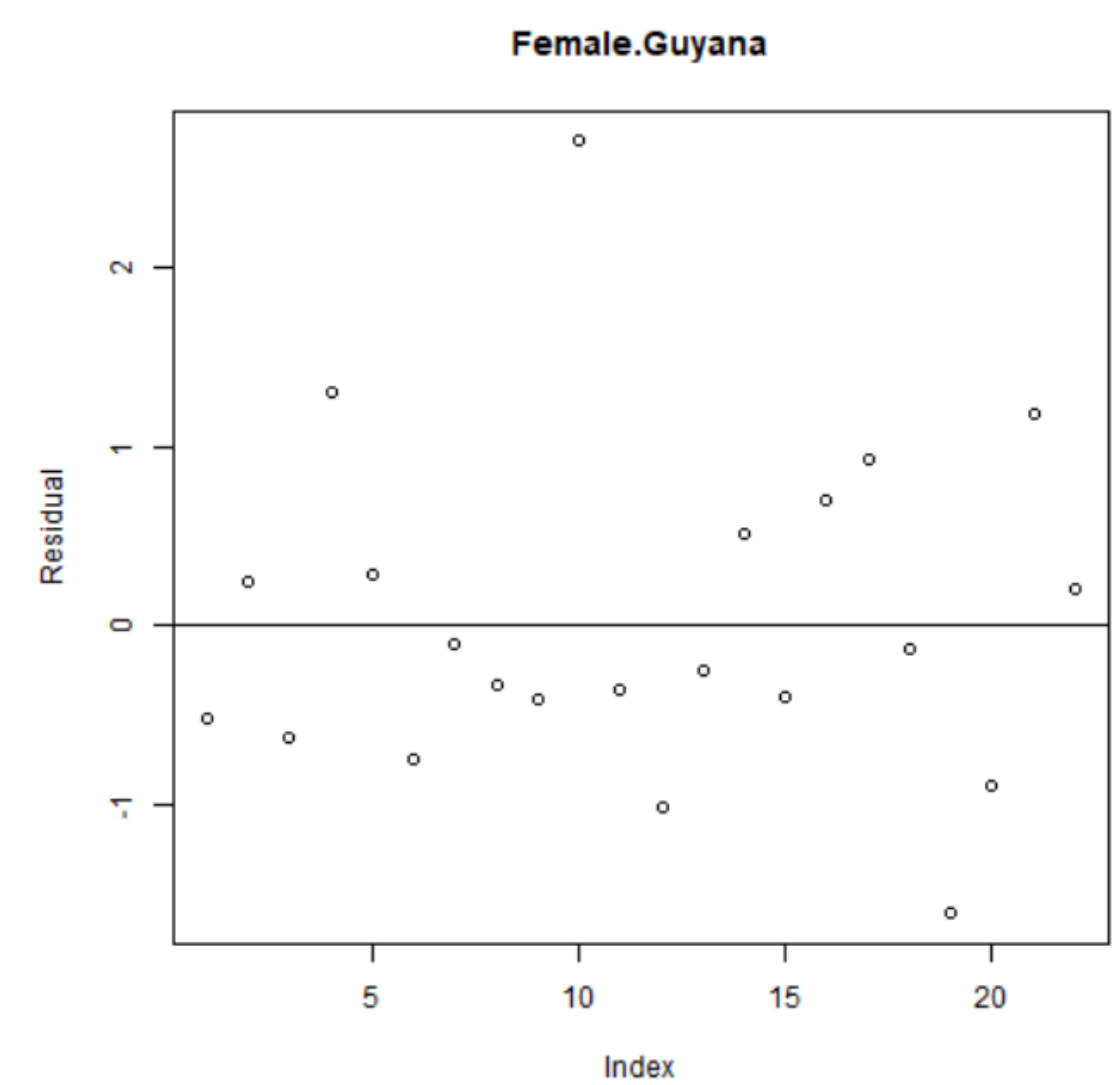
Female.Guyana
Residual
Index

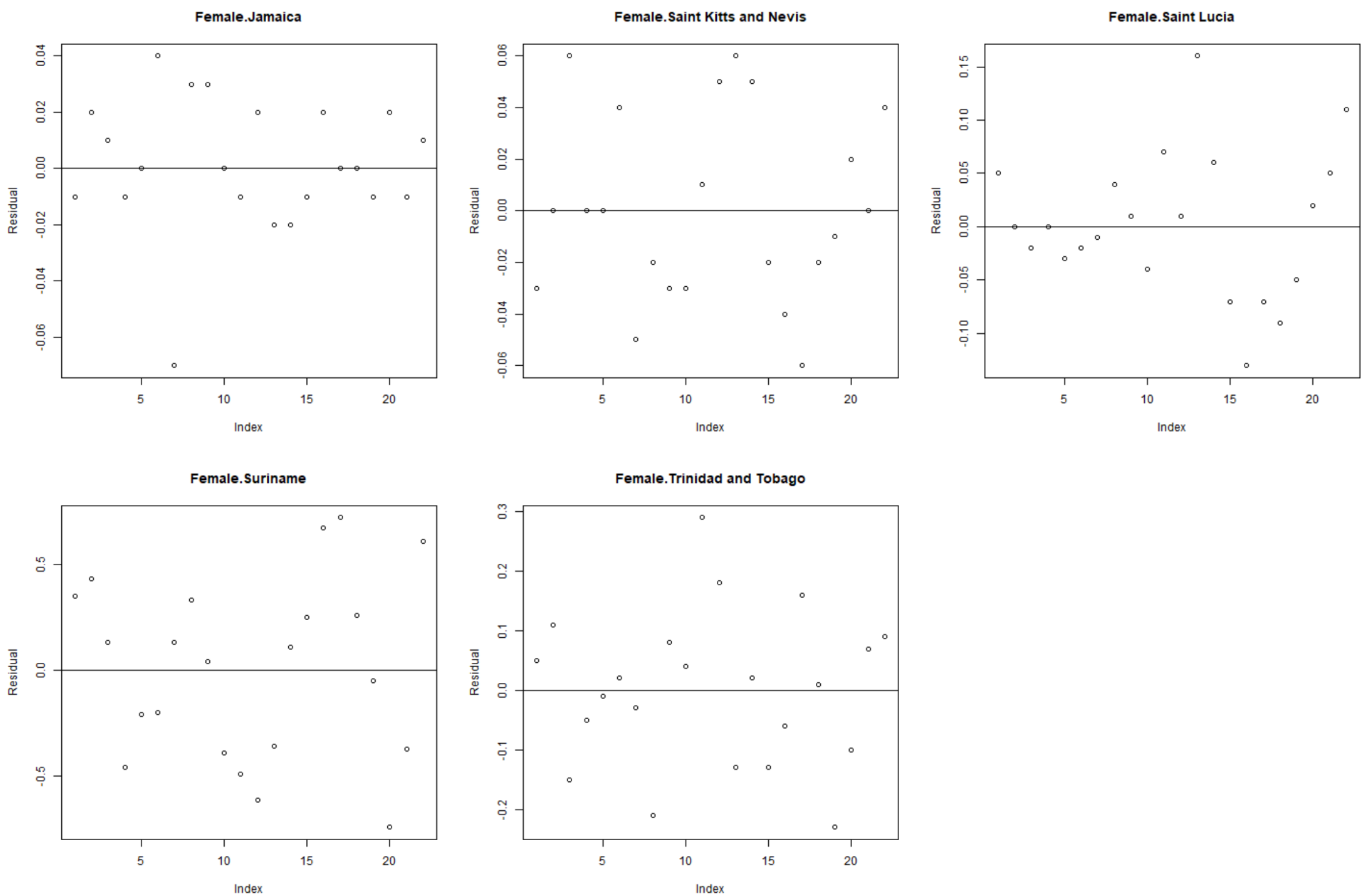
Female.Jamaica
Female.Saint Kitts and Nevis
Female.Saint Lucia
Female.Suriname
Female.Trinidad and Tobago
Residual
Index

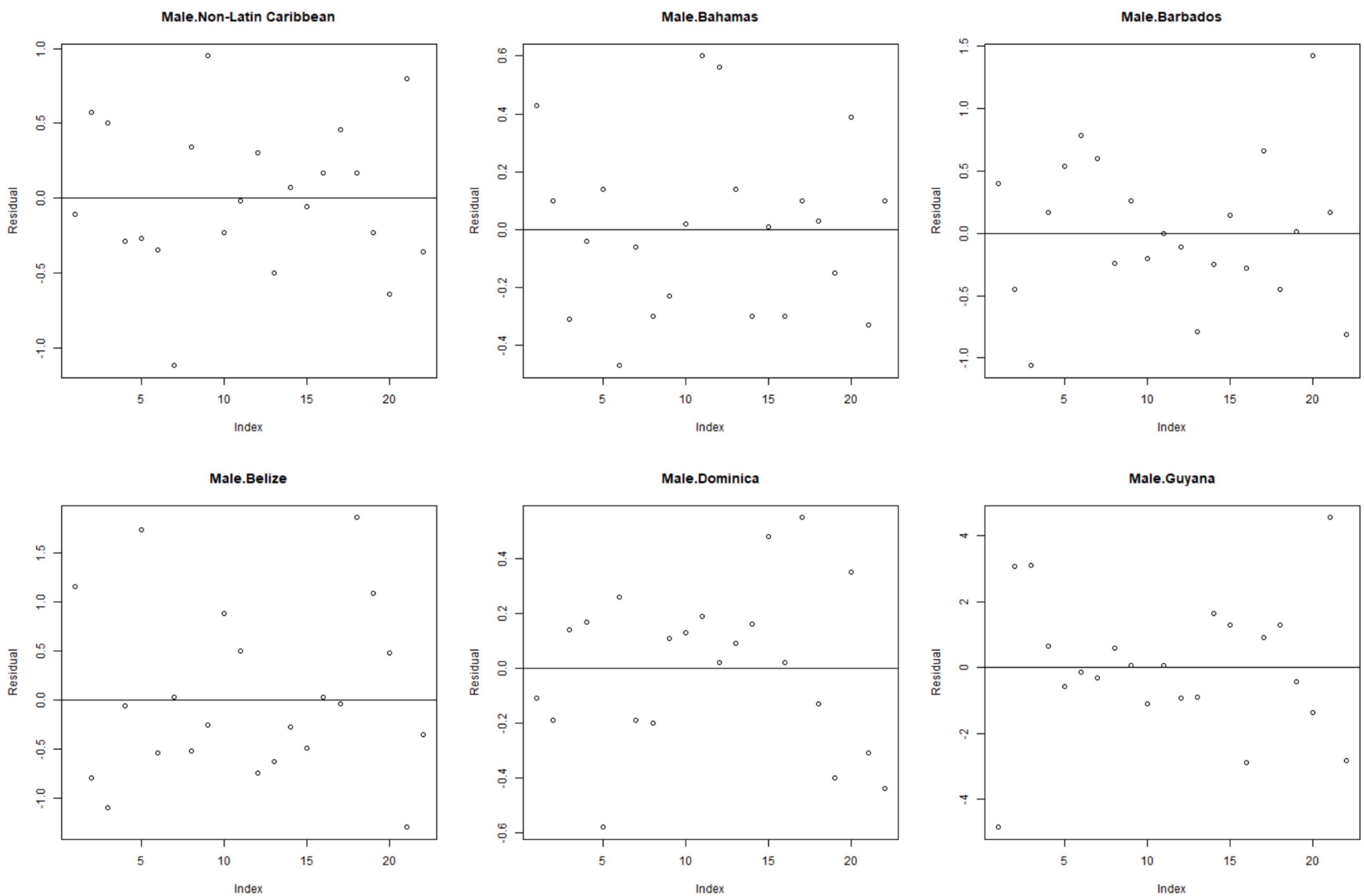
Male.Non-Latin Caribbean
Male.Bahamas
Male.Barbados
Male.Belize
Male.Dominica
Male.Guyana
Residual
Index

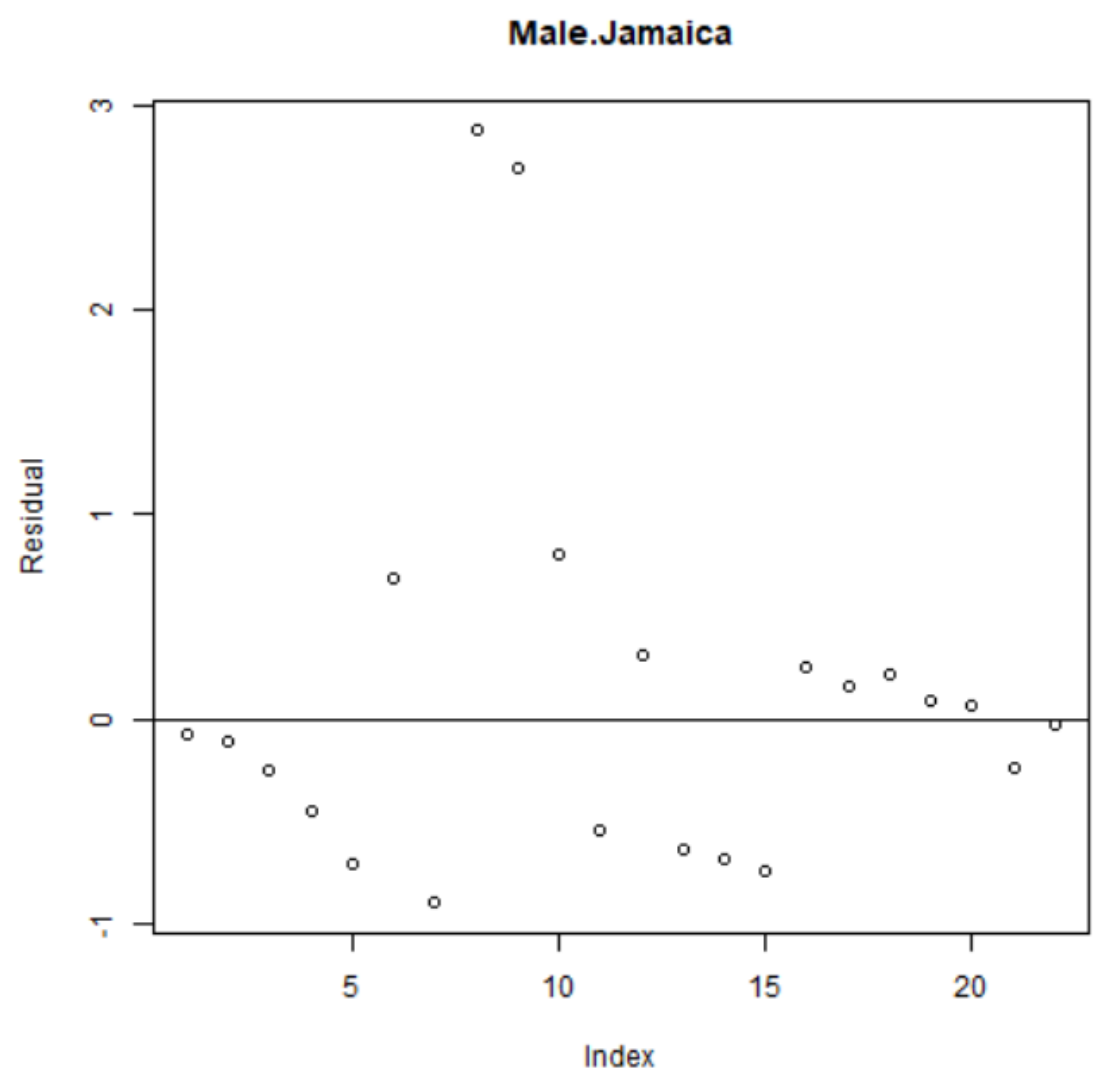
Male.Jamaica
Residual
Index
-1
0
1
2
3
5
10
15
20

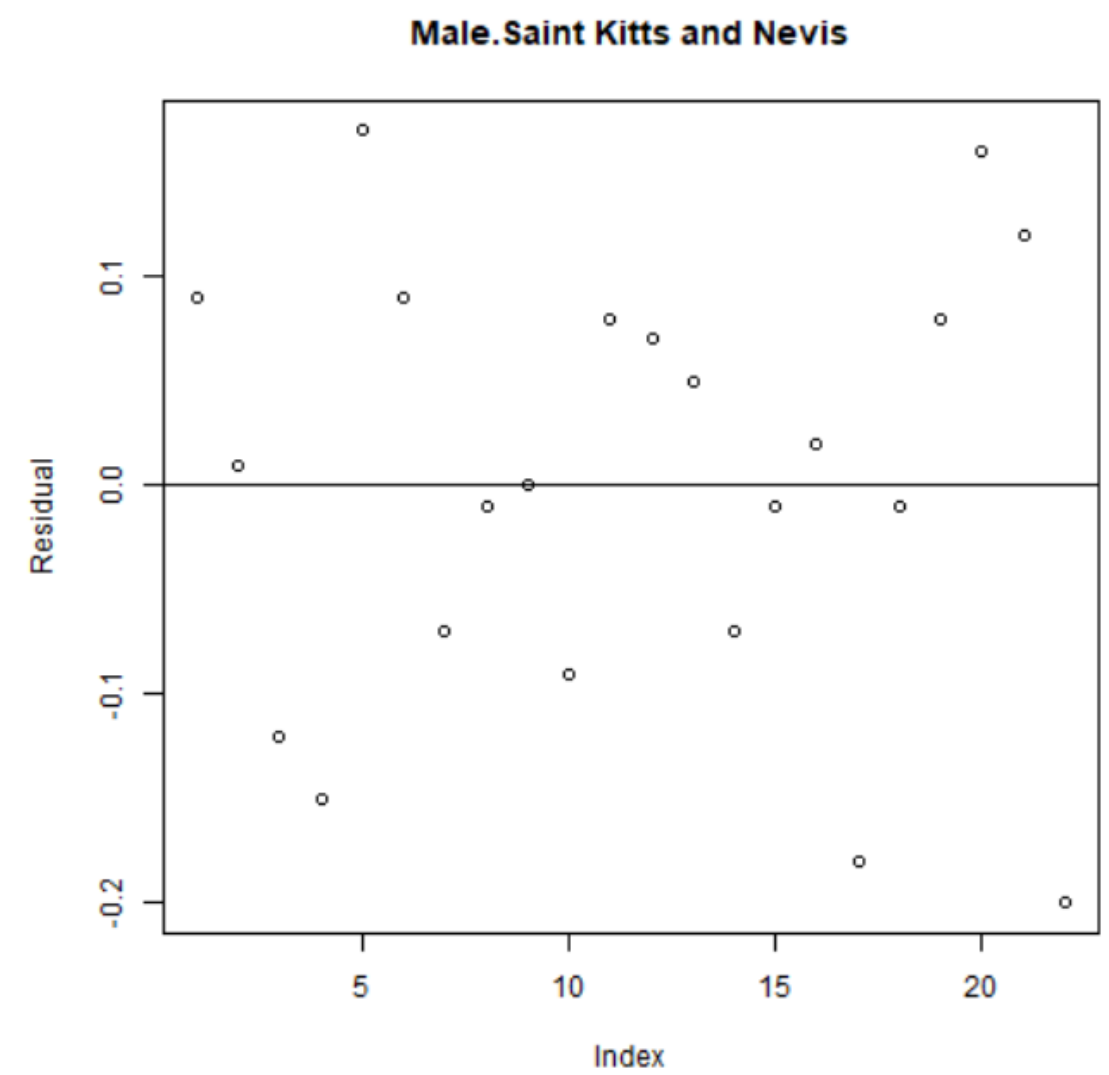
Male.Saint Kitts and Nevis
Residual
Index
-0.2
-0.1
0.0
0.1
5
10
15
20

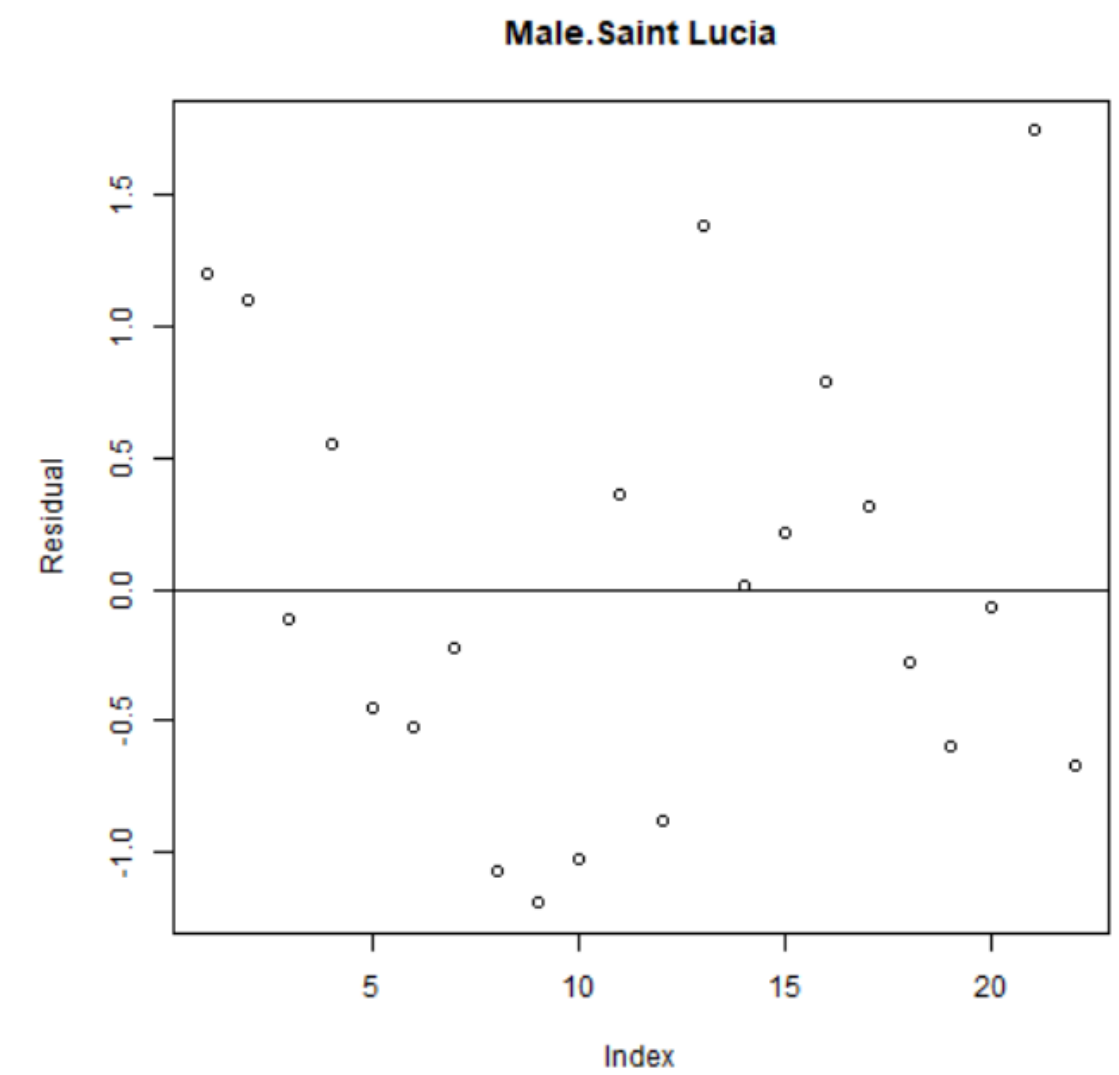
Male.Saint Lucia
Residual
Index
-1.0
-0.5
0.0
0.5
1.0
1.5
5
10
15
20

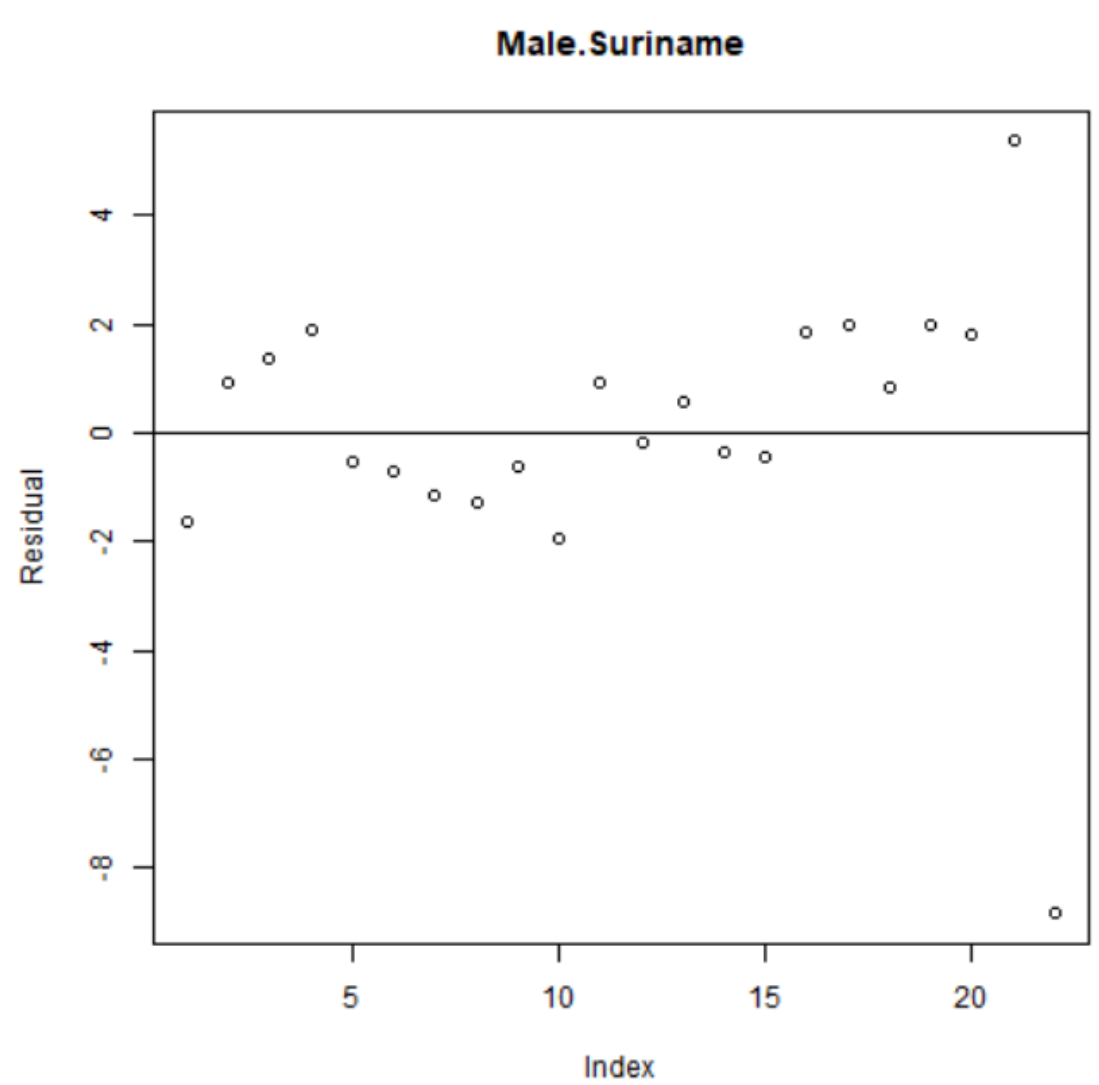
Male.Suriname
Residual
Index
-8
-6
-4
-2
0
2
4
5
10
15
20

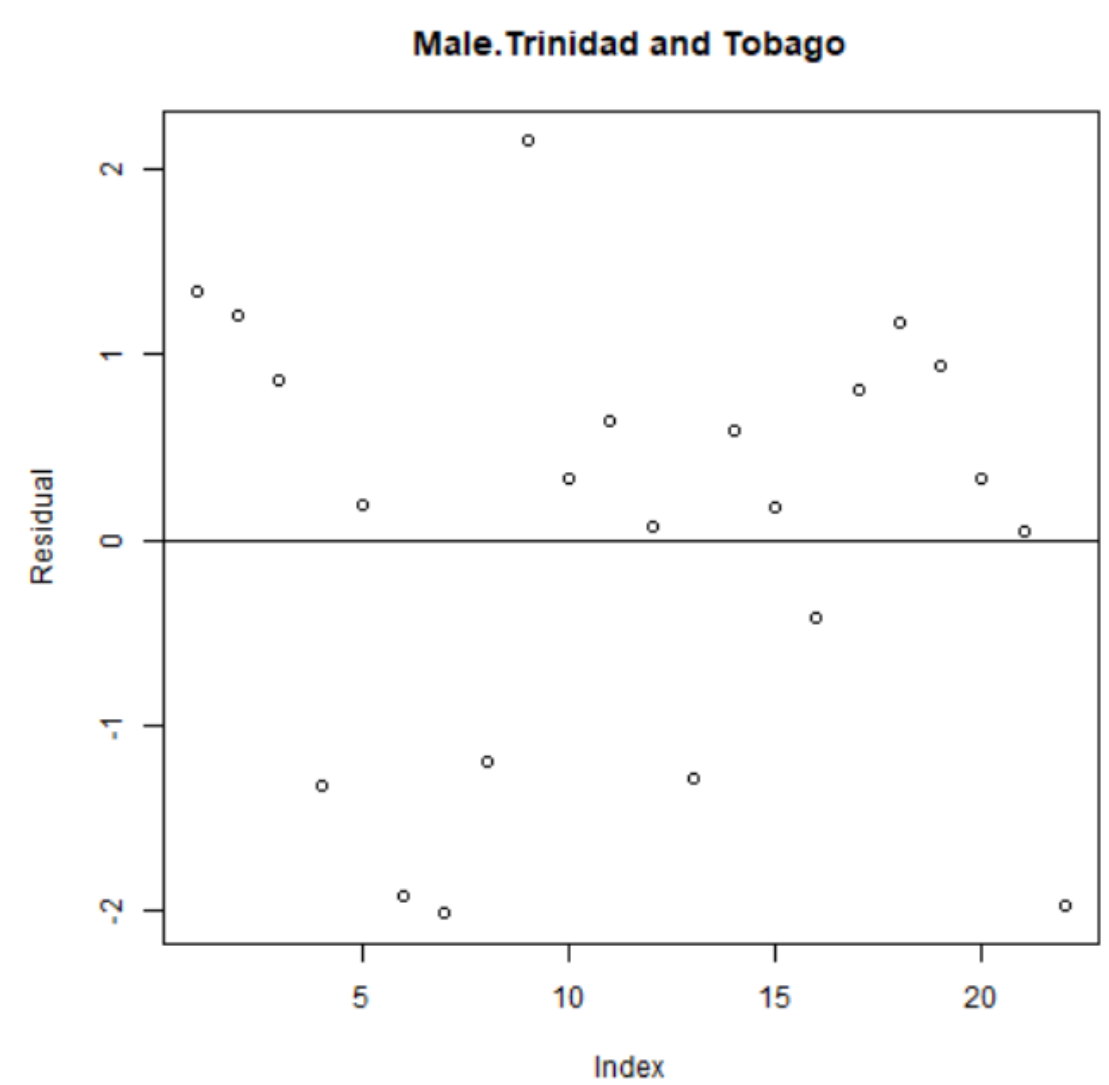
Male.Trinidad and Tobago
Residual
Index
-2
-1
0
1
2
5
10
15
20

(D)

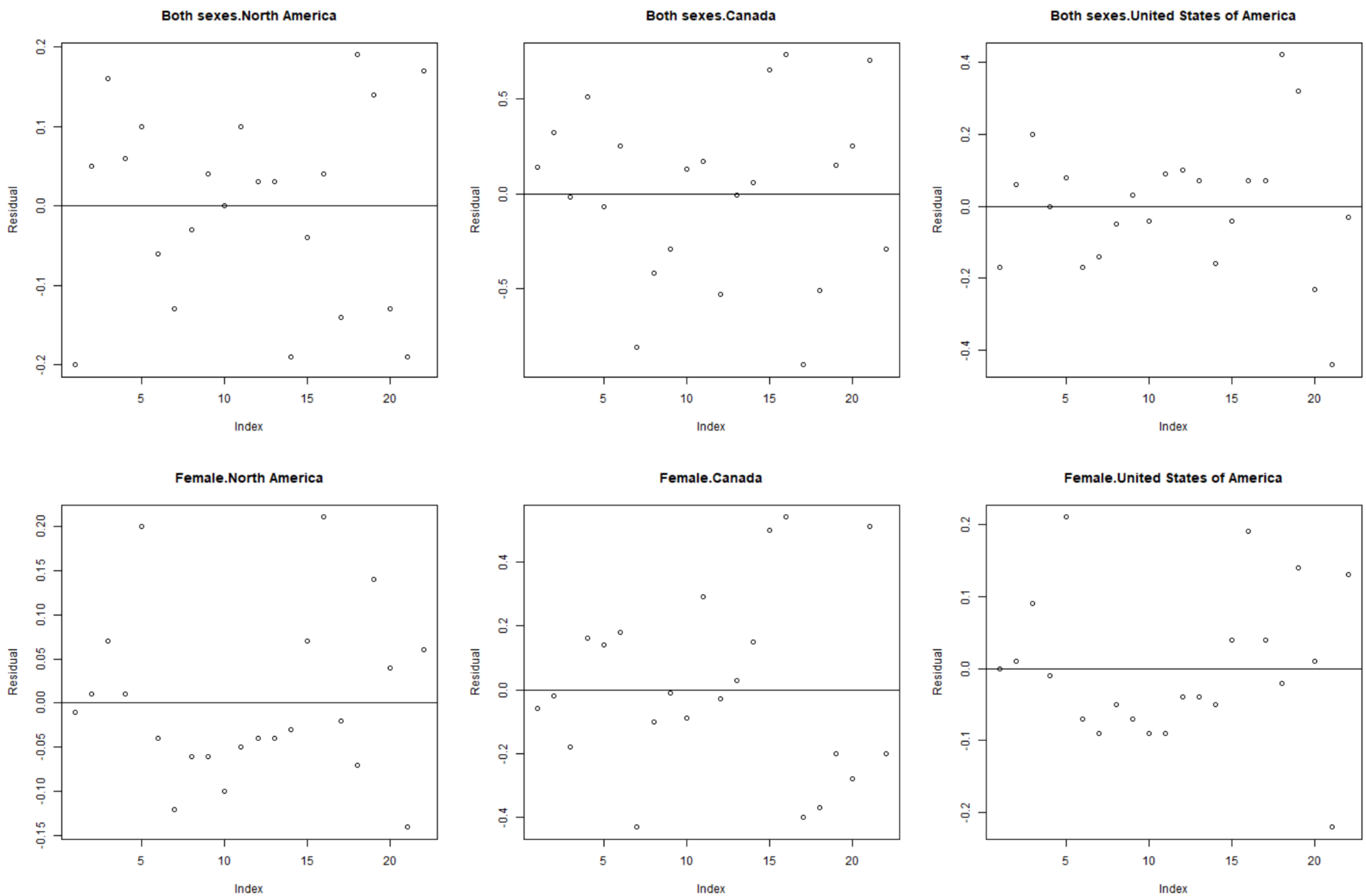

Both sexes.North America
Both sexes.Canada
Both sexes.United States of America
Female.North America
Female.Canada
Female.United States of America
Residual
Index

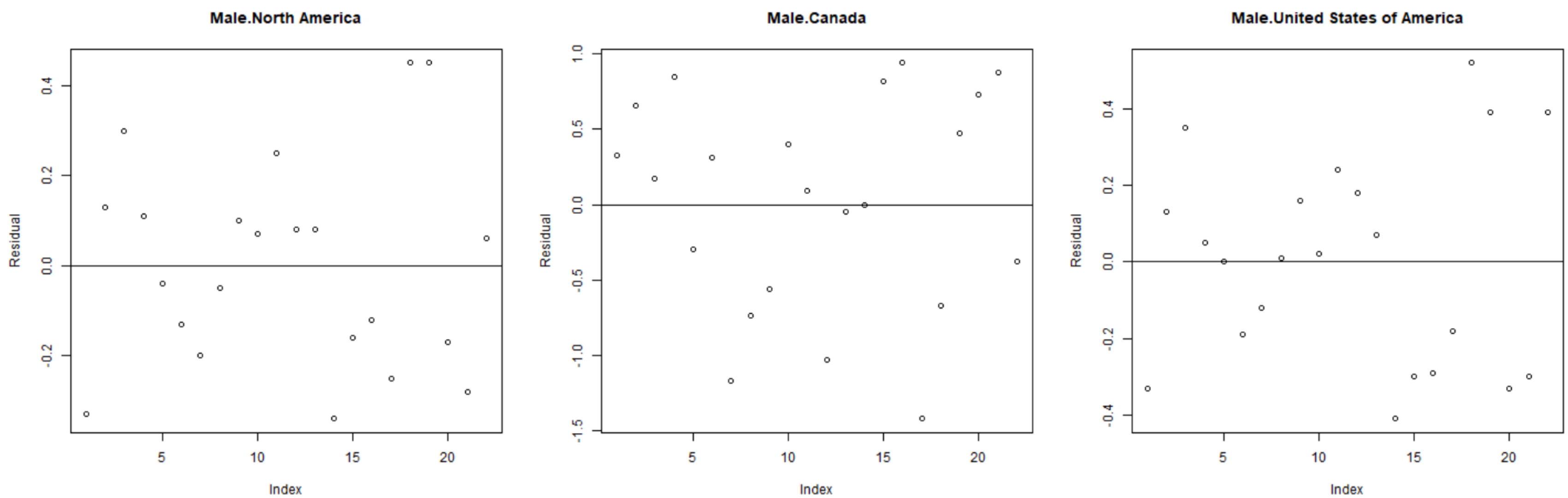
Male.North America
Male.Canada
Male.United States of America
Residual
Index

(E)

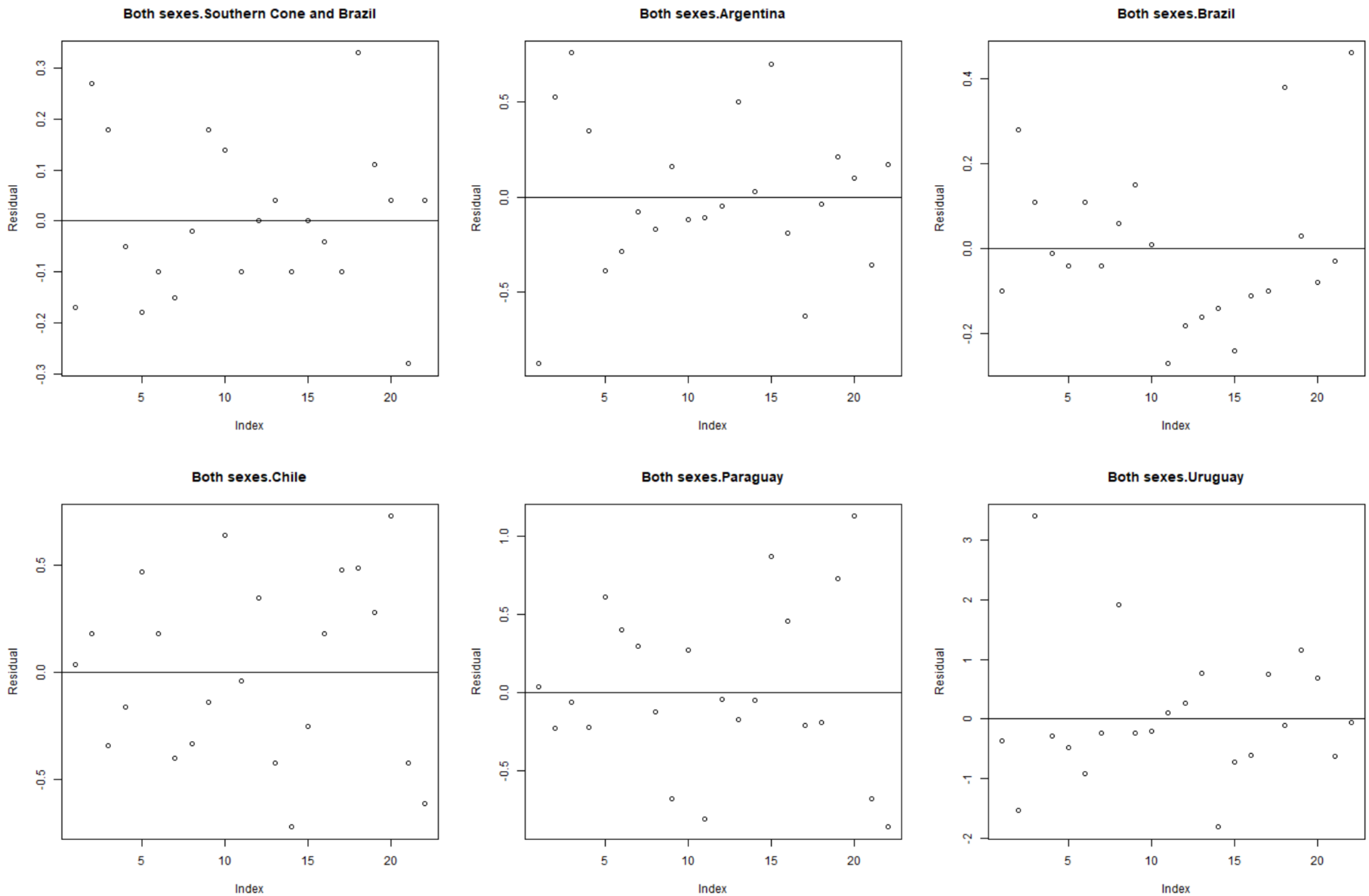
Both sexes.Southern Cone and Brazil
Both sexes.Argentina
Both sexes.Brazil
Both sexes.Chile
Both sexes.Paraguay
Both sexes.Uruguay
Residual
Index

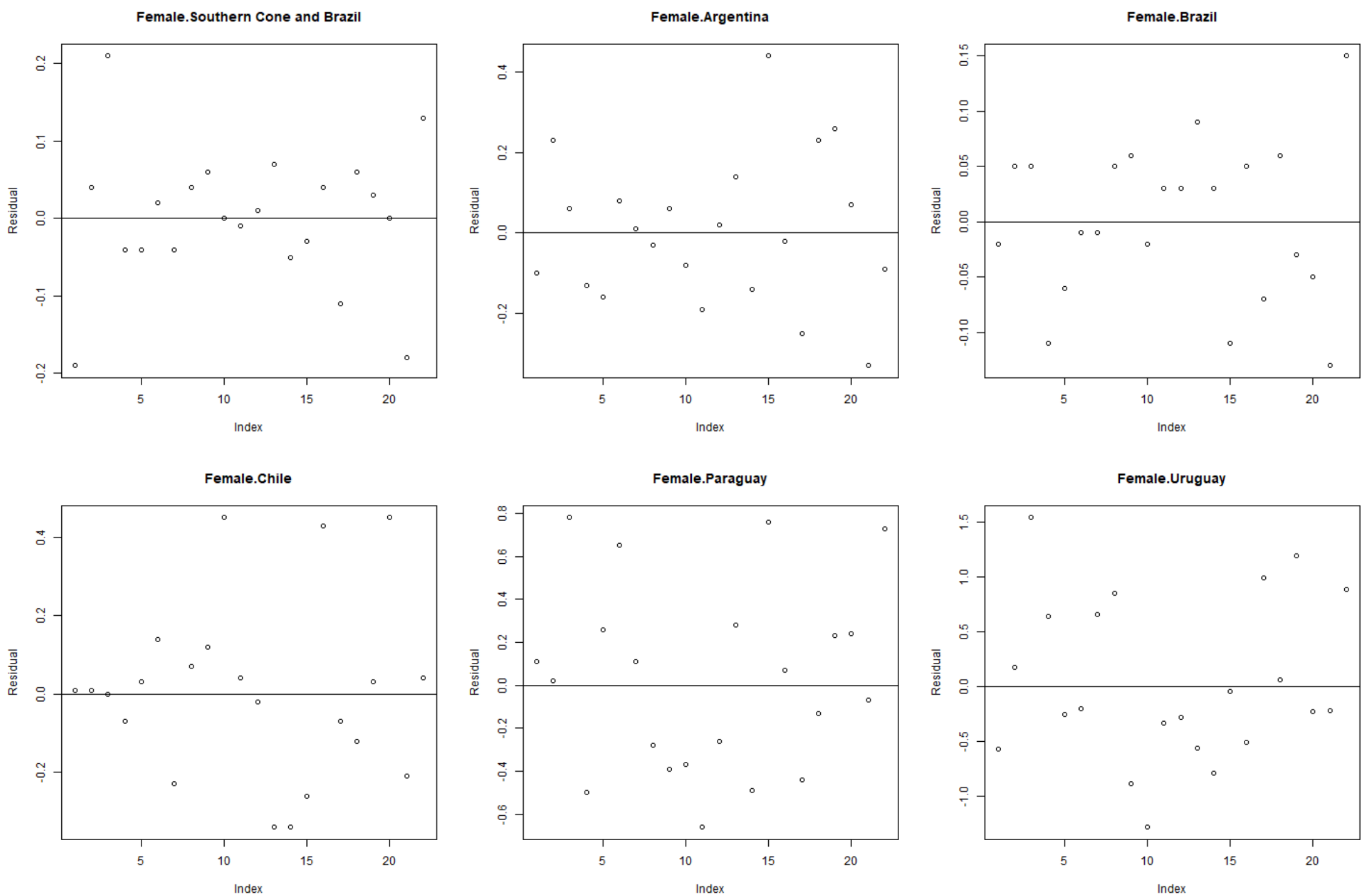
Female.Southern Cone and Brazil
Female.Argentina
Female.Brazil
Female.Chile
Female.Paraguay
Female.Uruguay
Residual
Index

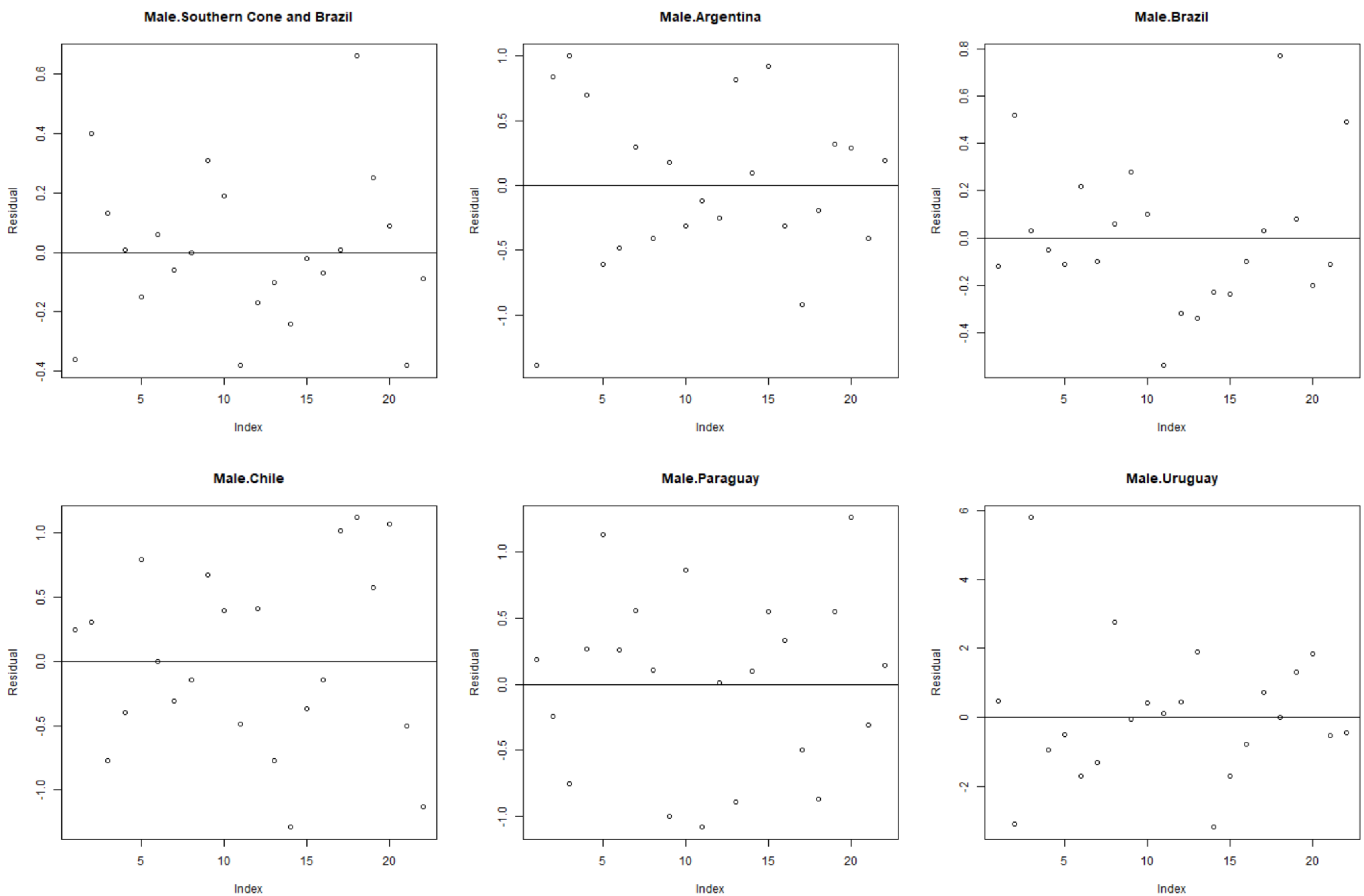
Male.Southern Cone and Brazil
Male.Argentina
Male.Brazil
Male.Chile
Male.Paraguay
Male.Uruguay
Residual
Index

(F)

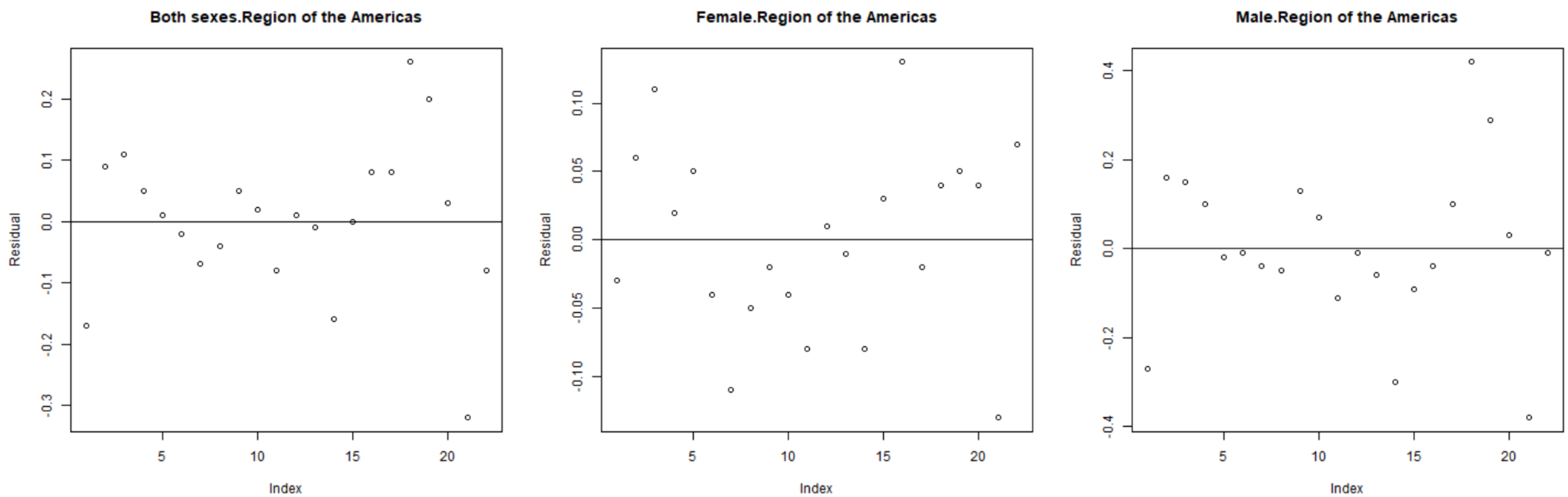
Both sexes.Region of the Americas
Female.Region of the Americas
Male.Region of the Americas
Residual
Index

Figure S2. Autocorrelation function (ACF) plots for suicide mortality rate models for (A) the Andean Area, (B) Central America, Mexico and Latin Caribbean, (C) Non-Latin Caribbean, (D) North America, (E) Southern Cone and Brazil and (F) the Region of the Americas

(A)

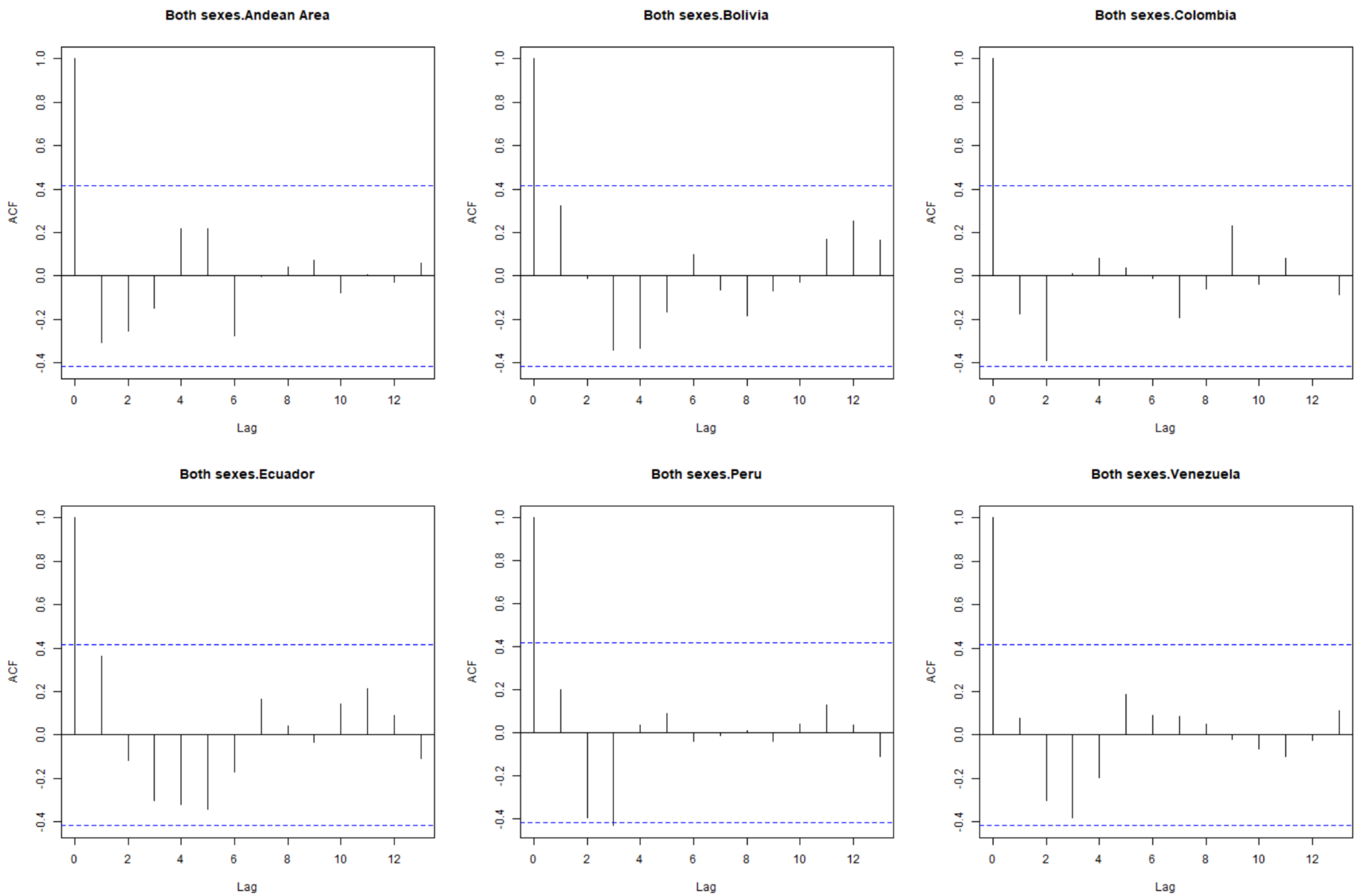

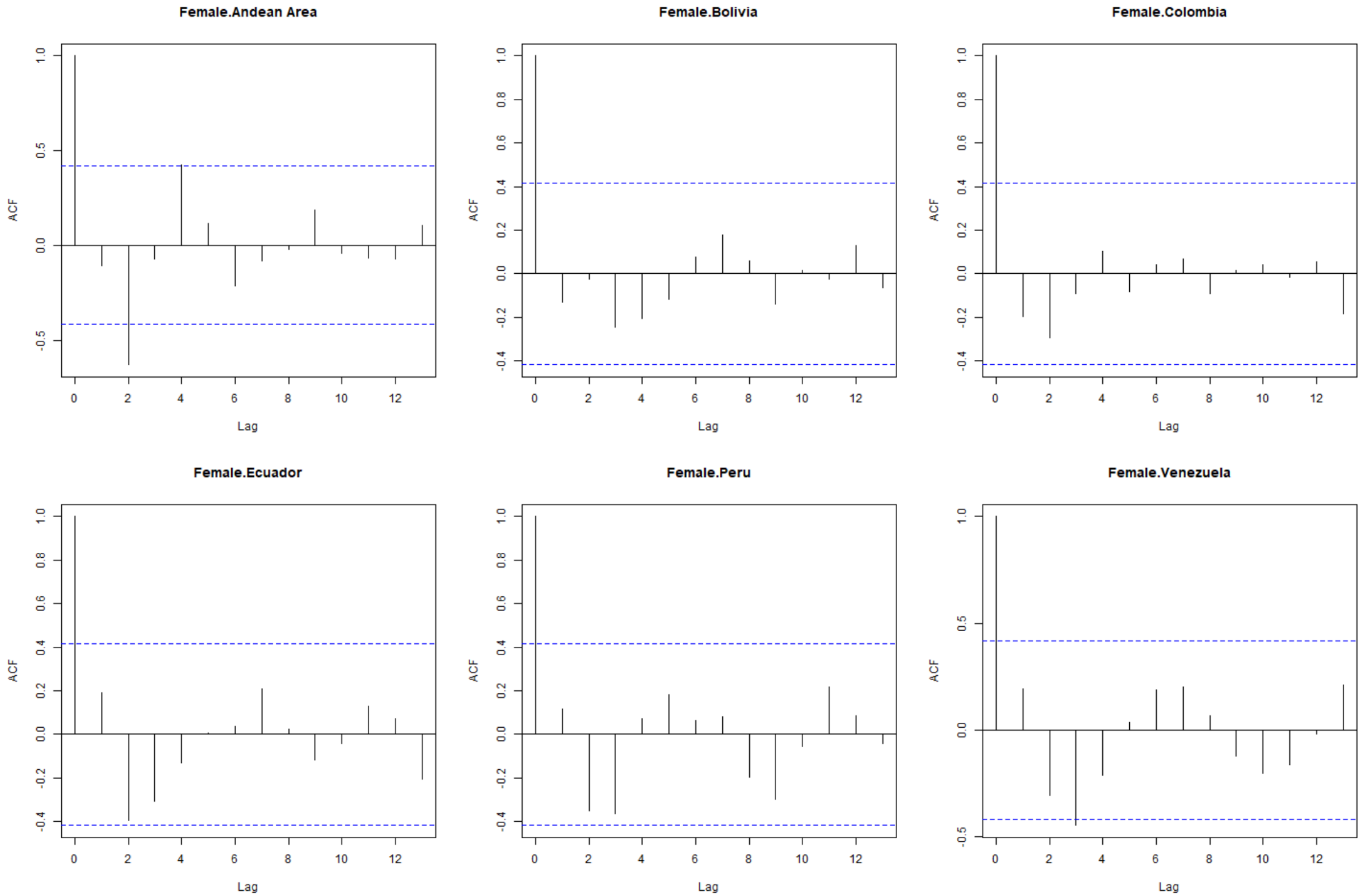
Female.Andean Area
Female.Bolivia
Female.Colombia
Female.Ecuador
Female.Peru
Female.Venezuela
ACF
Lag

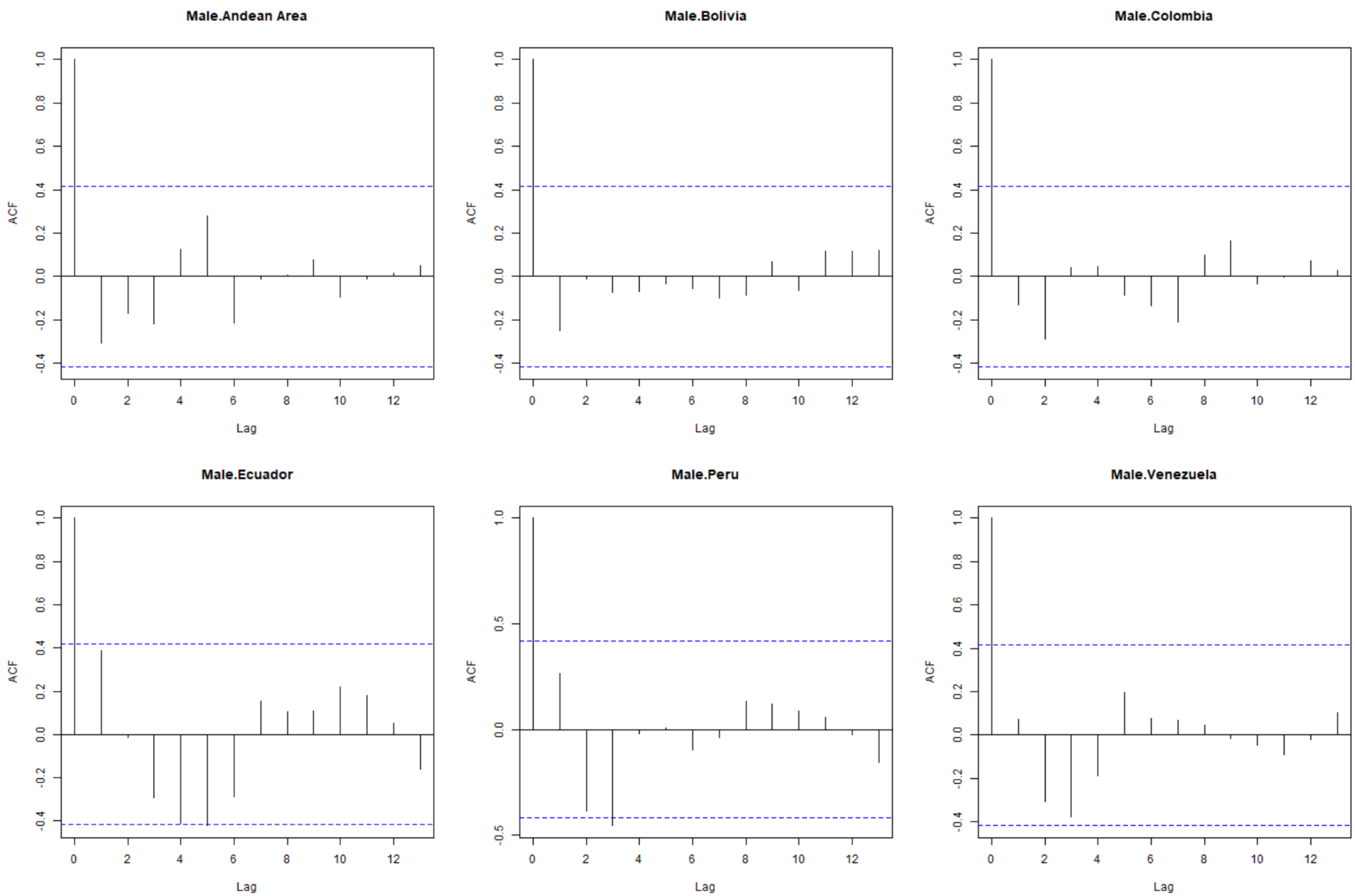
Male.Andean Area
Male.Bolivia
Male.Colombia
Male.Ecuador
Male.Peru
Male.Venezuela
ACF
Lag

(B)

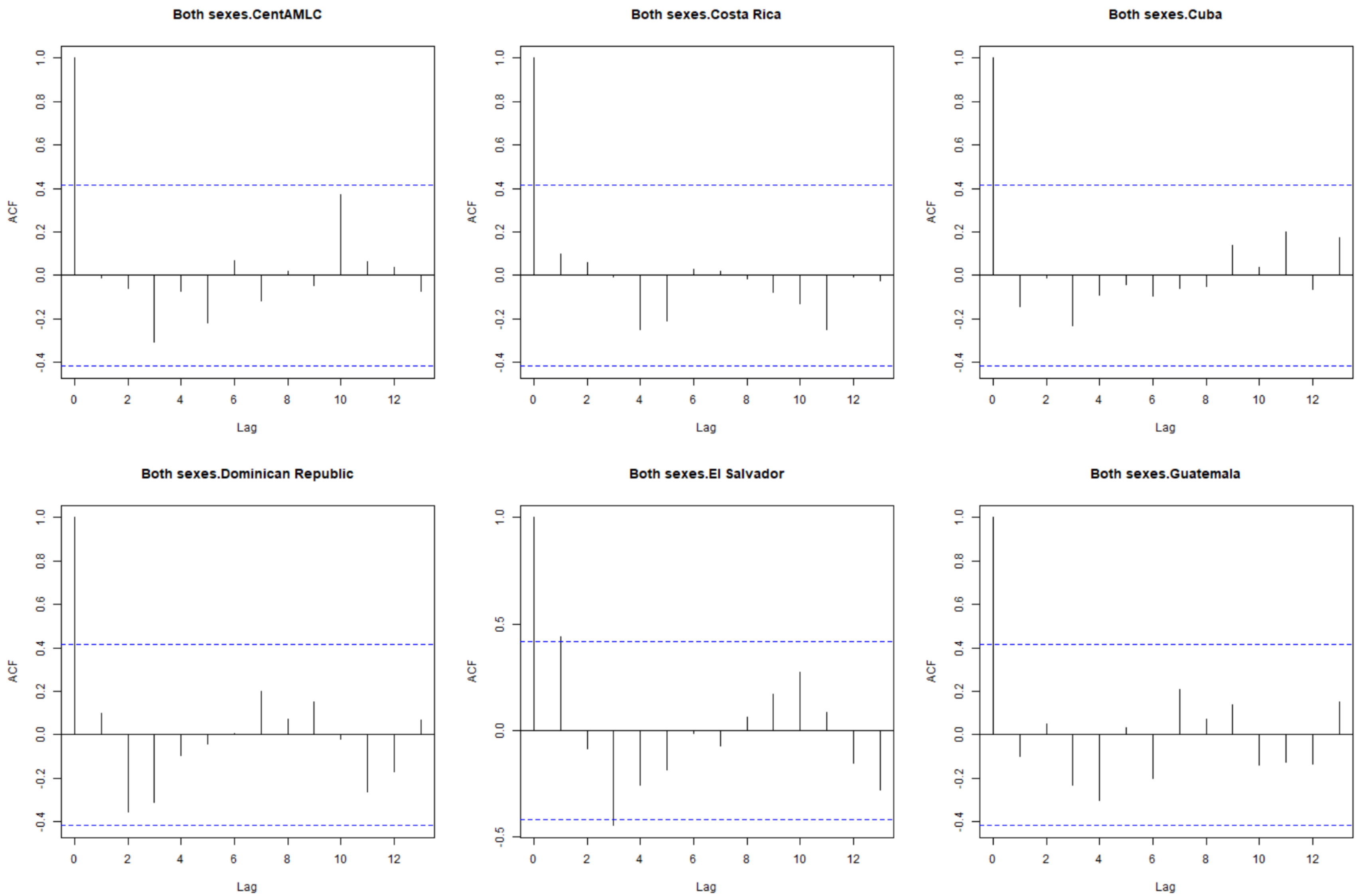
Both sexes.CentAMLC
Both sexes.Costa Rica
Both sexes.Cuba
Both sexes.Dominican Republic
Both sexes.El Salvador
Both sexes.Guatemala
ACF
Lag

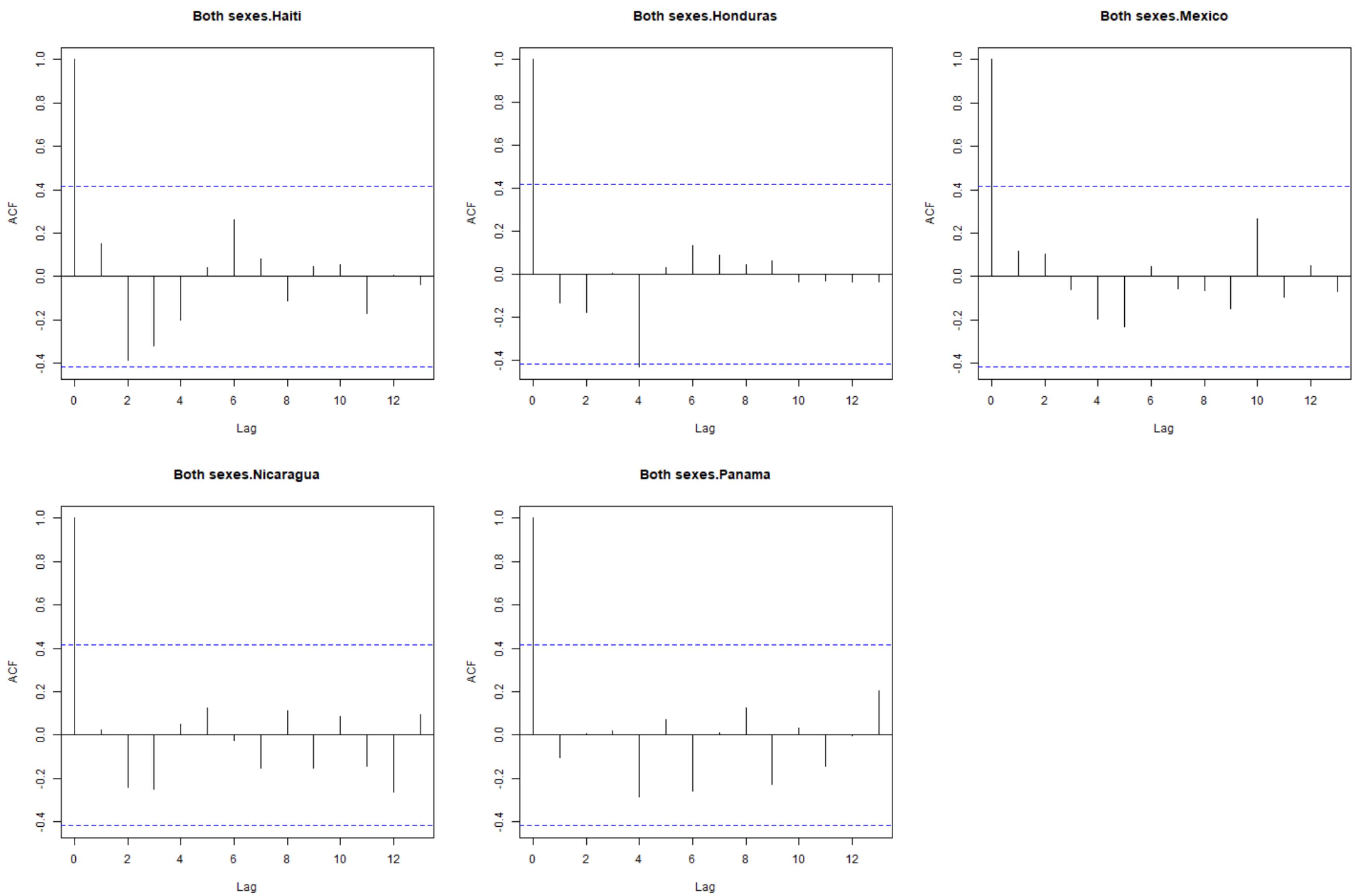
Both sexes.Haiti
Both sexes.Honduras
Both sexes.Mexico
Both sexes.Nicaragua
Both sexes.Panama
ACF
Lag
-0.4
-0.2
0.0
0.2
0.4
0.6
0.8
1.0
0
2
4
6
8
10
12

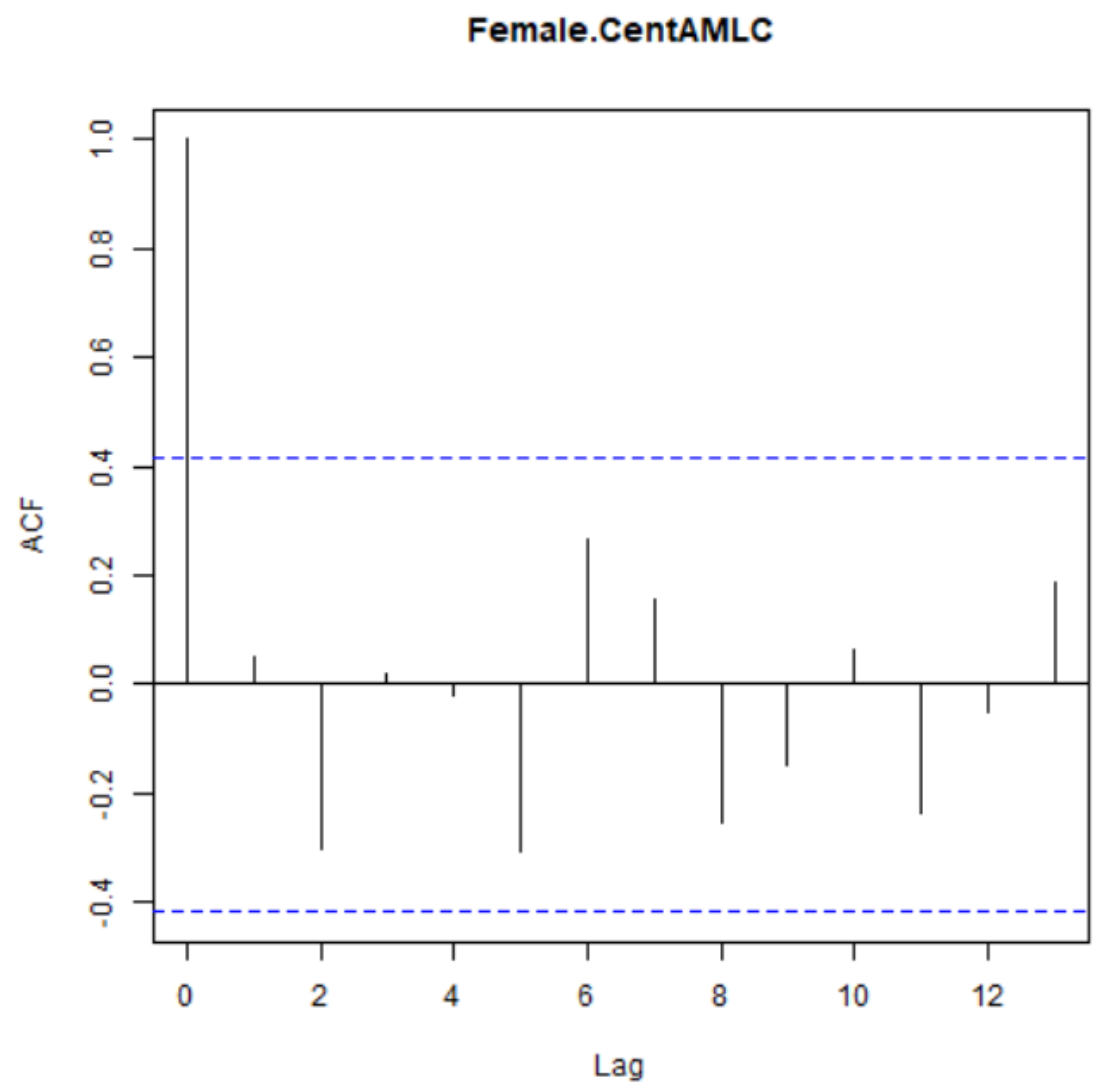
Female.CentAMLC
ACF
1.0 0.8 0.6 0.4 0.2 0.0 -0.2 -0.4
0 2 4 6 8 10 12
Lag

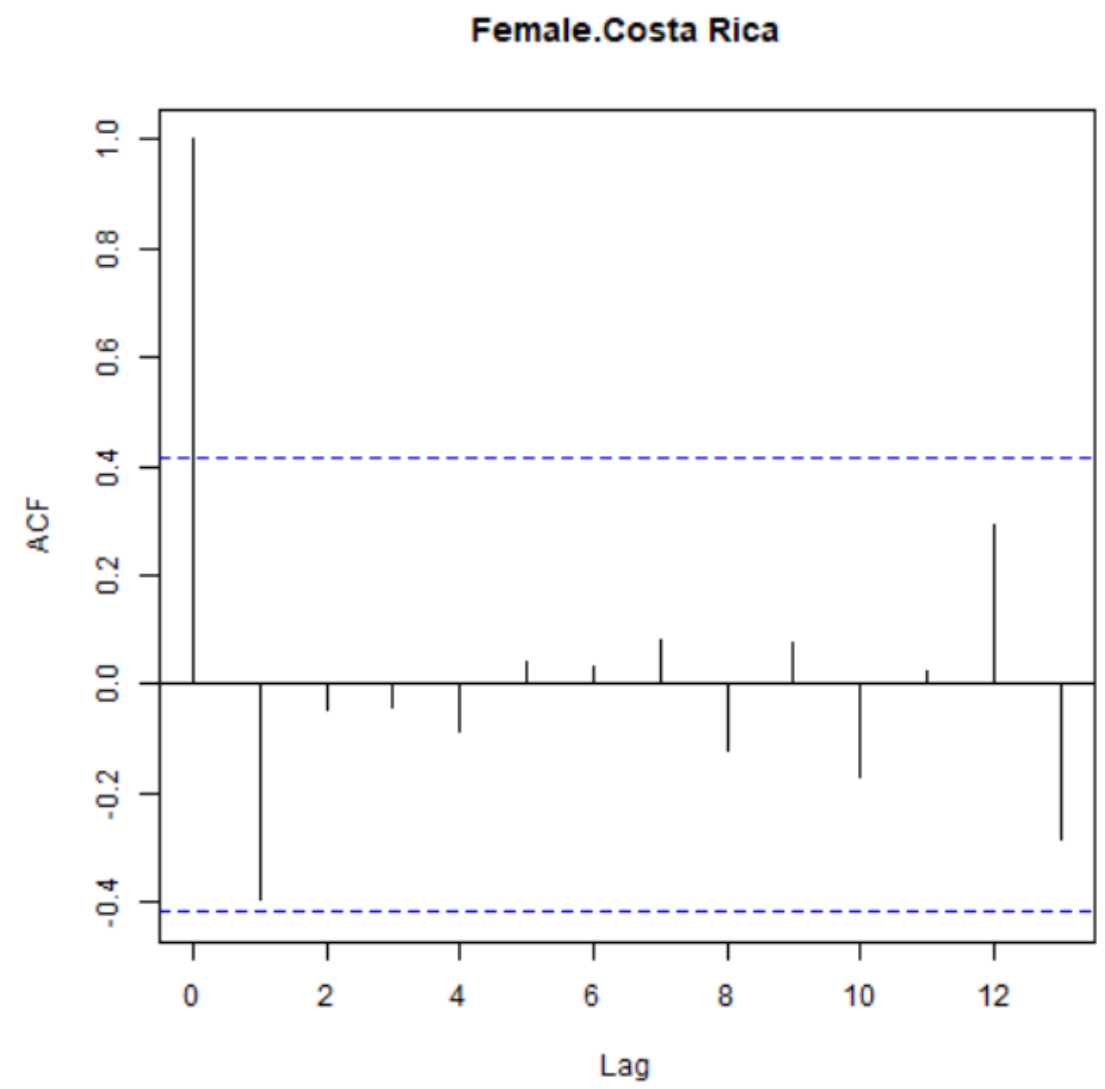
Female.Costa Rica
ACF
1.0 0.8 0.6 0.4 0.2 0.0 -0.2 -0.4
0 2 4 6 8 10 12
Lag

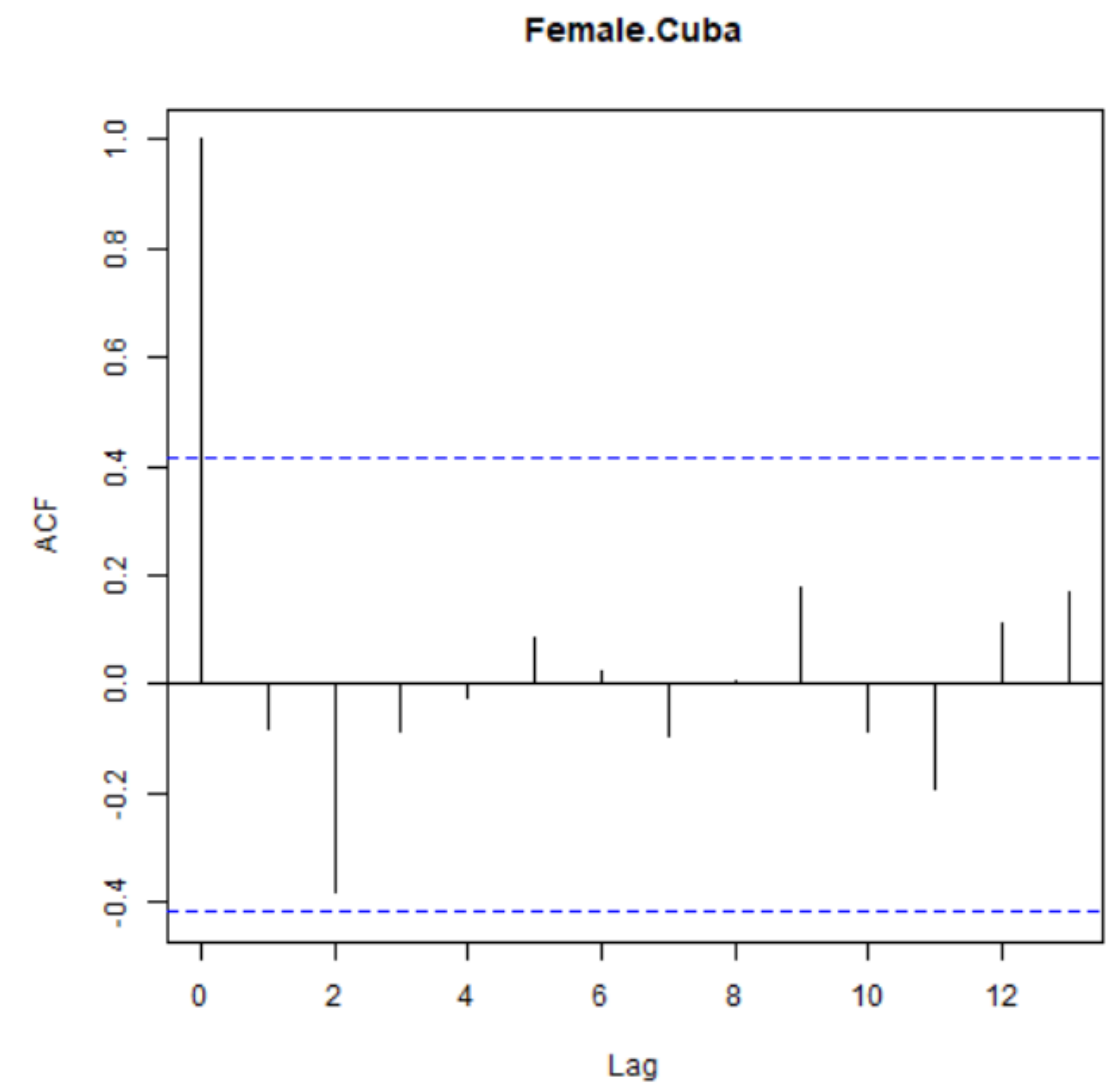
Female.Cuba
ACF
1.0 0.8 0.6 0.4 0.2 0.0 -0.2 -0.4
0 2 4 6 8 10 12
Lag

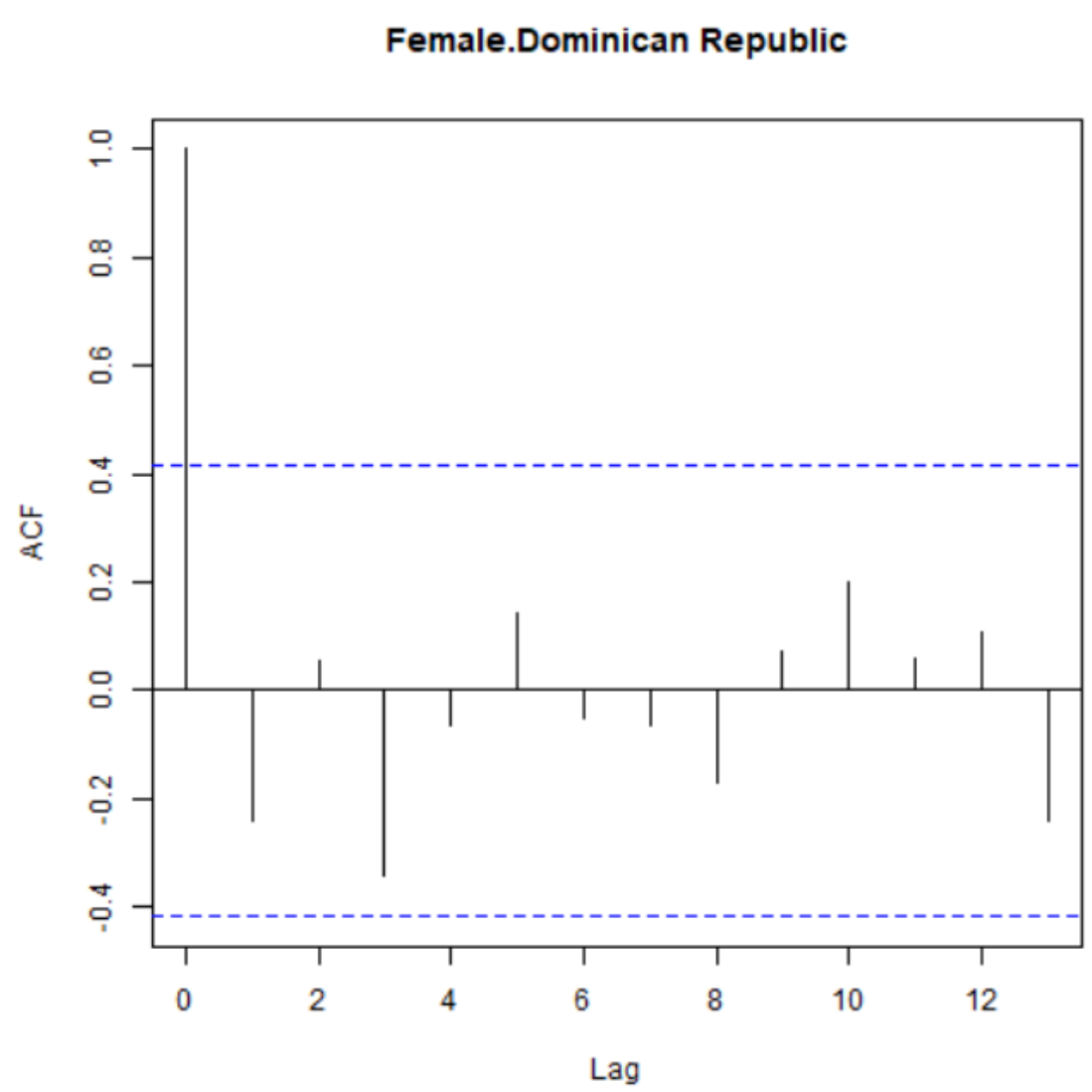
Female.Dominican Republic
ACF
1.0 0.8 0.6 0.4 0.2 0.0 -0.2 -0.4
0 2 4 6 8 10 12
Lag

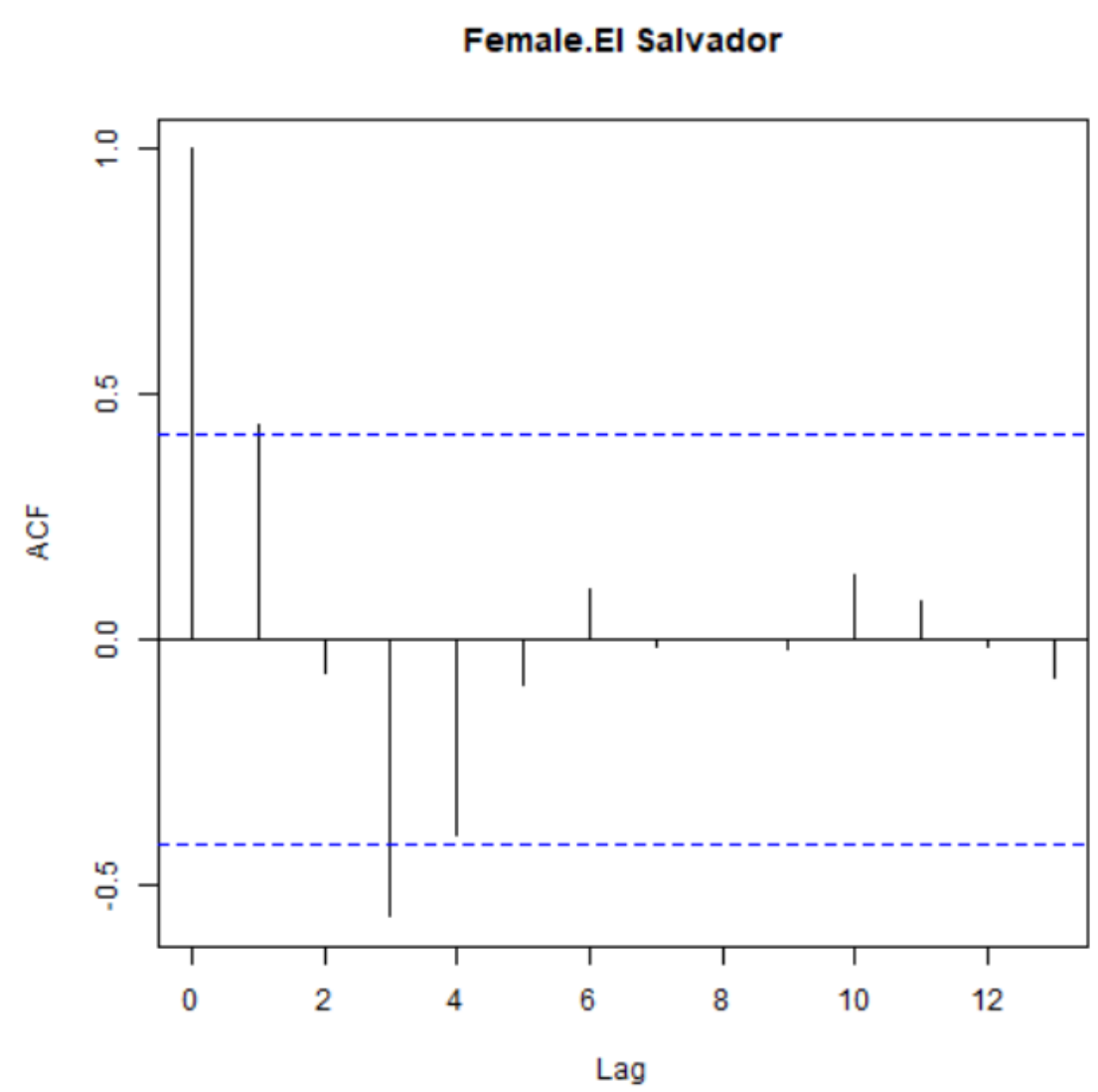
Female.El Salvador
ACF
1.0 0.5 0.0 -0.5
0 2 4 6 8 10 12
Lag

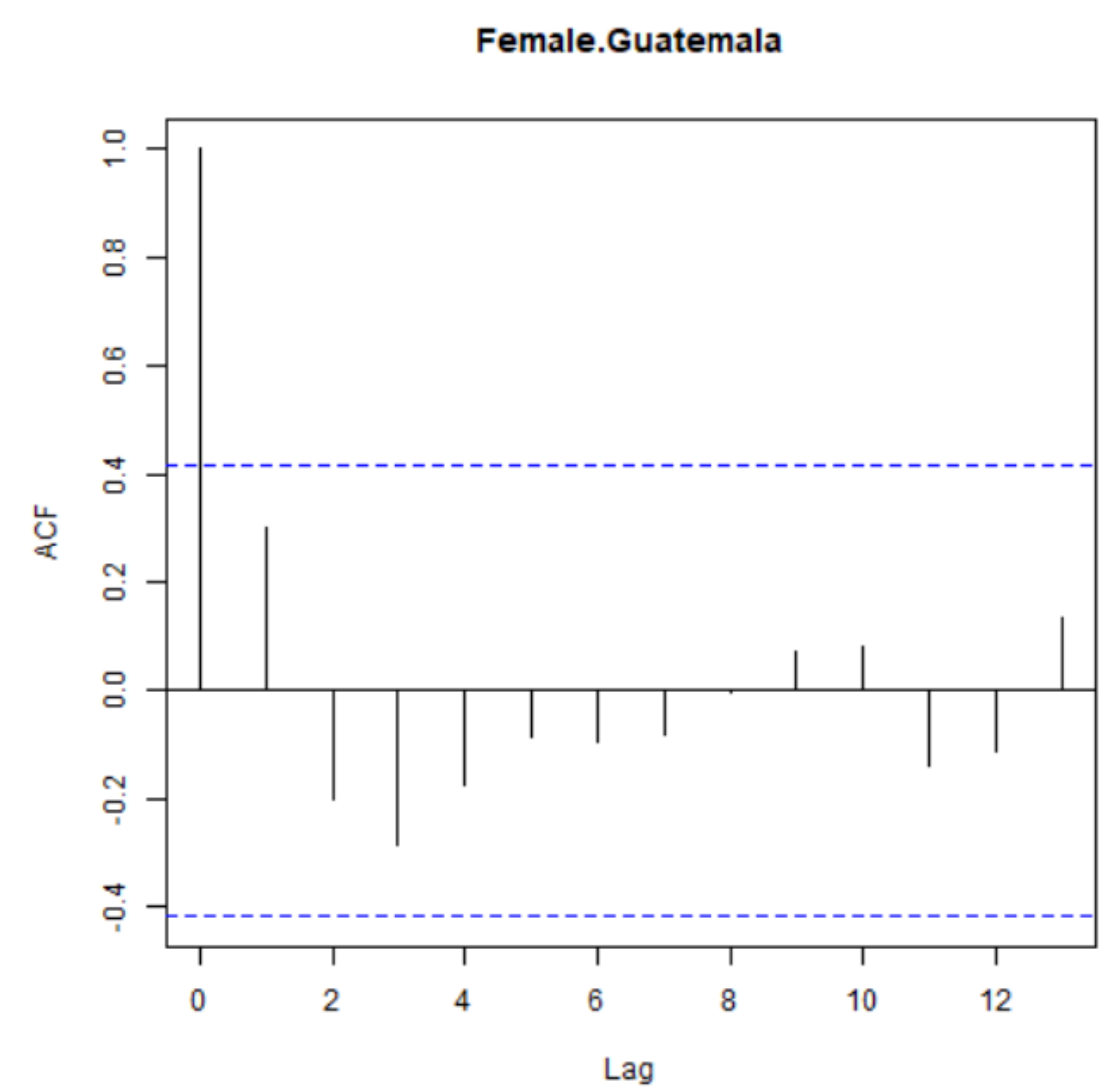
Female.Guatemala
ACF
1.0 0.8 0.6 0.4 0.2 0.0 -0.2 -0.4
0 2 4 6 8 10 12
Lag

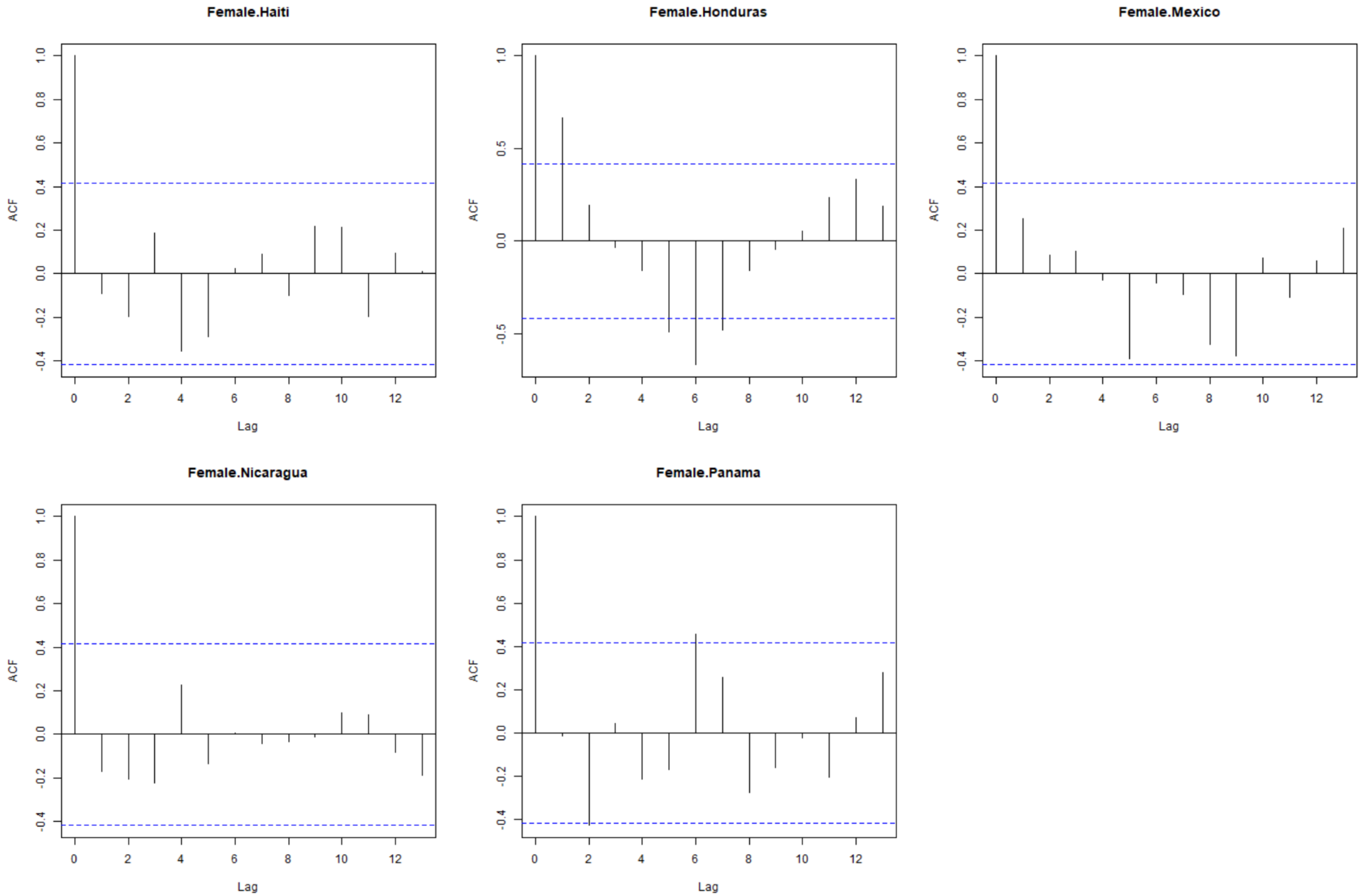
Female.Haiti
Female.Honduras
Female.Mexico
Female.Nicaragua
Female.Panama
ACF
Lag

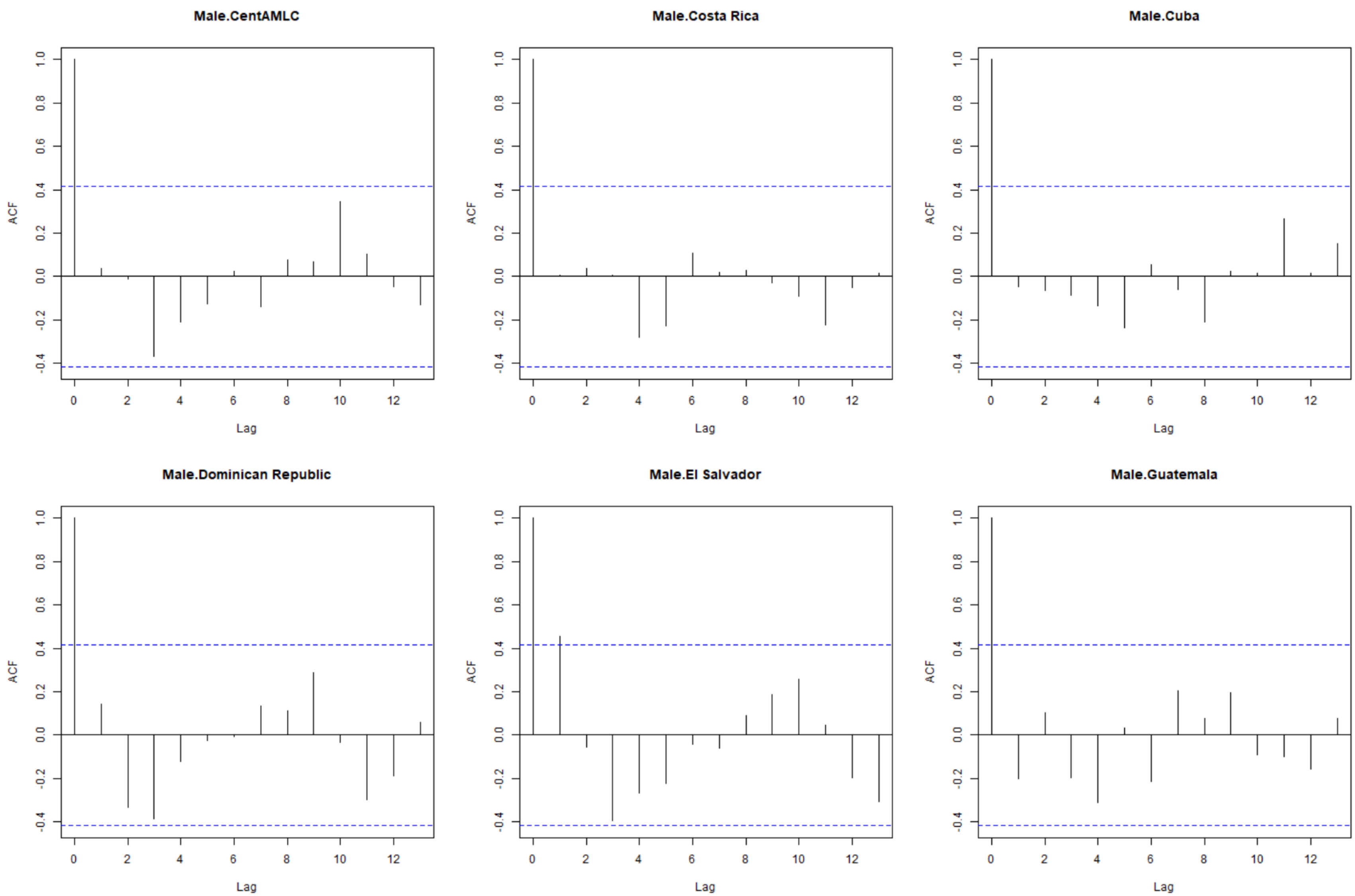
Male.CentAMLC
Male.Costa Rica
Male.Cuba
Male.Dominican Republic
Male.El Salvador
Male.Guatemala
ACF
Lag
1.0
0.8
0.6
0.4
0.2
0.0
-0.2
-0.4
0
2
4
6
8
10
12

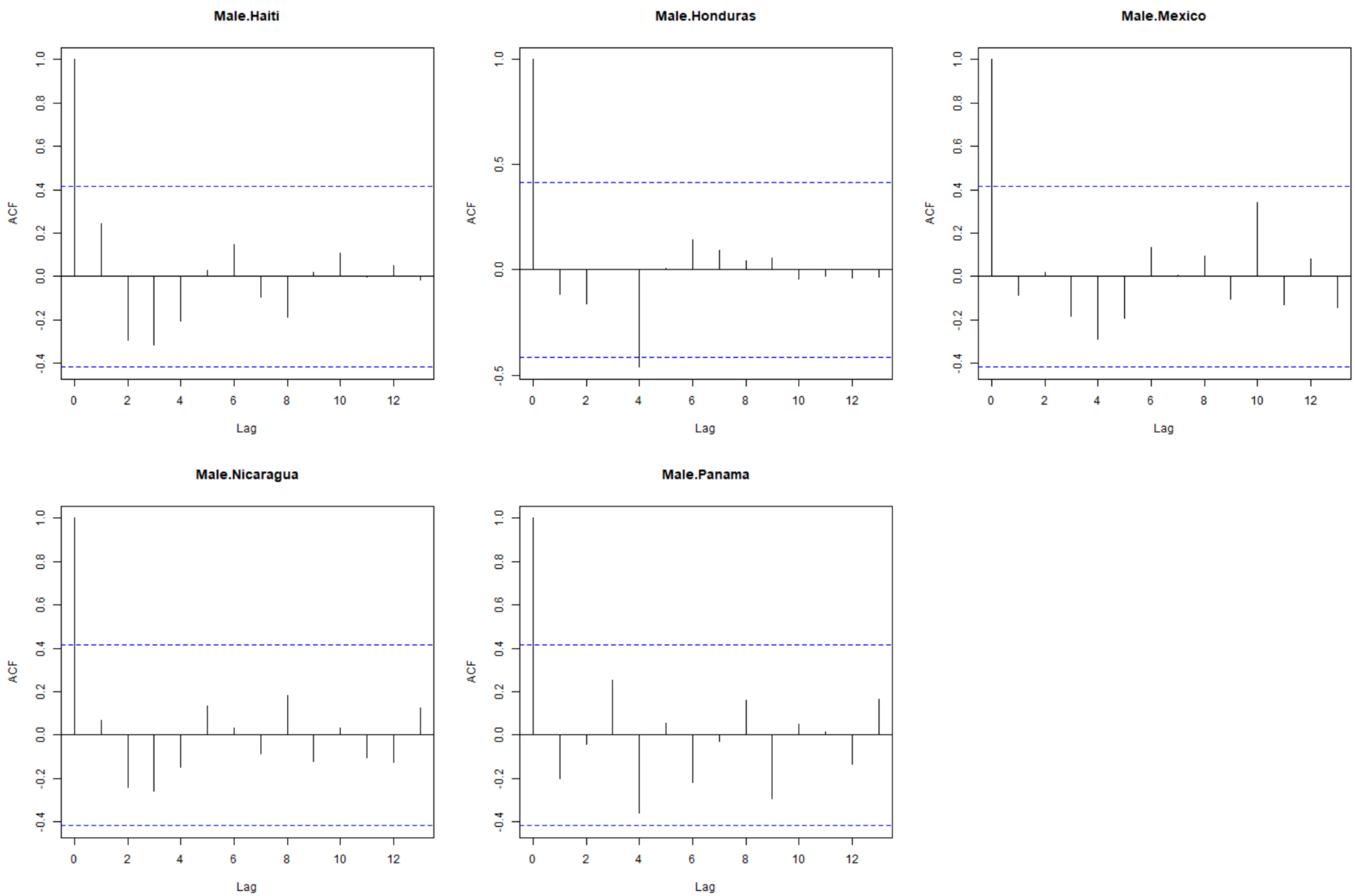
Male.Haiti
Male.Honduras
Male.Mexico
Male.Nicaragua
Male.Panama
ACF
Lag
1.0
0.8
0.6
0.4
0.2
0.0
-0.2
-0.4
0.5
-0.5
0
2
4
6
8
10
12

(C)

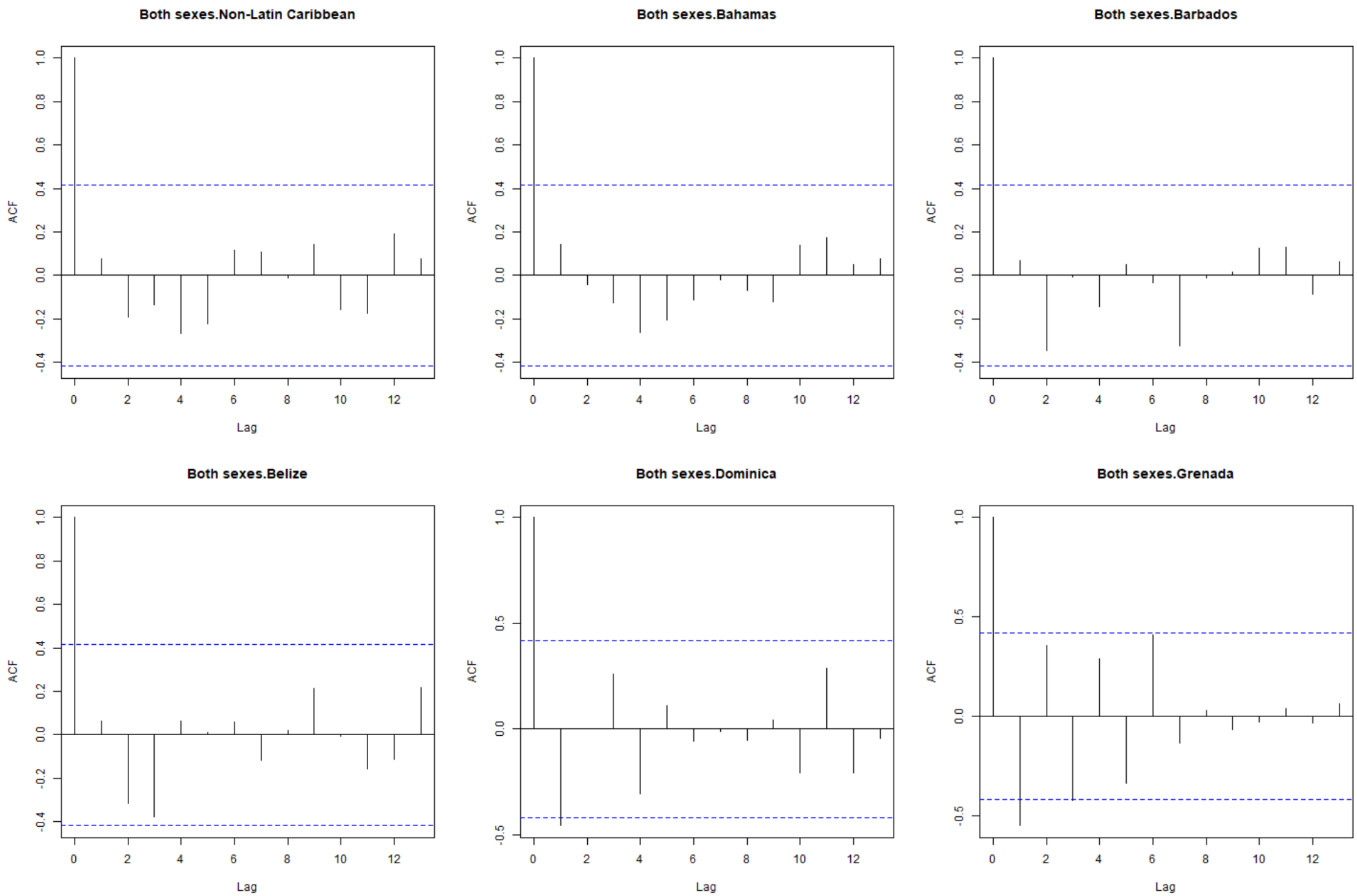
Both sexes.Non-Latin Caribbean
Both sexes.Bahamas
Both sexes.Barbados
Both sexes.Belize
Both sexes.Dominica
Both sexes.Grenada
ACF
Lag

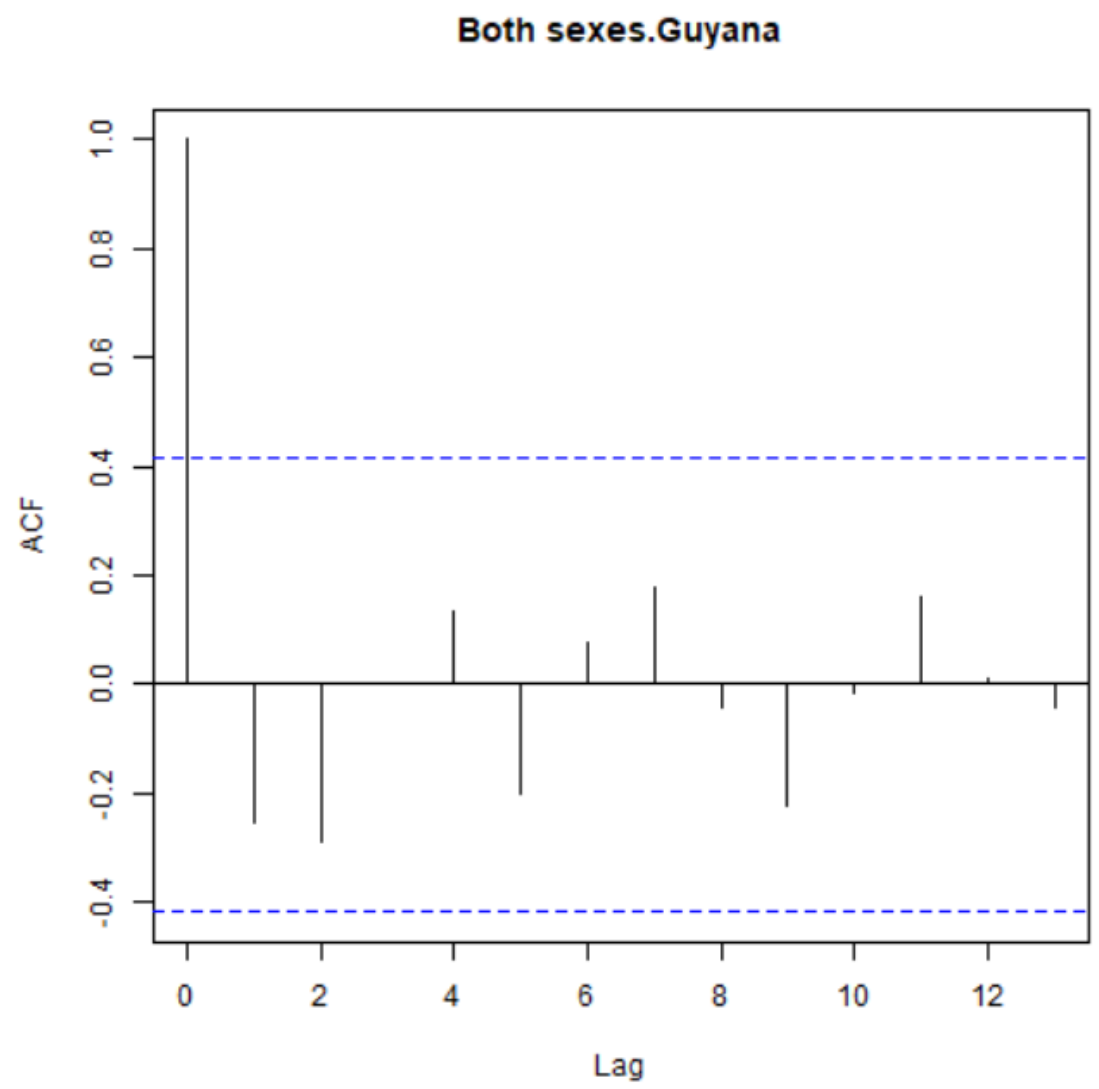
Both sexes.Guyana
ACF
Lag

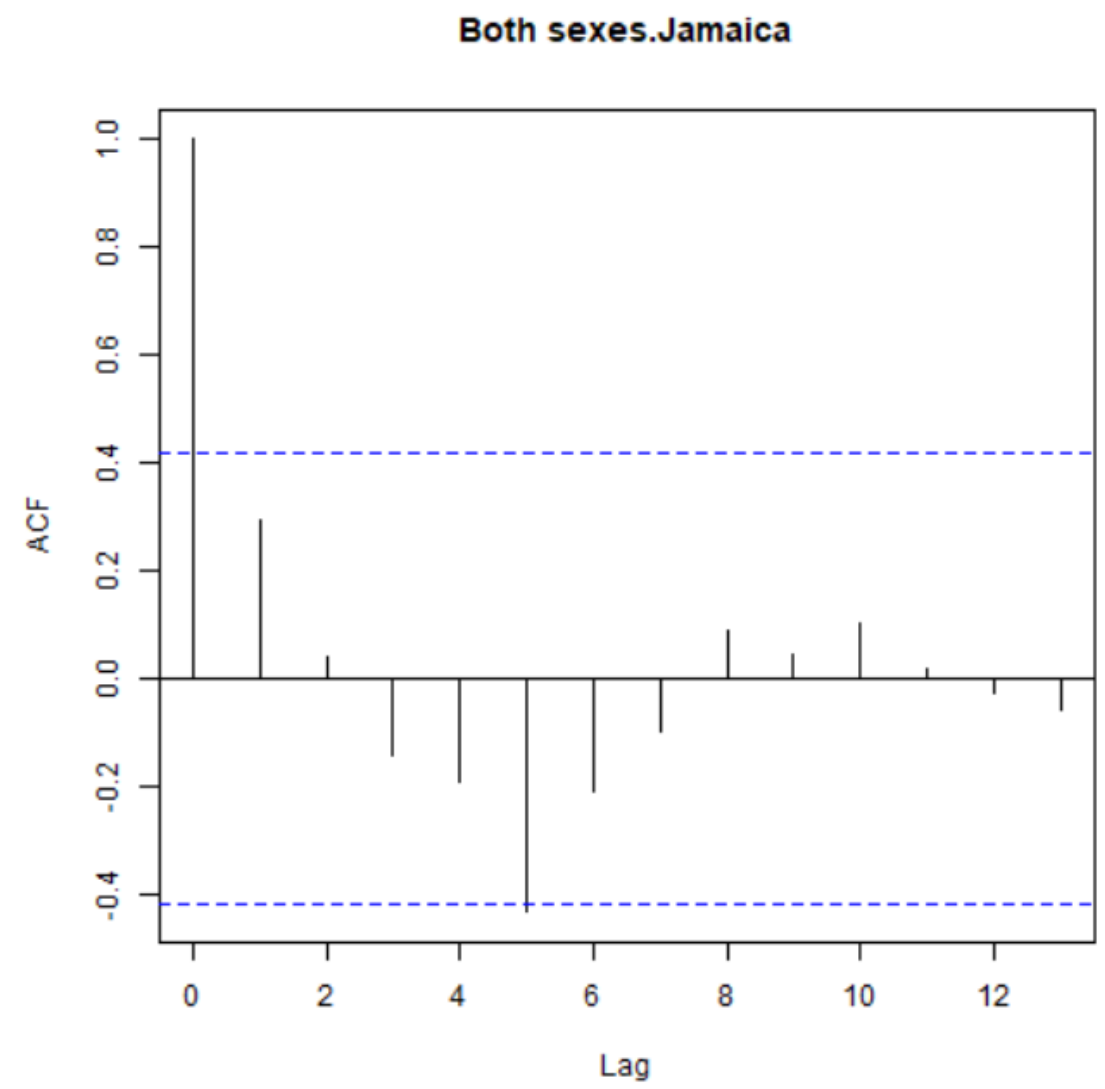
Both sexes.Jamaica
ACF
Lag

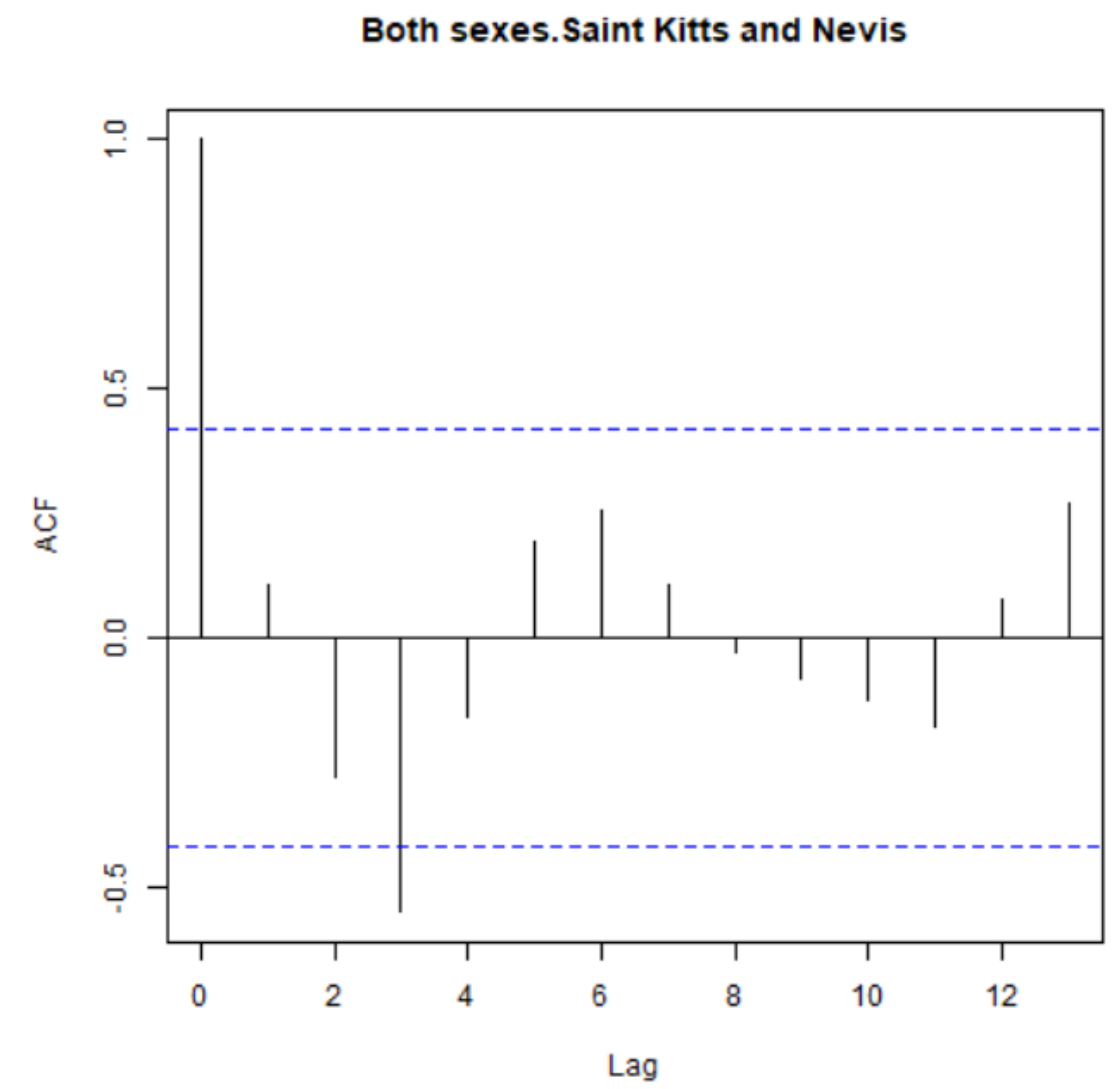
Both sexes.Saint Kitts and Nevis
ACF
Lag

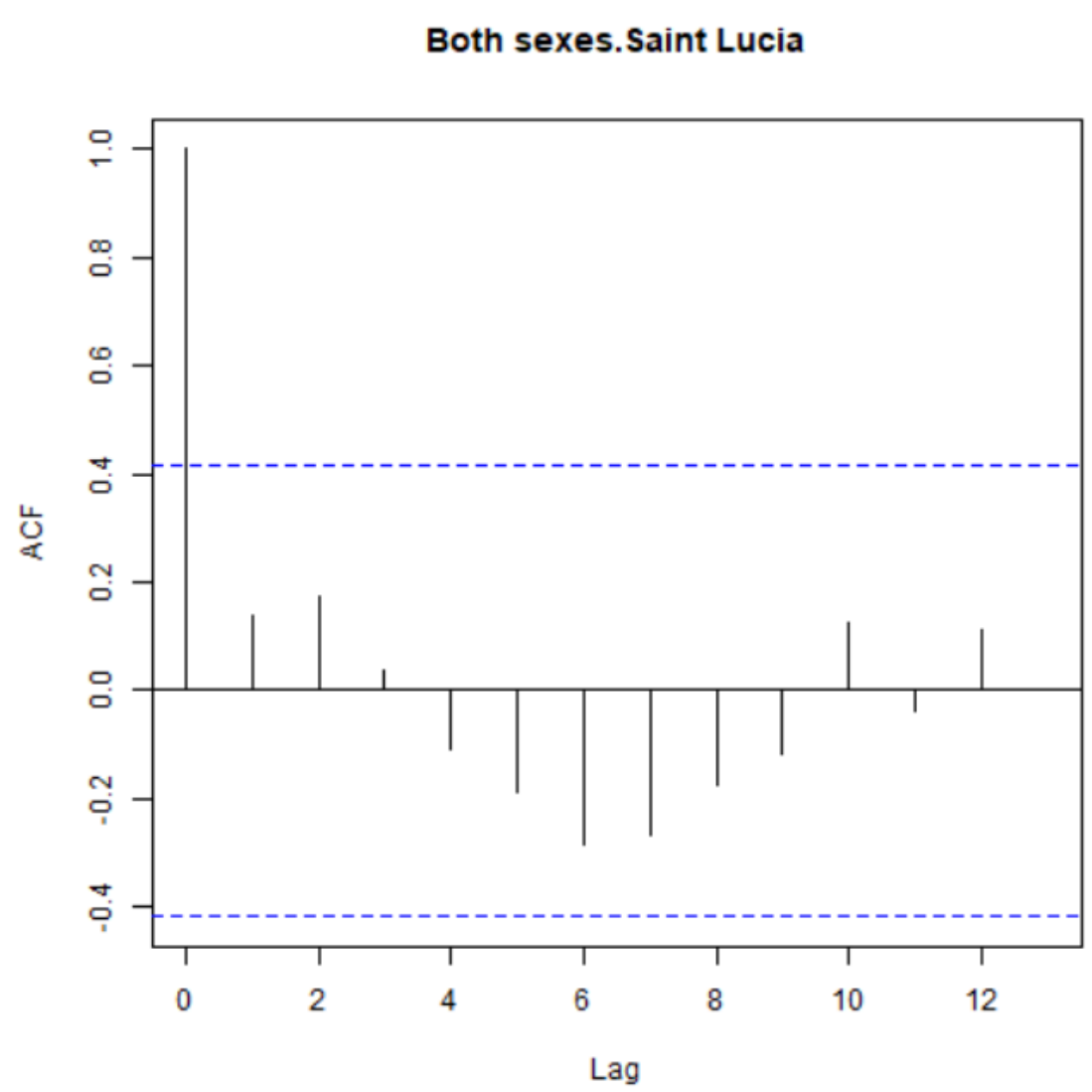
Both sexes.Saint Lucia
ACF
Lag

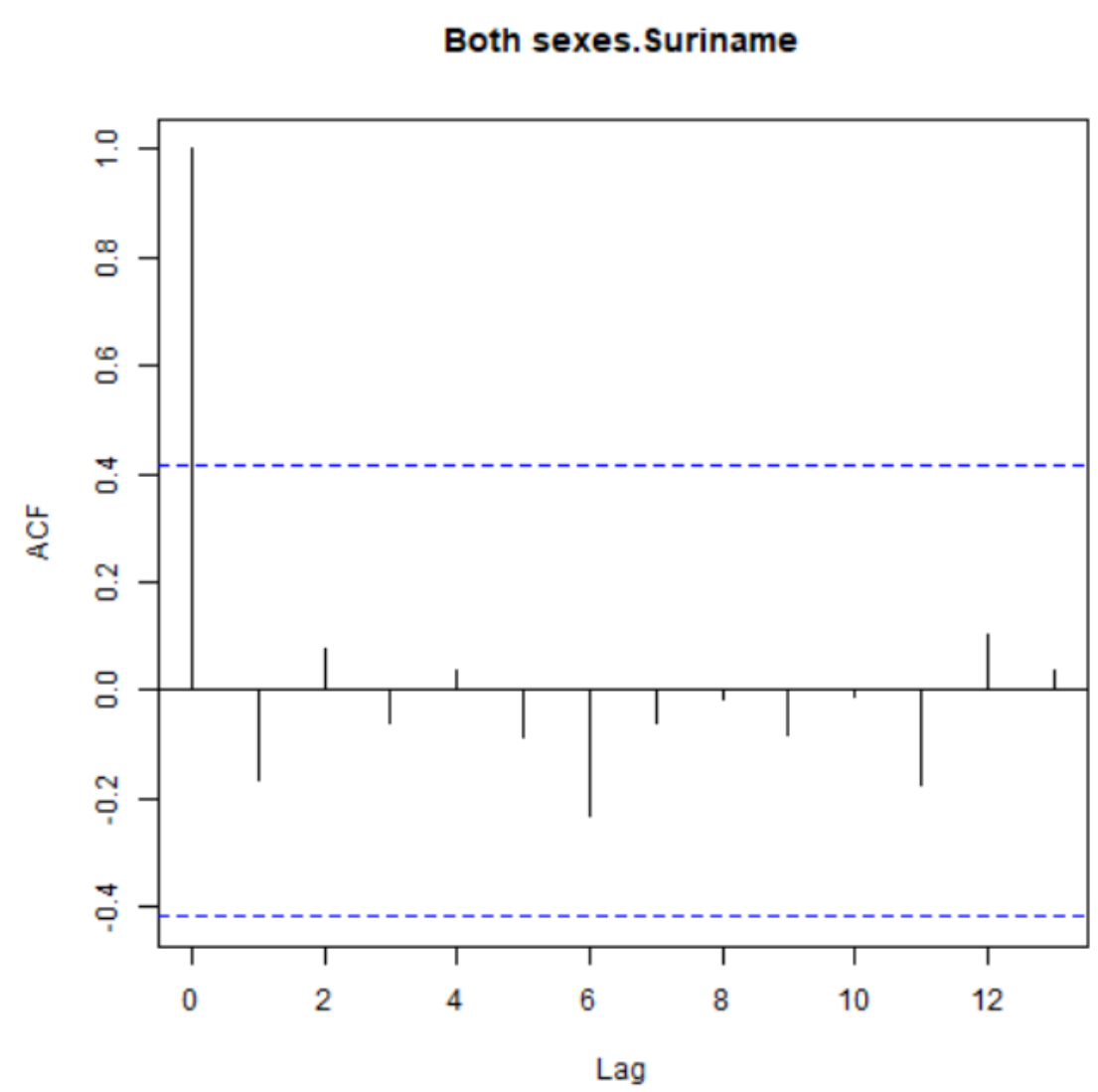
Both sexes.Suriname
ACF
Lag

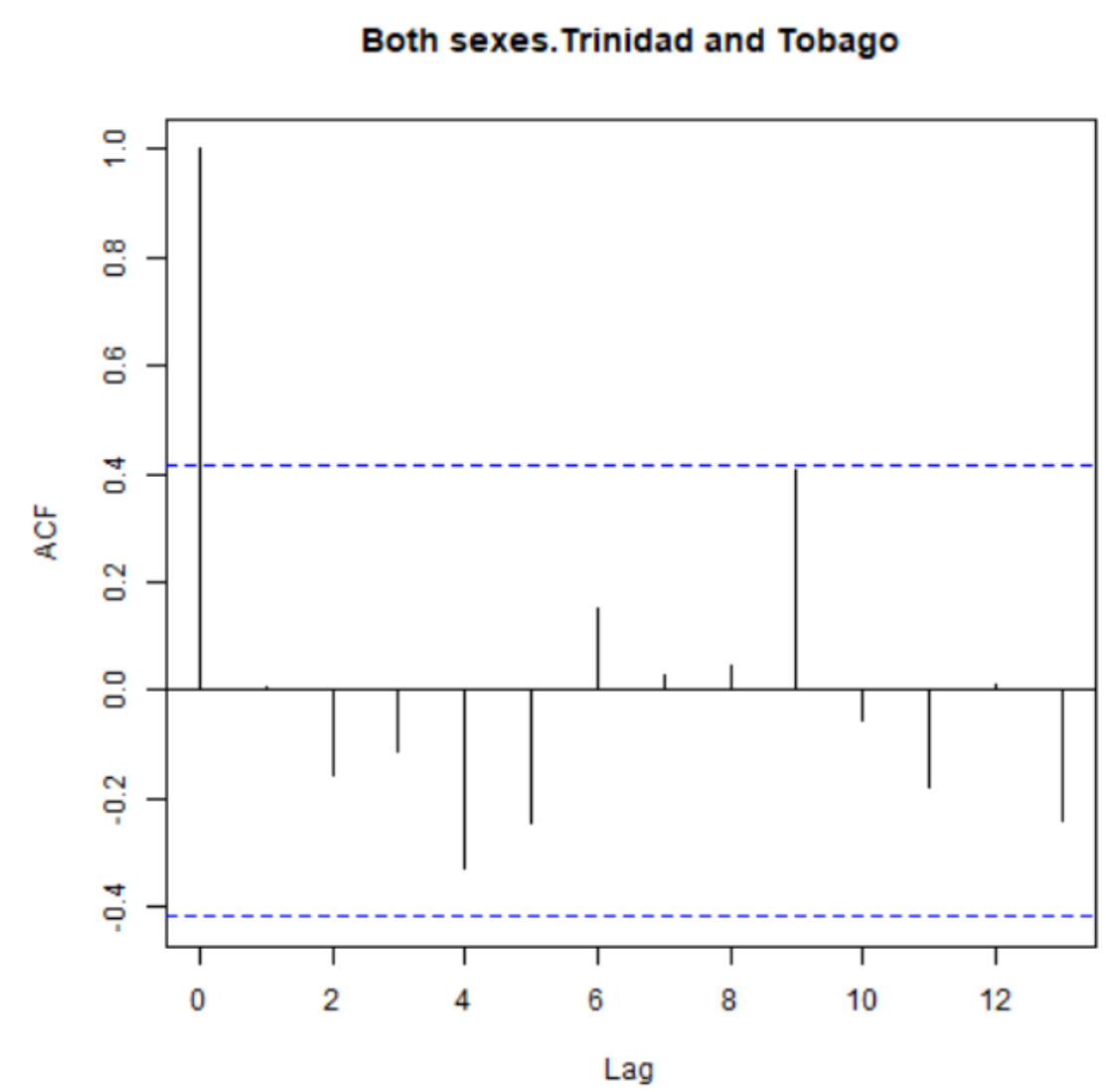
Both sexes.Trinidad and Tobago
ACF
Lag

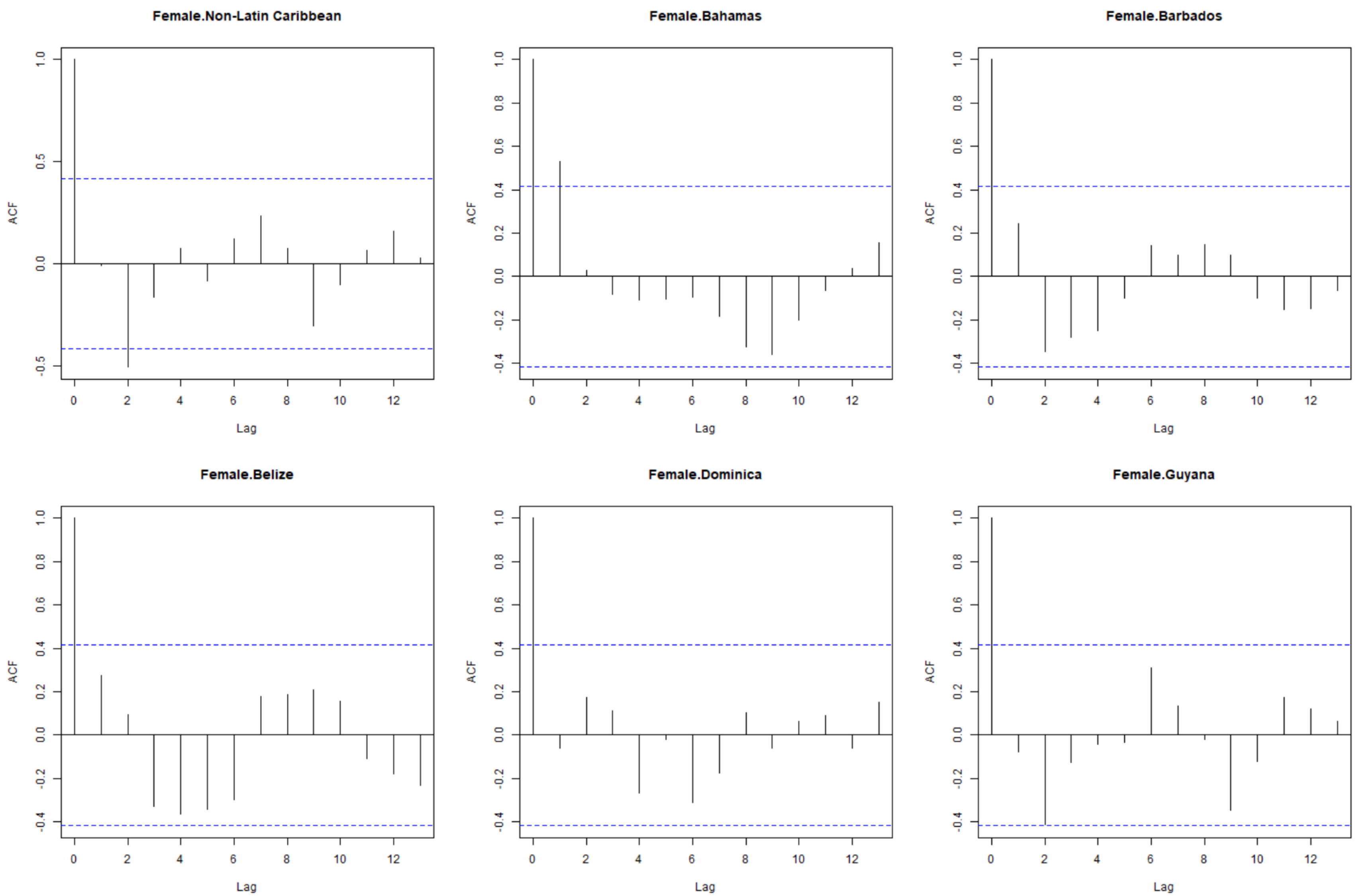
Female.Non-Latin Caribbean
Female.Bahamas
Female.Barbados
Female.Belize
Female.Dominica
Female.Guyana
ACF
Lag

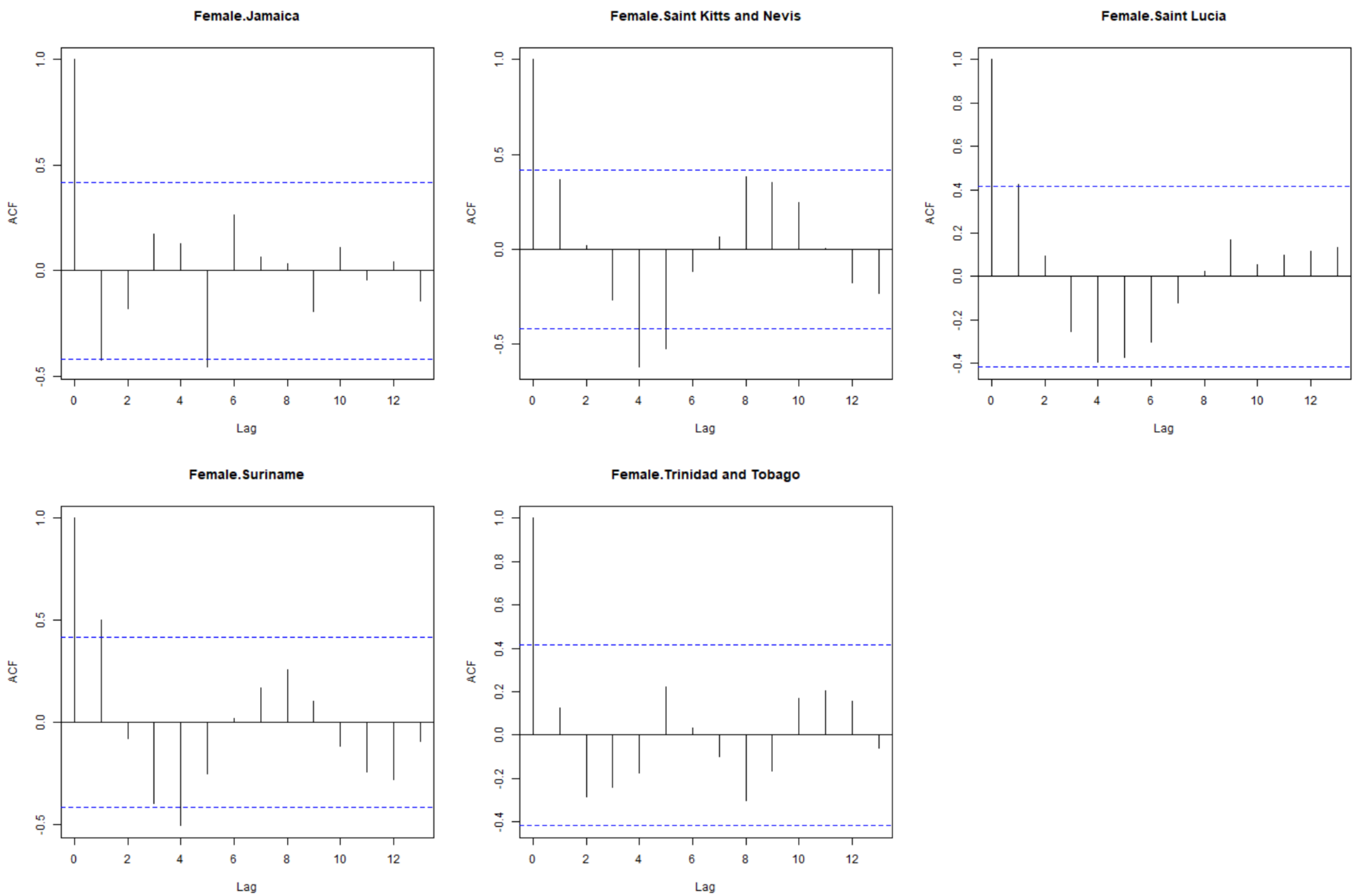
Female.Jamaica
Female.Saint Kitts and Nevis
Female.Saint Lucia
Female.Suriname
Female.Trinidad and Tobago
ACF
Lag

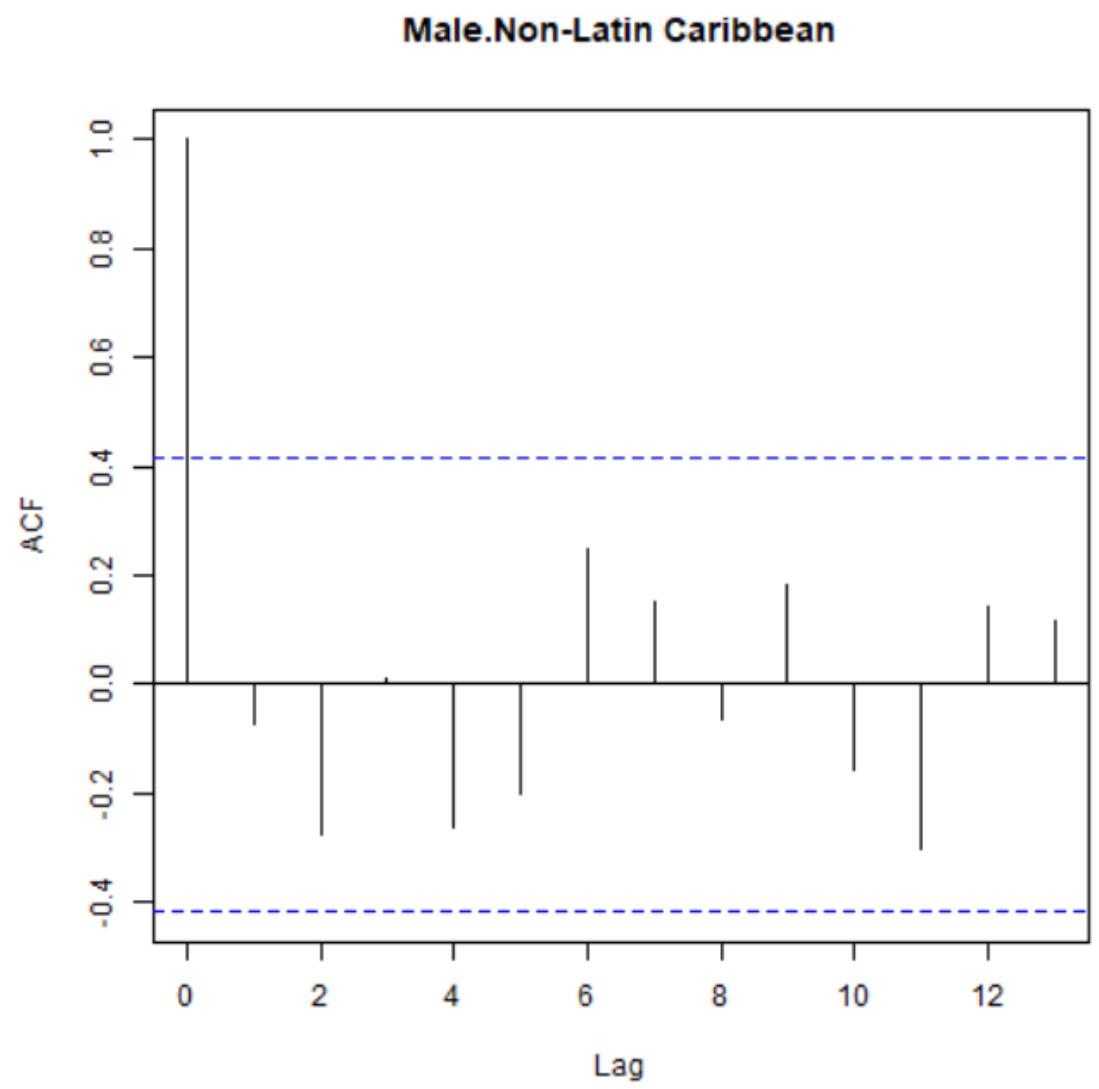
Male.Non-Latin Caribbean
ACF
Lag
-0.4 -0.2 0.0 0.2 0.4 0.6 0.8 1.0
0 2 4 6 8 10 12

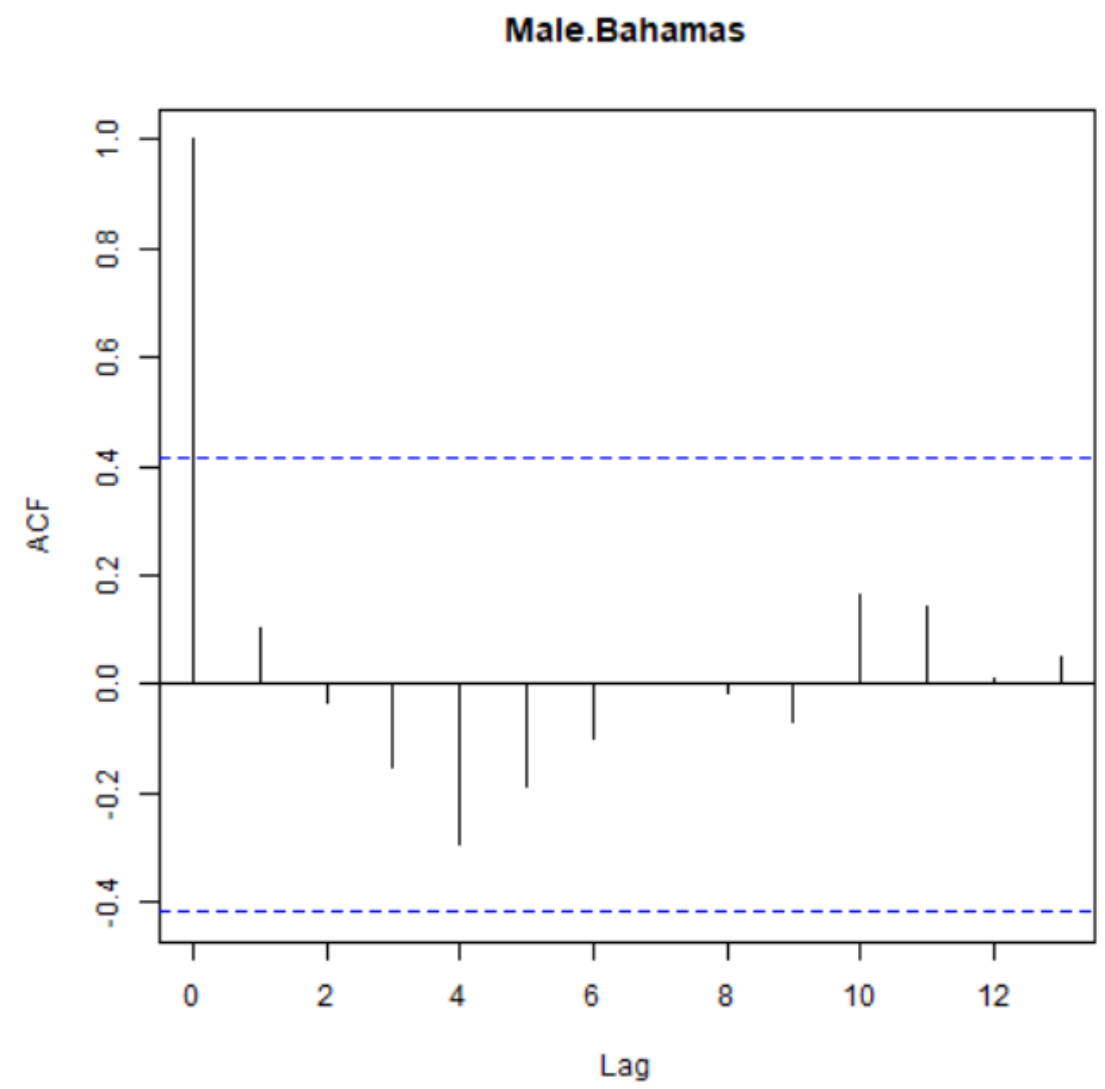
Male.Bahamas
ACF
Lag
-0.4 -0.2 0.0 0.2 0.4 0.6 0.8 1.0
0 2 4 6 8 10 12

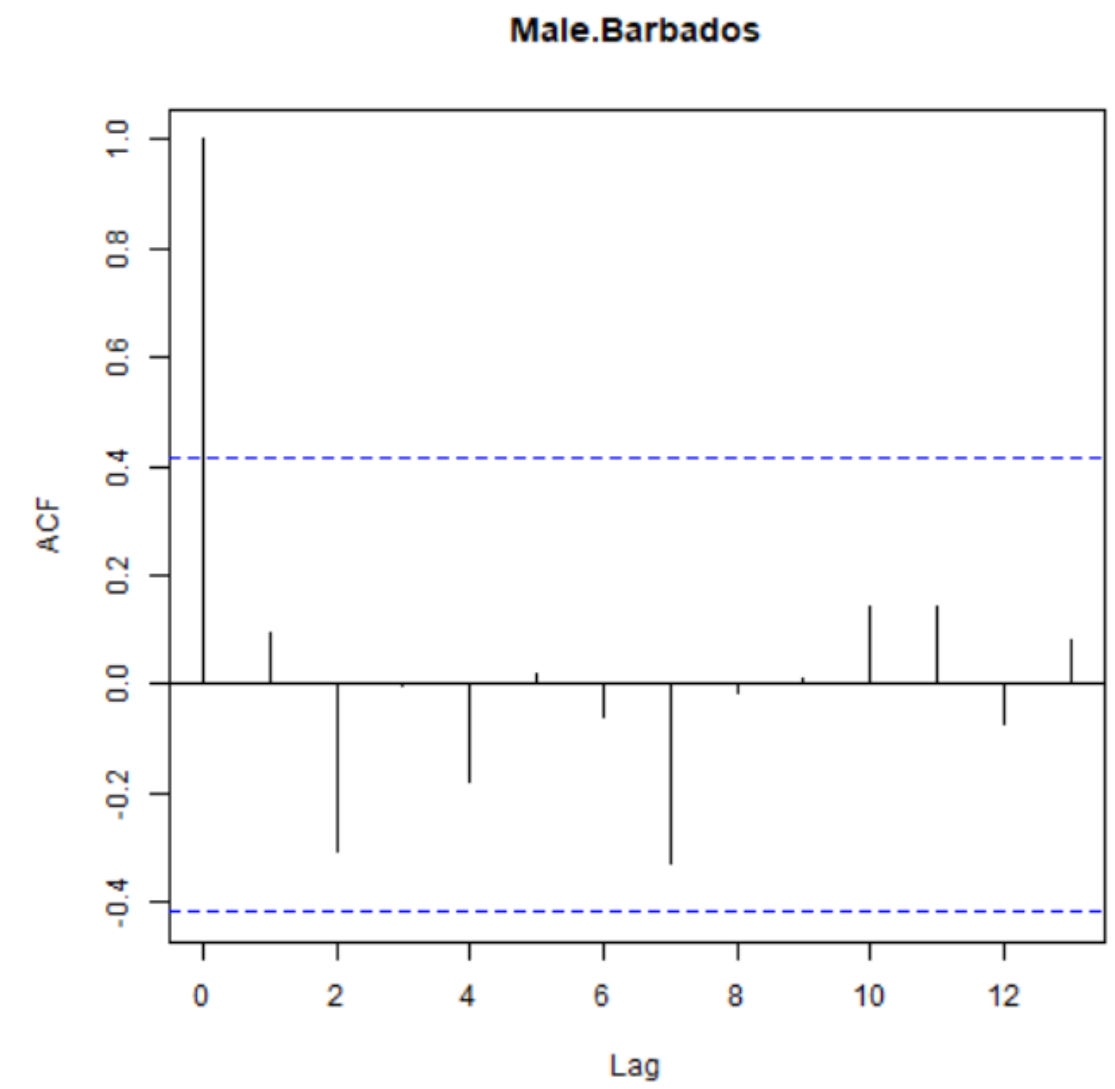
Male.Barbados
ACF
Lag
-0.4 -0.2 0.0 0.2 0.4 0.6 0.8 1.0
0 2 4 6 8 10 12

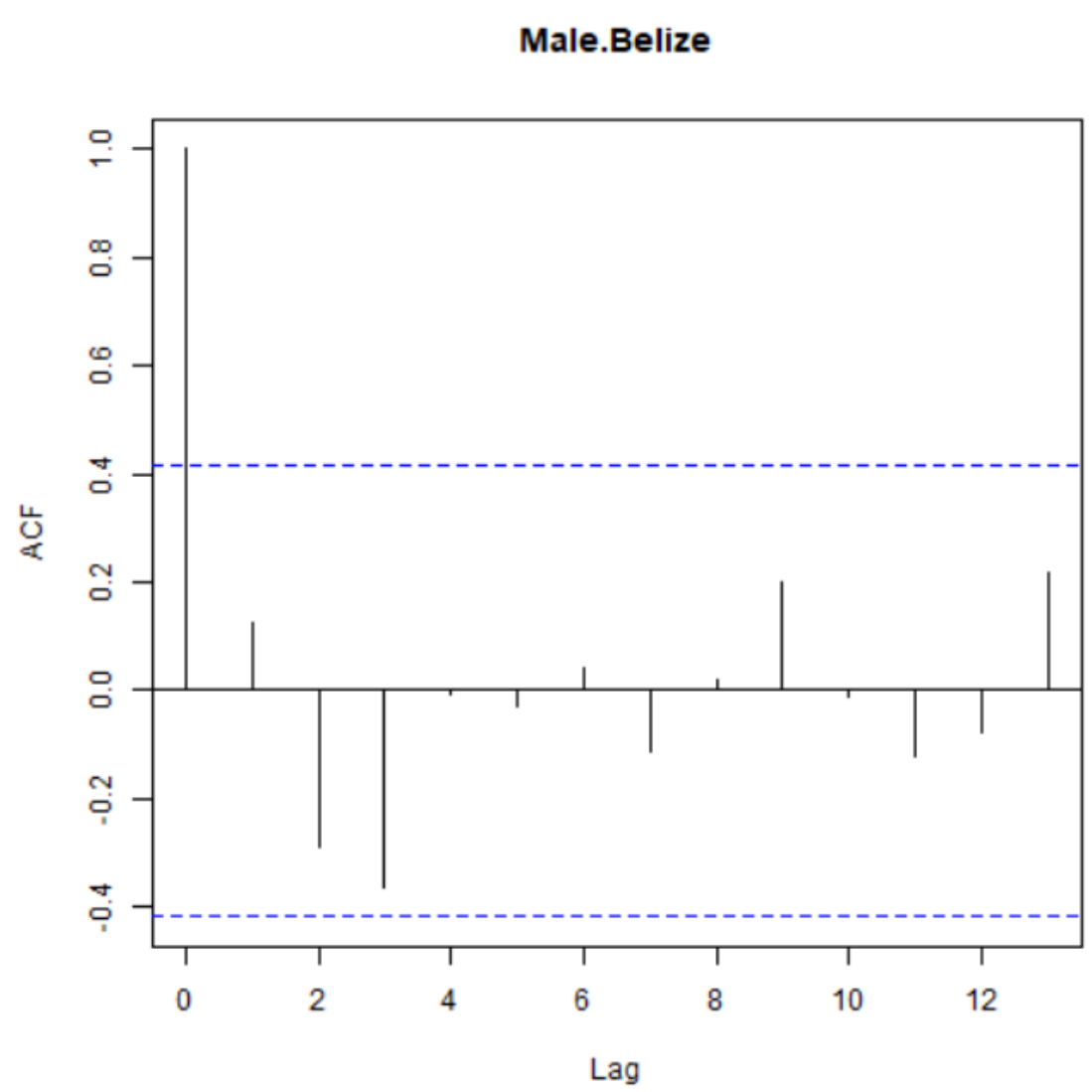
Male.Belize
ACF
Lag
-0.4 -0.2 0.0 0.2 0.4 0.6 0.8 1.0
0 2 4 6 8 10 12

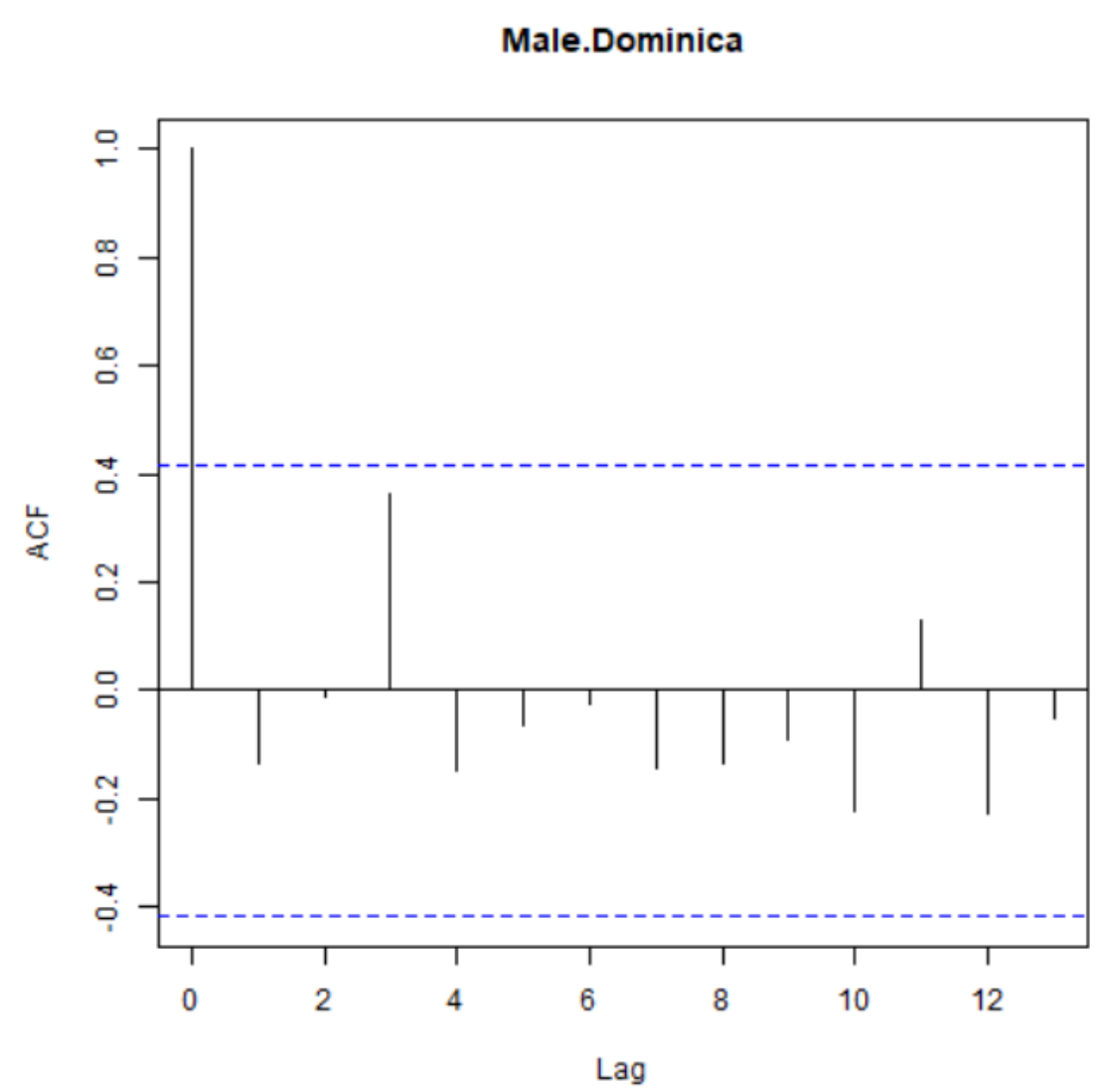
Male.Dominica
ACF
Lag
-0.4 -0.2 0.0 0.2 0.4 0.6 0.8 1.0
0 2 4 6 8 10 12

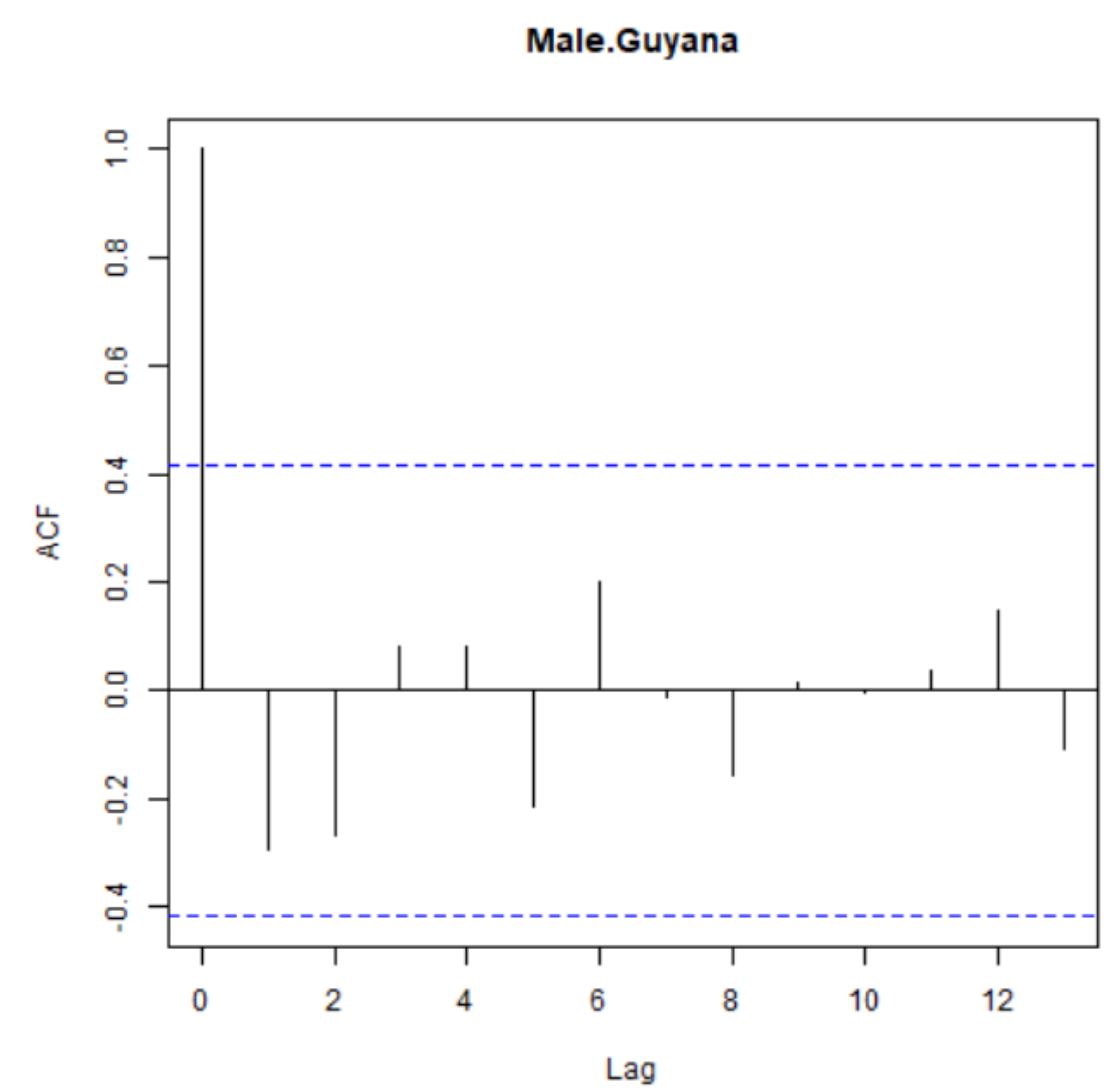
Male.Guyana
ACF
Lag
-0.4 -0.2 0.0 0.2 0.4 0.6 0.8 1.0
0 2 4 6 8 10 12

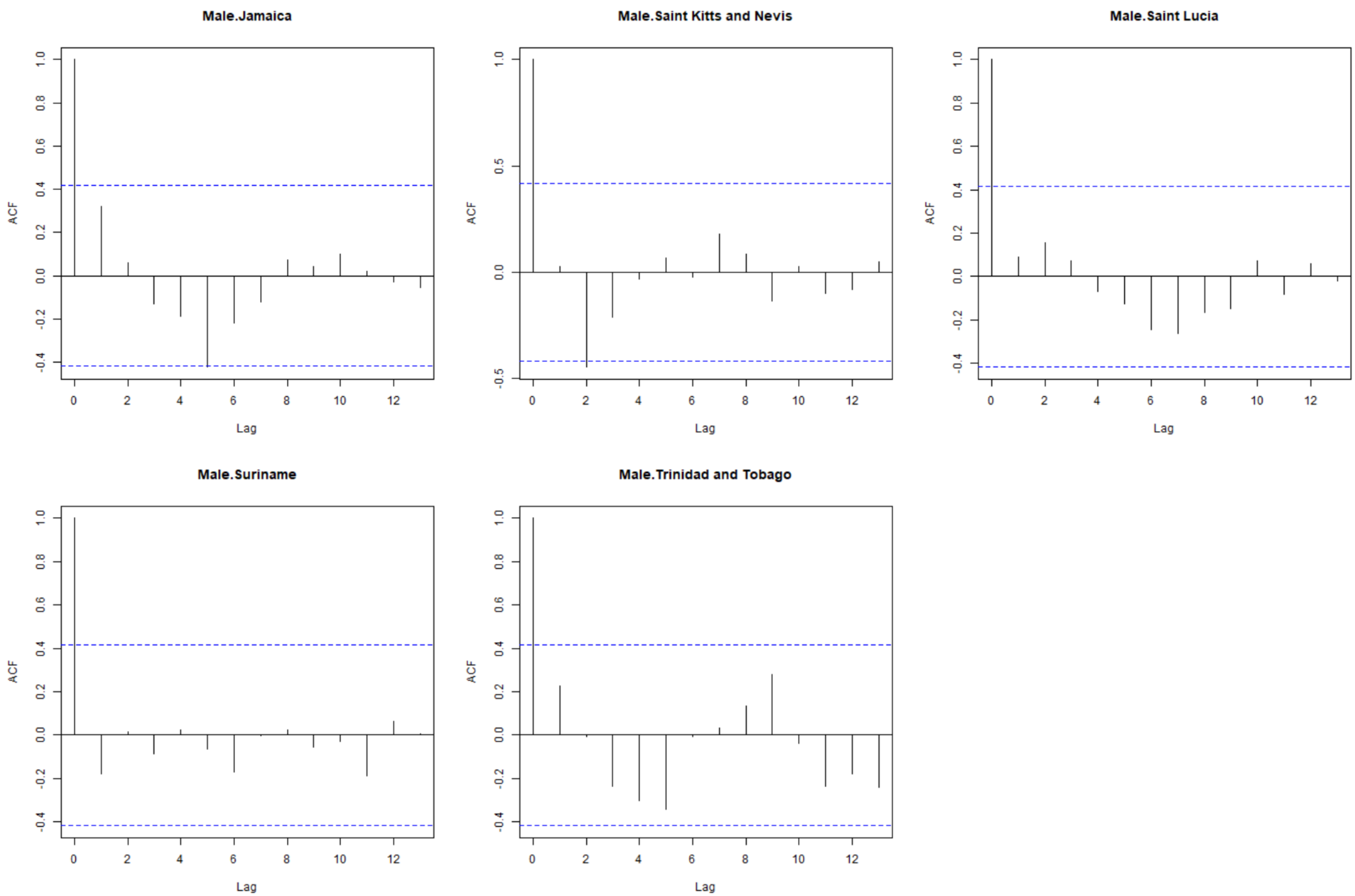

Male.Jamaica
Male.Saint Kitts and Nevis
Male.Saint Lucia
Male.Suriname
Male.Trinidad and Tobago
ACF
Lag

(D)

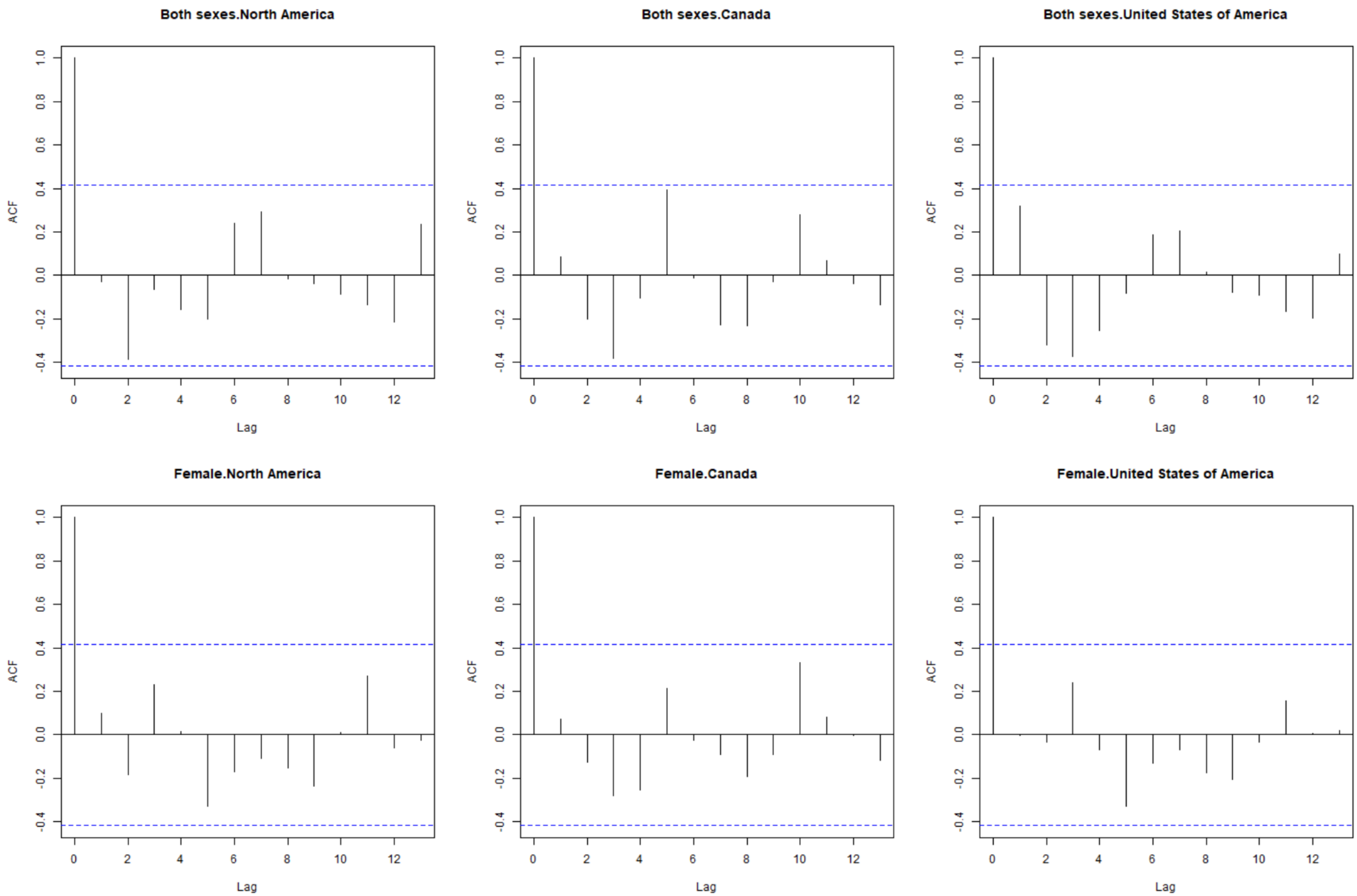
Both sexes.North America
Both sexes.Canada
Both sexes.United States of America
Female.North America
Female.Canada
Female.United States of America
ACF
Lag
1.0
0.8
0.6
0.4
0.2
0.0
-0.2
-0.4
0
2
4
6
8
10
12

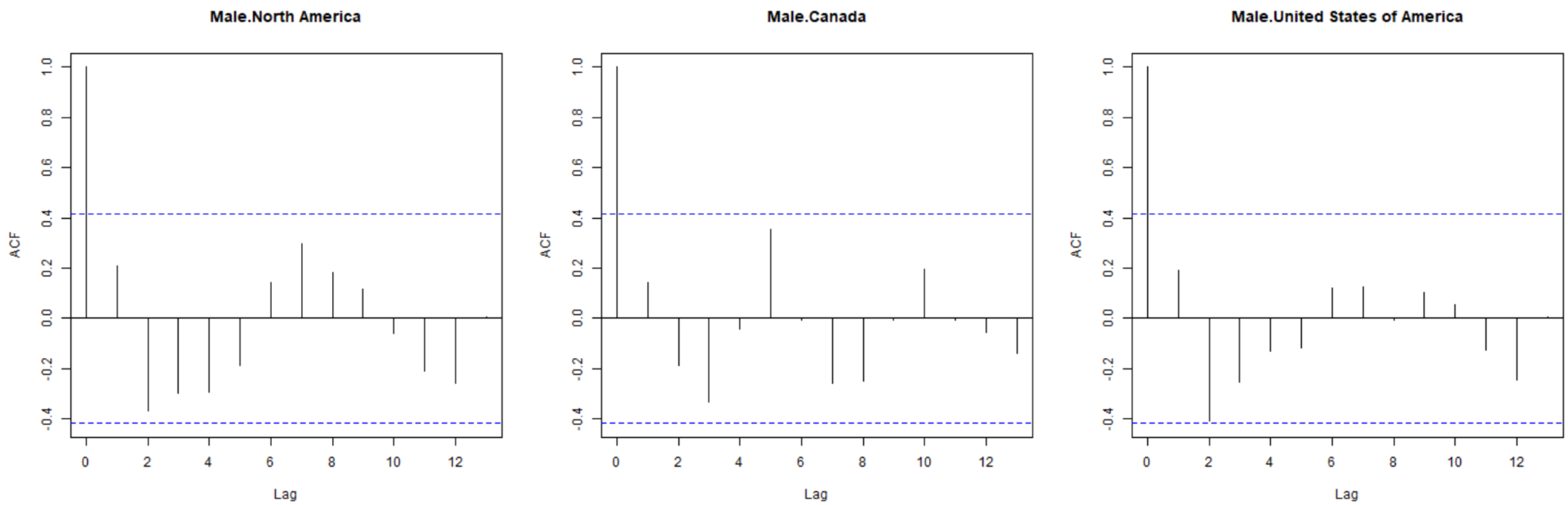

Male.North America
Male.Canada
Male.United States of America
ACF
Lag

(E)

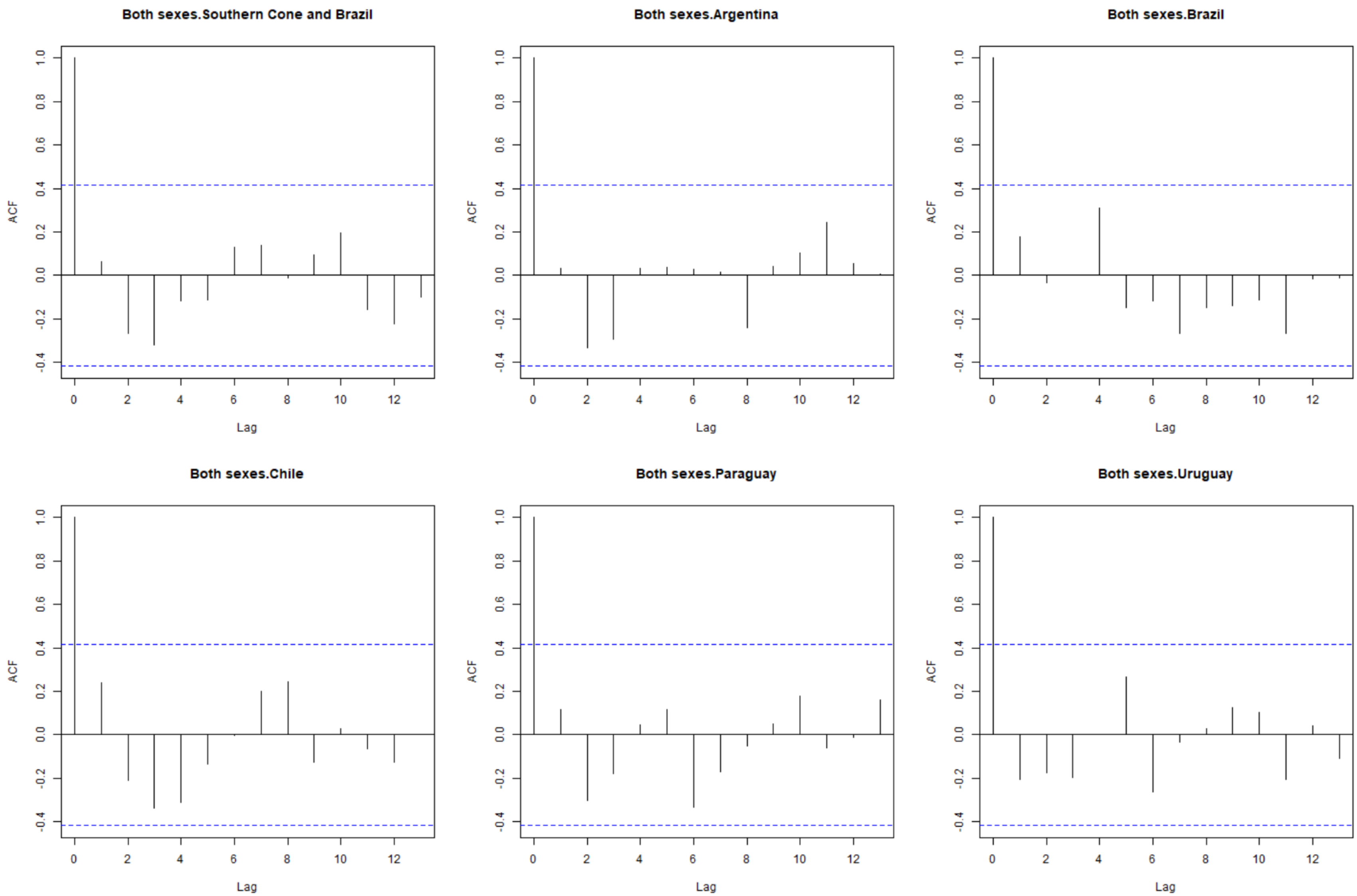

Both sexes.Southern Cone and Brazil
Both sexes.Argentina
Both sexes.Brazil
Both sexes.Chile
Both sexes.Paraguay
Both sexes.Uruguay
ACF
Lag

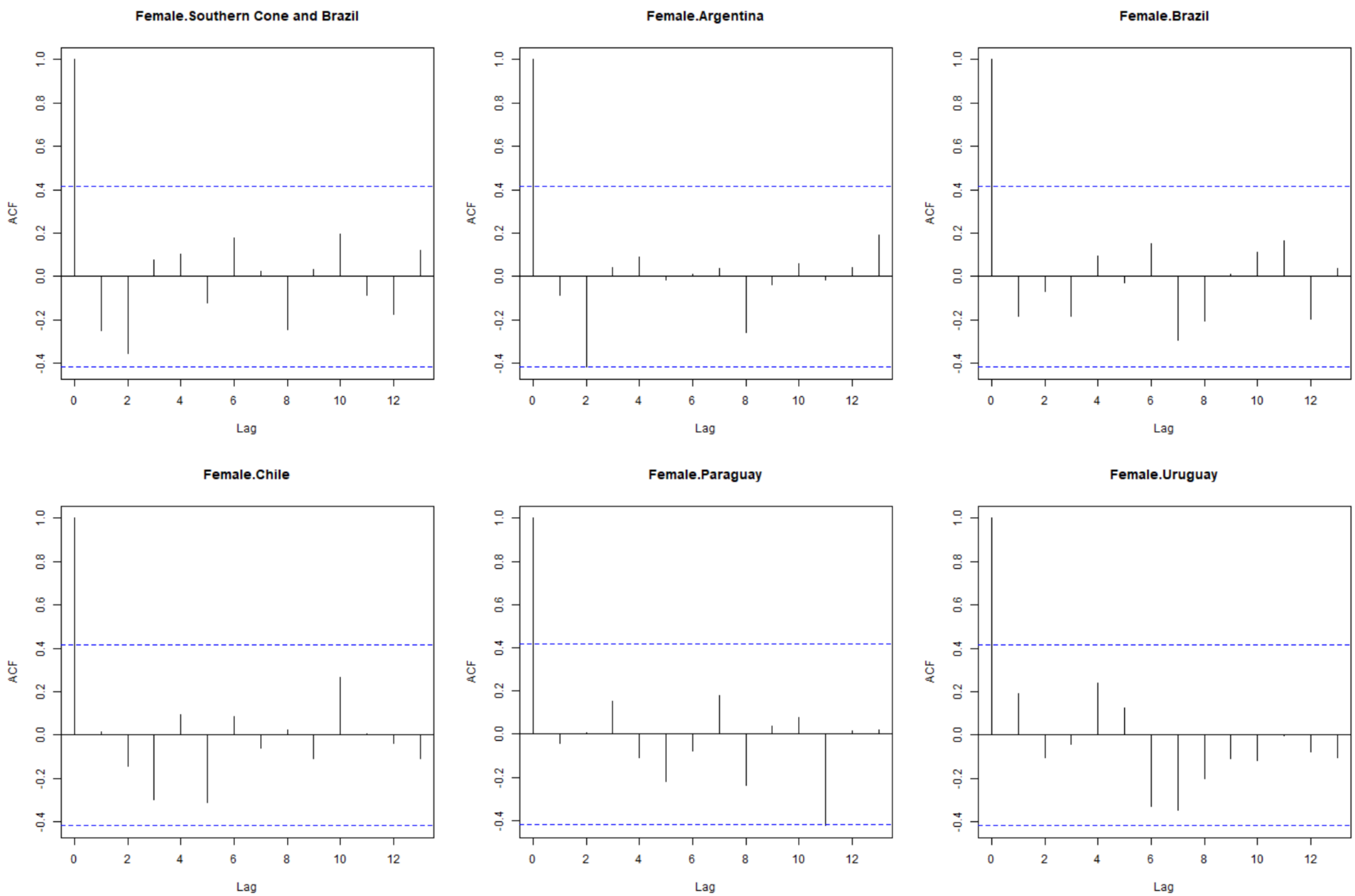
Female.Southern Cone and Brazil
Female.Argentina
Female.Brazil
Female.Chile
Female.Paraguay
Female.Uruguay
ACF
Lag
1.0
0.8
0.6
0.4
0.2
0.0
-0.2
-0.4
0
2
4
6
8
10
12

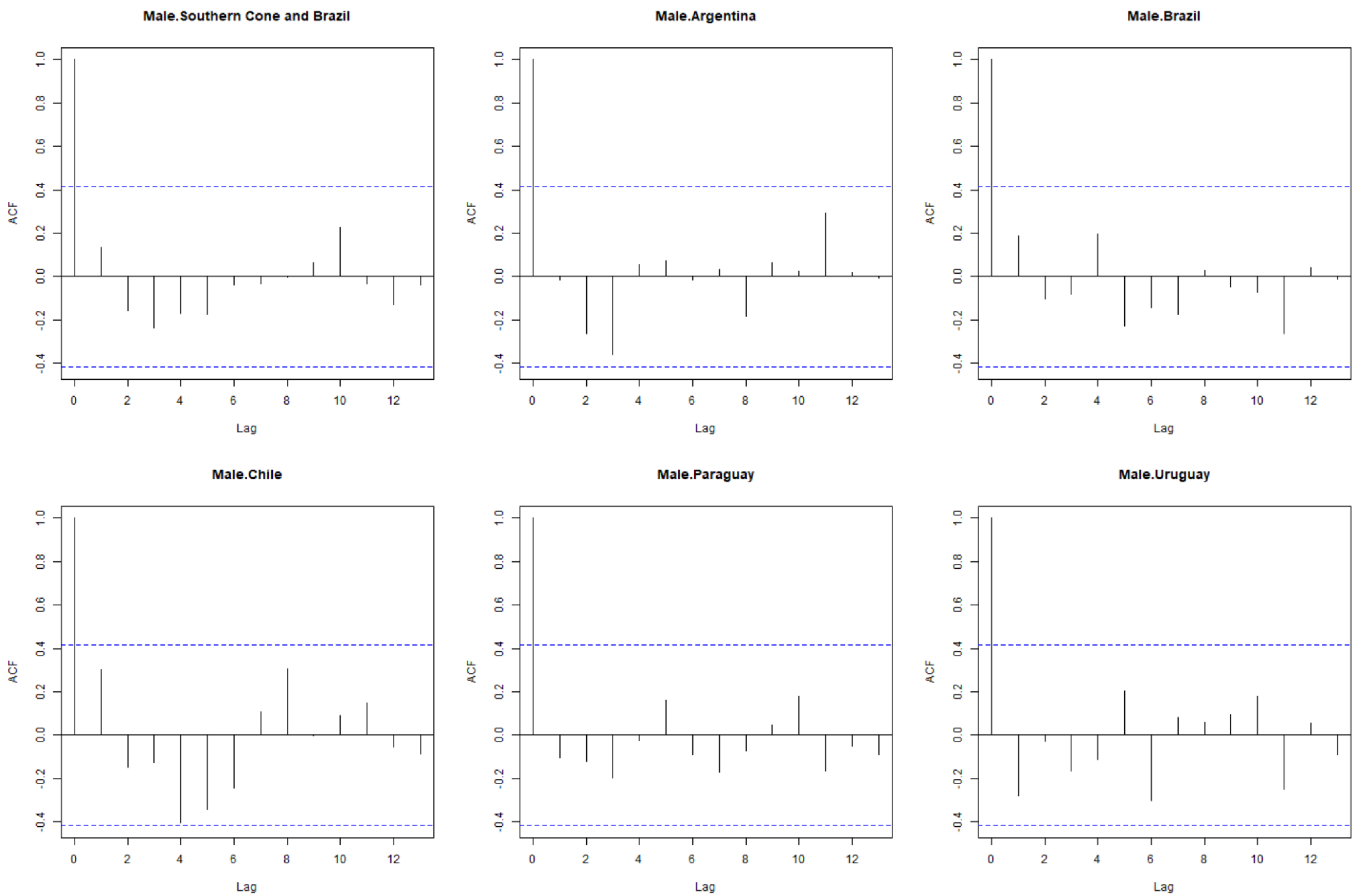
Male.Southern Cone and Brazil
Male.Argentina
Male.Brazil
Male.Chile
Male.Paraguay
Male.Uruguay
ACF
Lag
-0.4
-0.2
0.0
0.2
0.4
0.6
0.8
1.0
0
2
4
6
8
10
12

(F)

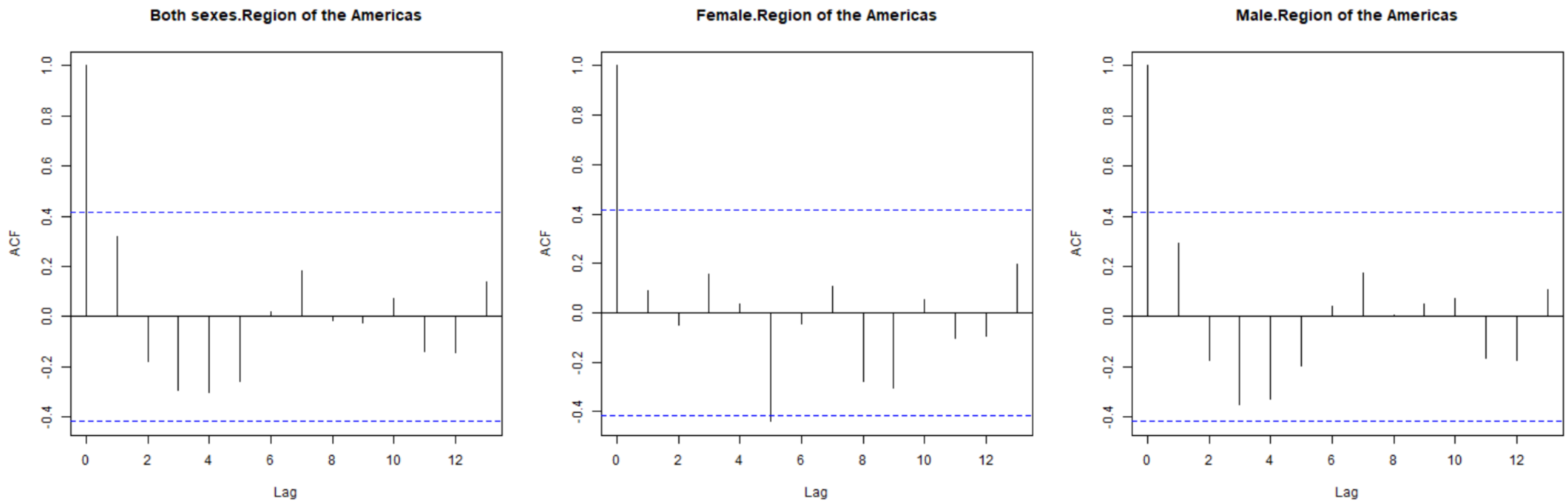
Both sexes.Region of the Americas
Female.Region of the Americas
Male.Region of the Americas
ACF
Lag
1.0
0.8
0.6
0.4
0.2
0.0
-0.2
-0.4
0
2
4
6
8
10
12

Figure S3. Suicide mortality rate trends for (A) the Andean Area, (B) Central America, Mexico and Latin Caribbean, (C) Non-Latin Caribbean, (D) North America, (E) Southern Cone and Brazil and (F) the Region of the Americas by country

(A)

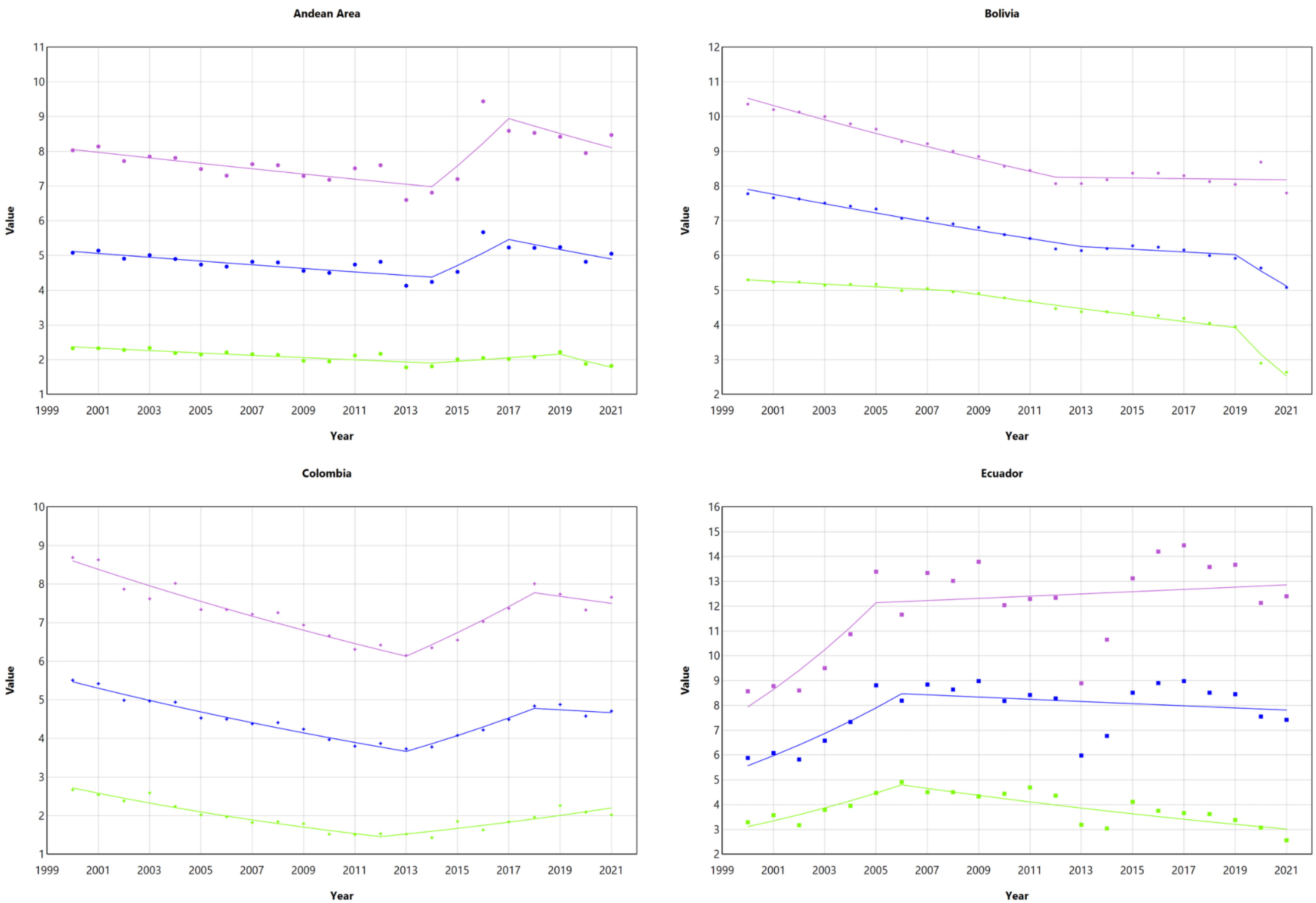

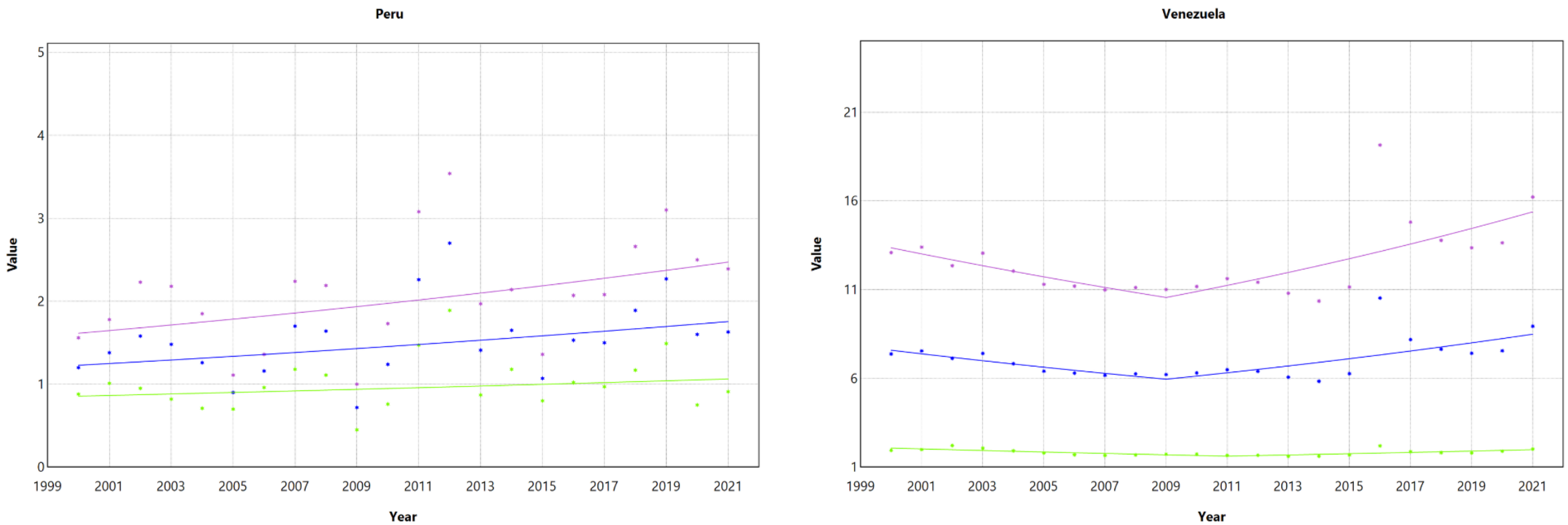
Peru
Value
Year
Venezuela
Value
Year

(B)

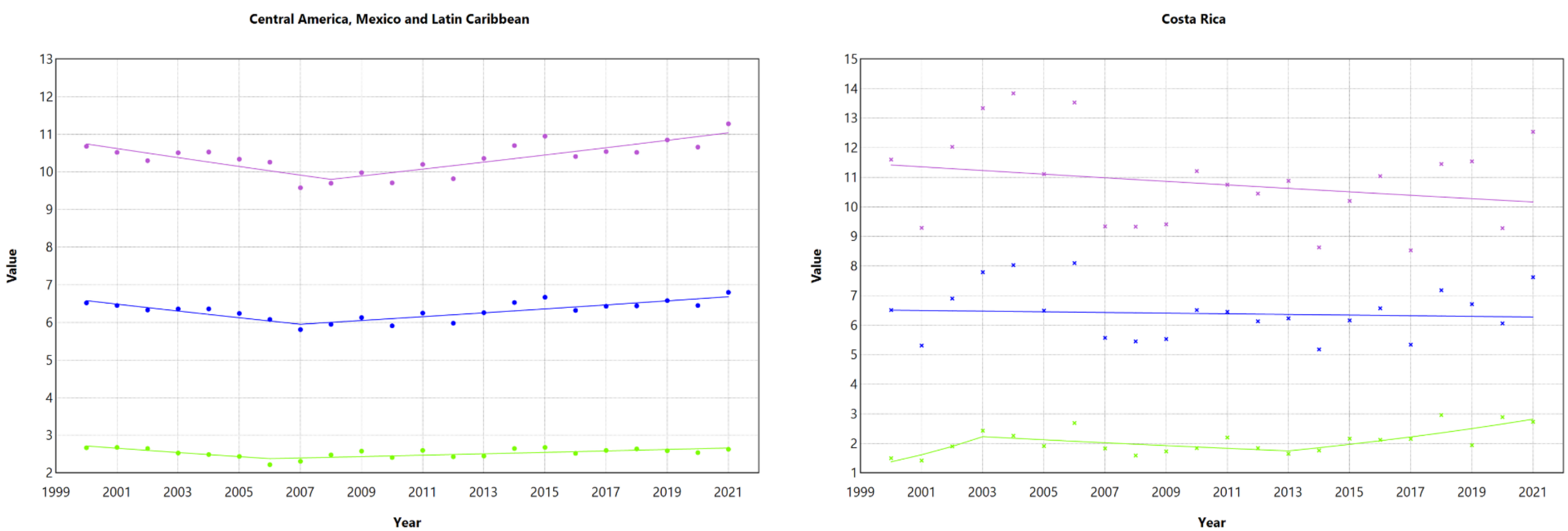
Central America, Mexico and Latin Caribbean
Value
Year
Costa Rica
Value
Year

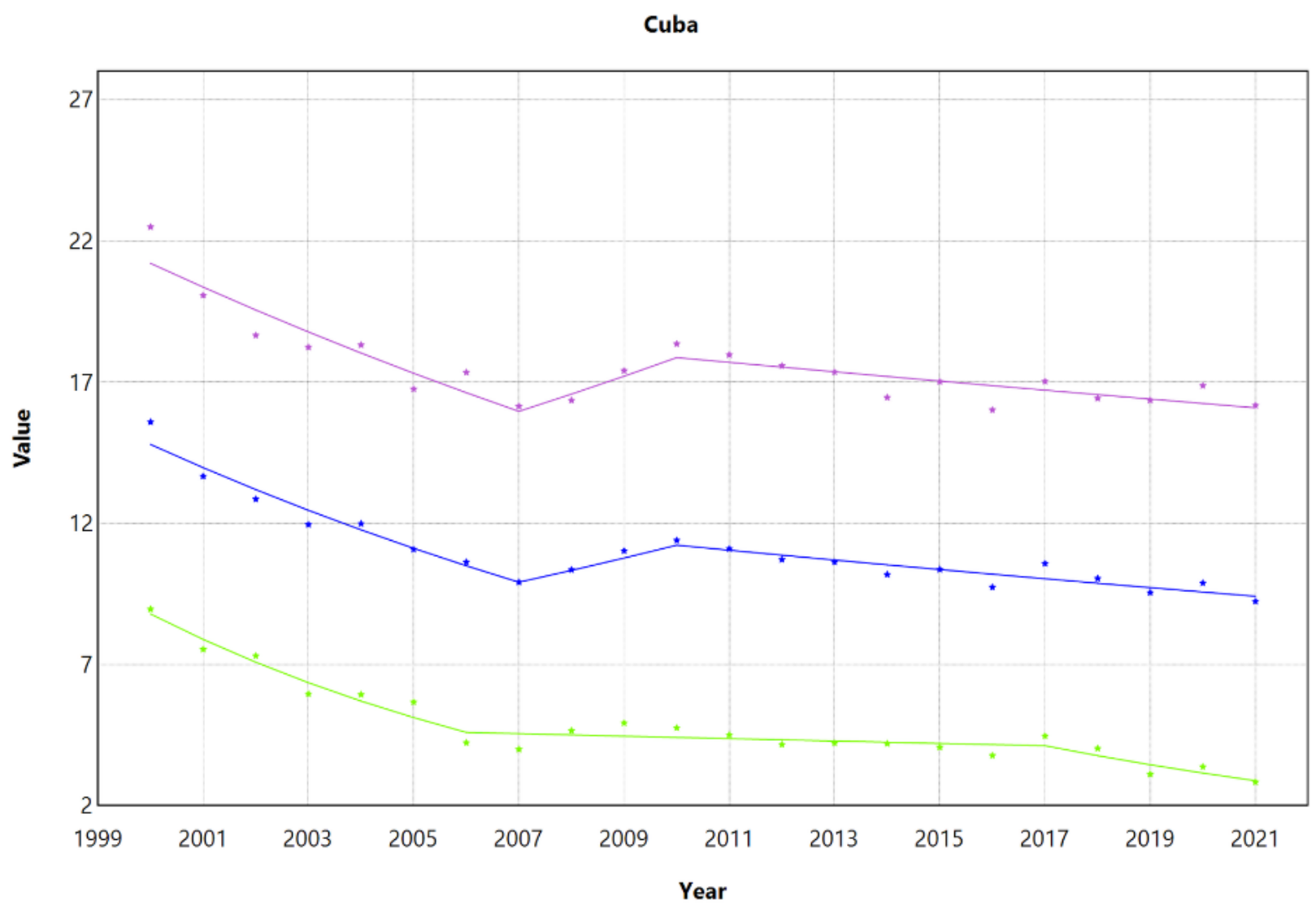
Cuba
Value
27
22
17
12
7
2
1999
2001
2003
2005
2007
2009
2011
2013
2015
2017
2019
2021
Year

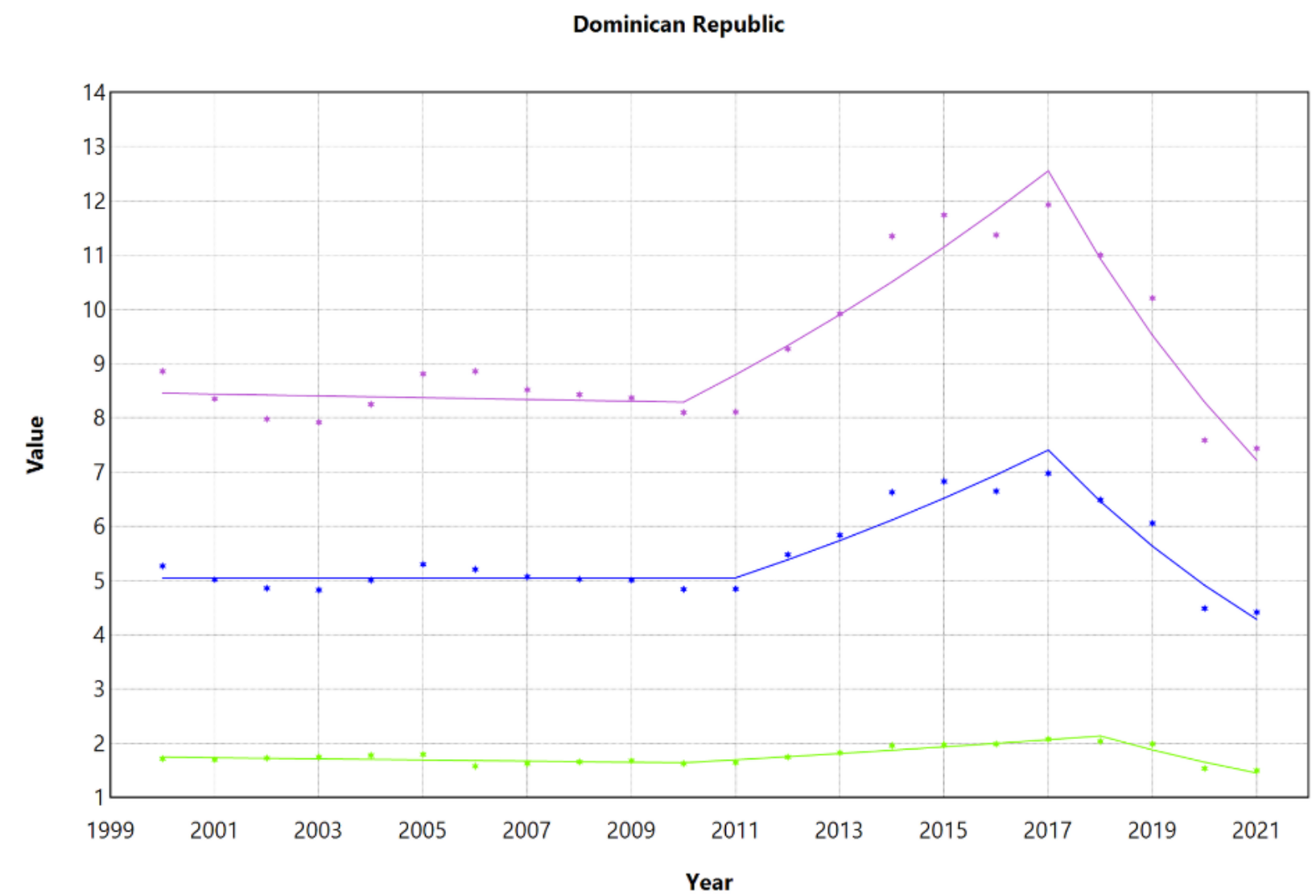
Dominican Republic
Value
14
13
12
11
10
9
8
7
6
5
4
3
2
1
1999
2001
2003
2005
2007
2009
2011
2013
2015
2017
2019
2021
Year

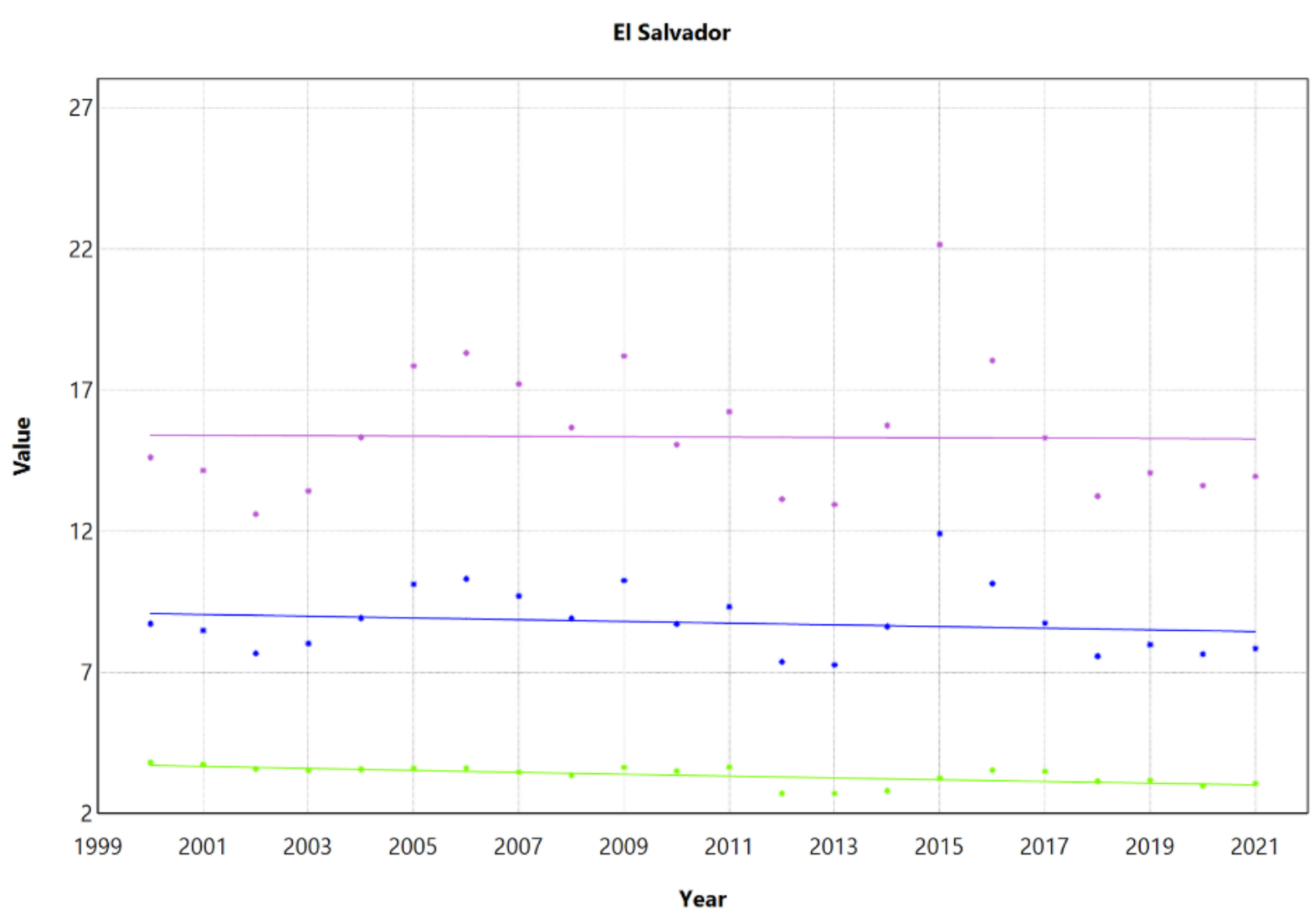
El Salvador
Value
27
22
17
12
7
2
1999
2001
2003
2005
2007
2009
2011
2013
2015
2017
2019
2021
Year

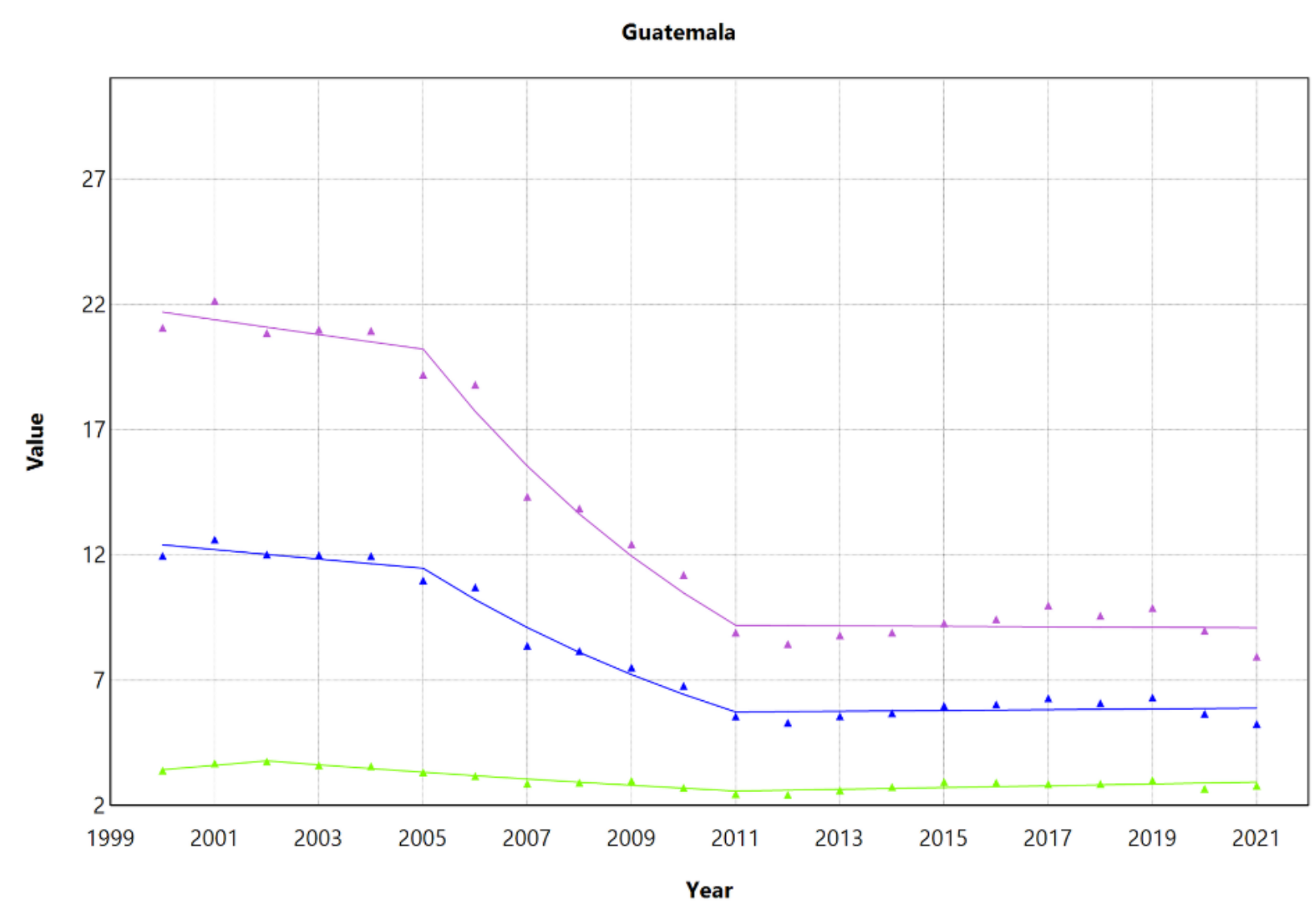
Guatemala
Value
27
22
17
12
7
2
1999
2001
2003
2005
2007
2009
2011
2013
2015
2017
2019
2021
Year

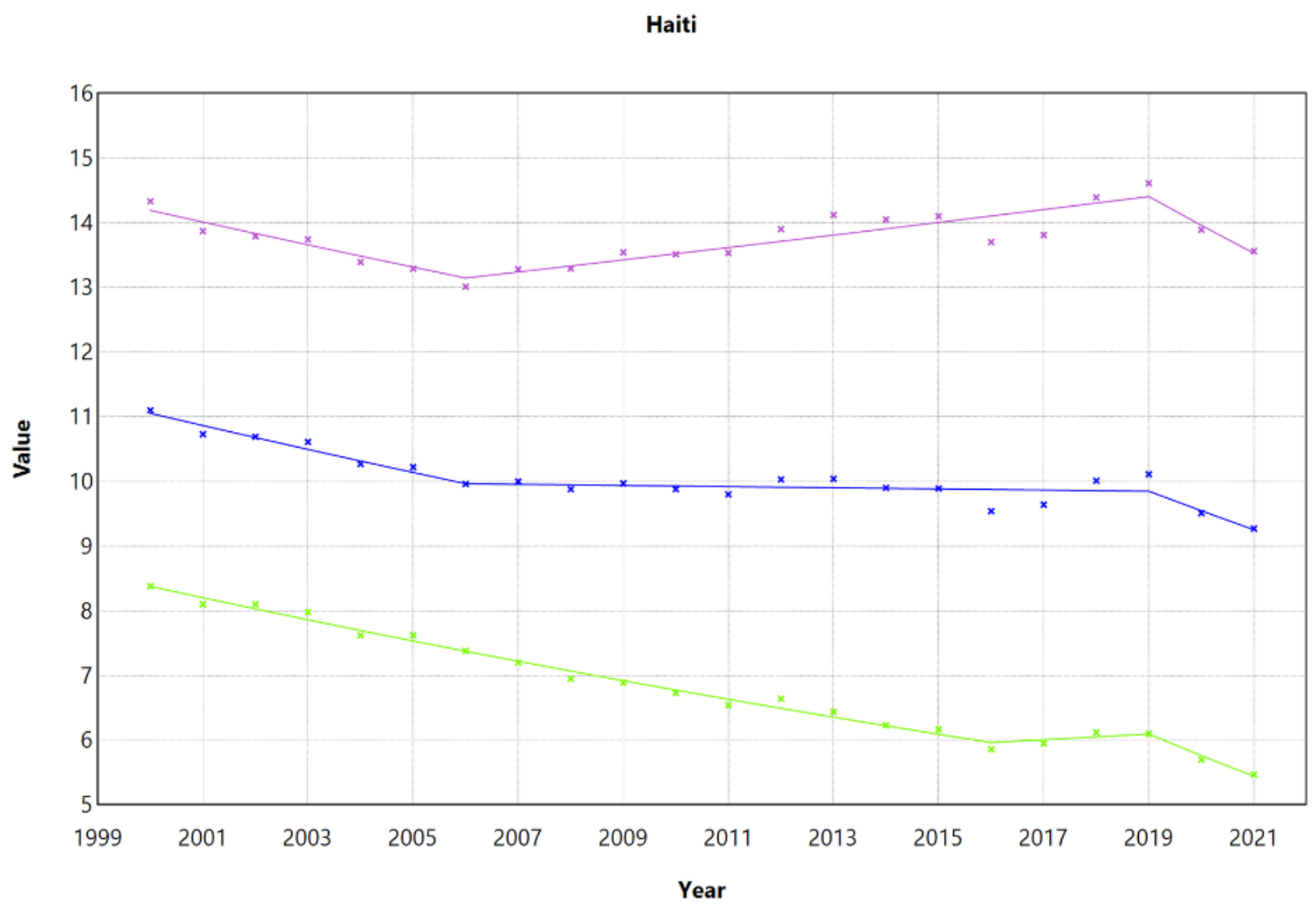
Haiti
Value
Year

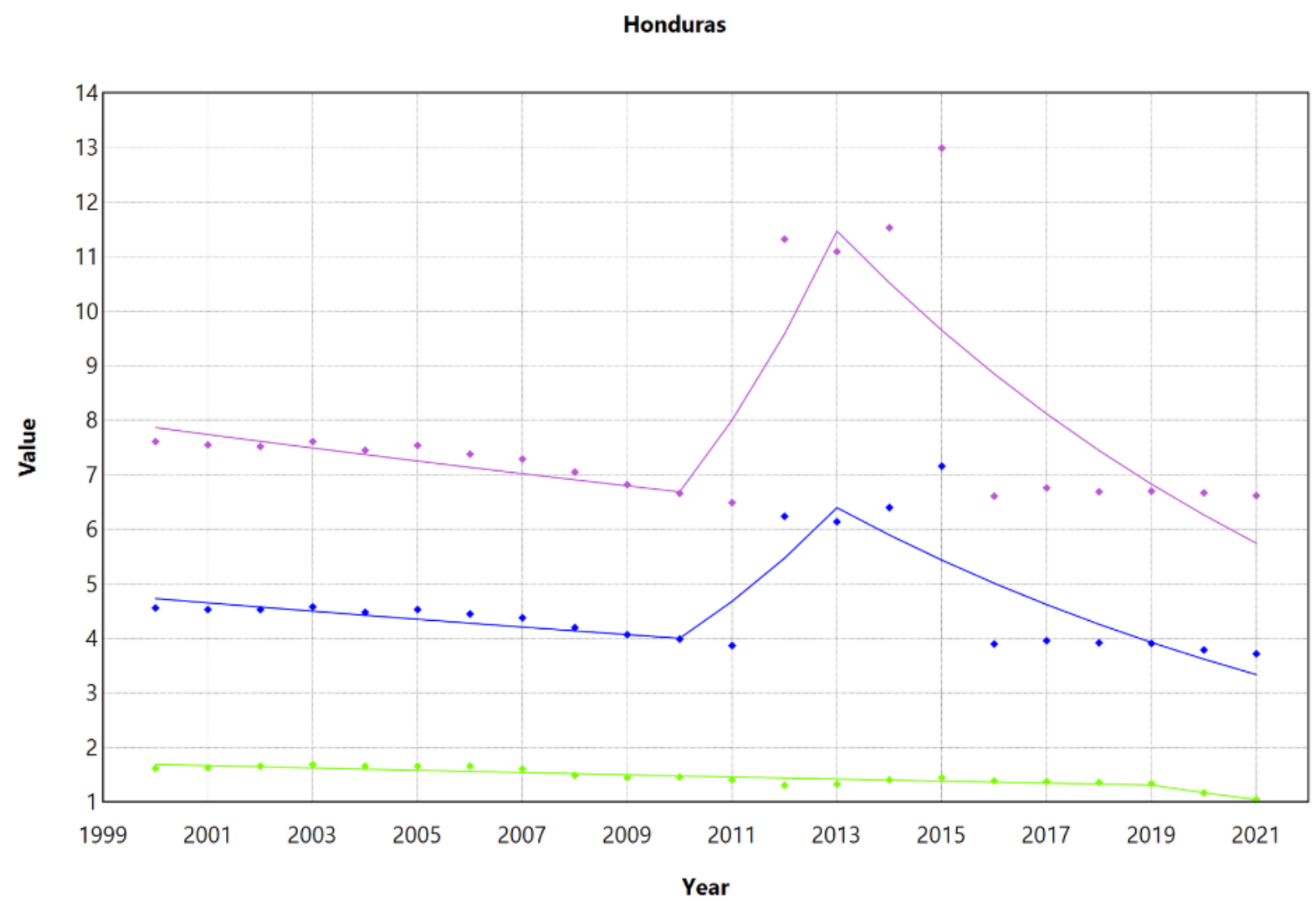
Honduras
Value
Year

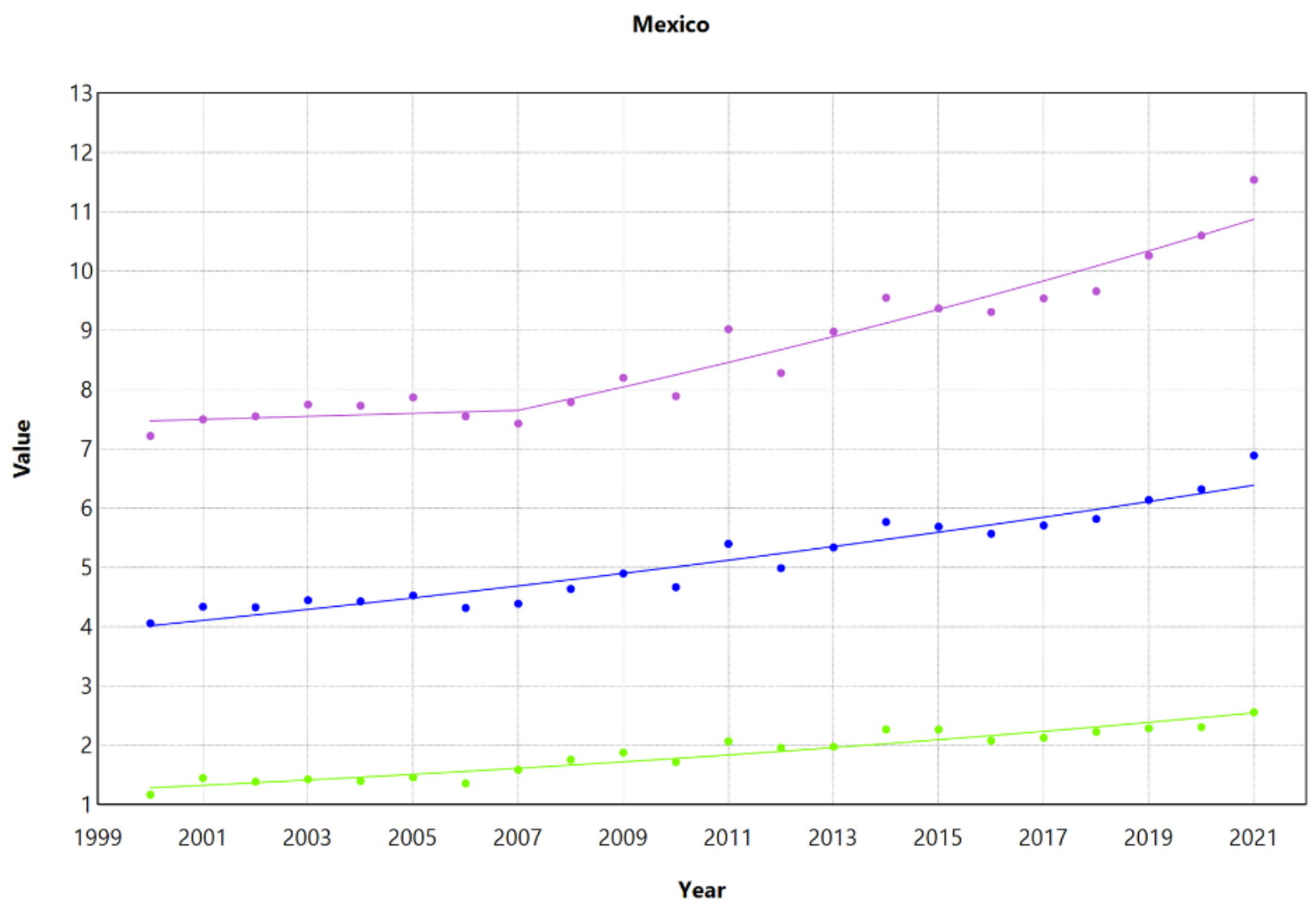
Mexico
Value
Year

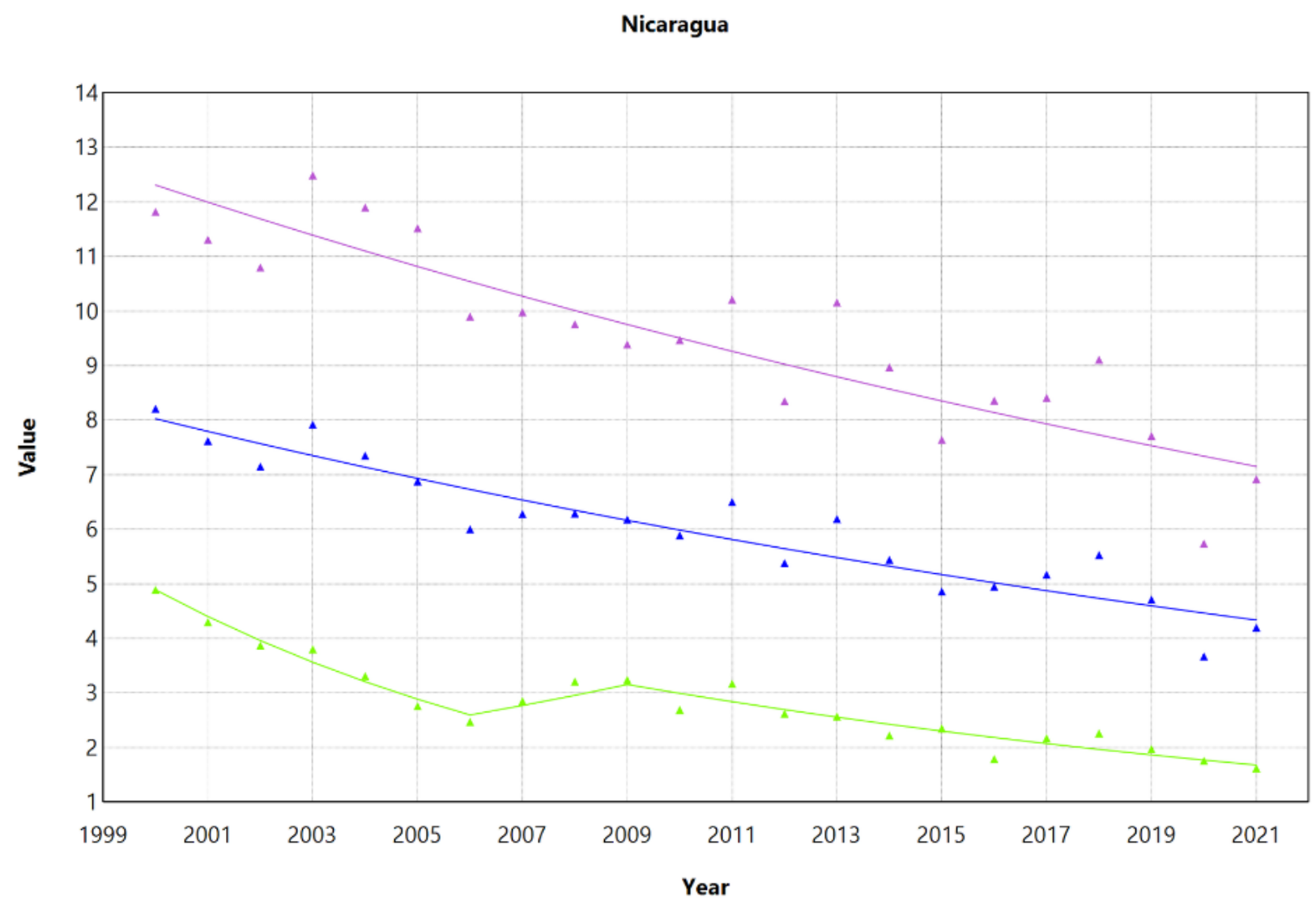
Nicaragua
Value
Year

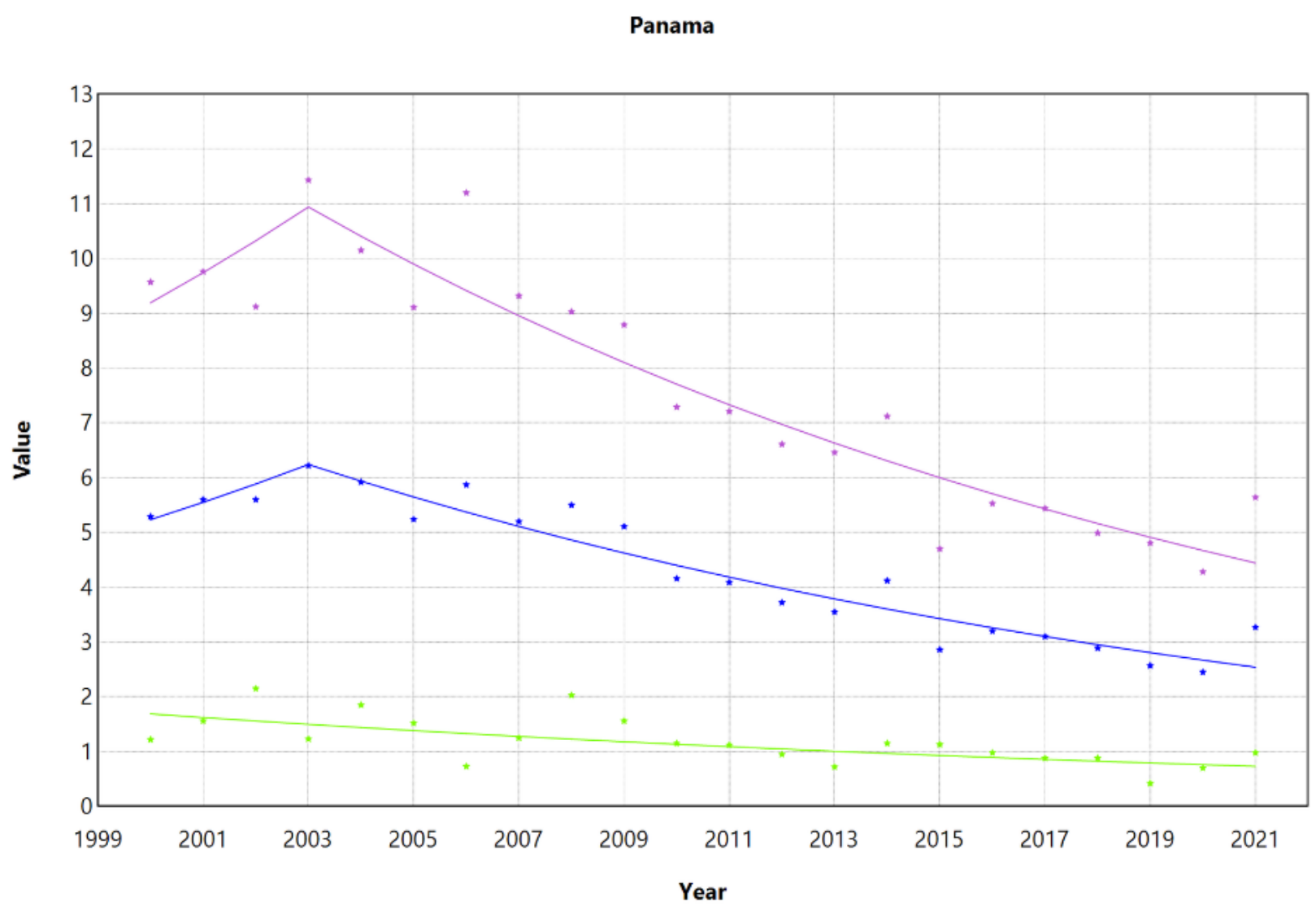
Panama
Value
Year
13
12
11
10
9
8
7
6
5
4
3
2
1
0
1999
2001
2003
2005
2007
2009
2011
2013
2015
2017
2019
2021

(C)

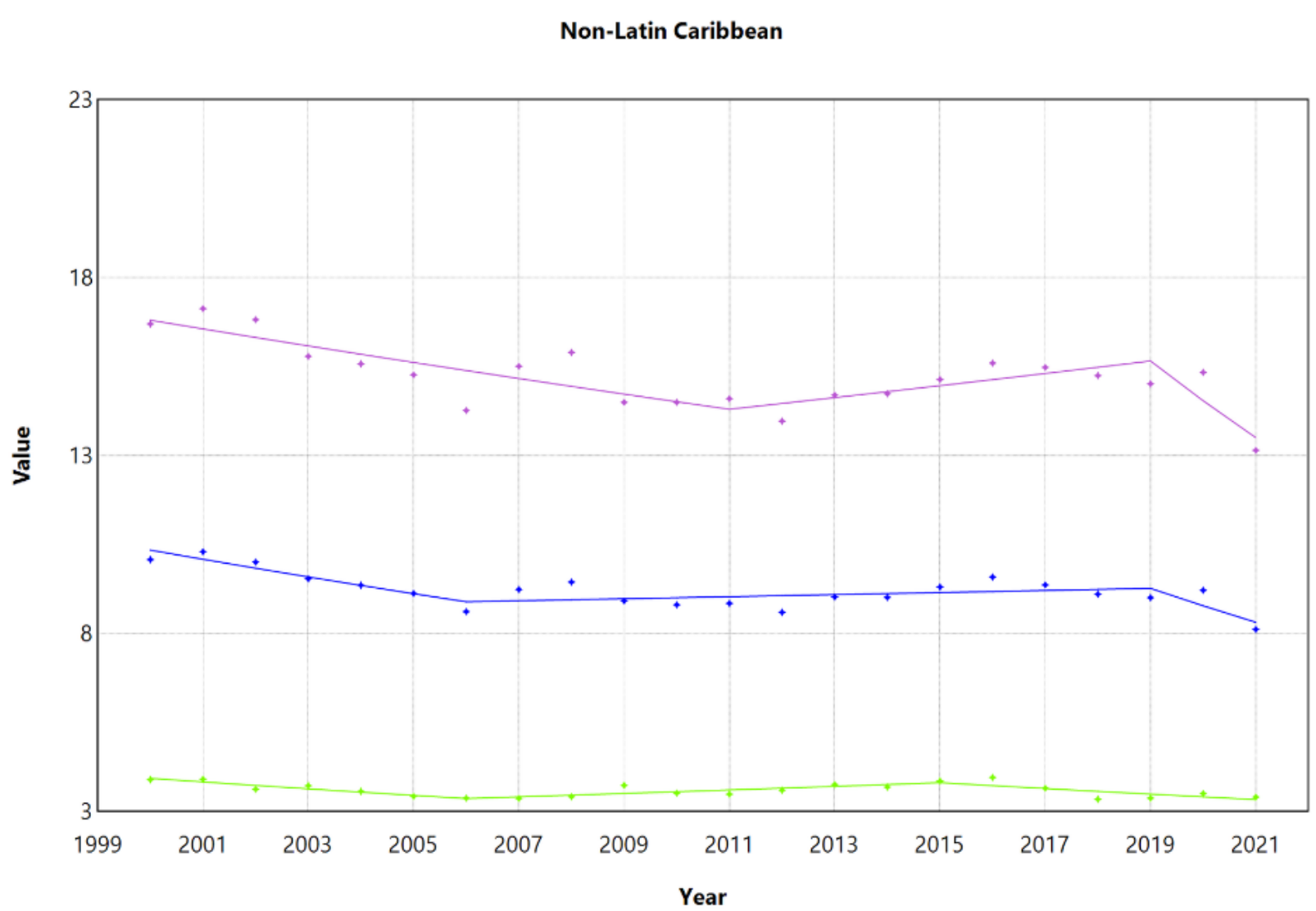
Non-Latin Caribbean
Value
Year
23
18
13
8
3
1999
2001
2003
2005
2007
2009
2011
2013
2015
2017
2019
2021

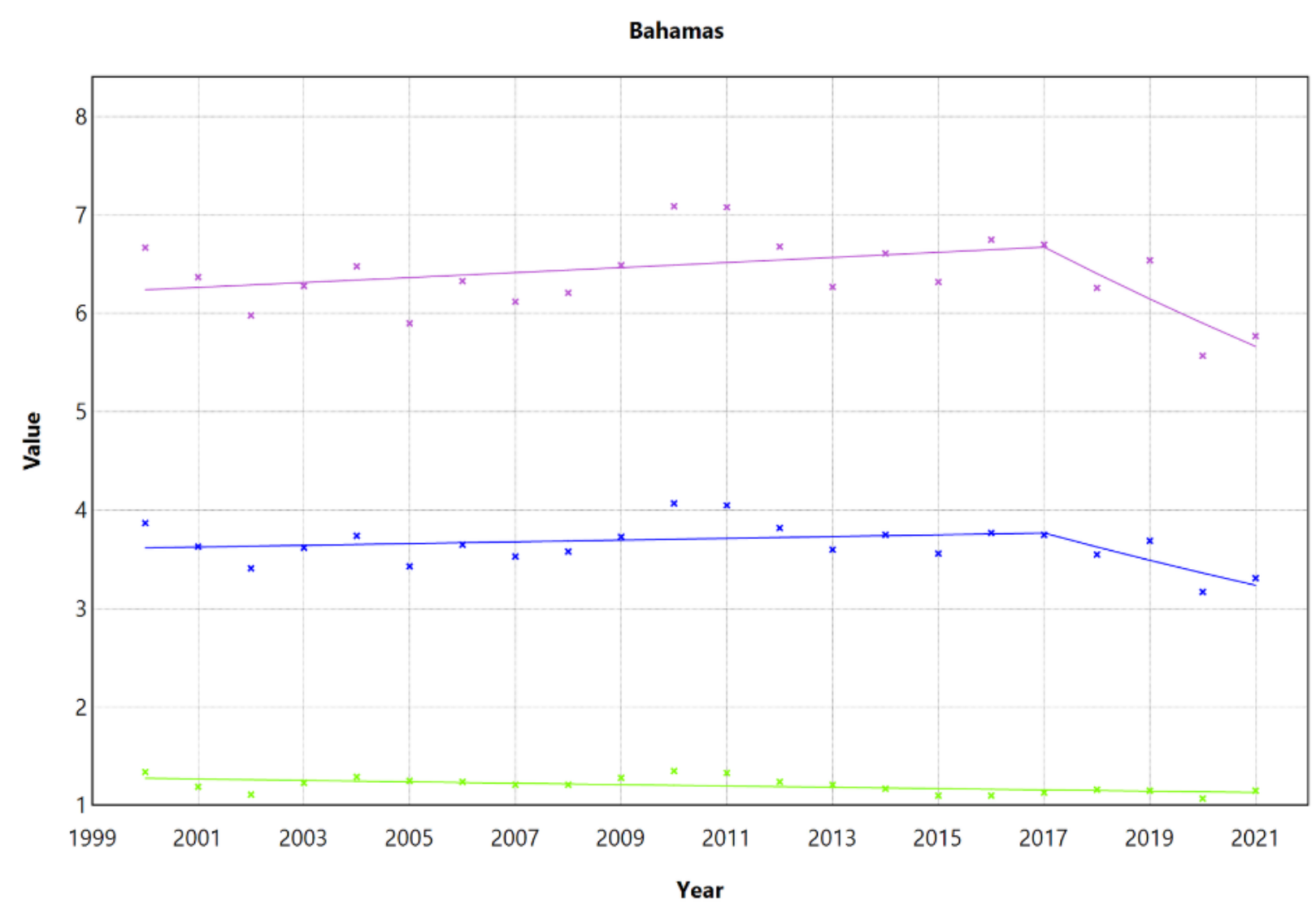
Bahamas
Value
Year
8
7
6
5
4
3
2
1
1999
2001
2003
2005
2007
2009
2011
2013
2015
2017
2019
2021

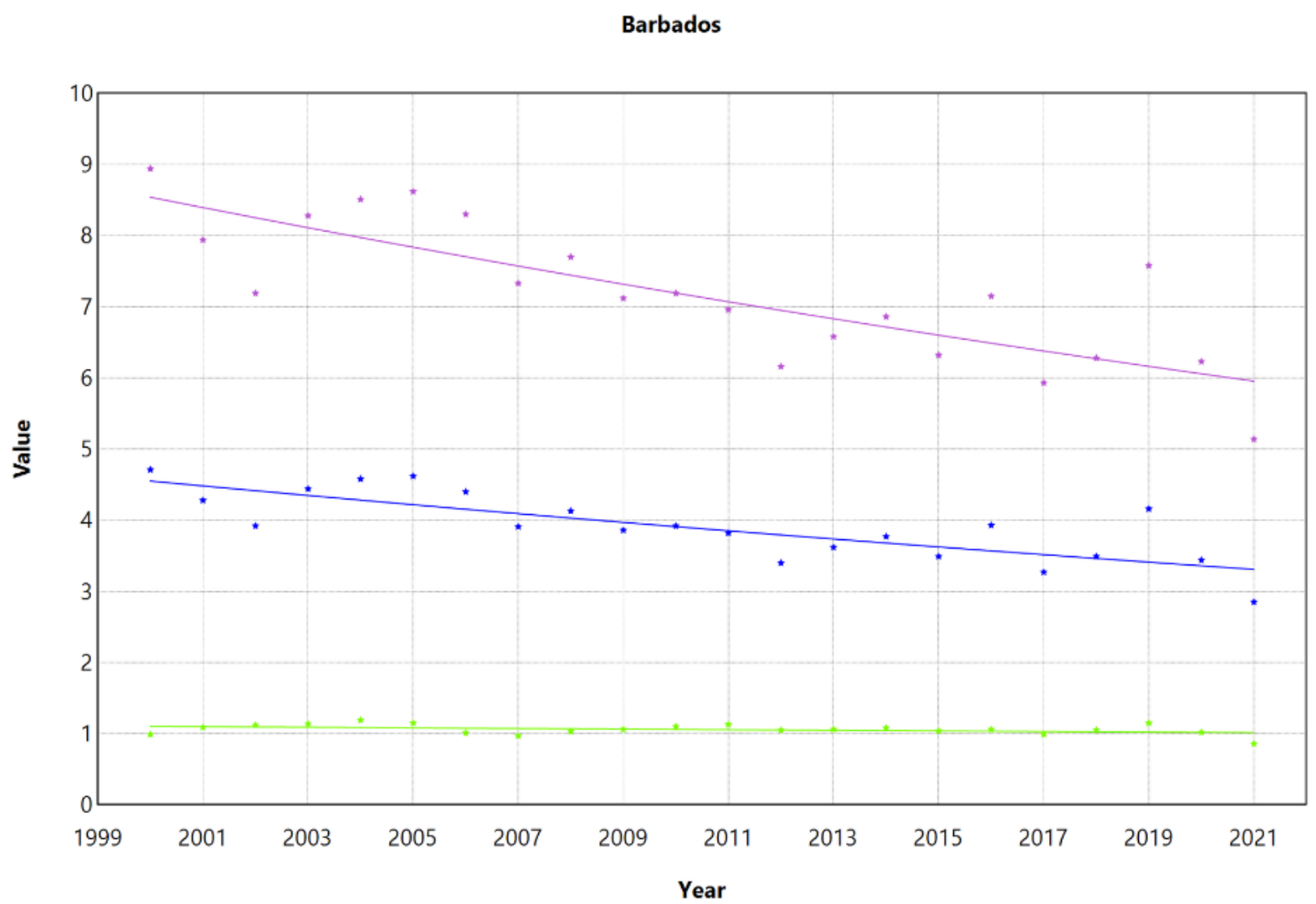
Barbados
Value
Year

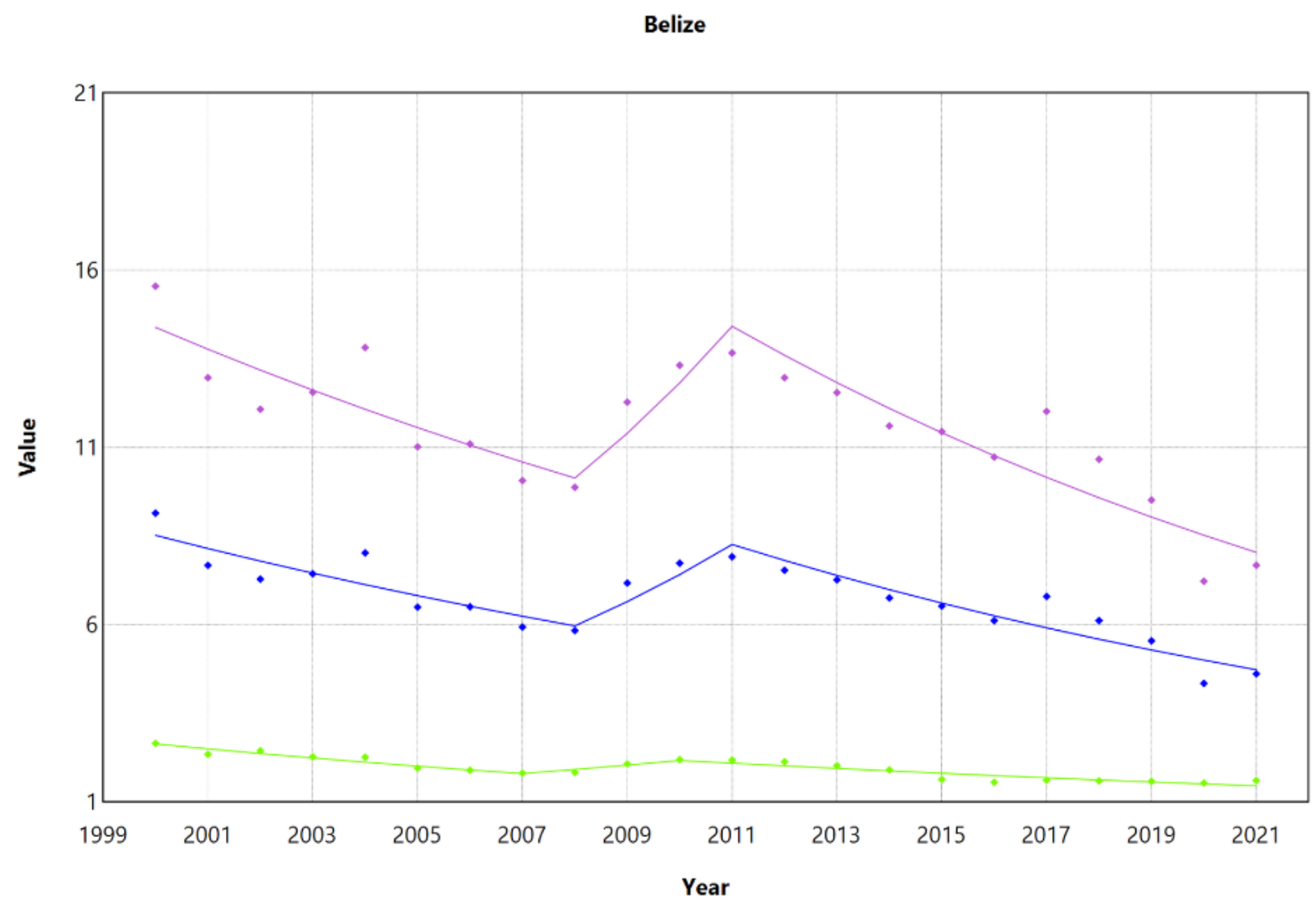
Belize
Value
Year

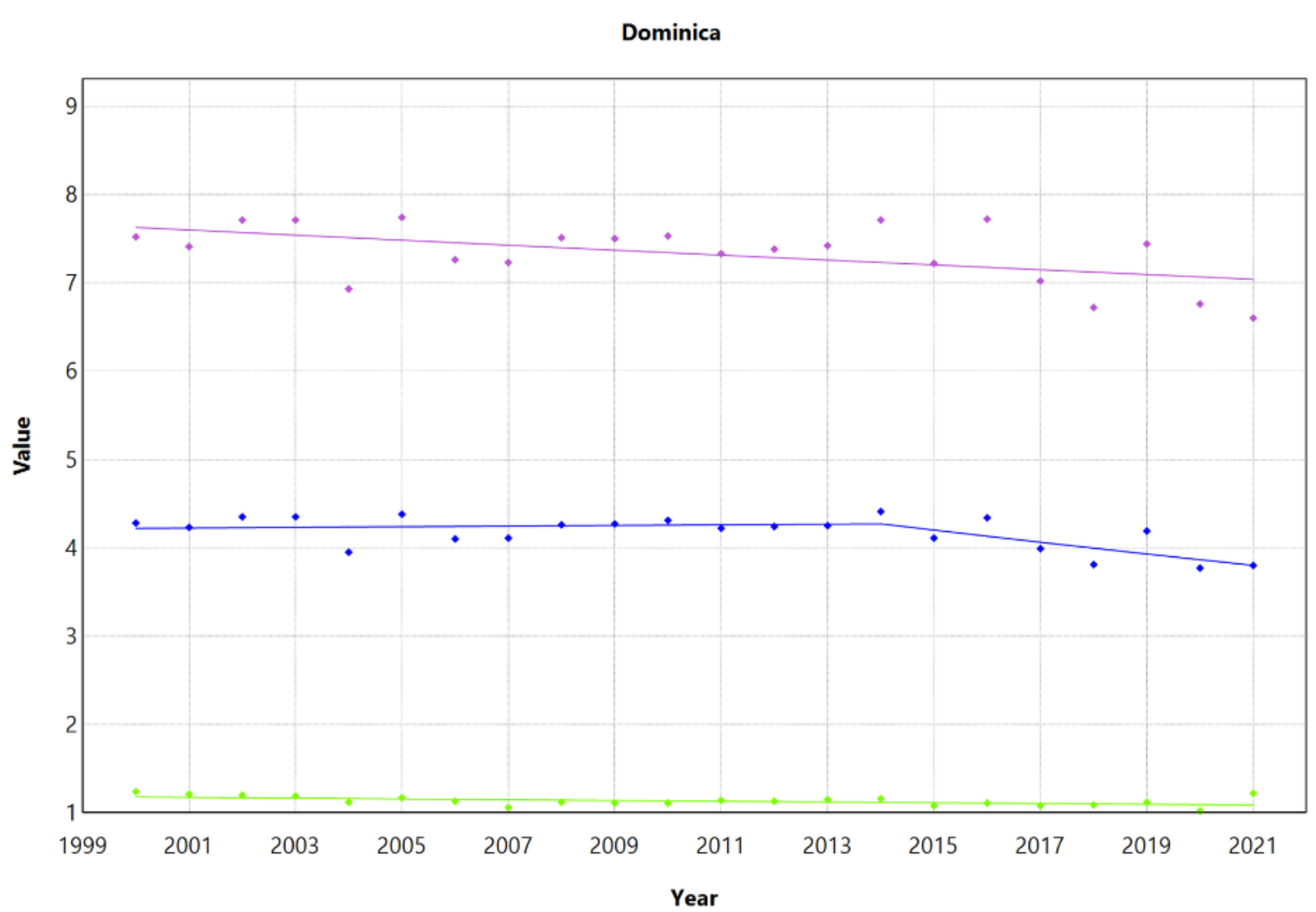
Dominica
Value
Year

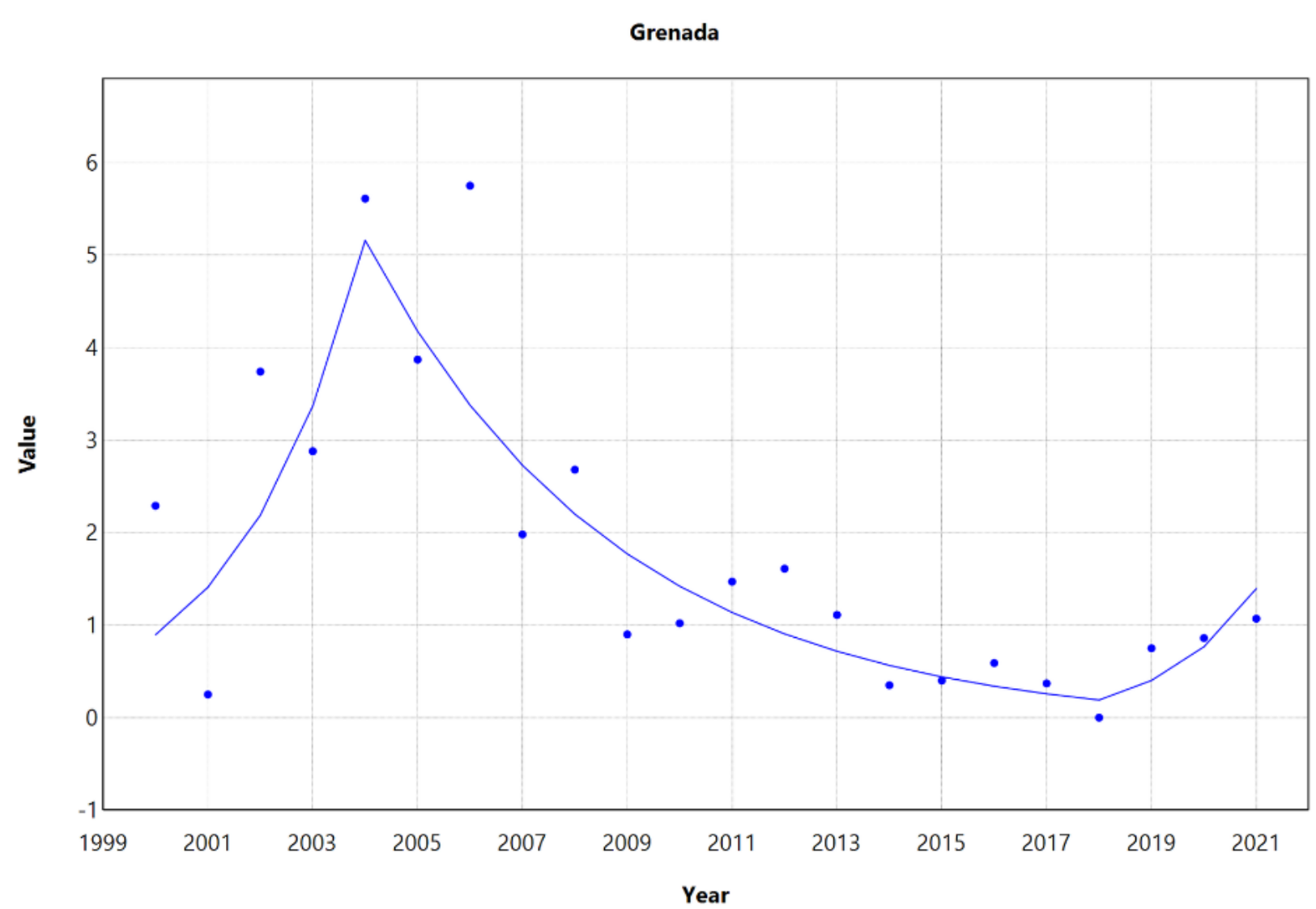
Grenada
Value
Year

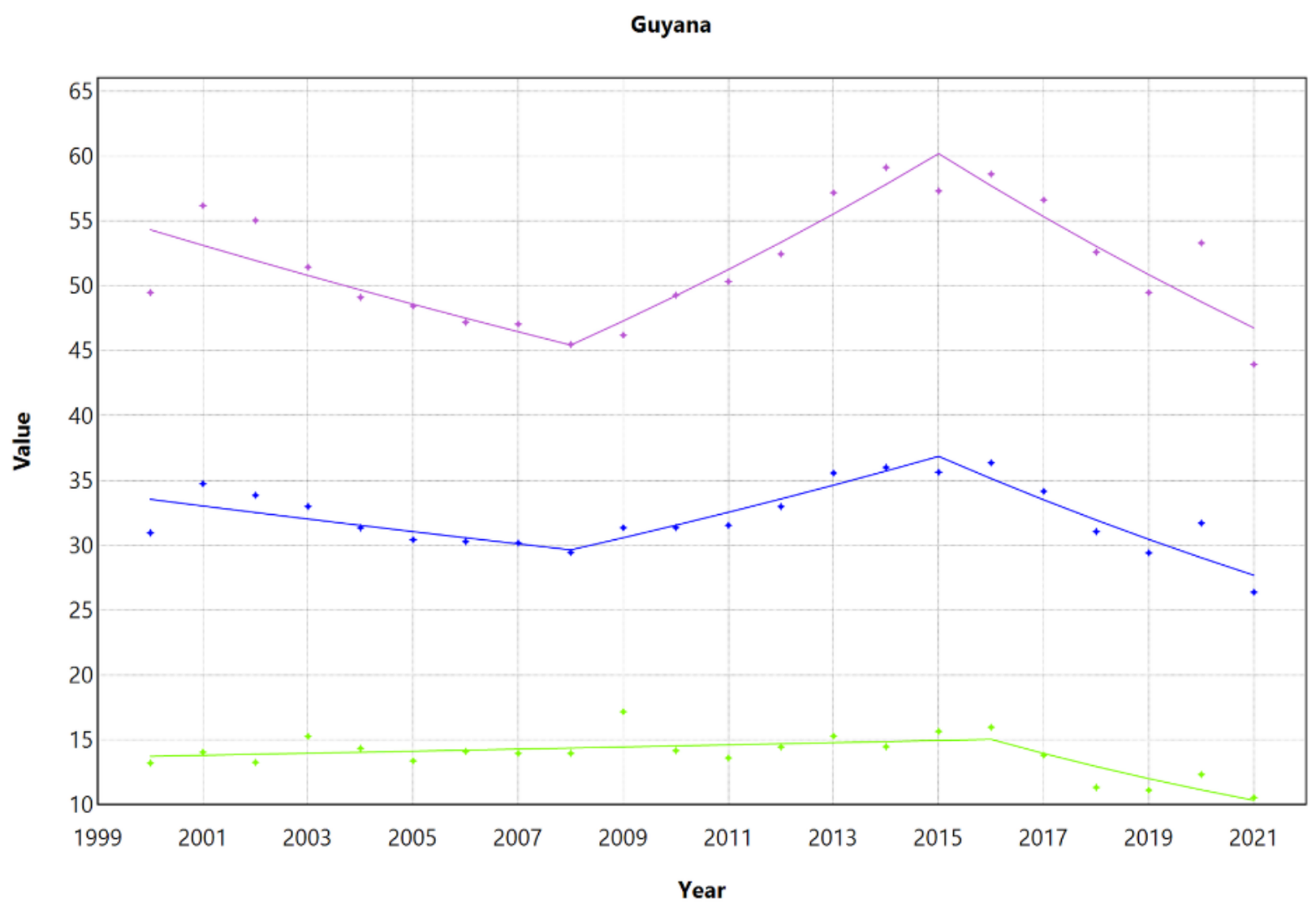
Guyana
Value
Year
65
60
55
50
45
40
35
30
25
20
15
10
1999
2001
2003
2005
2007
2009
2011
2013
2015
2017
2019
2021

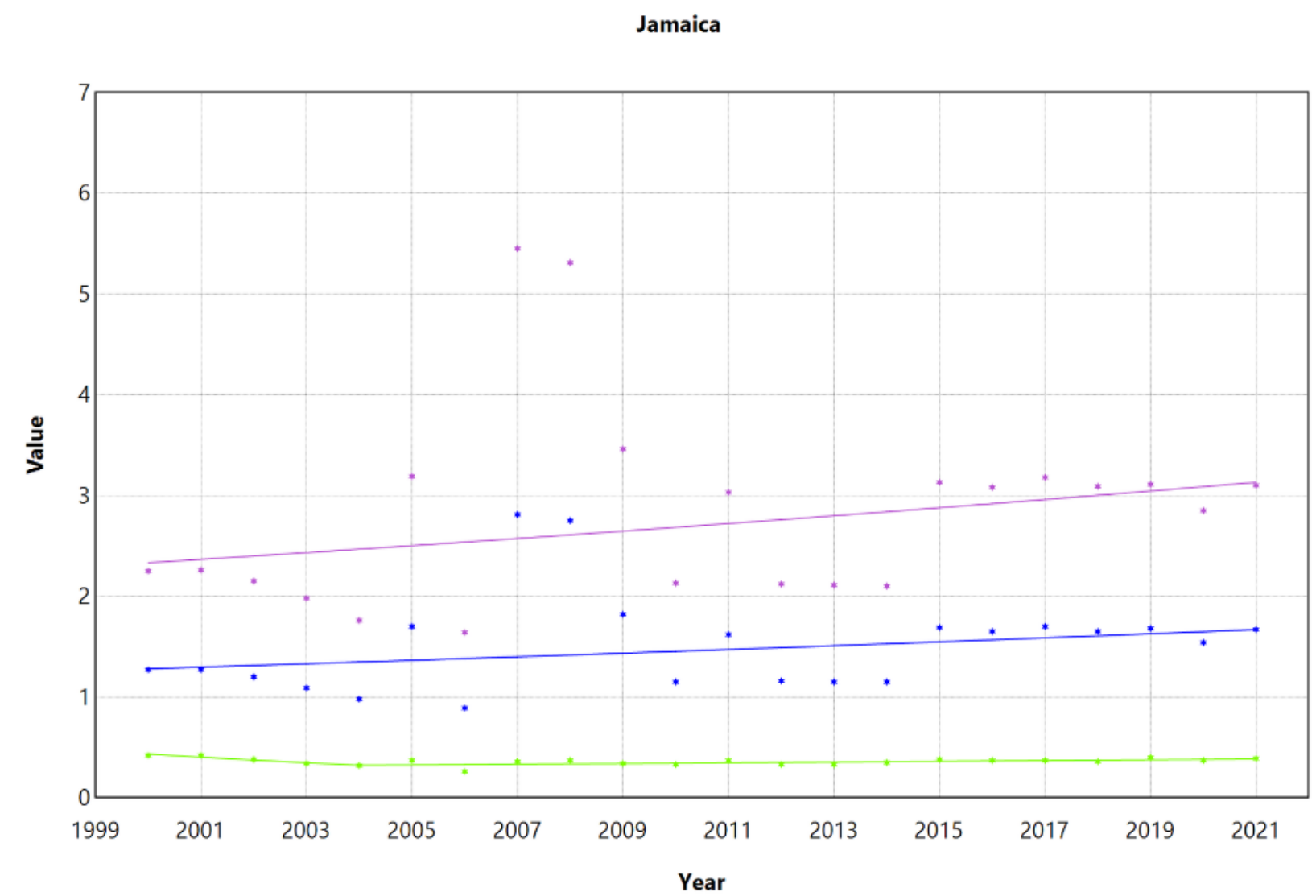
Jamaica
Value
Year
7
6
5
4
3
2
1
0
1999
2001
2003
2005
2007
2009
2011
2013
2015
2017
2019
2021

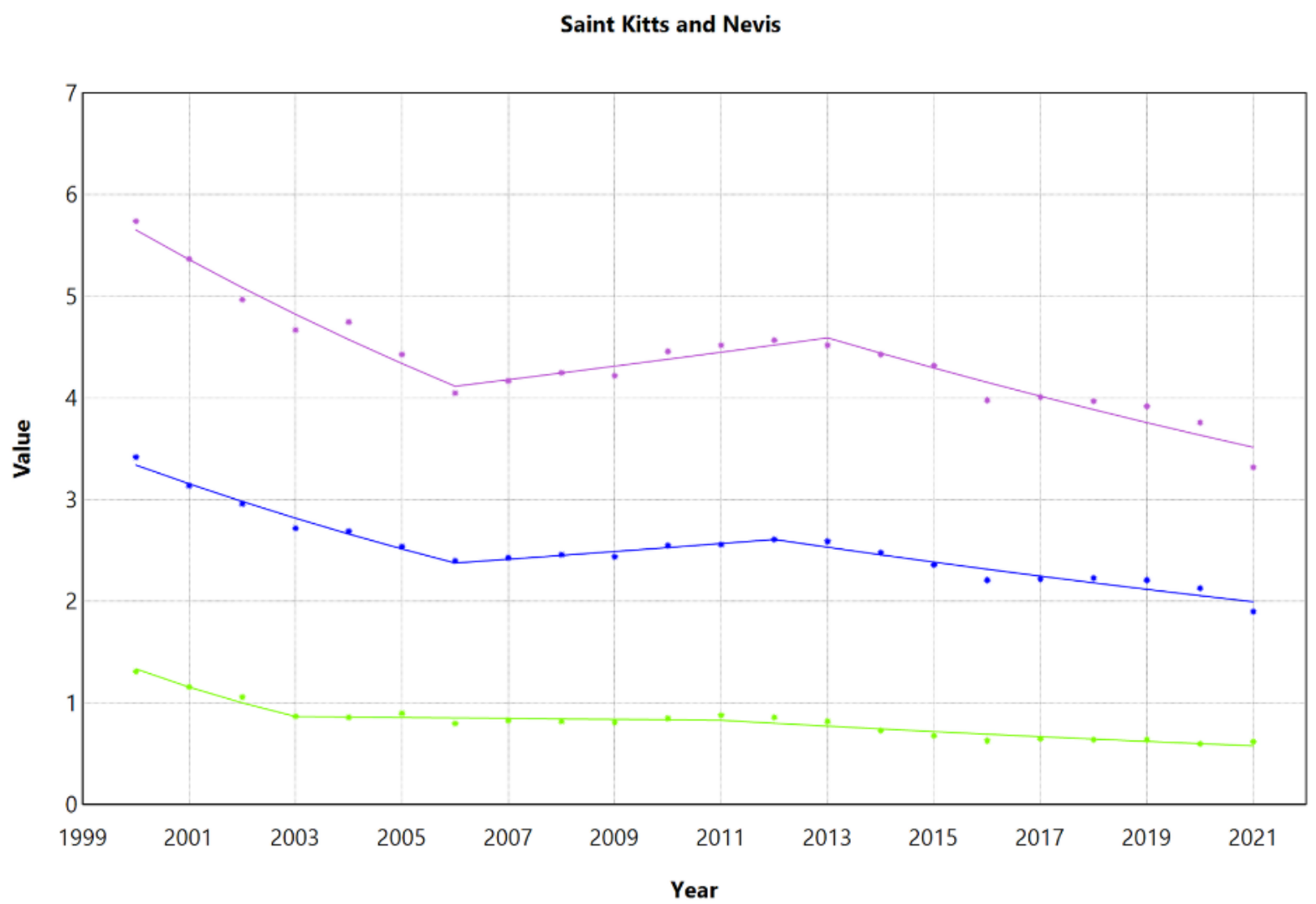
Saint Kitts and Nevis
Value
Year
7
6
5
4
3
2
1
0
1999
2001
2003
2005
2007
2009
2011
2013
2015
2017
2019
2021

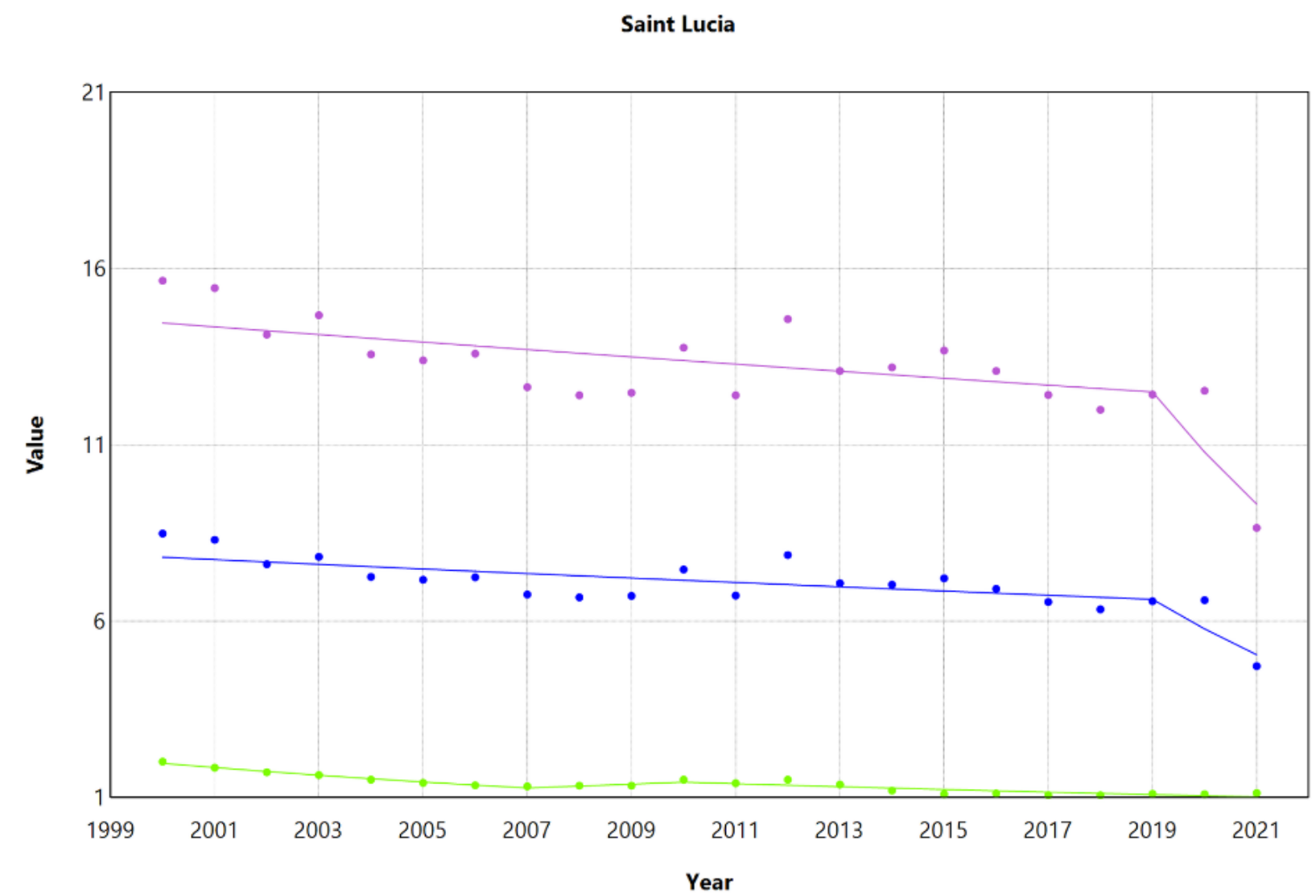
Saint Lucia
Value
Year
21
16
11
6
1
1999
2001
2003
2005
2007
2009
2011
2013
2015
2017
2019
2021

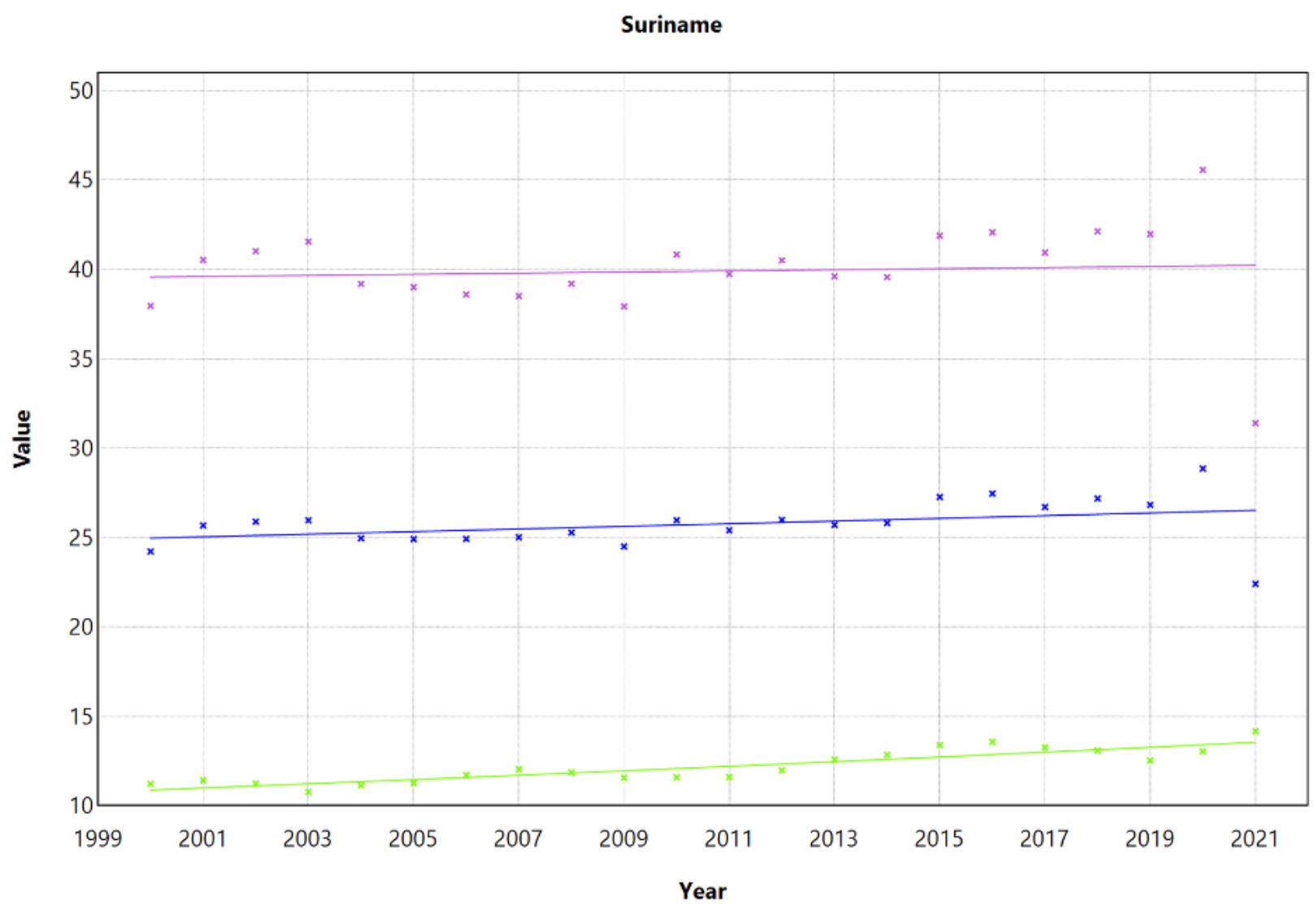
Suriname
Value
Year
50
45
40
35
30
25
20
15
10
1999
2001
2003
2005
2007
2009
2011
2013
2015
2017
2019
2021

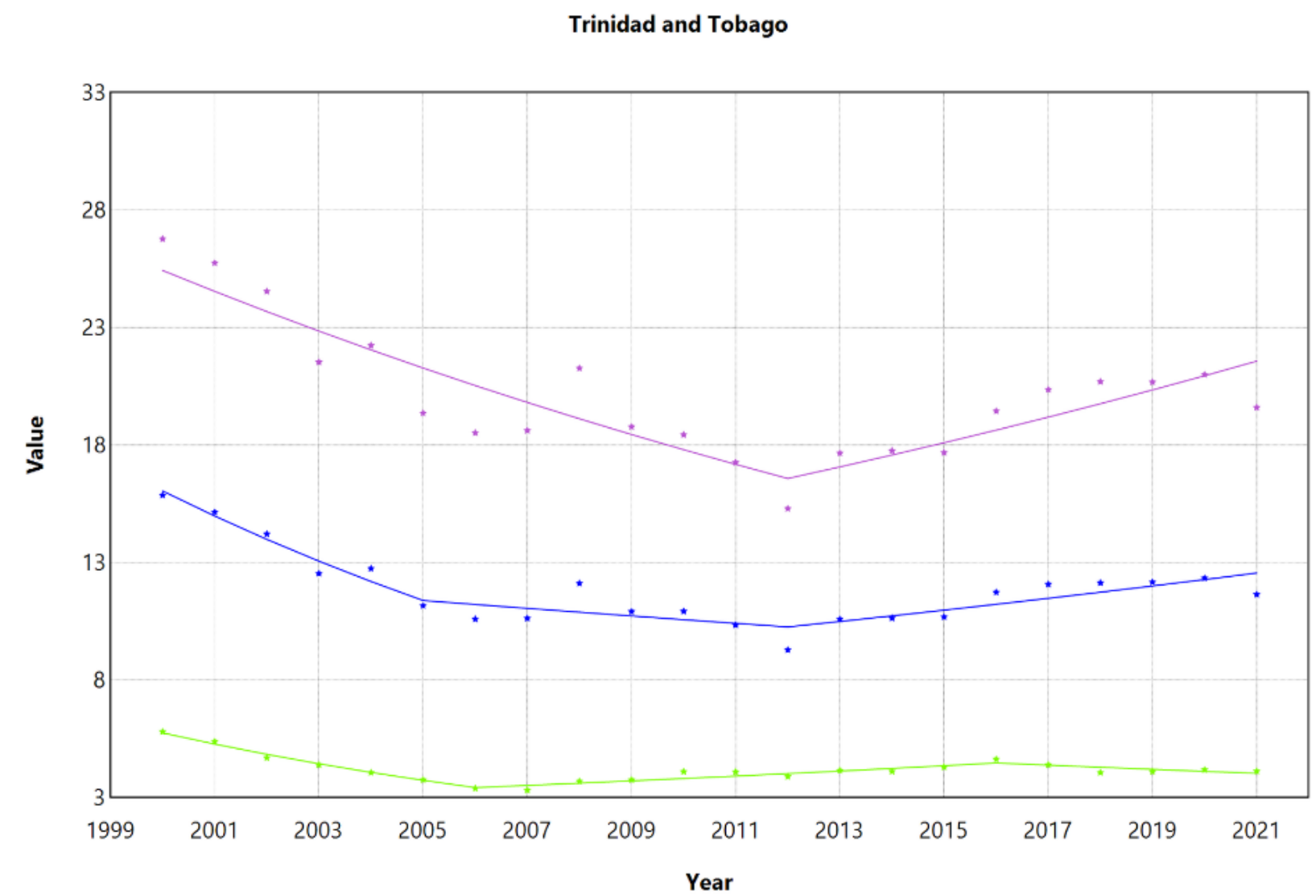
Trinidad and Tobago
Value
Year
33
28
23
18
13
8
3
1999
2001
2003
2005
2007
2009
2011
2013
2015
2017
2019
2021

(D)

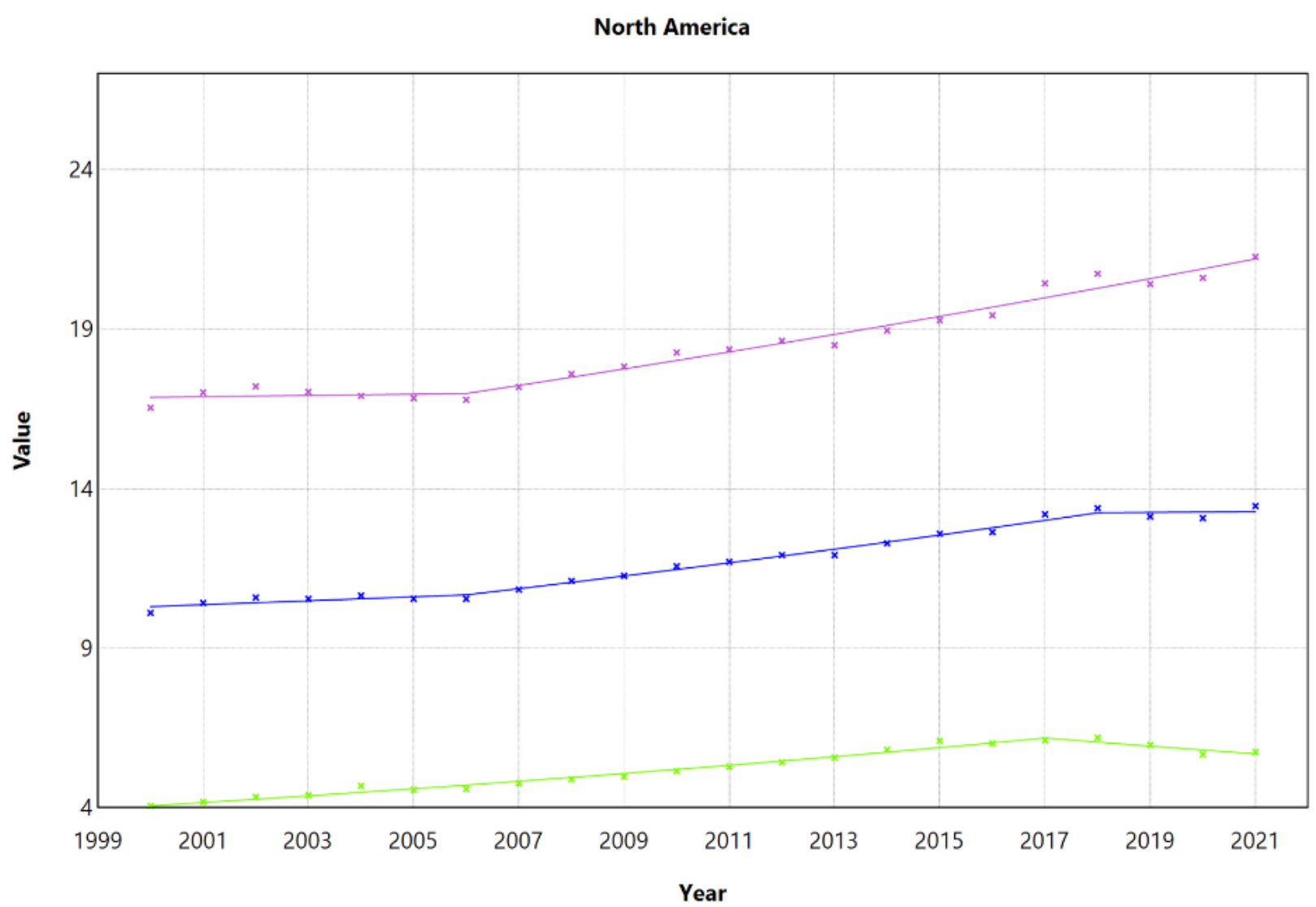
North America
Value
Year
24
19
14
9
4
1999
2001
2003
2005
2007
2009
2011
2013
2015
2017
2019
2021

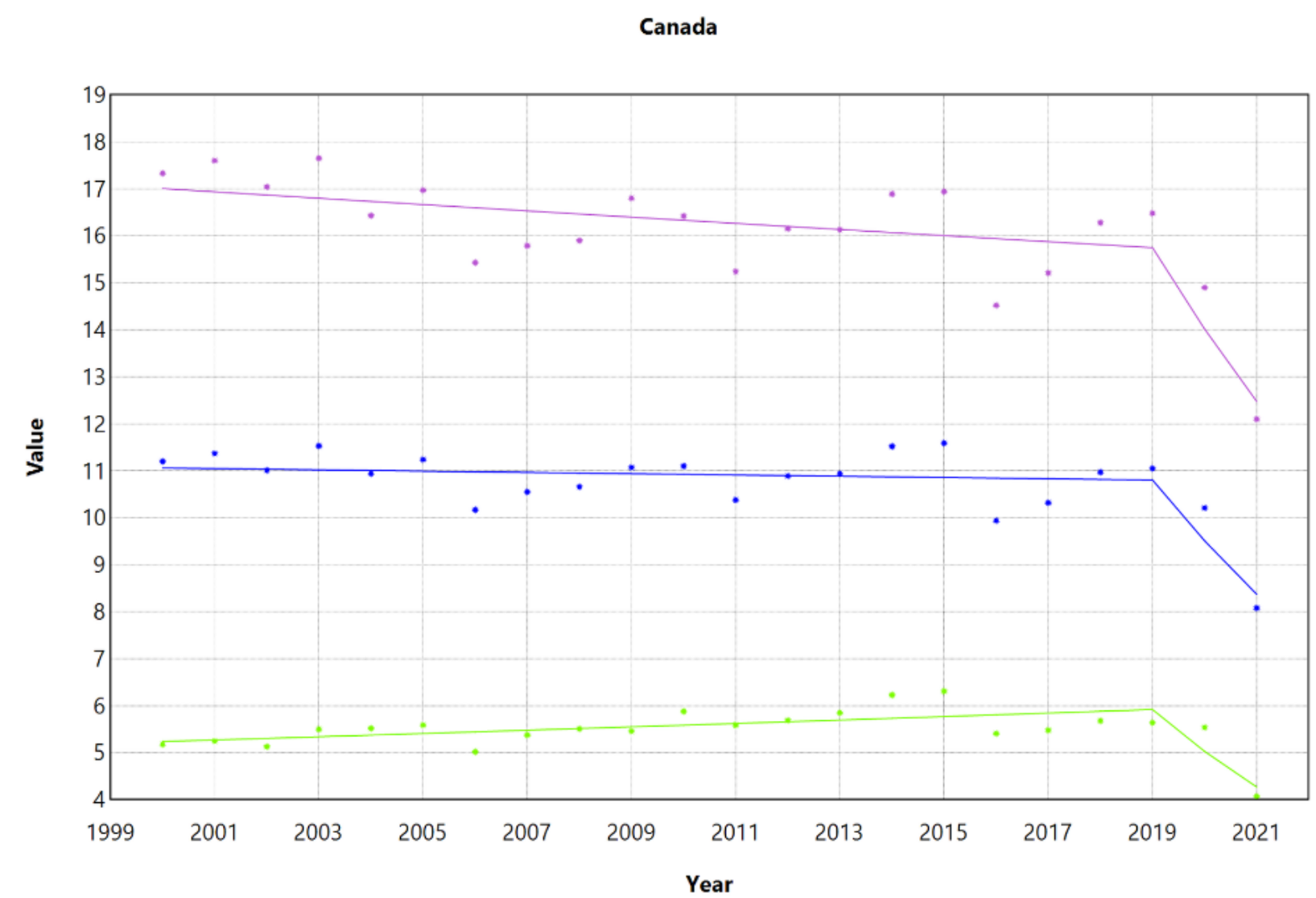
Canada
Value
Year
19
18
17
16
15
14
13
12
11
10
9
8
7
6
5
4
1999
2001
2003
2005
2007
2009
2011
2013
2015
2017
2019
2021

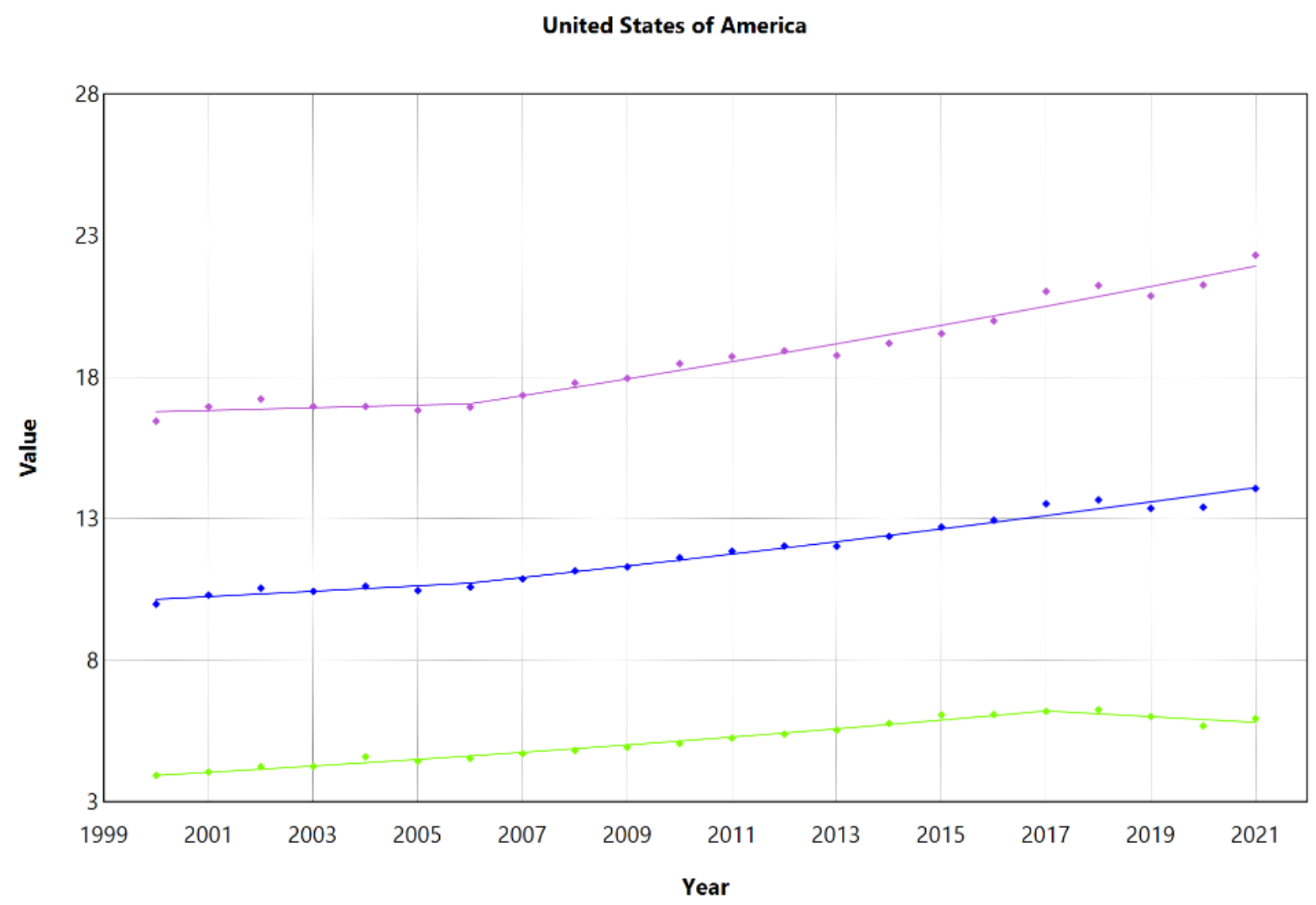
United States of America
Value
28
23
18
13
8
3
1999
2001
2003
2005
2007
2009
2011
2013
2015
2017
2019
2021
Year

(E)

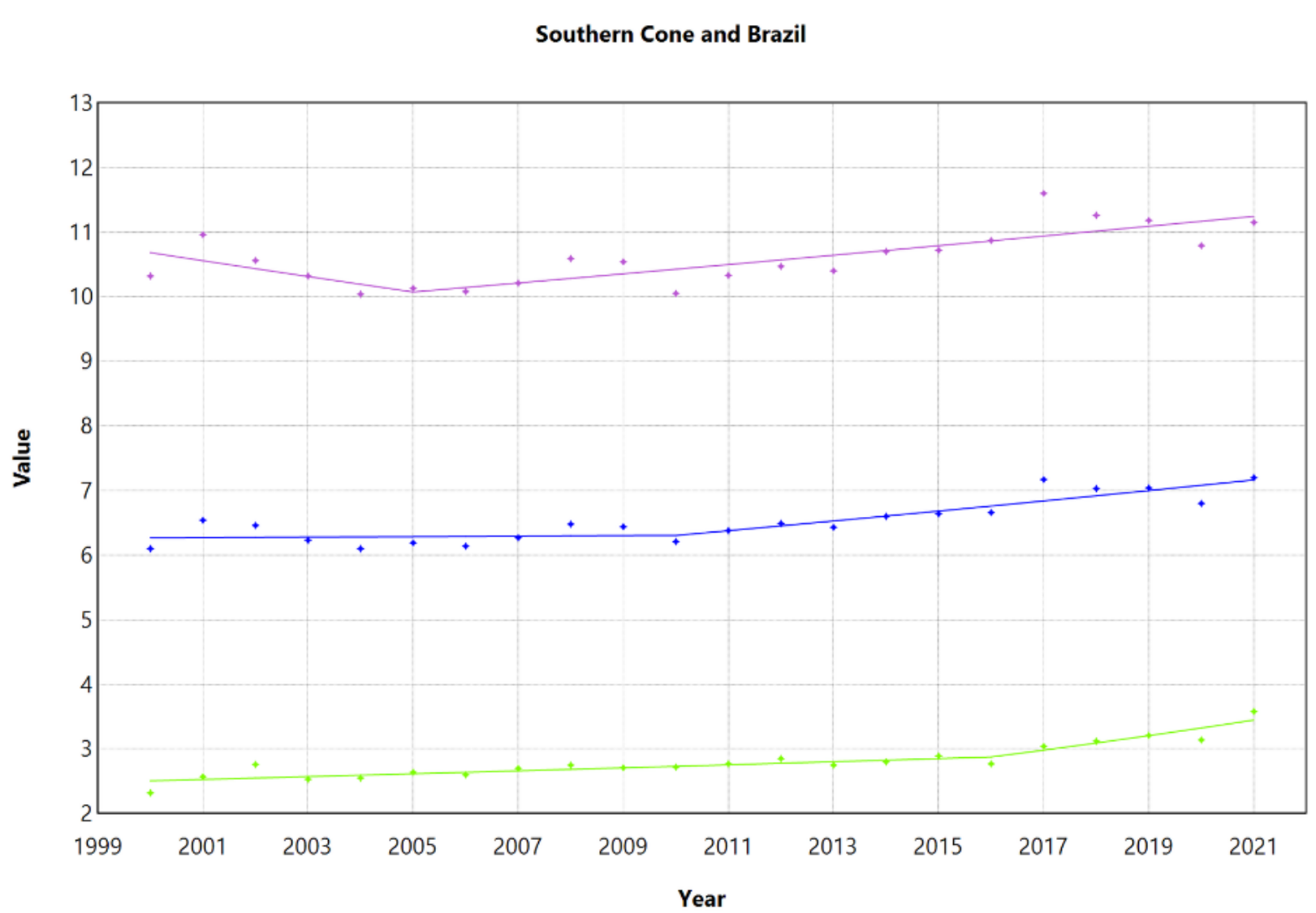
Southern Cone and Brazil
Value
13
12
11
10
9
8
7
6
5
4
3
2
1999
2001
2003
2005
2007
2009
2011
2013
2015
2017
2019
2021
Year

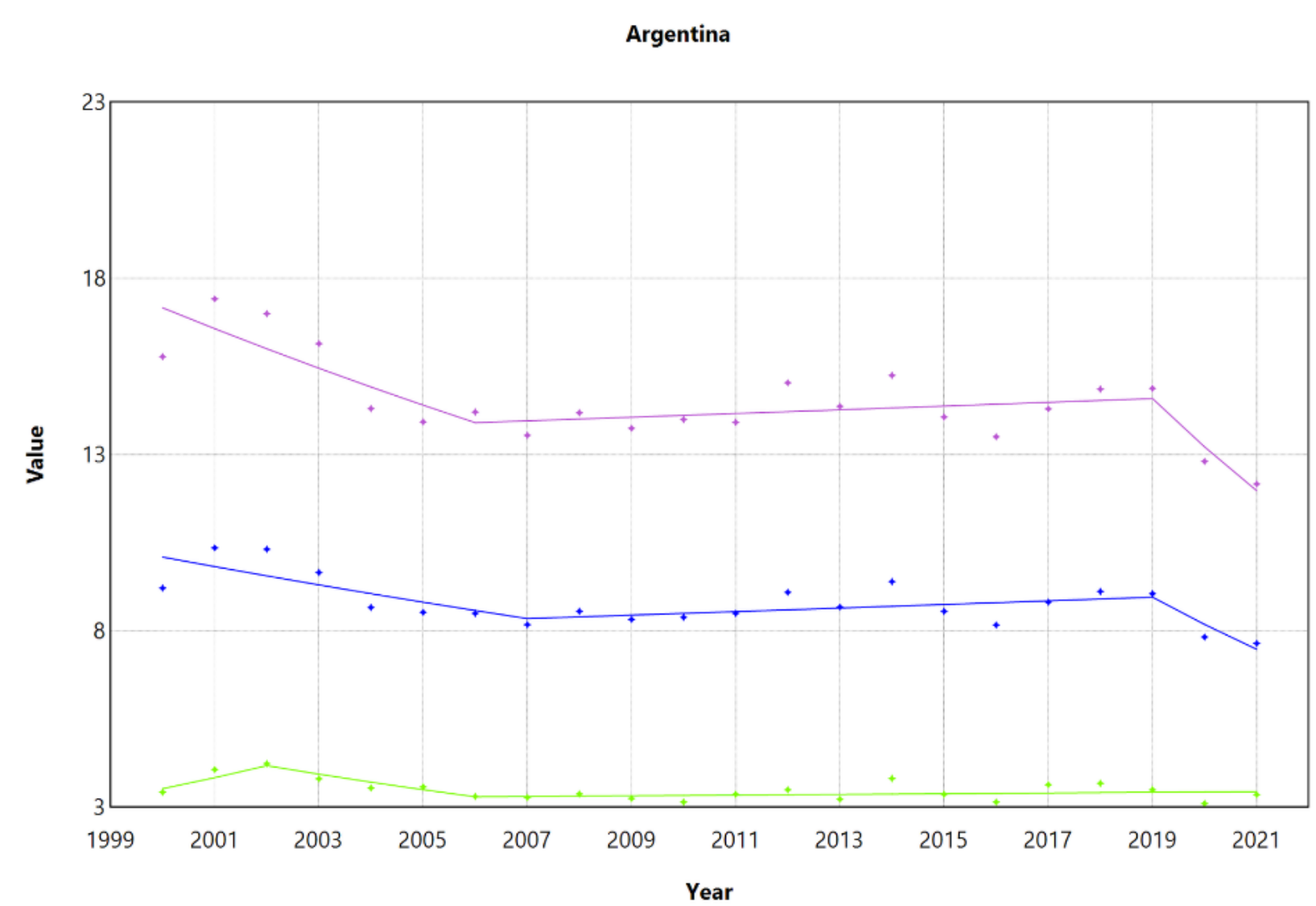
Argentina
Value
23
18
13
8
3
1999
2001
2003
2005
2007
2009
2011
2013
2015
2017
2019
2021
Year

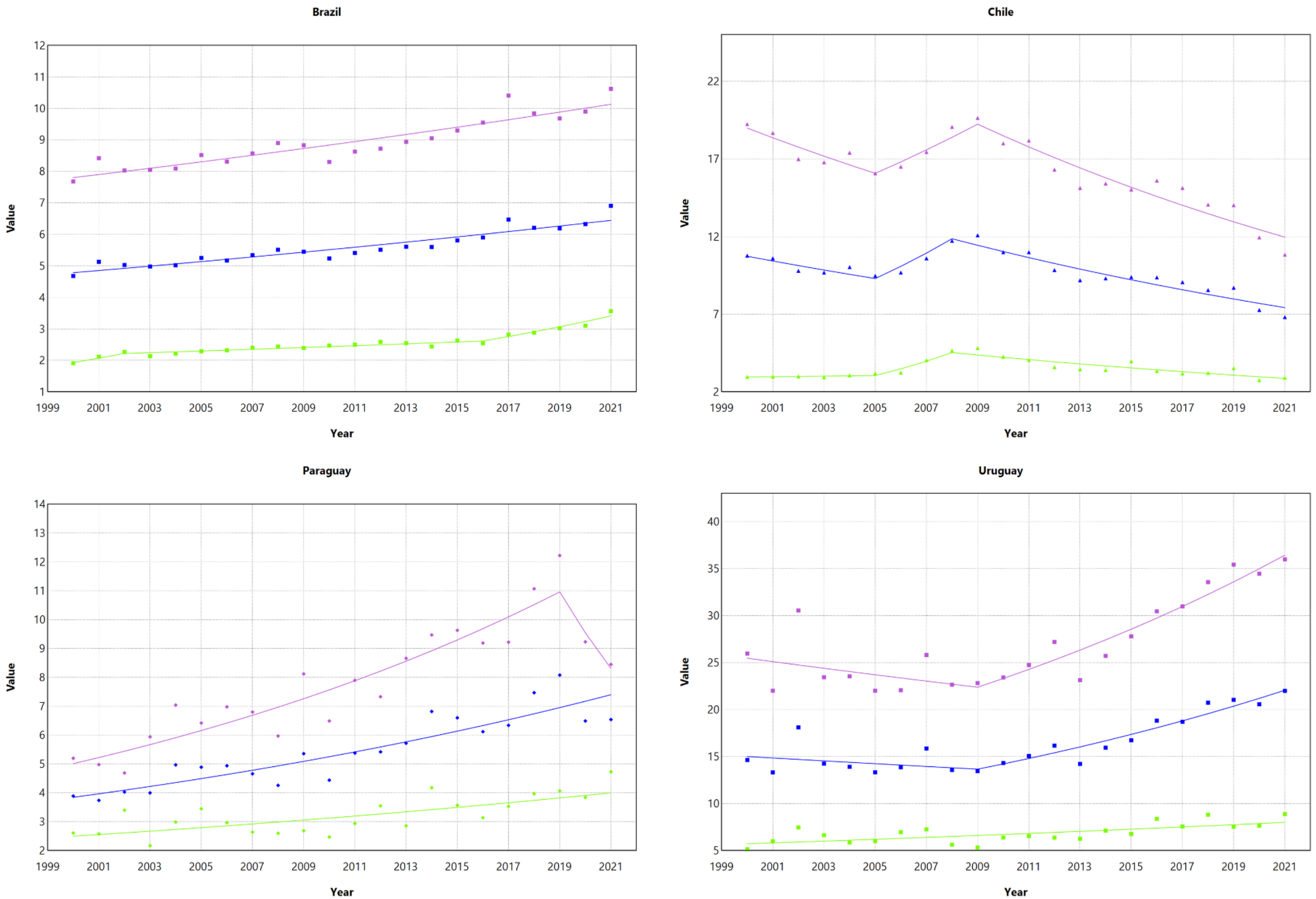
Brazil
Value
Year
Chile
Value
Year
Paraguay
Value
Year
Uruguay
Value
Year

(F)

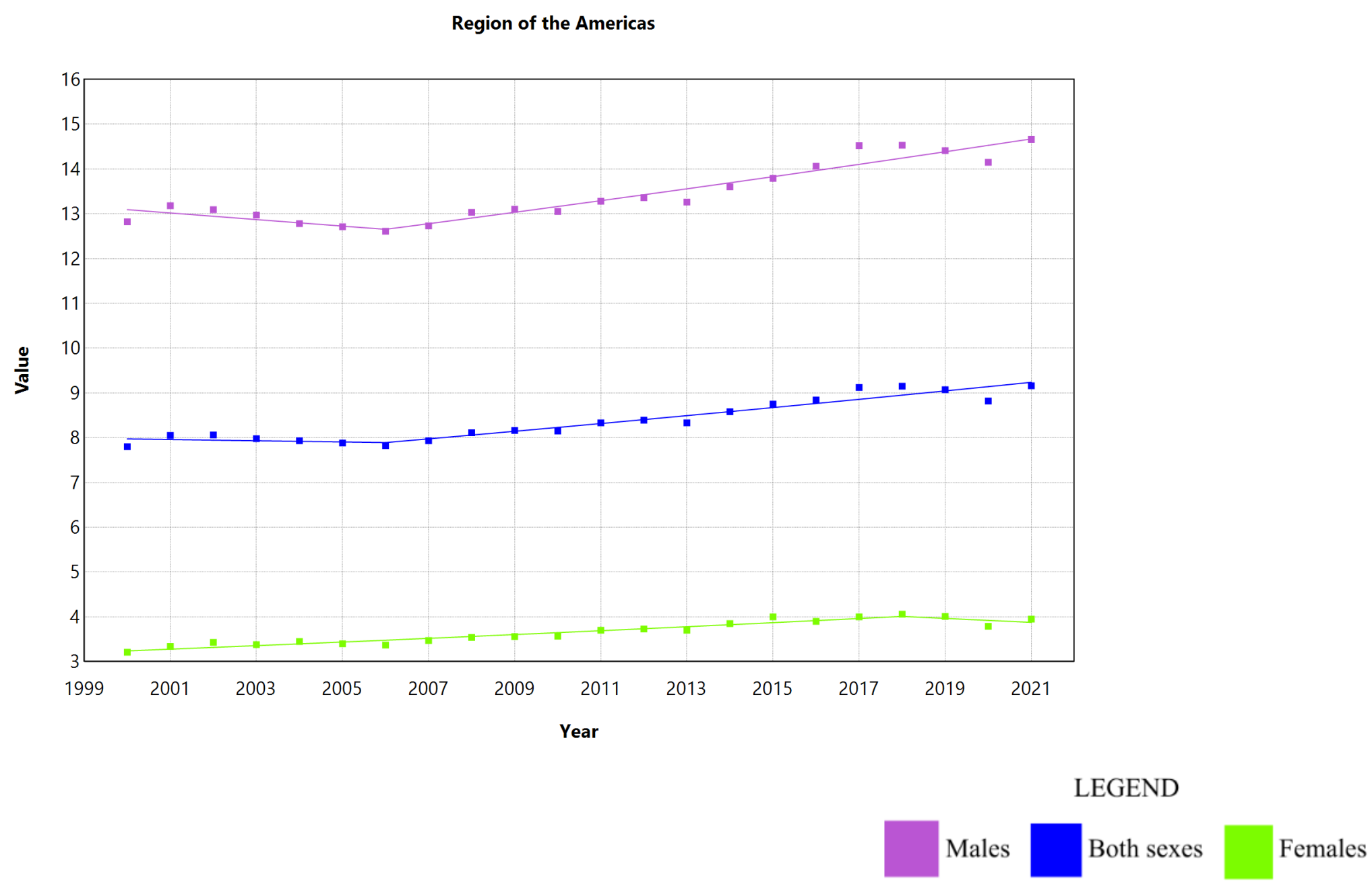
Region of the Americas
Value
Year
16
15
14
13
12
11
10
9
8
7
6
5
4
3
1999
2001
2003
2005
2007
2009
2011
2013
2015
2017
2019
2021
LEGEND
Males
Both sexes
Females

Figure S4. Age-standardized suicide mortality rate per 100,000 population among males in 2021 and average annual percentage change between 2015 and 2021 in 31 countries in the Region of the Americas (indicated by ISO3 country code)

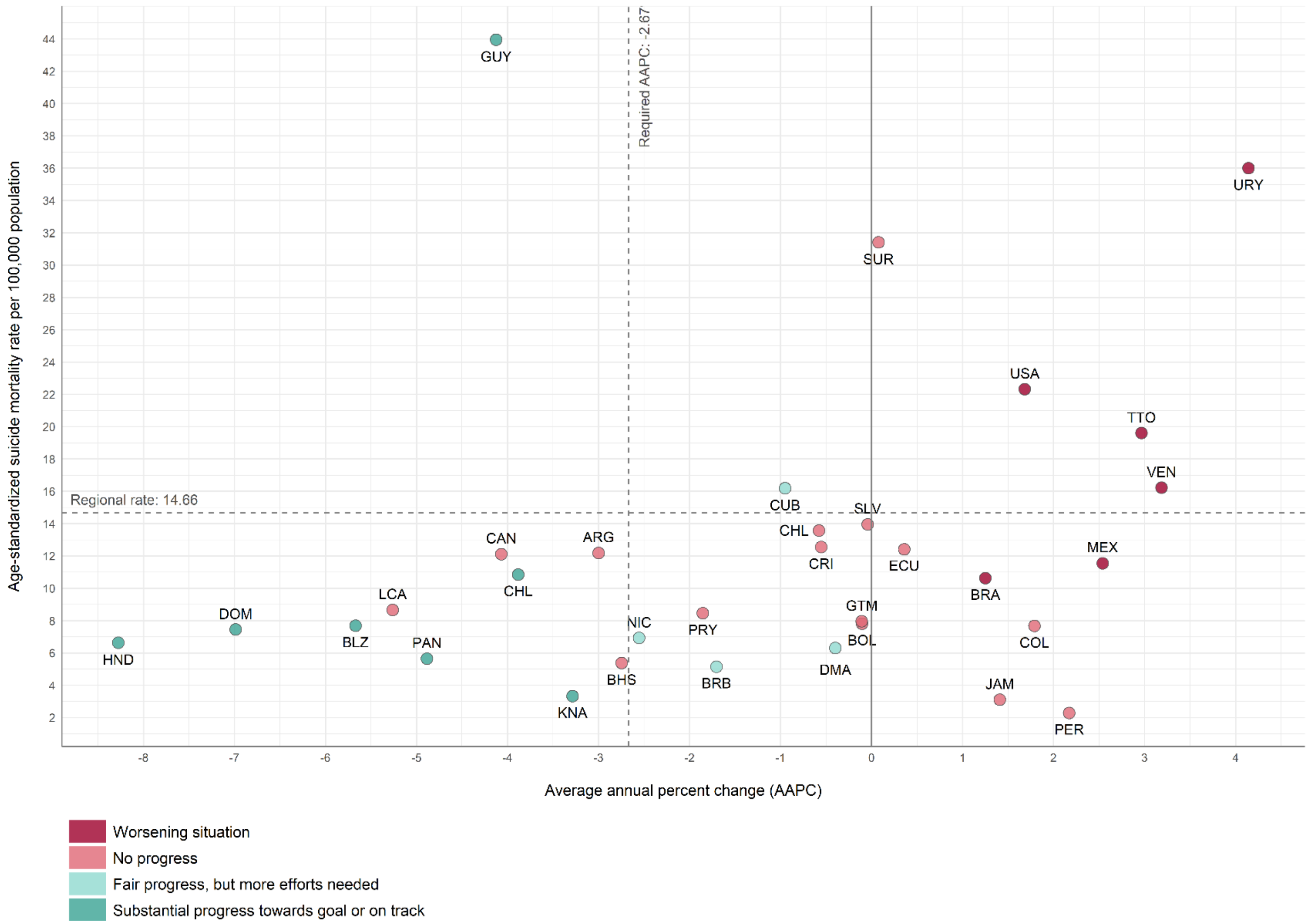

Note. The vertical dotted line indicates an AAPC of -2·67%, representing the compound annual reduction required to achieve the one-third reduction target by 2030. The horizontal dotted line indicates the suicide mortality rate among males in the region in 2021.

Grenada was excluded from this figure because its AAPC was an extreme outlier relative to the other countries. Inclusion of Grenada would have substantially expanded the x-axis scale and reduced the readability of the remaining country estimates.

Figure S5. Age-standardized suicide mortality rate per 100,000 population among females in 2021 and average annual percentage change between 2015 and 2021 in 31 countries in the Region of the Americas (indicated by ISO3 country code)

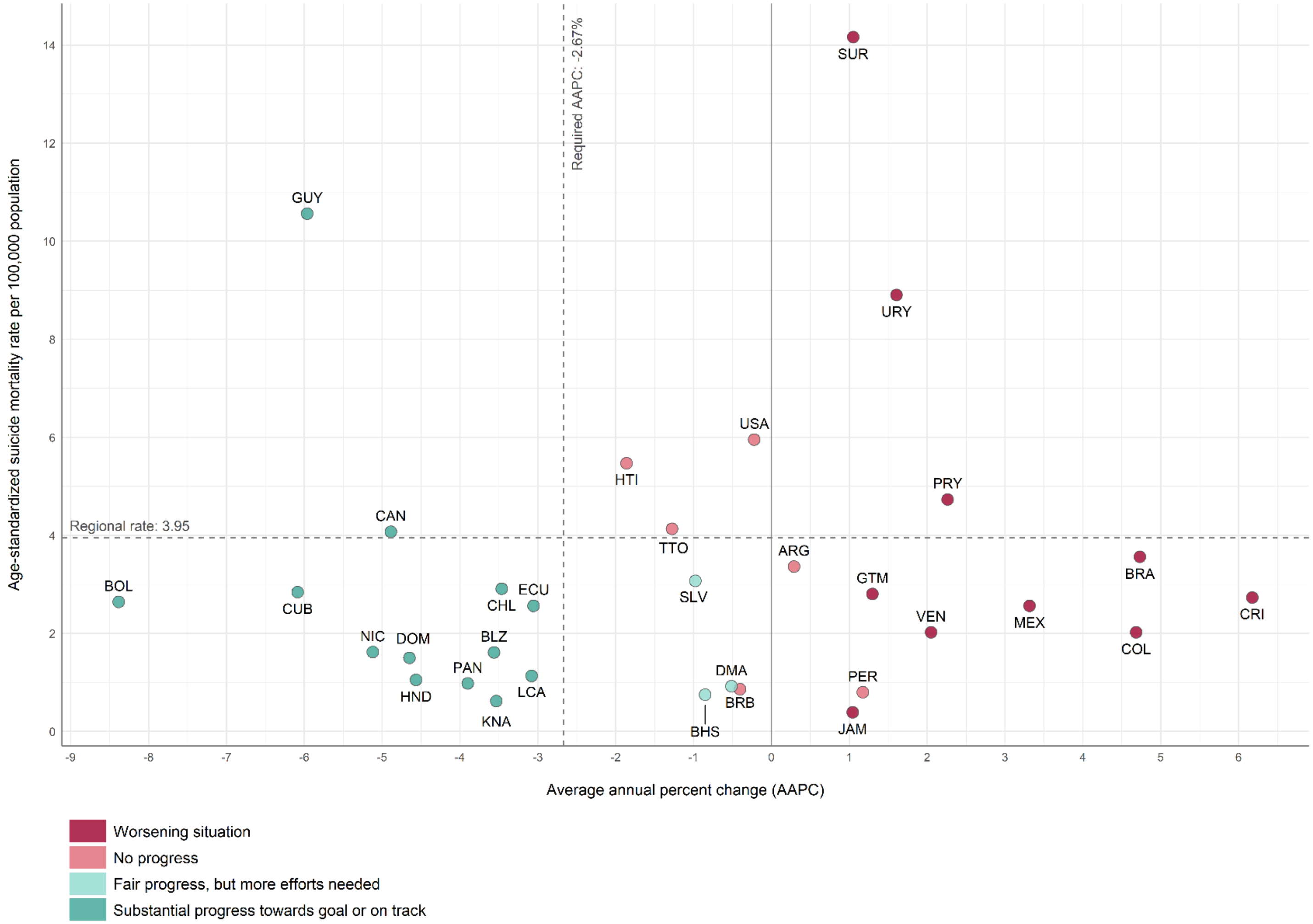

Note. The vertical dotted line indicates an AAPC of -2·67%, representing the compound annual reduction required to achieve the one-third reduction target by 2030. The horizontal dotted line indicates the suicide mortality rate among females in the region in 2021.

Grenada was excluded from this figure because its AAPC was an extreme outlier relative to the other countries. Inclusion of Grenada would have substantially expanded the x-axis scale and reduced the readability of the remaining country estimates.

Figure S6. Trends for the proportion of suicides by method for (A) Both sexes, (B) Males and (C) Females by country

(A)

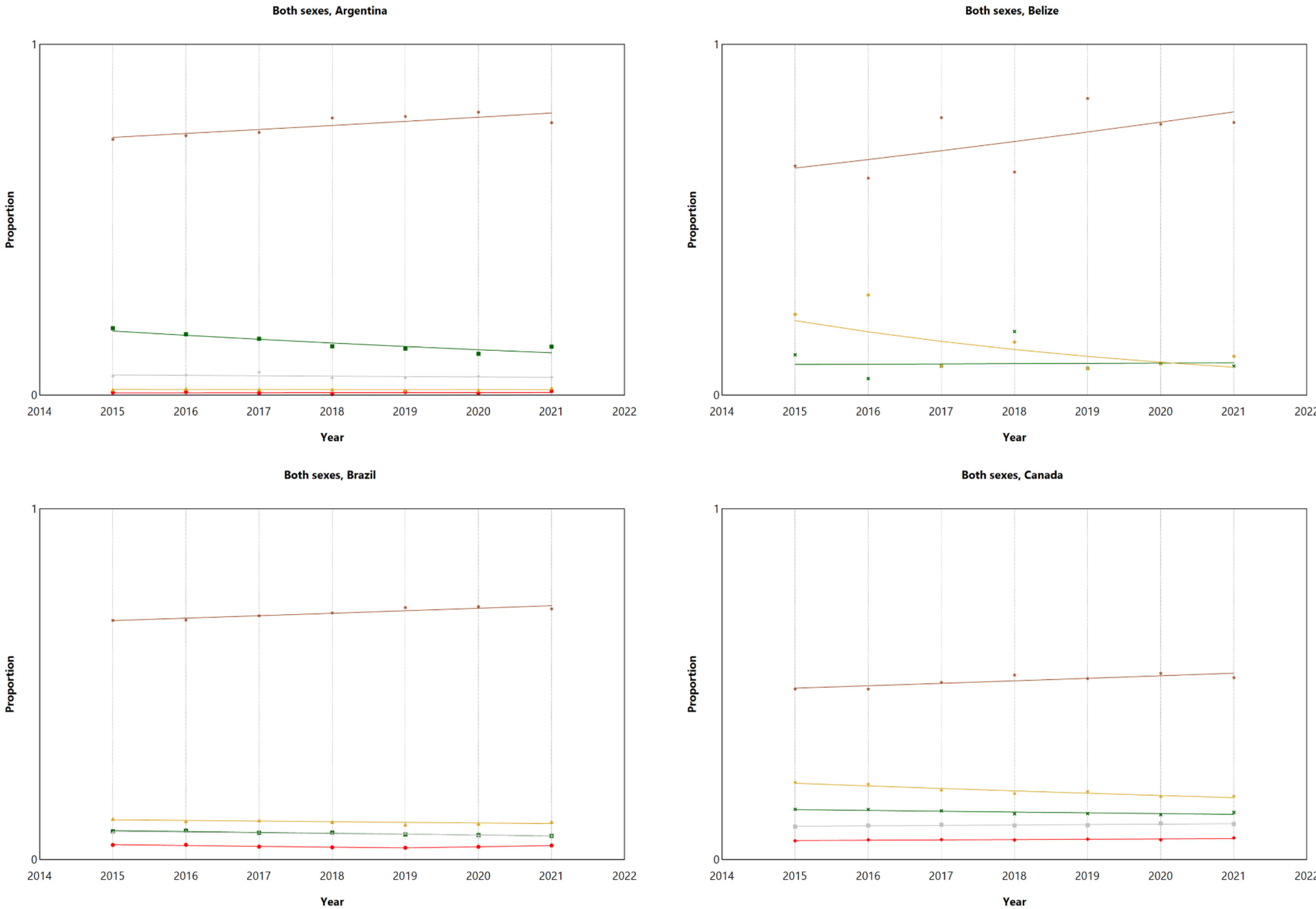

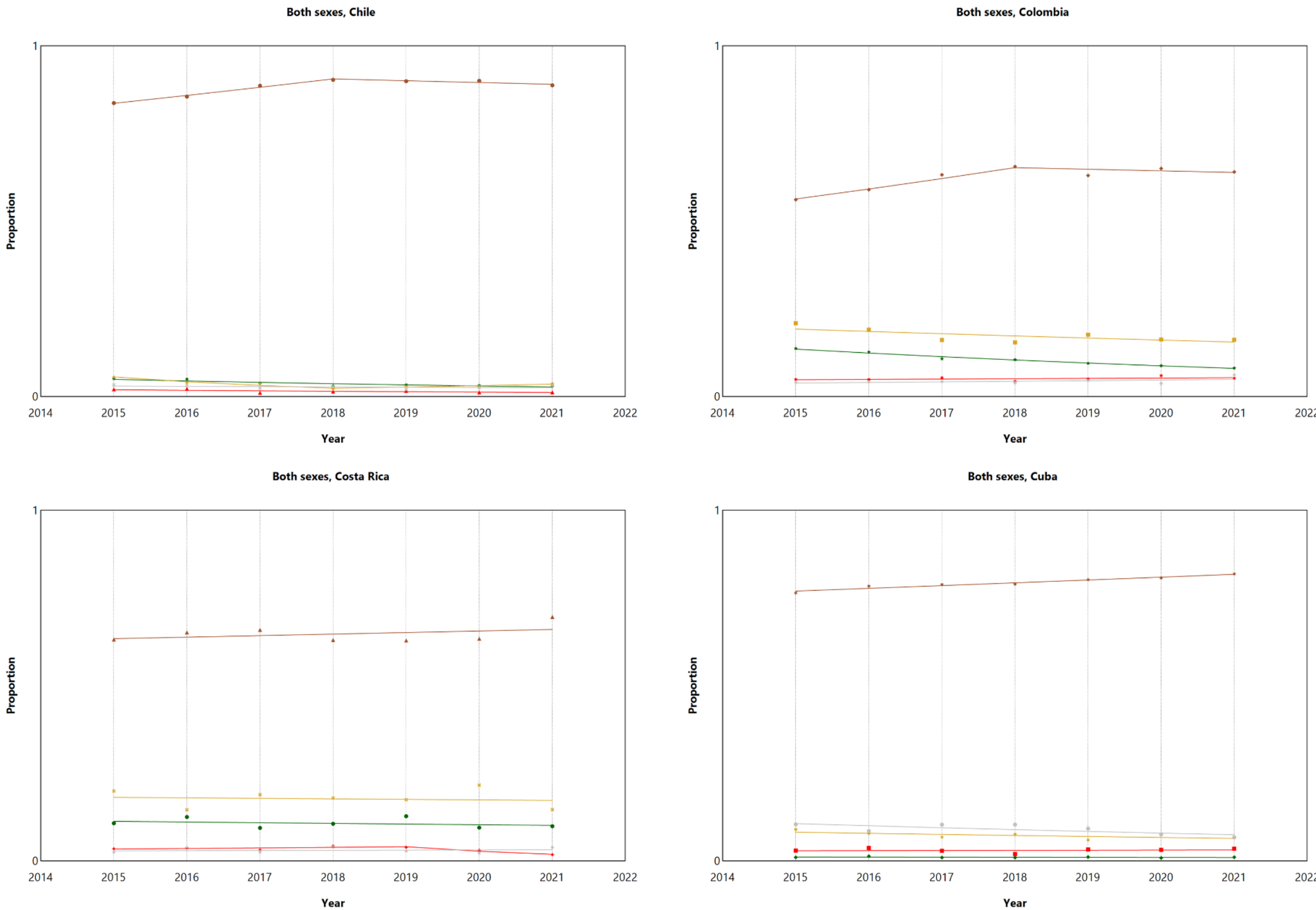

Both sexes, Chile
Both sexes, Colombia
Both sexes, Costa Rica
Both sexes, Cuba
Proportion
Year
1
0
2014
2015
2016
2017
2018
2019
2020
2021
2022

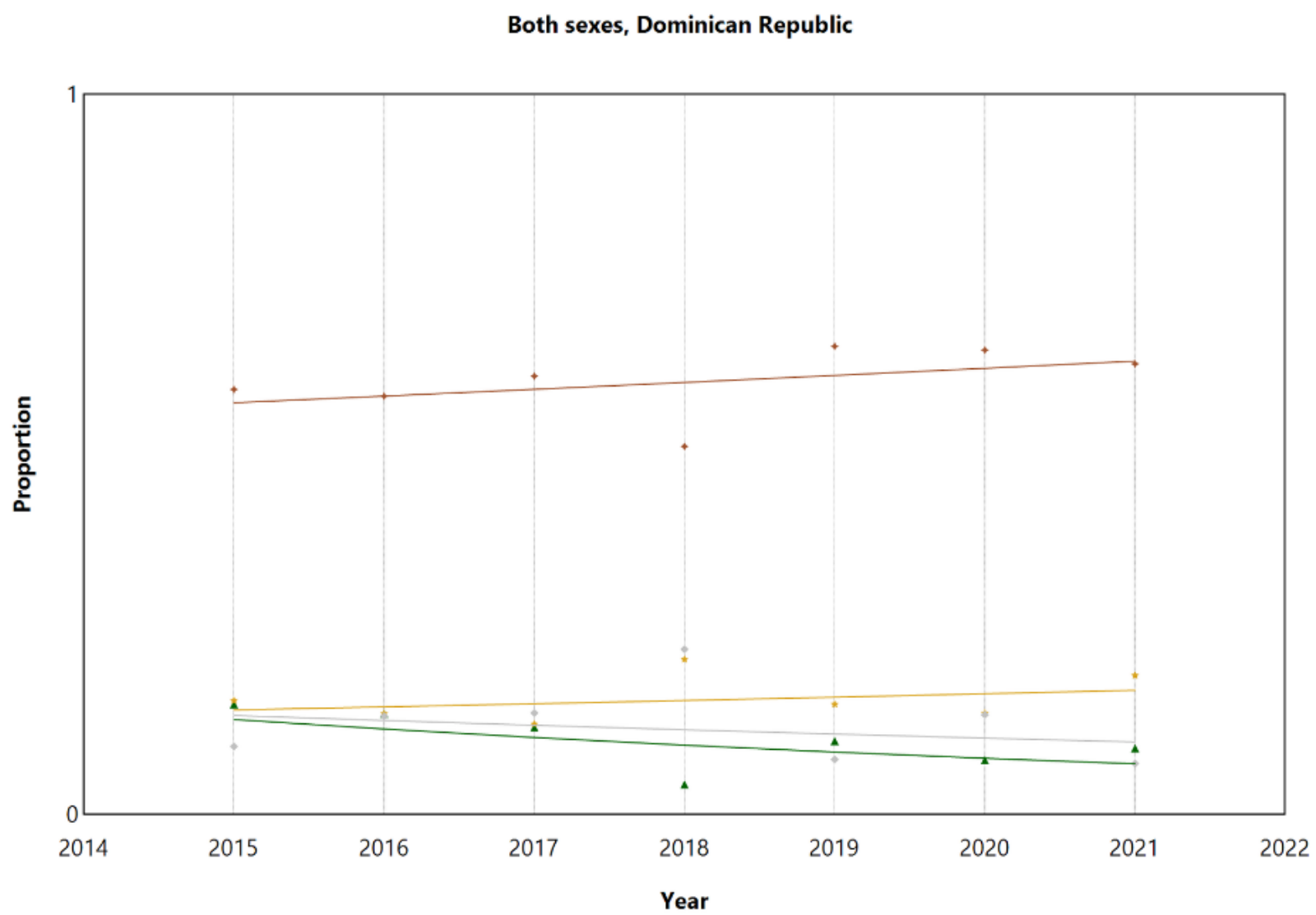
Both sexes, Dominican Republic
1
0
Proportion
2014
2015
2016
2017
2018
2019
2020
2021
2022
Year

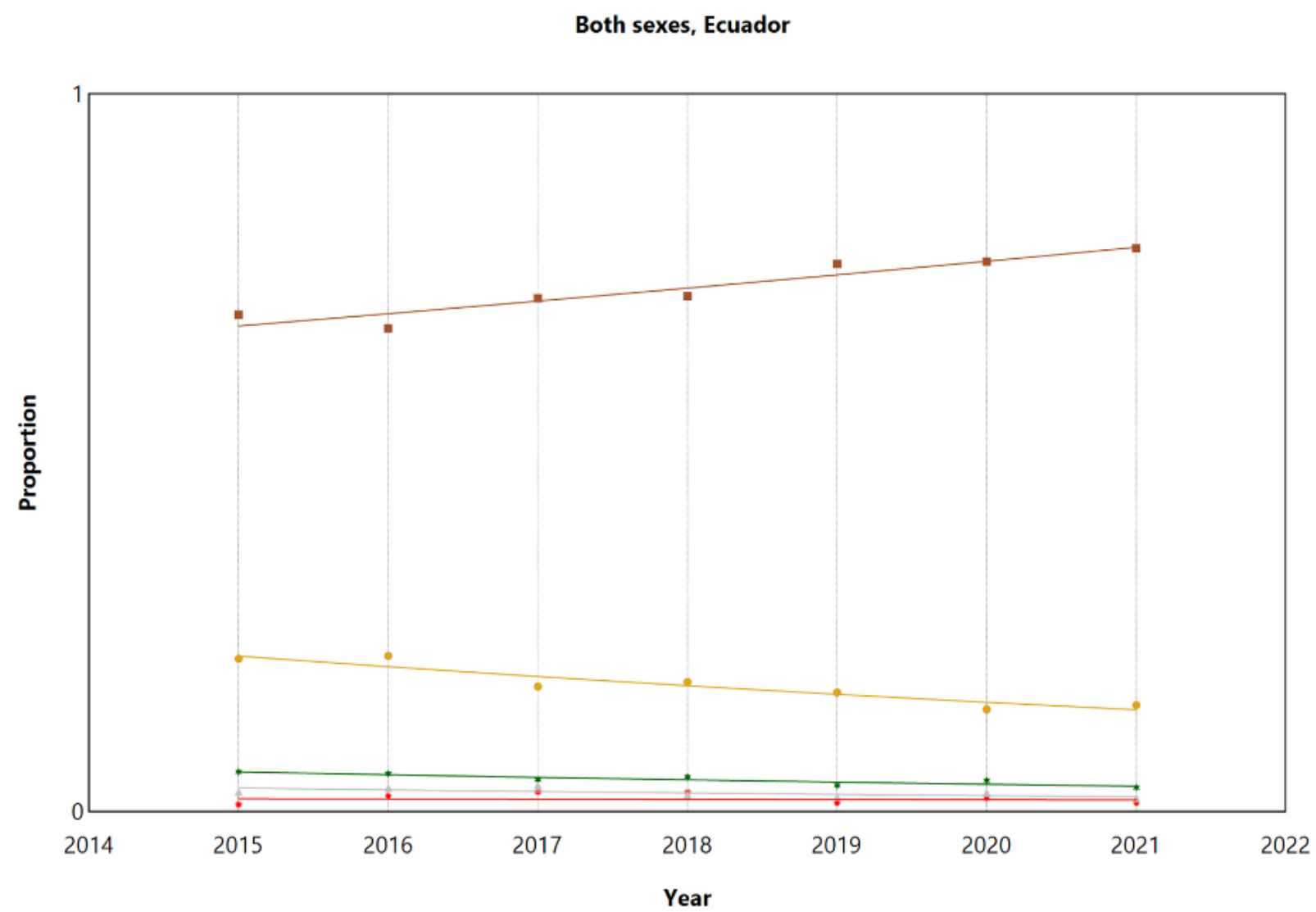
Both sexes, Ecuador
1
0
Proportion
2014
2015
2016
2017
2018
2019
2020
2021
2022
Year

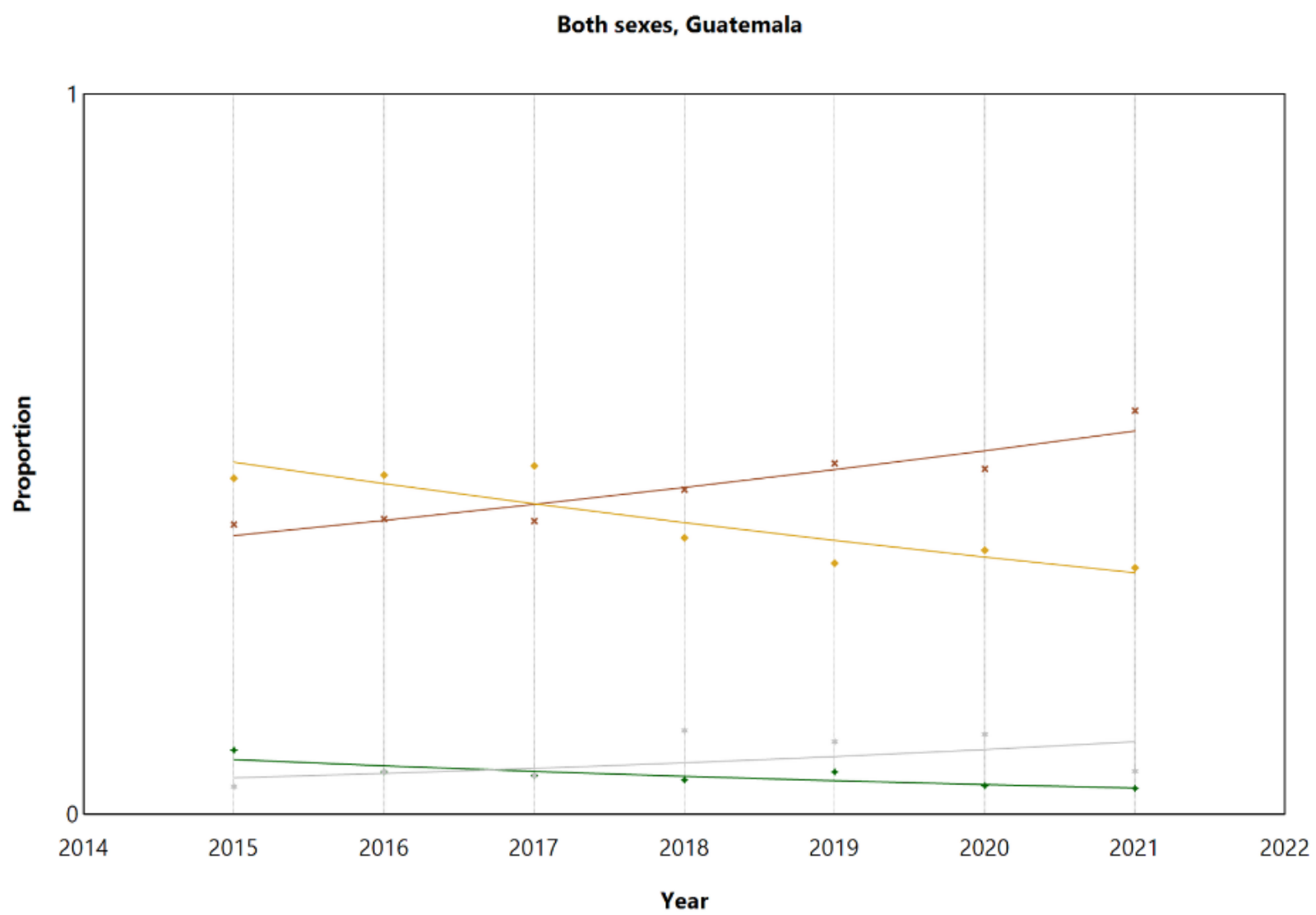
Both sexes, Guatemala
1
0
Proportion
2014
2015
2016
2017
2018
2019
2020
2021
2022
Year

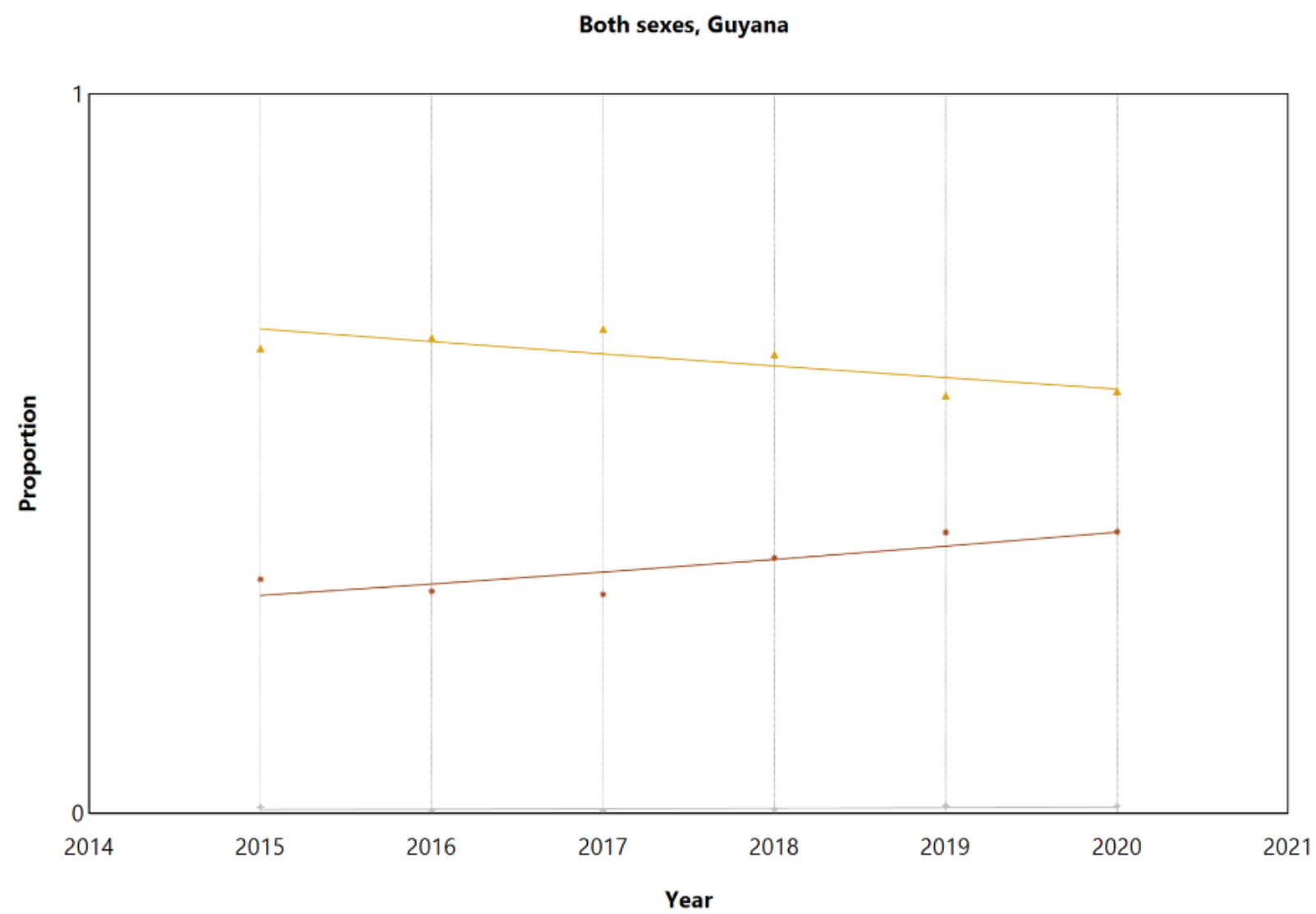
Both sexes, Guyana
1
0
Proportion
2014
2015
2016
2017
2018
2019
2020
2021
Year

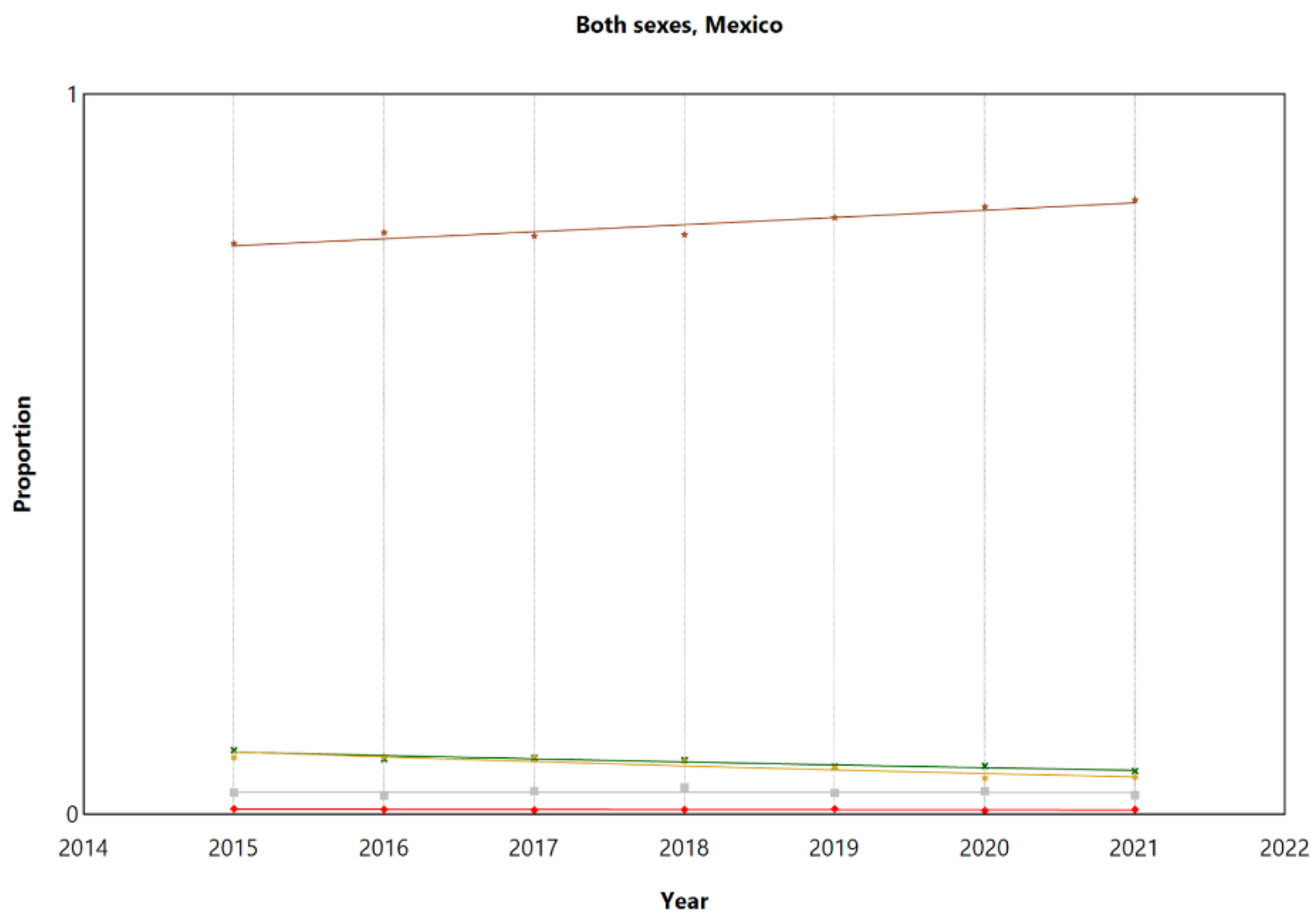
Both sexes, Mexico
Proportion
Year
0
1
2014
2015
2016
2017
2018
2019
2020
2021
2022

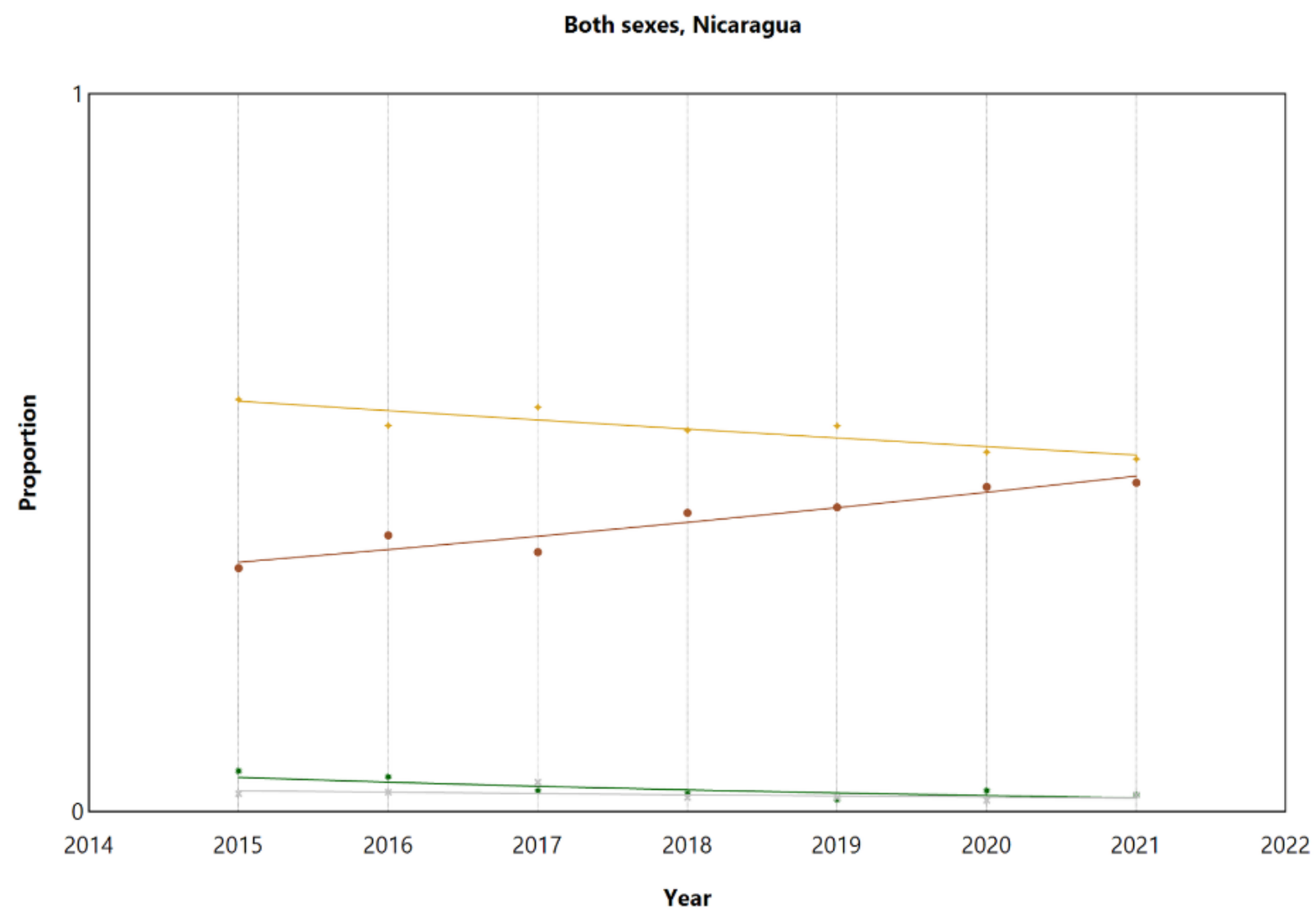
Both sexes, Nicaragua
Proportion
Year
0
1
2014
2015
2016
2017
2018
2019
2020
2021
2022

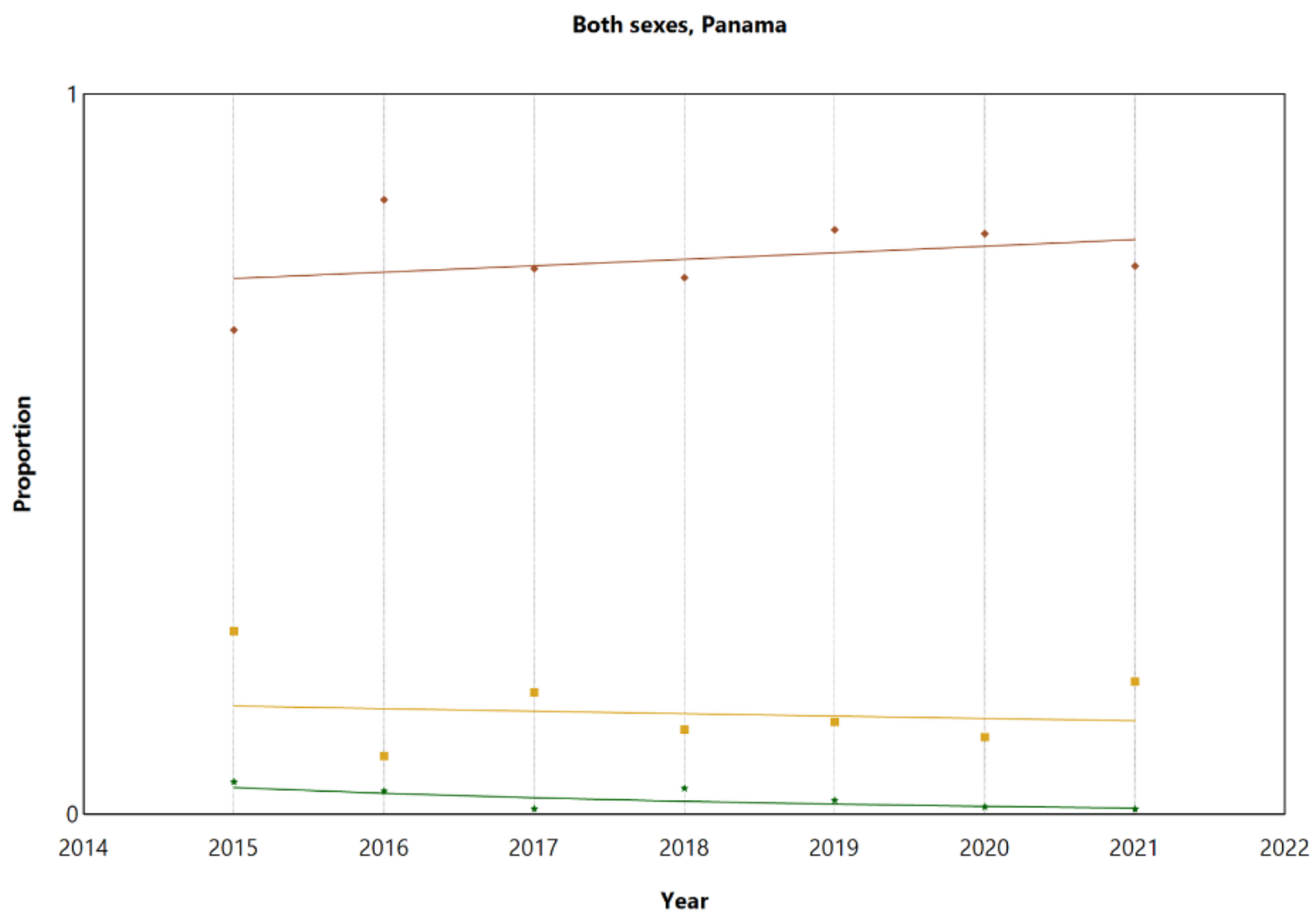
Both sexes, Panama
Proportion
Year
0
1
2014
2015
2016
2017
2018
2019
2020
2021
2022

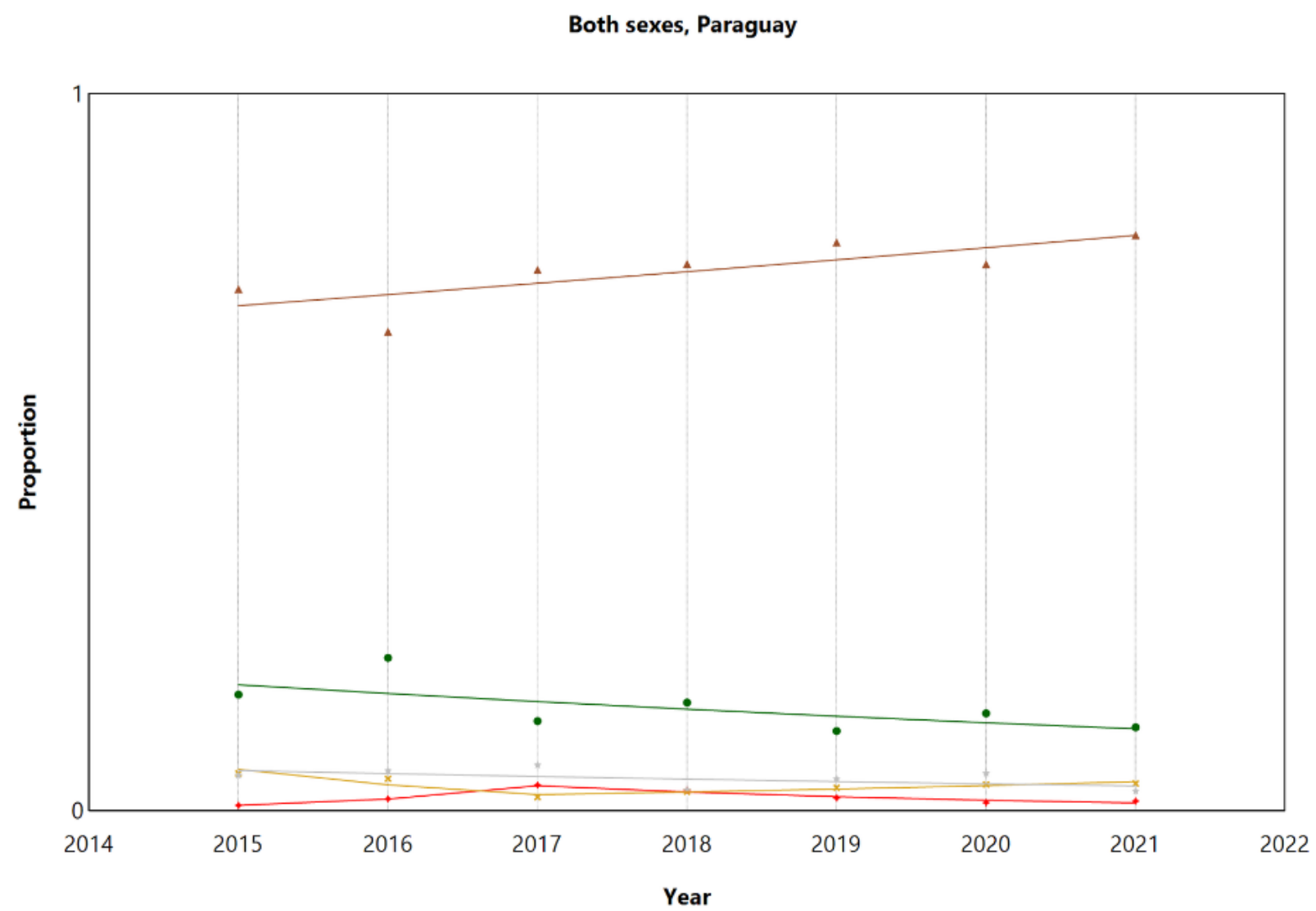
Both sexes, Paraguay
Proportion
Year
0
1
2014
2015
2016
2017
2018
2019
2020
2021
2022

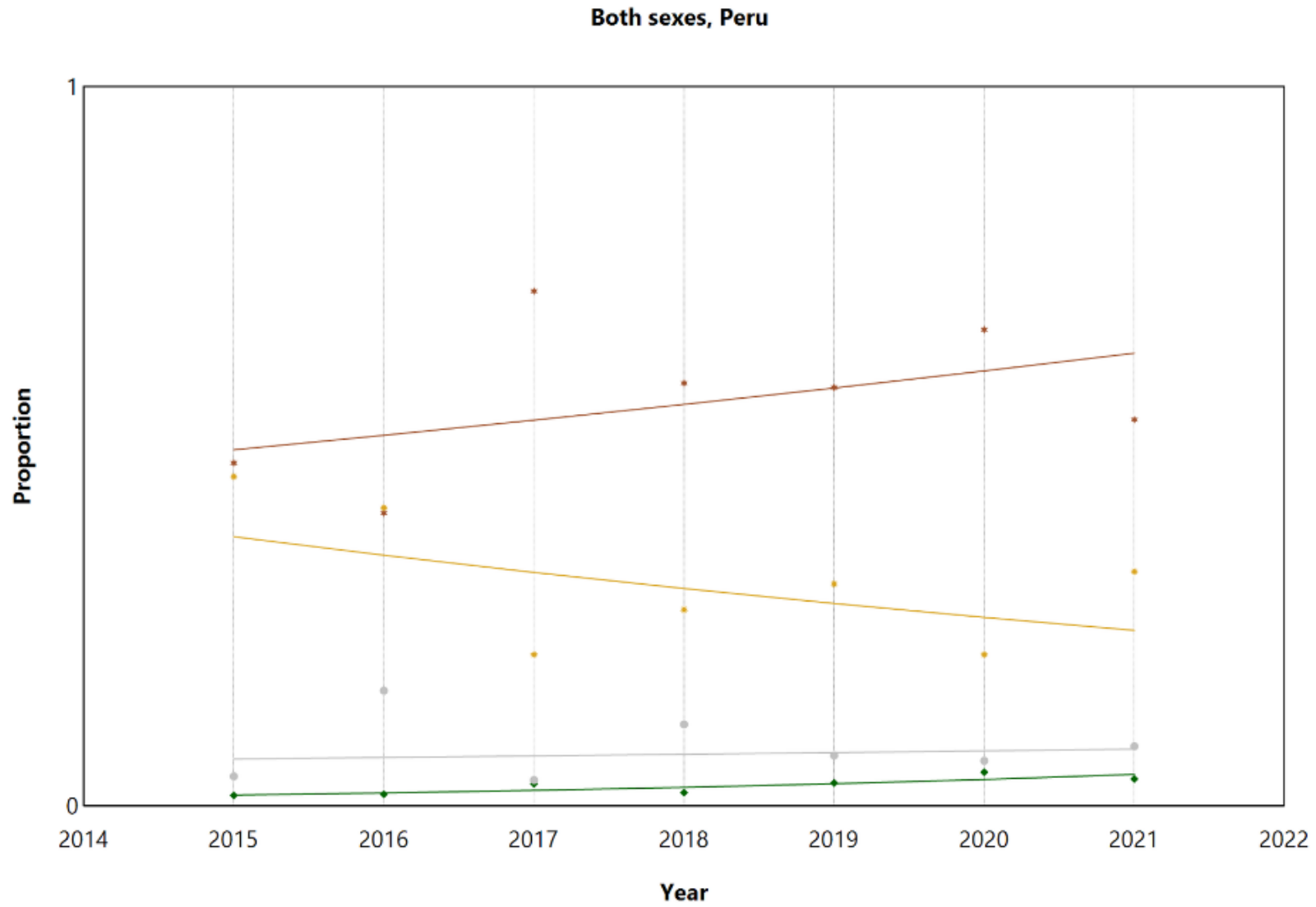
Both sexes, Peru
1
0
Proportion
2014
2015
2016
2017
2018
2019
2020
2021
2022
Year

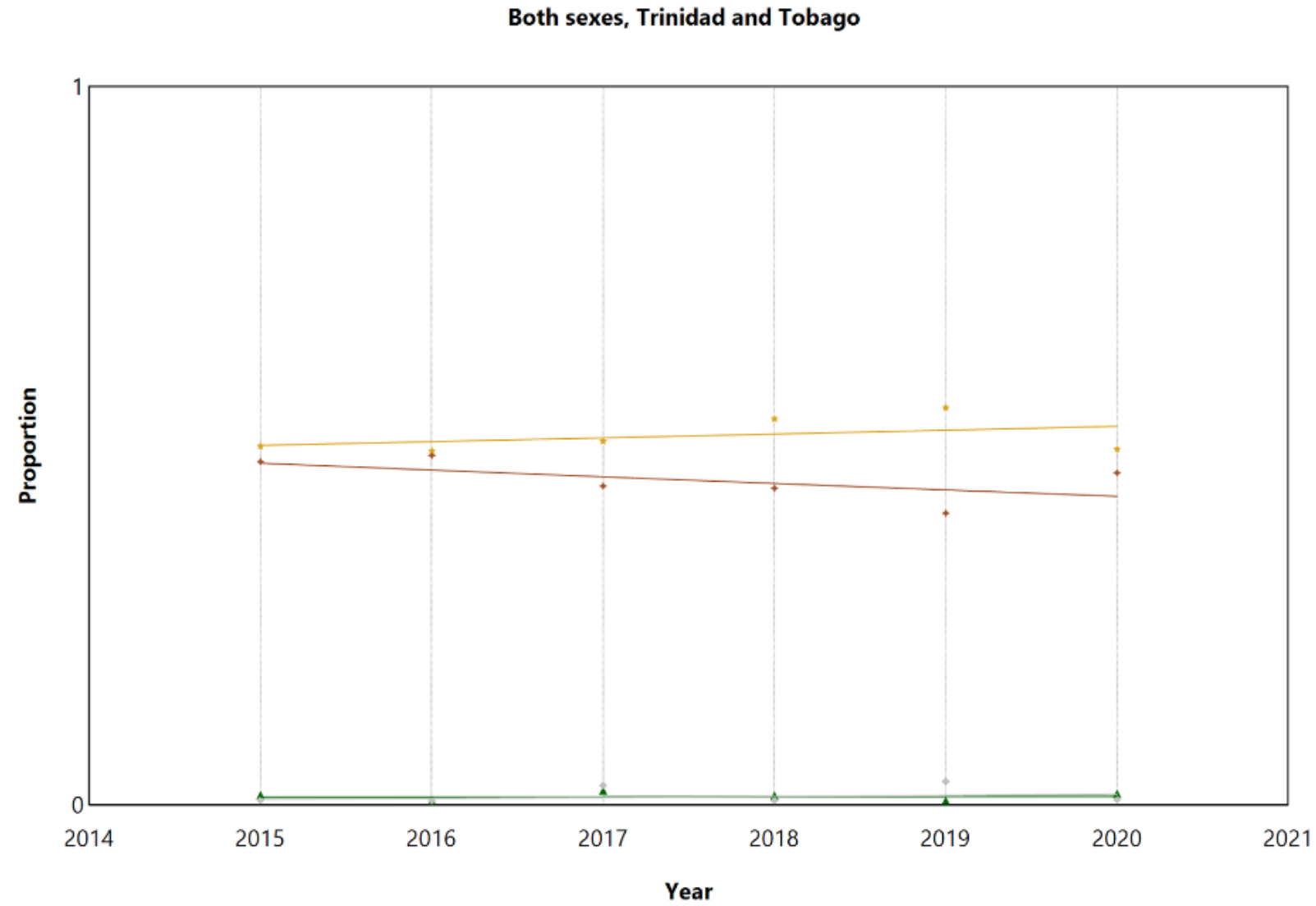
Both sexes, Trinidad and Tobago
1
0
Proportion
2014
2015
2016
2017
2018
2019
2020
2021
Year

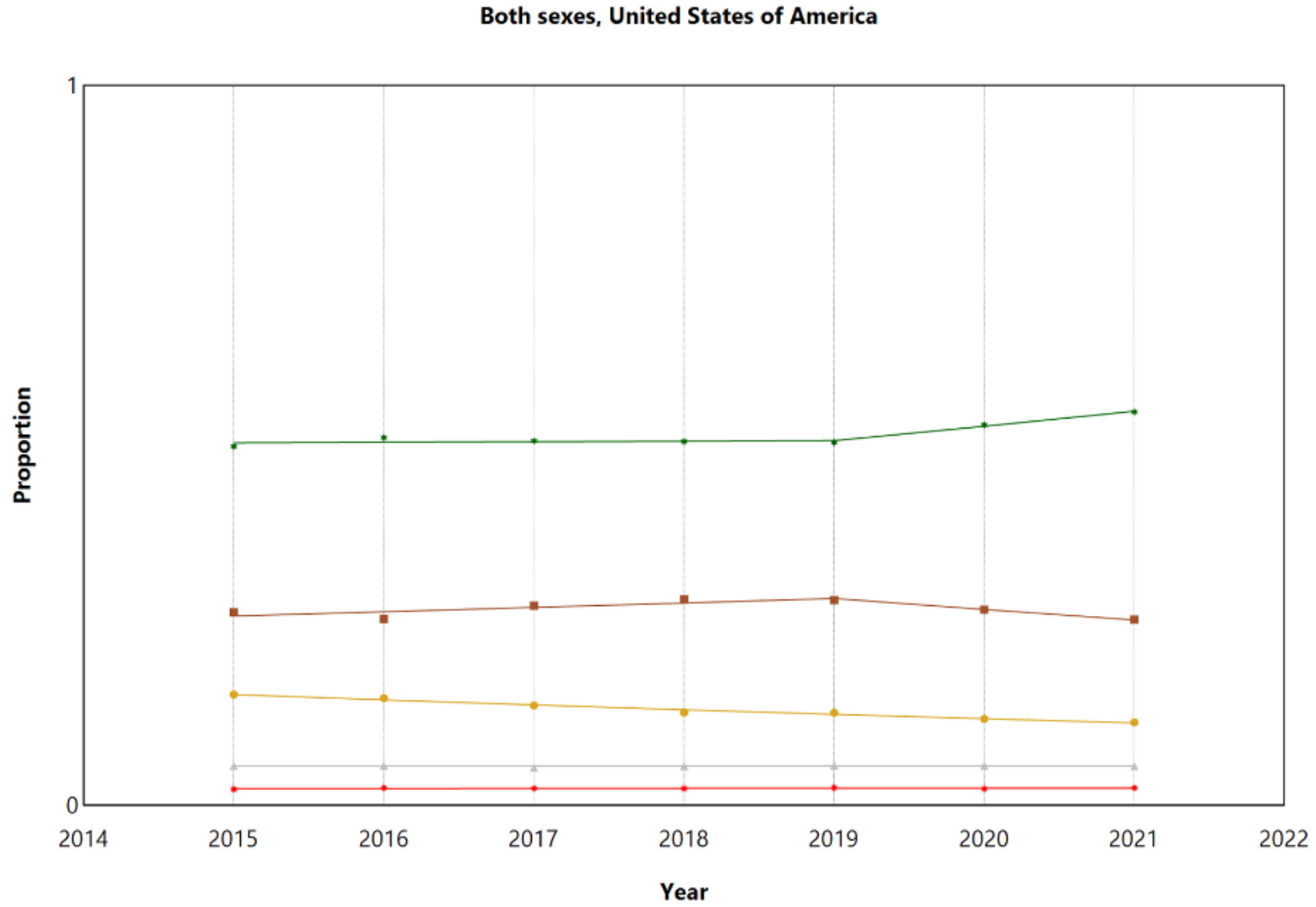
Both sexes, United States of America
1
0
Proportion
2014
2015
2016
2017
2018
2019
2020
2021
2022
Year

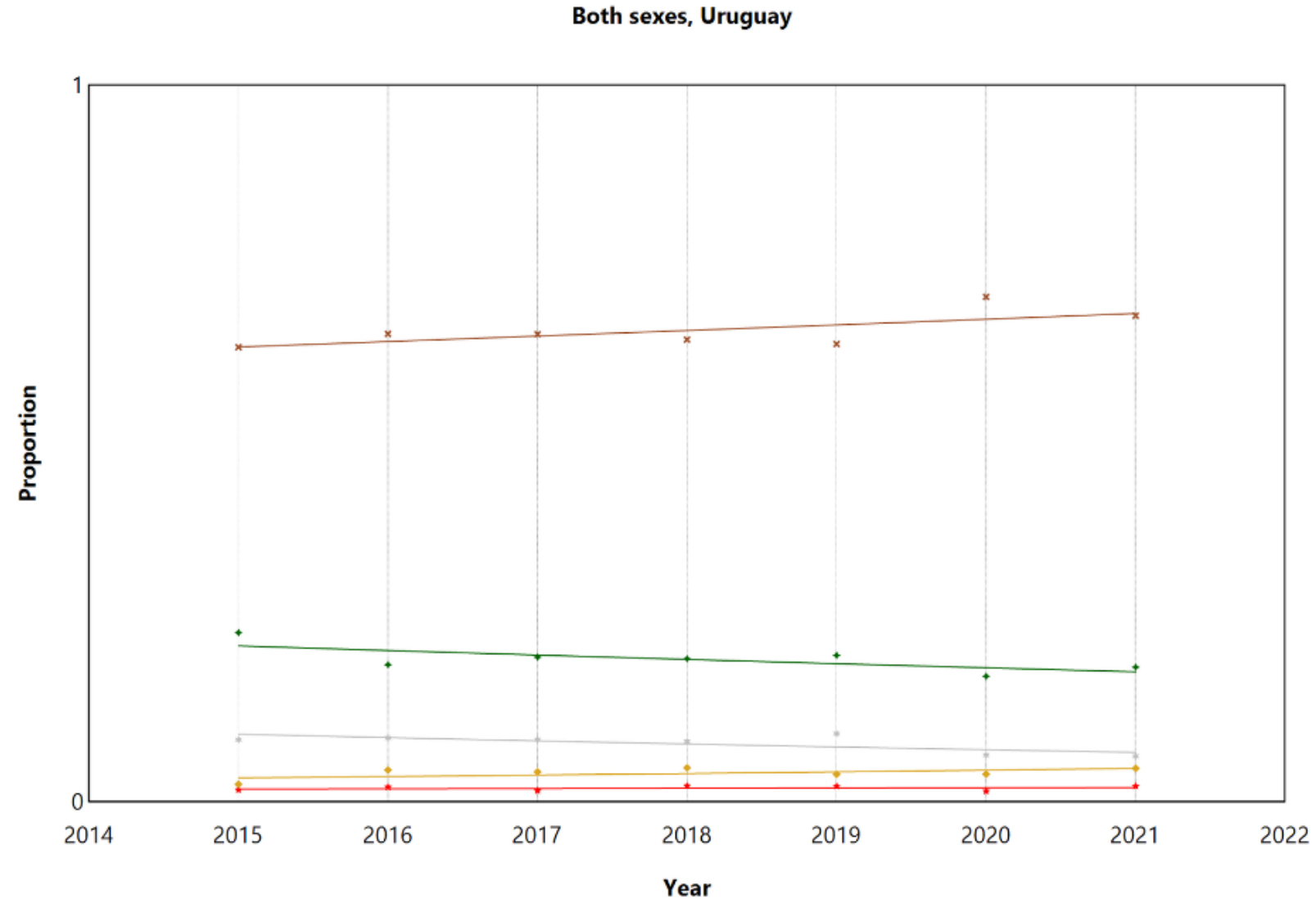
Both sexes, Uruguay
1
0
Proportion
2014
2015
2016
2017
2018
2019
2020
2021
2022
Year

(B)

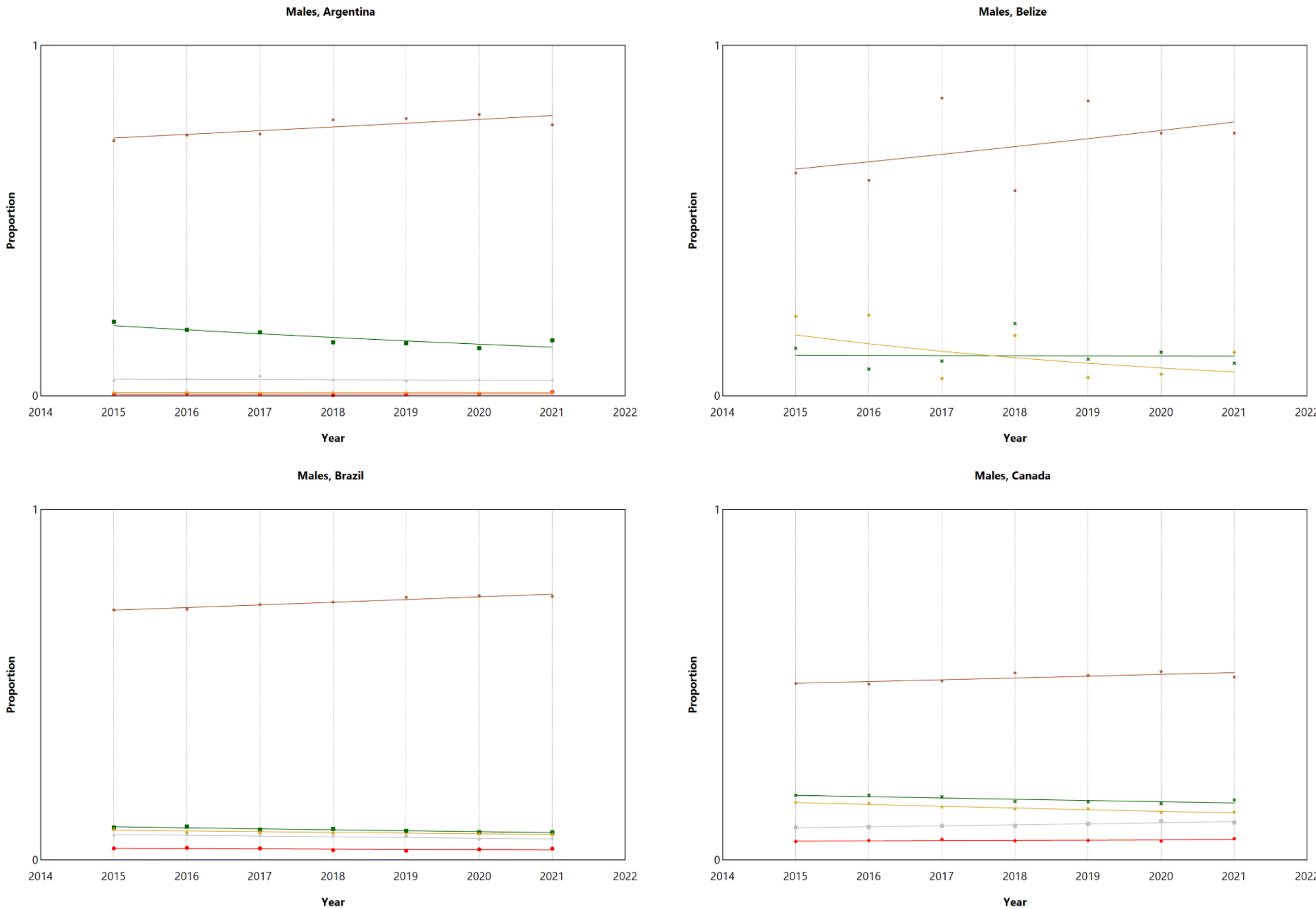

Males, Argentina
Males, Belize
Males, Brazil
Males, Canada
Proportion
Year
0
1
2014
2015
2016
2017
2018
2019
2020
2021
2022

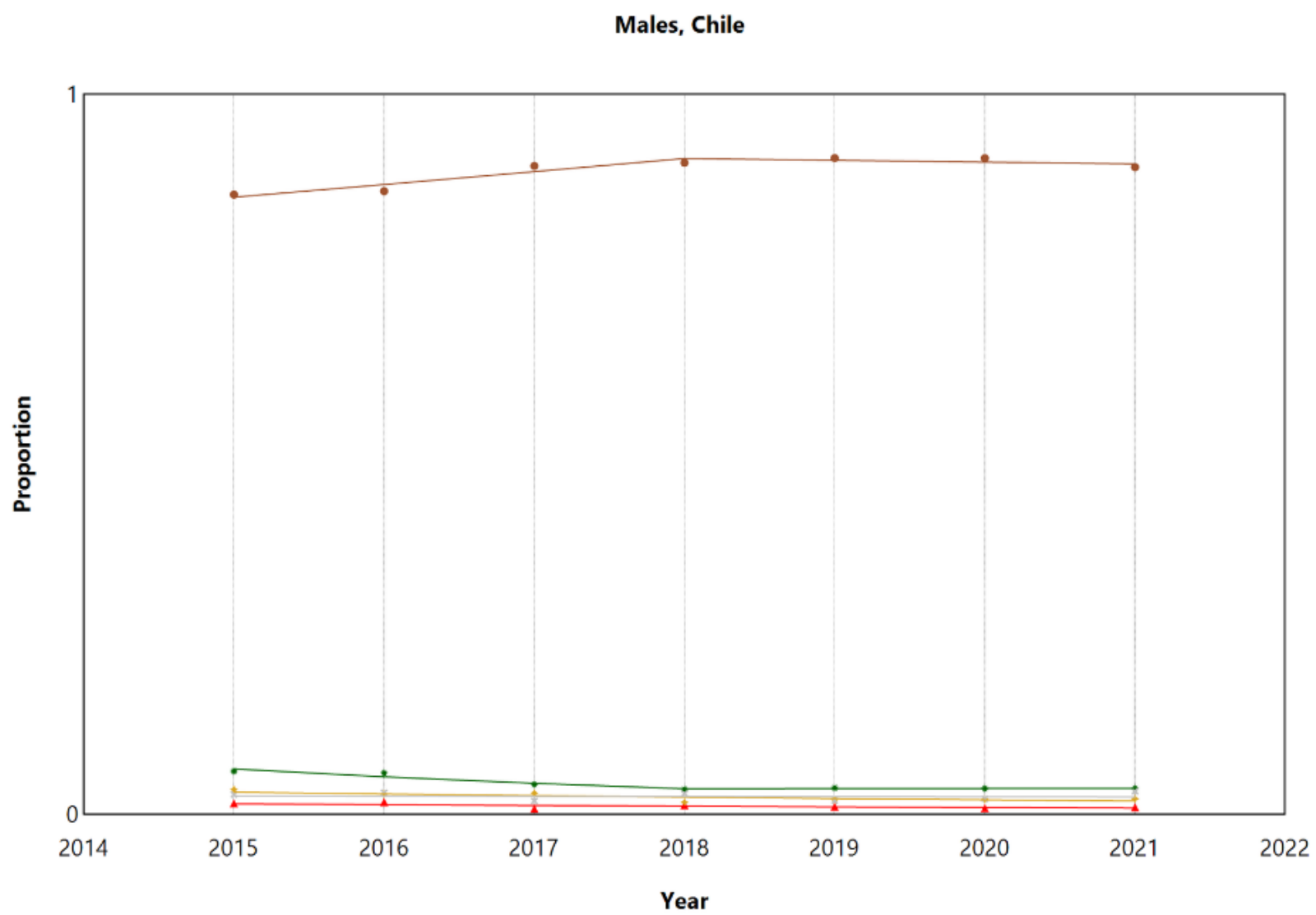
Males, Chile
Proportion
Year
0
1
2014
2015
2016
2017
2018
2019
2020
2021
2022

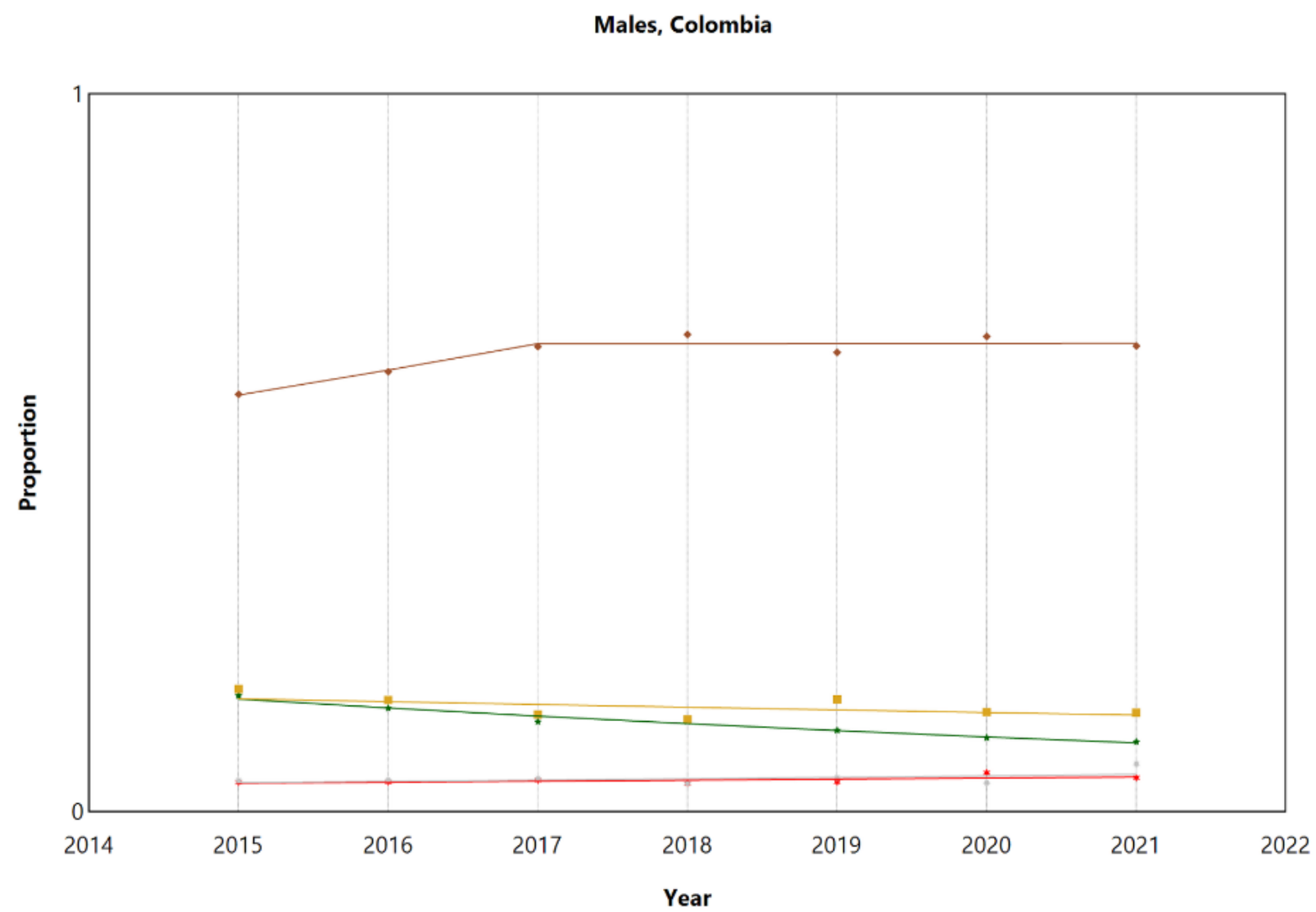
Males, Colombia
Proportion
Year
0
1
2014
2015
2016
2017
2018
2019
2020
2021
2022

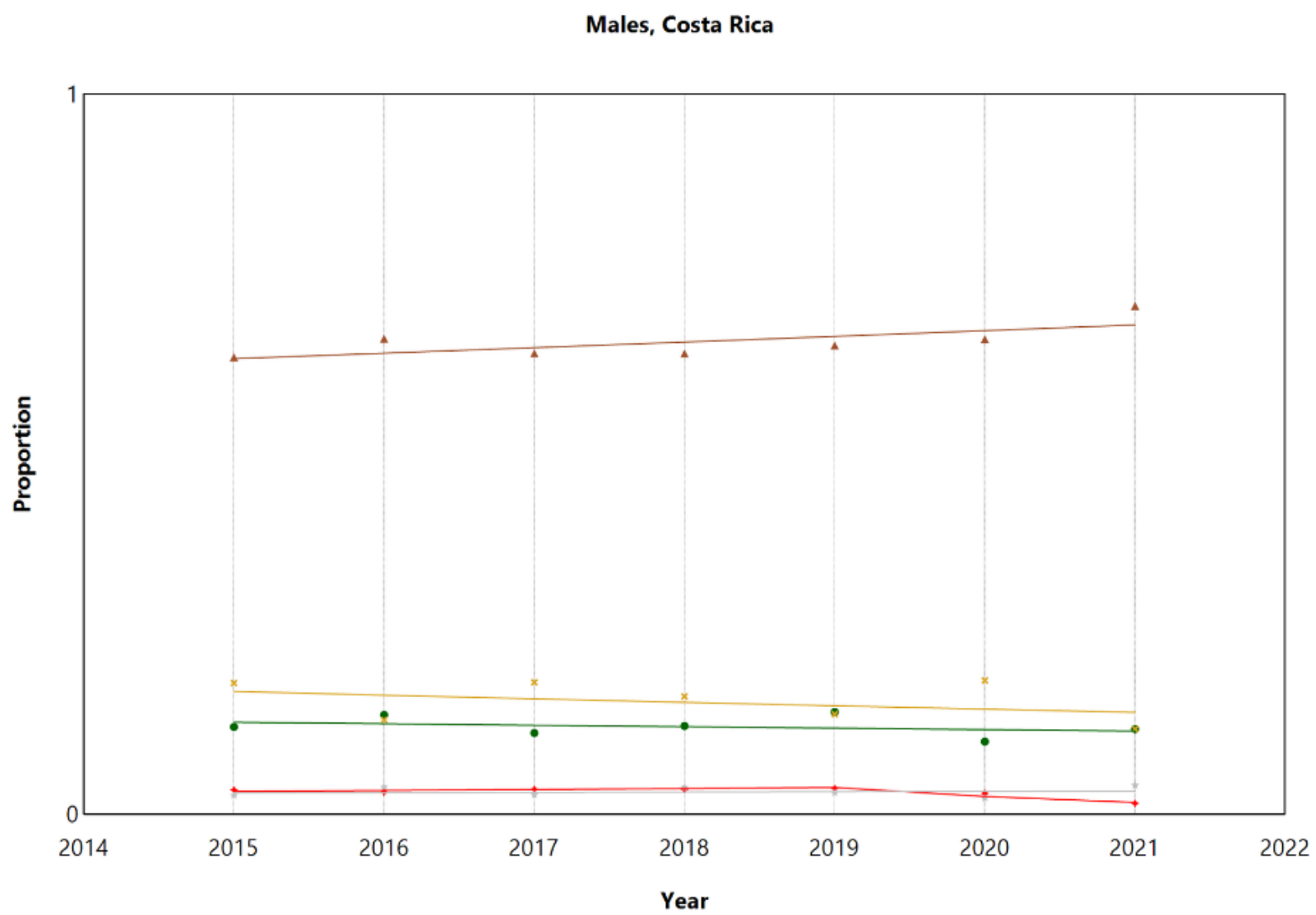
Males, Costa Rica
Proportion
Year
0
1
2014
2015
2016
2017
2018
2019
2020
2021
2022

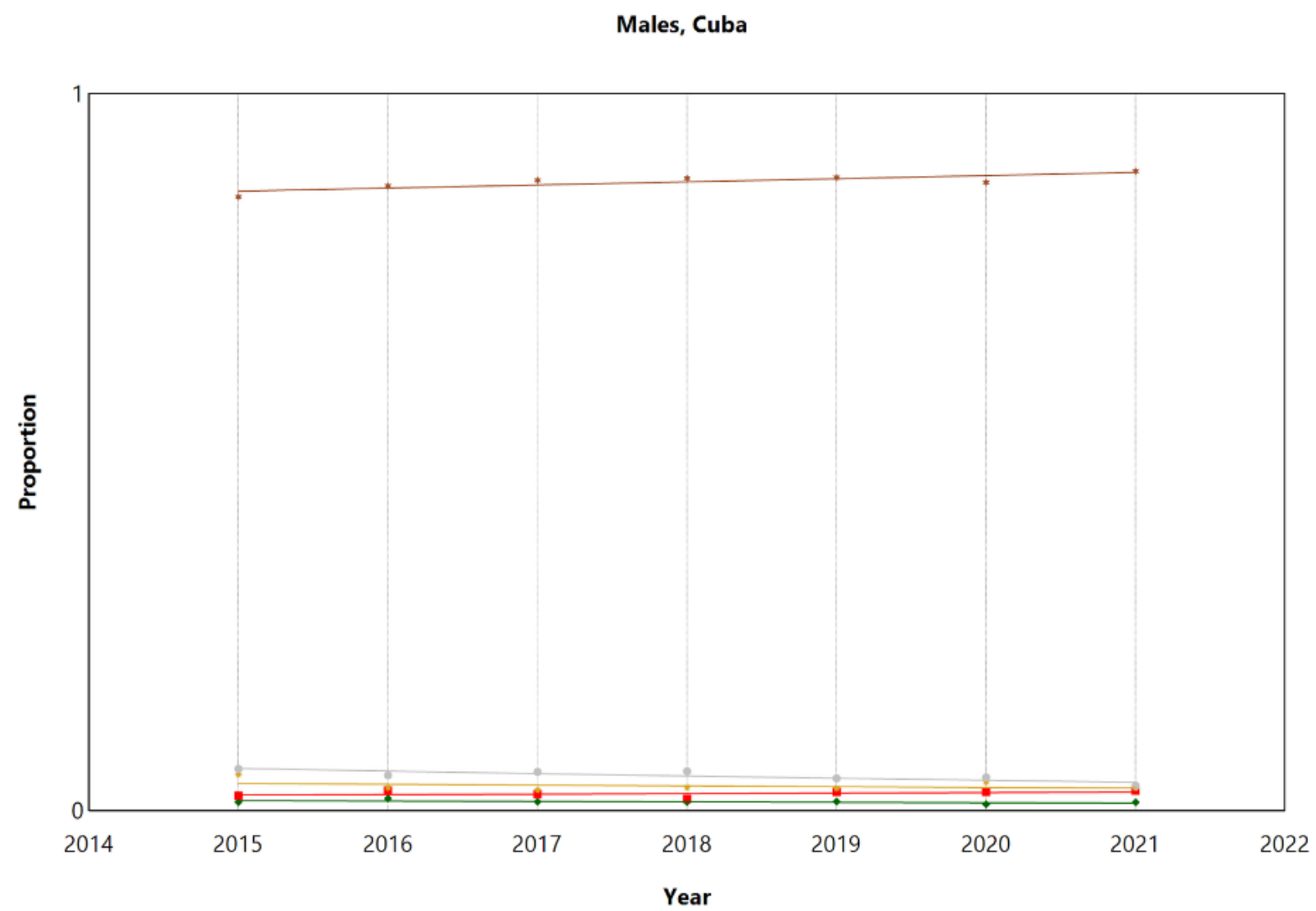
Males, Cuba
Proportion
Year
0
1
2014
2015
2016
2017
2018
2019
2020
2021
2022

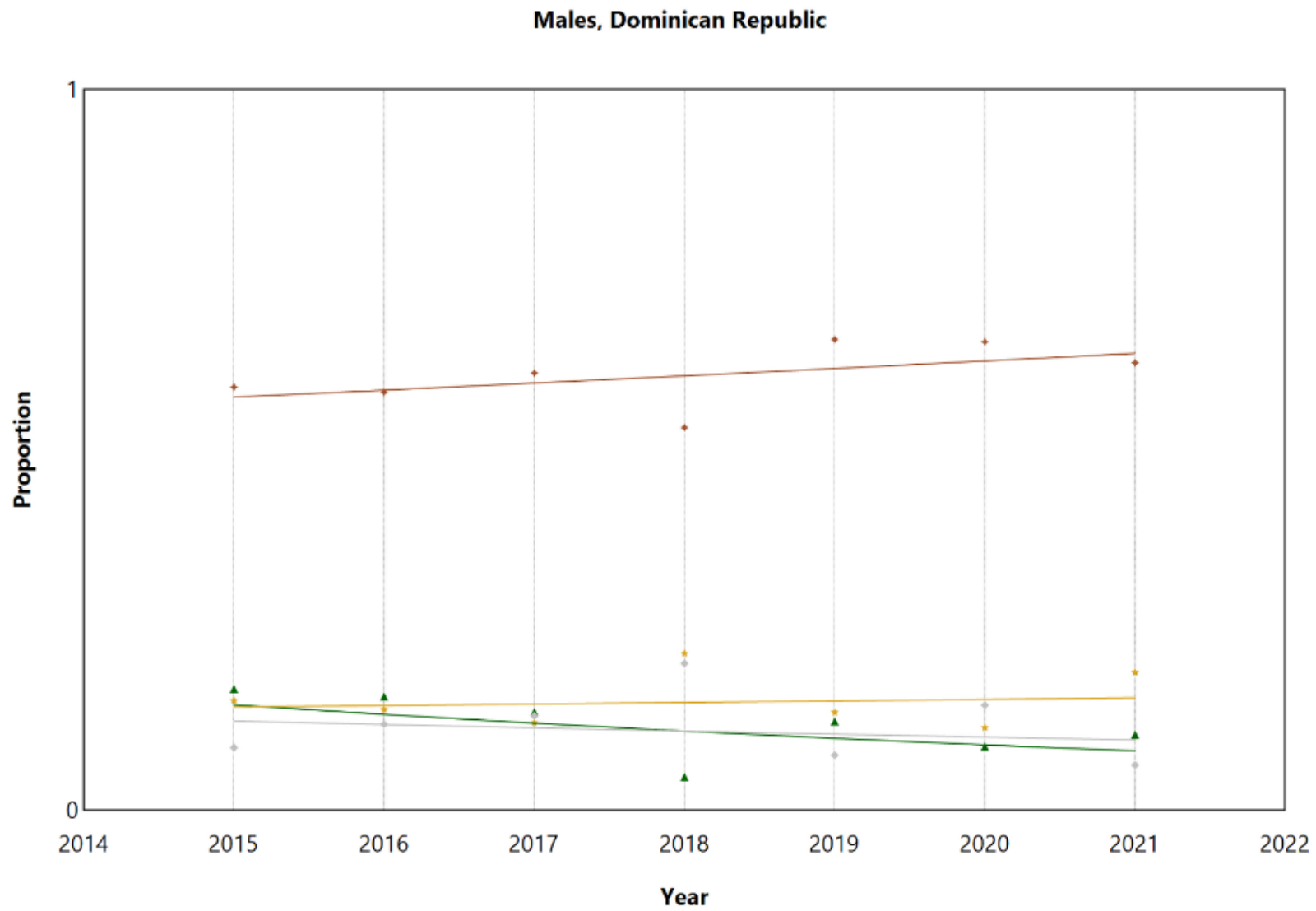

Males, Dominican Republic
Proportion
Year

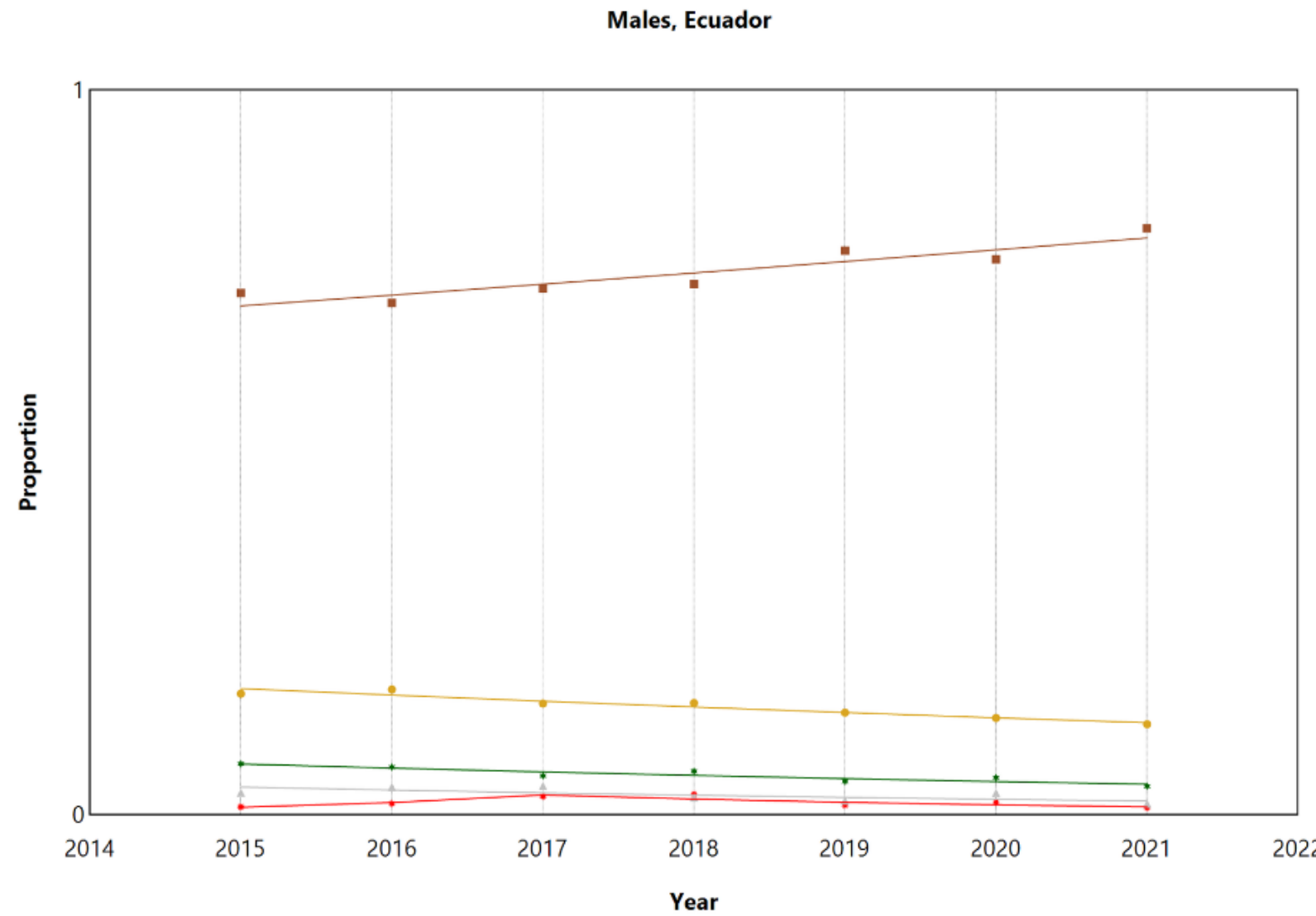

Males, Ecuador
Proportion
Year

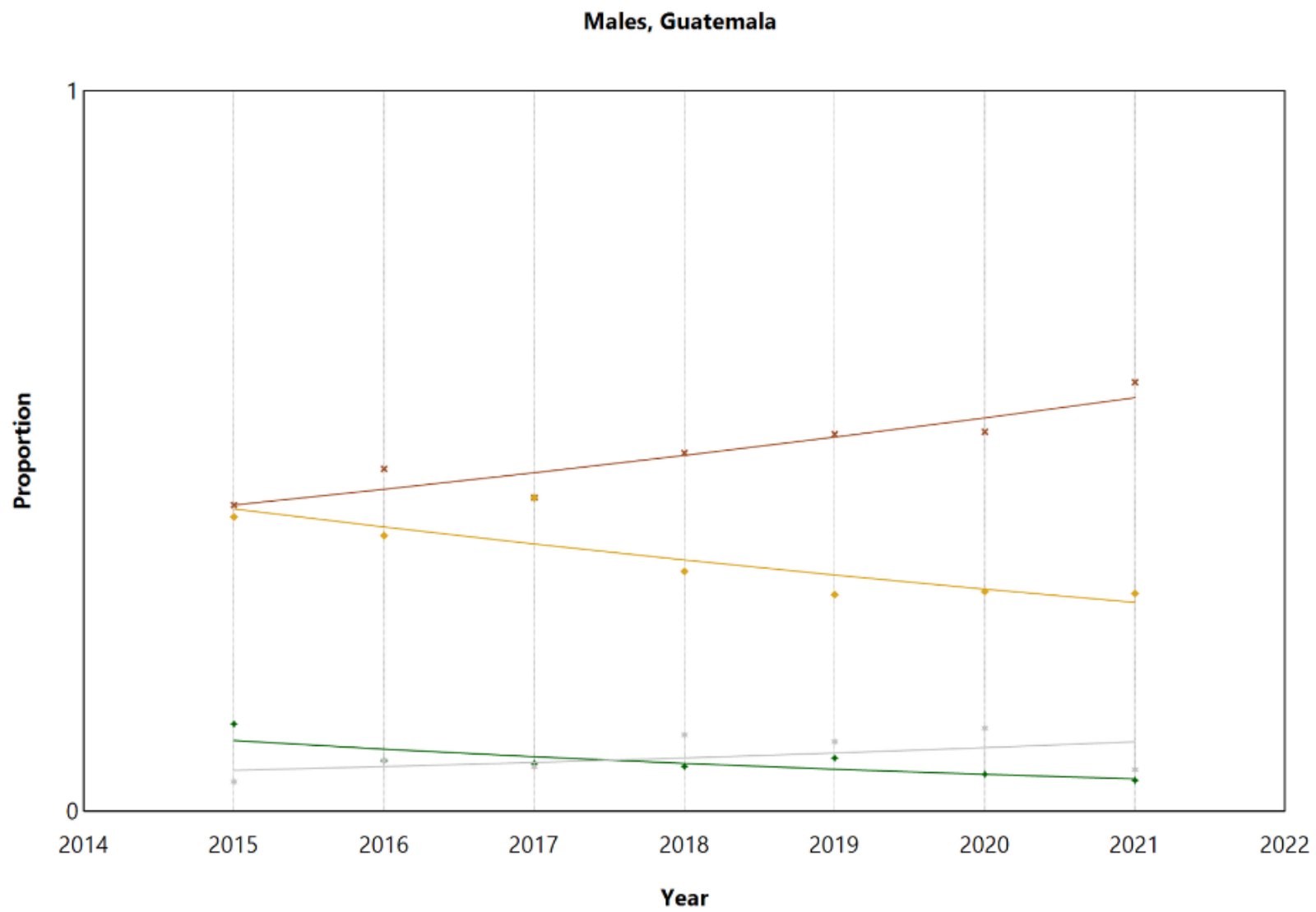

Males, Guatemala
Proportion
Year

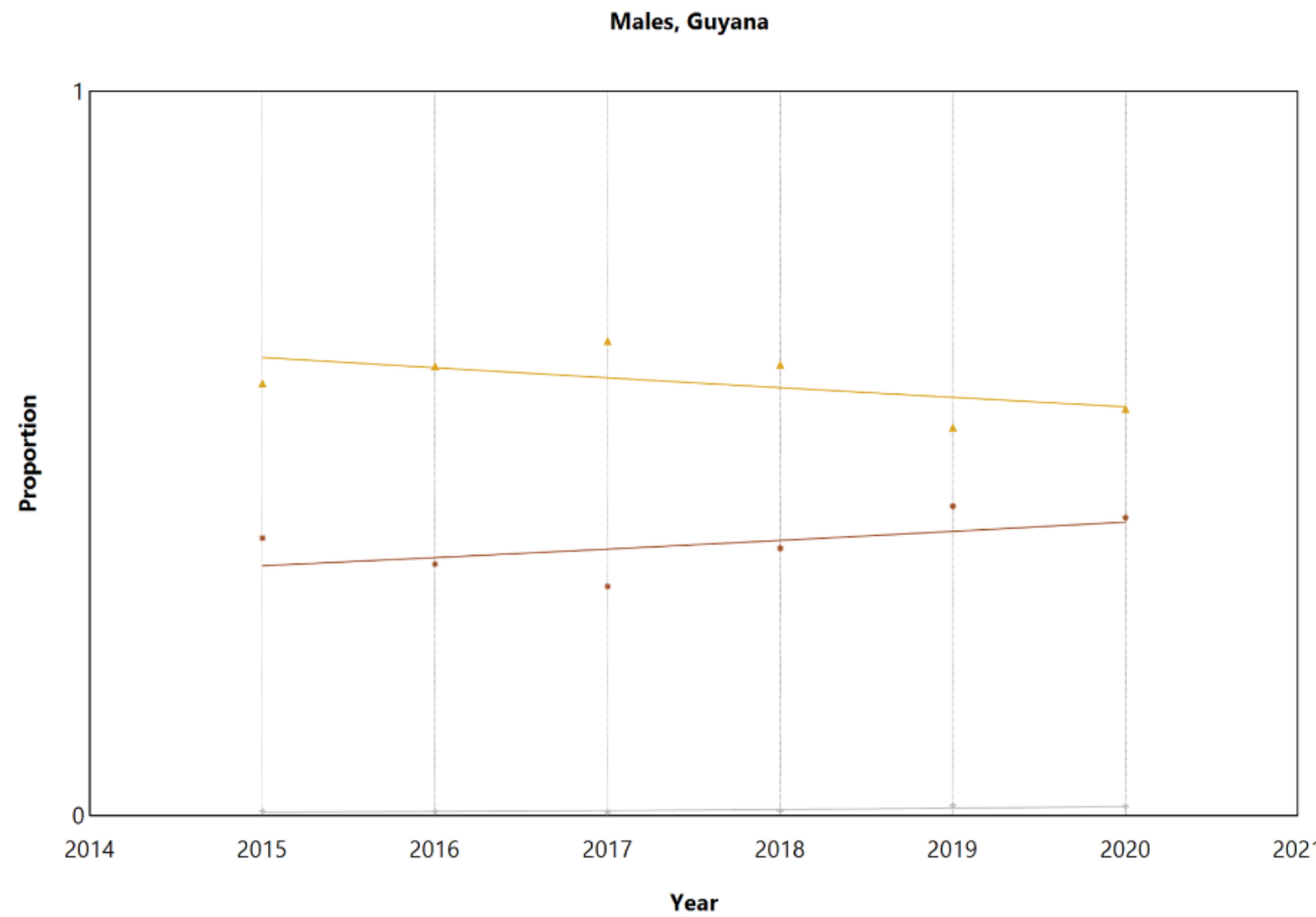

Males, Guyana
Proportion
Year

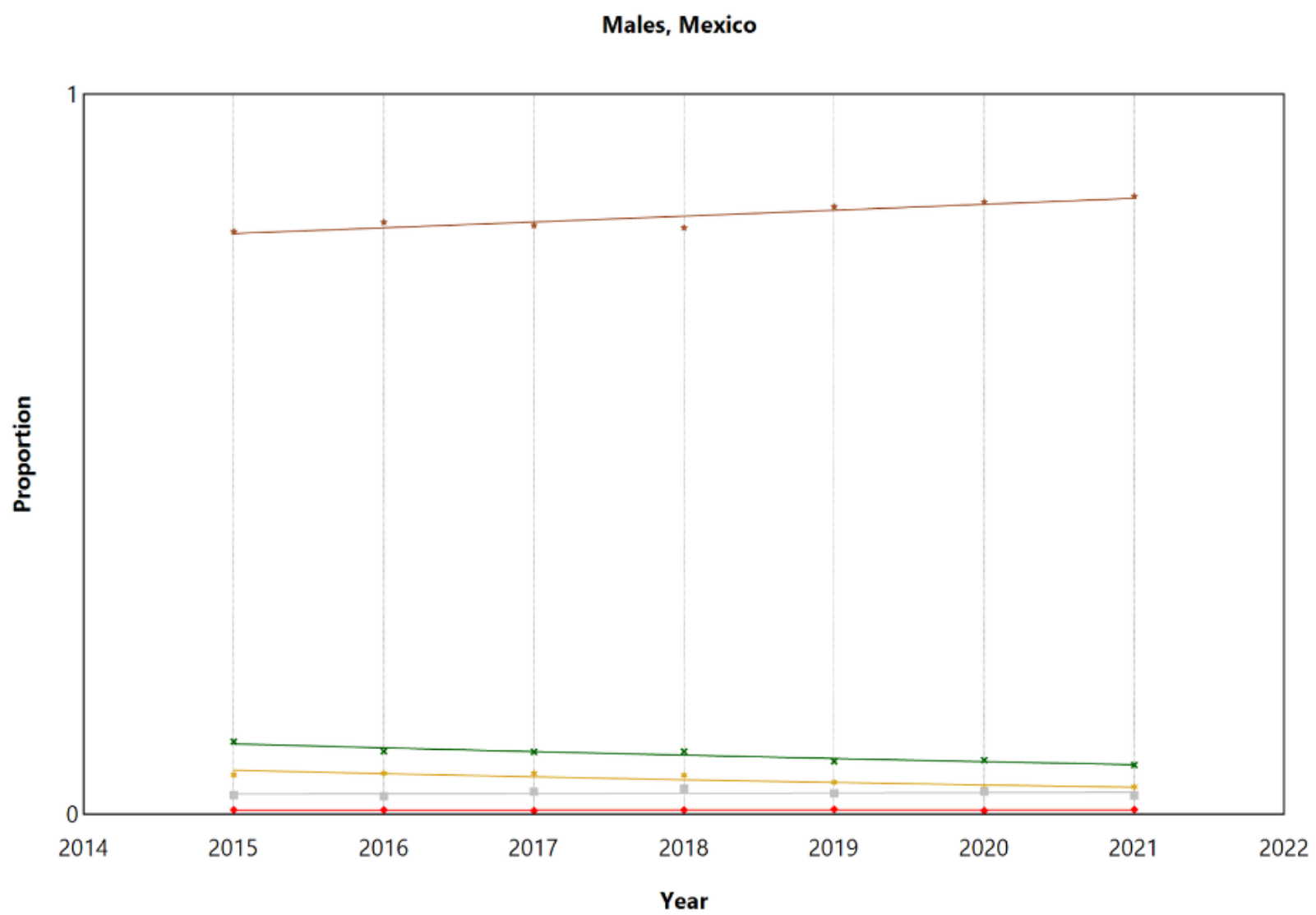
Males, Mexico
1
0
Proportion
2014
2015
2016
2017
2018
2019
2020
2021
2022
Year

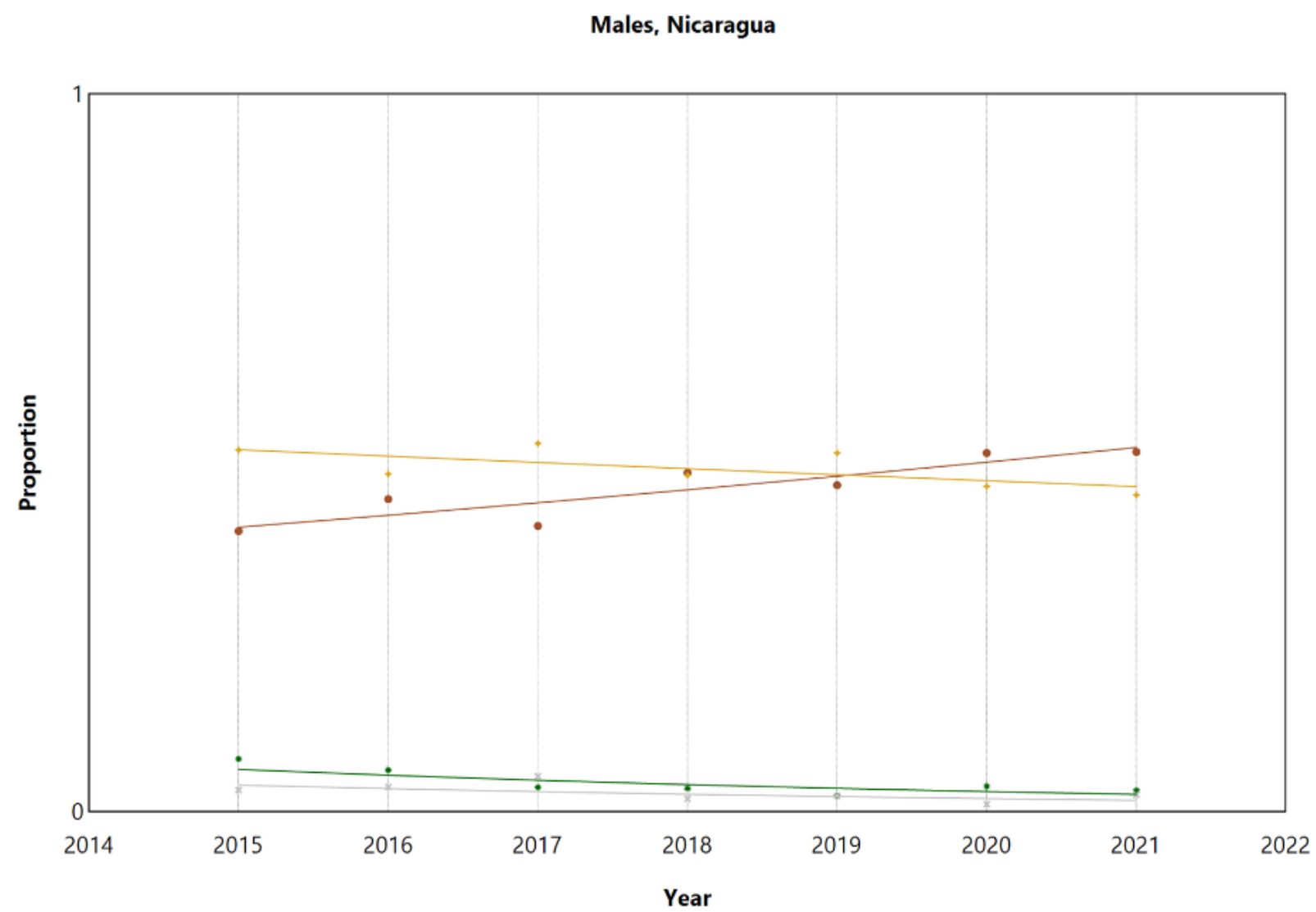
Males, Nicaragua
1
0
Proportion
2014
2015
2016
2017
2018
2019
2020
2021
2022
Year

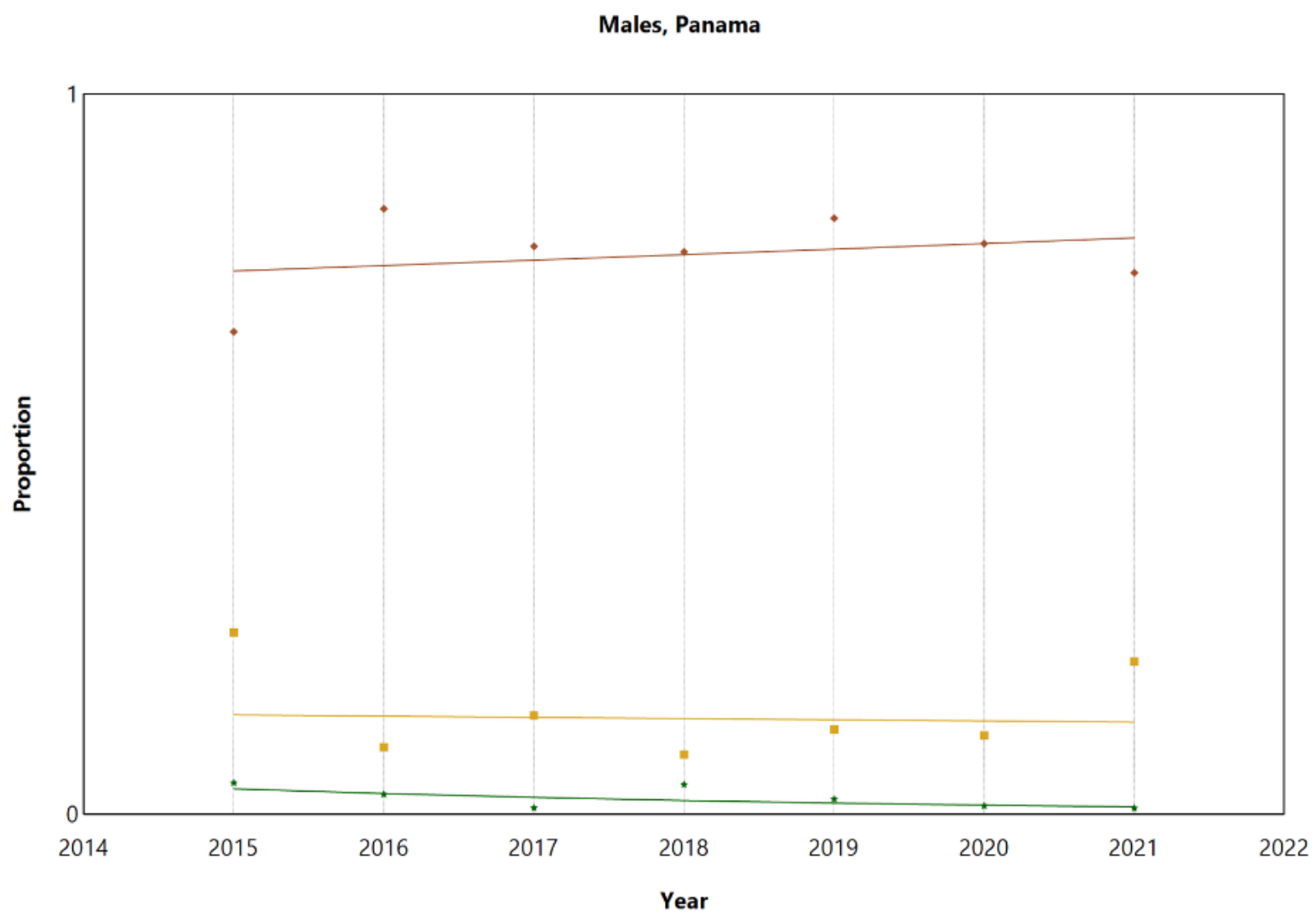
Males, Panama
1
0
Proportion
2014
2015
2016
2017
2018
2019
2020
2021
2022
Year

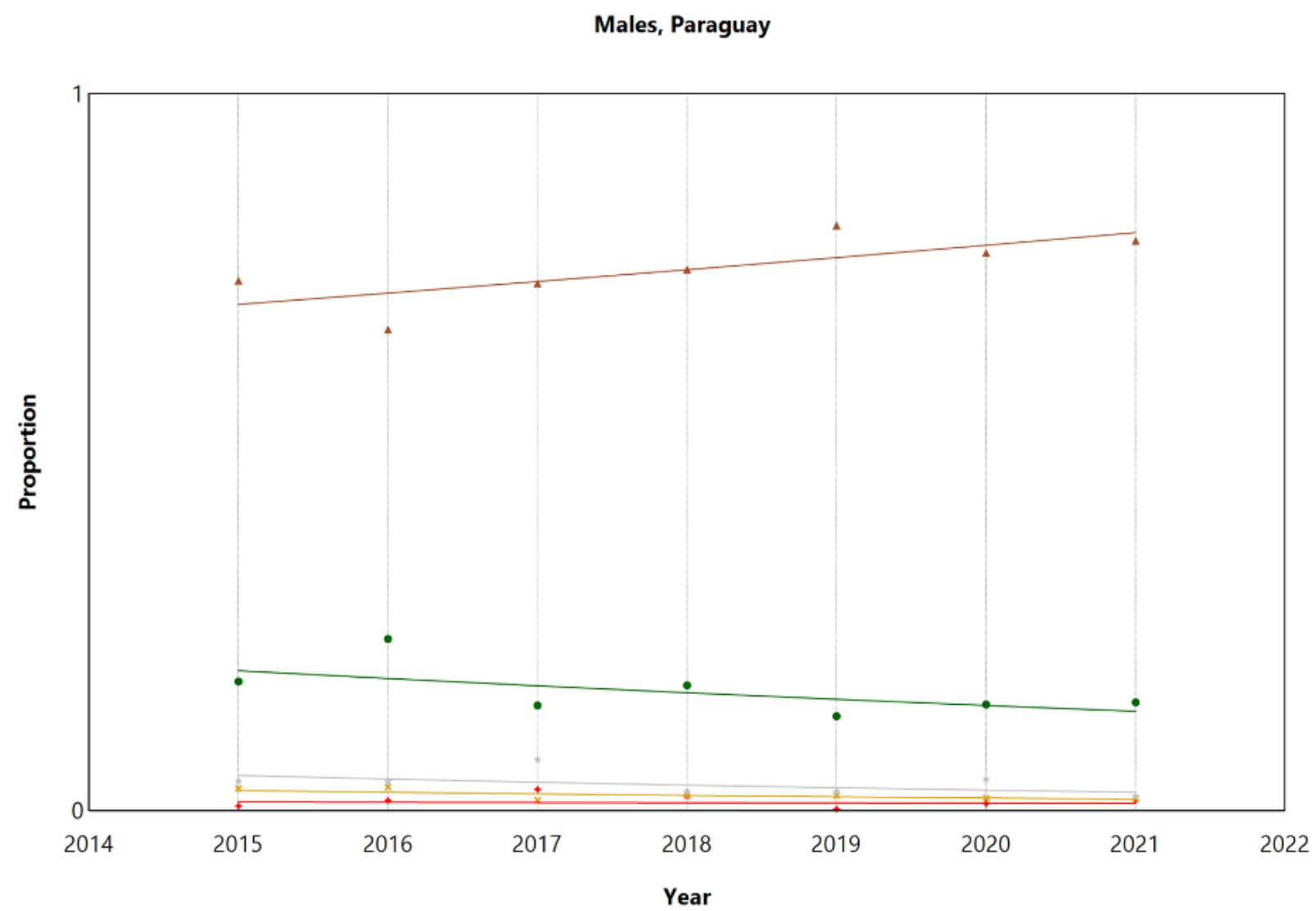
Males, Paraguay
1
0
Proportion
2014
2015
2016
2017
2018
2019
2020
2021
2022
Year

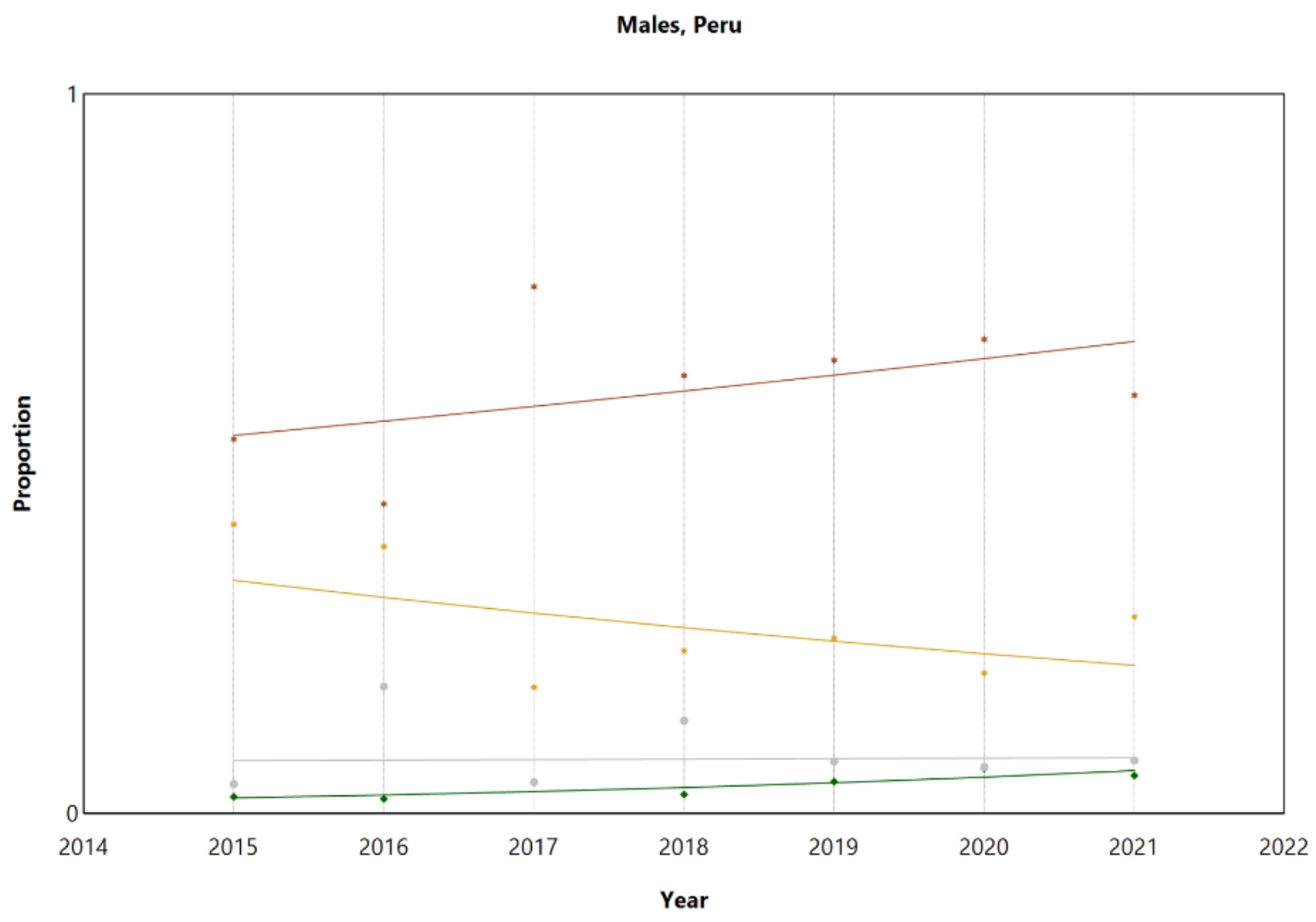
Males, Peru
1
0
Proportion
2014
2015
2016
2017
2018
2019
2020
2021
2022
Year

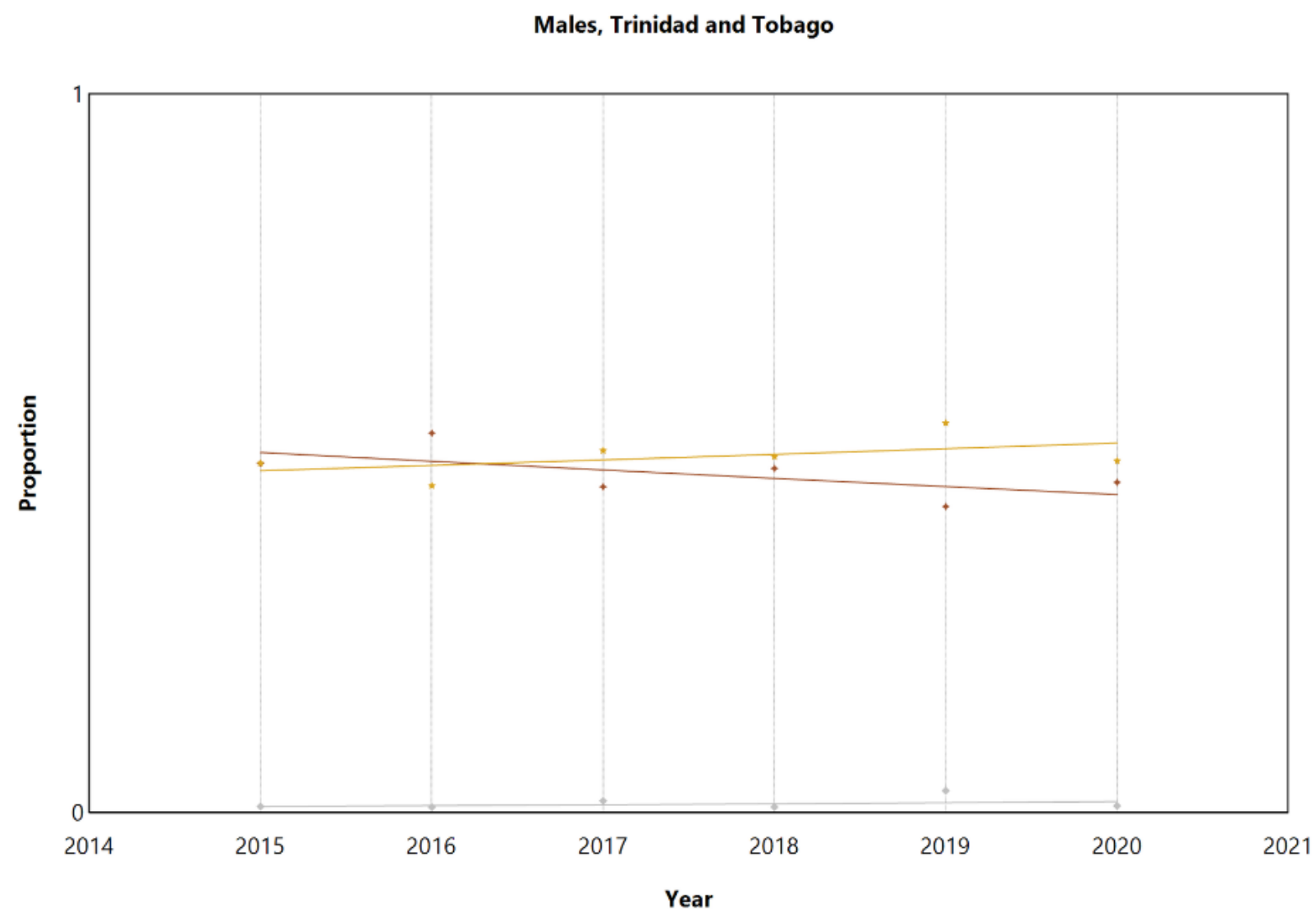
Males, Trinidad and Tobago
1
0
Proportion
2014
2015
2016
2017
2018
2019
2020
2021
Year

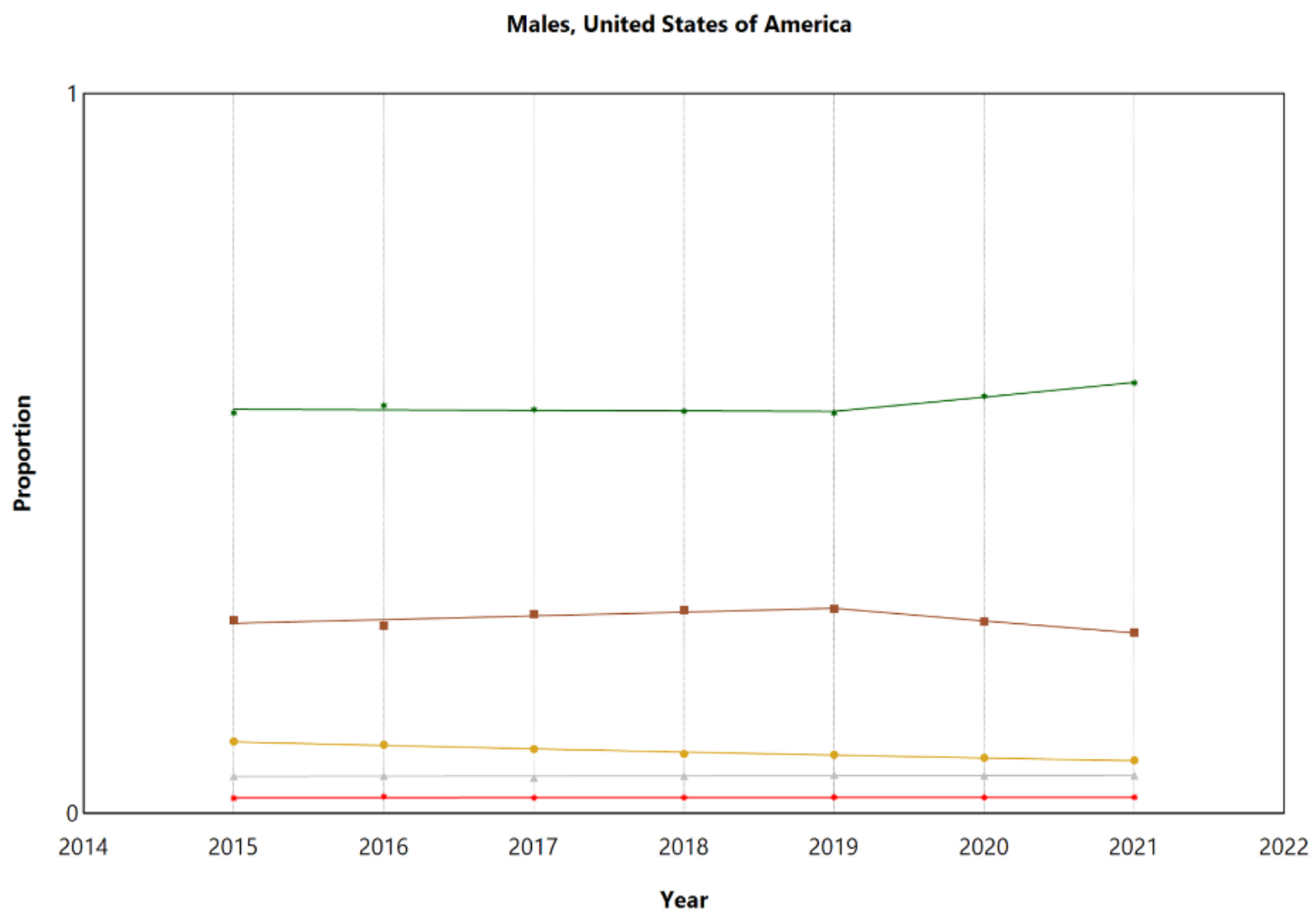
Males, United States of America
1
0
Proportion
2014
2015
2016
2017
2018
2019
2020
2021
2022
Year

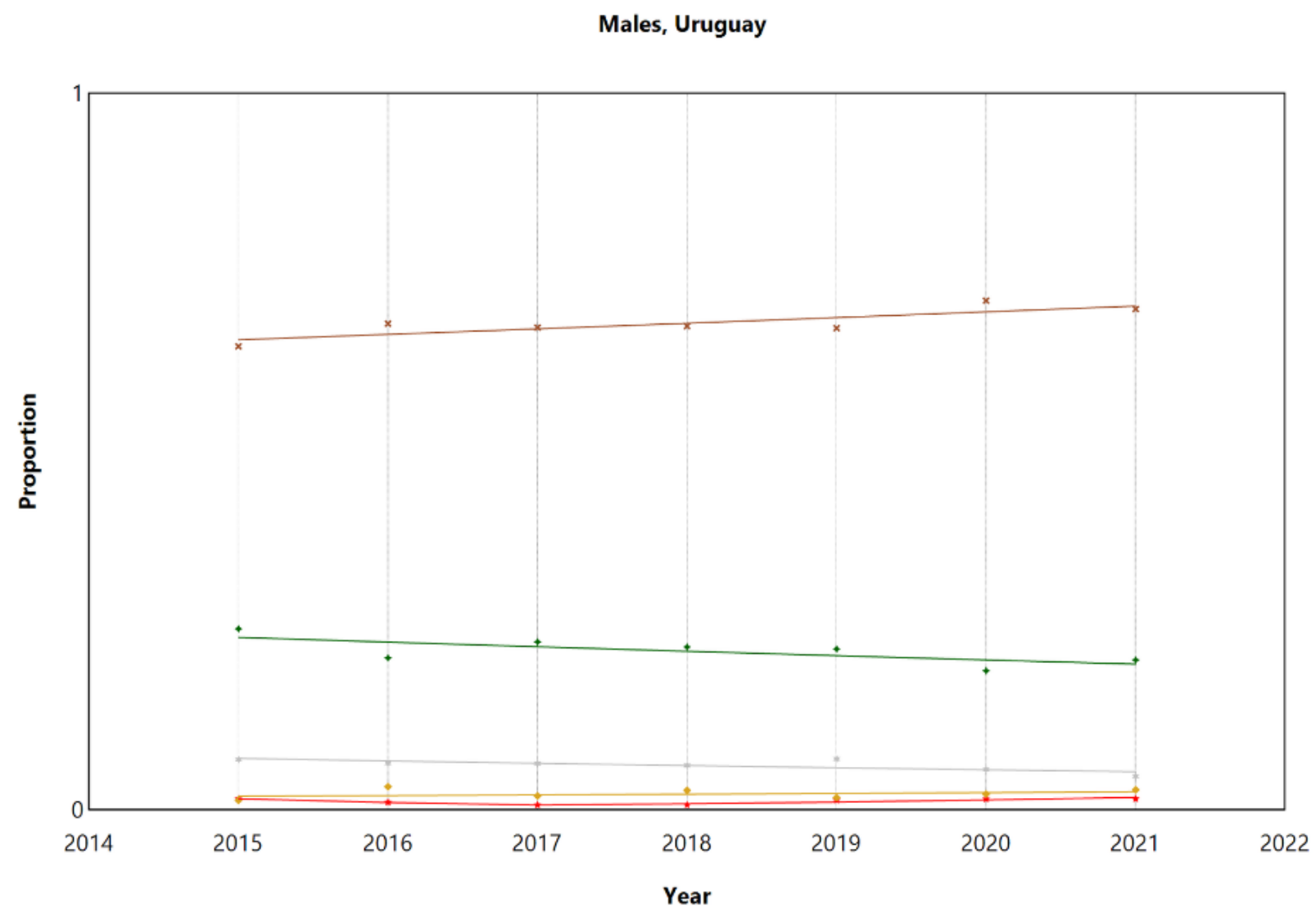
Males, Uruguay
1
0
Proportion
2014
2015
2016
2017
2018
2019
2020
2021
2022
Year

(C)

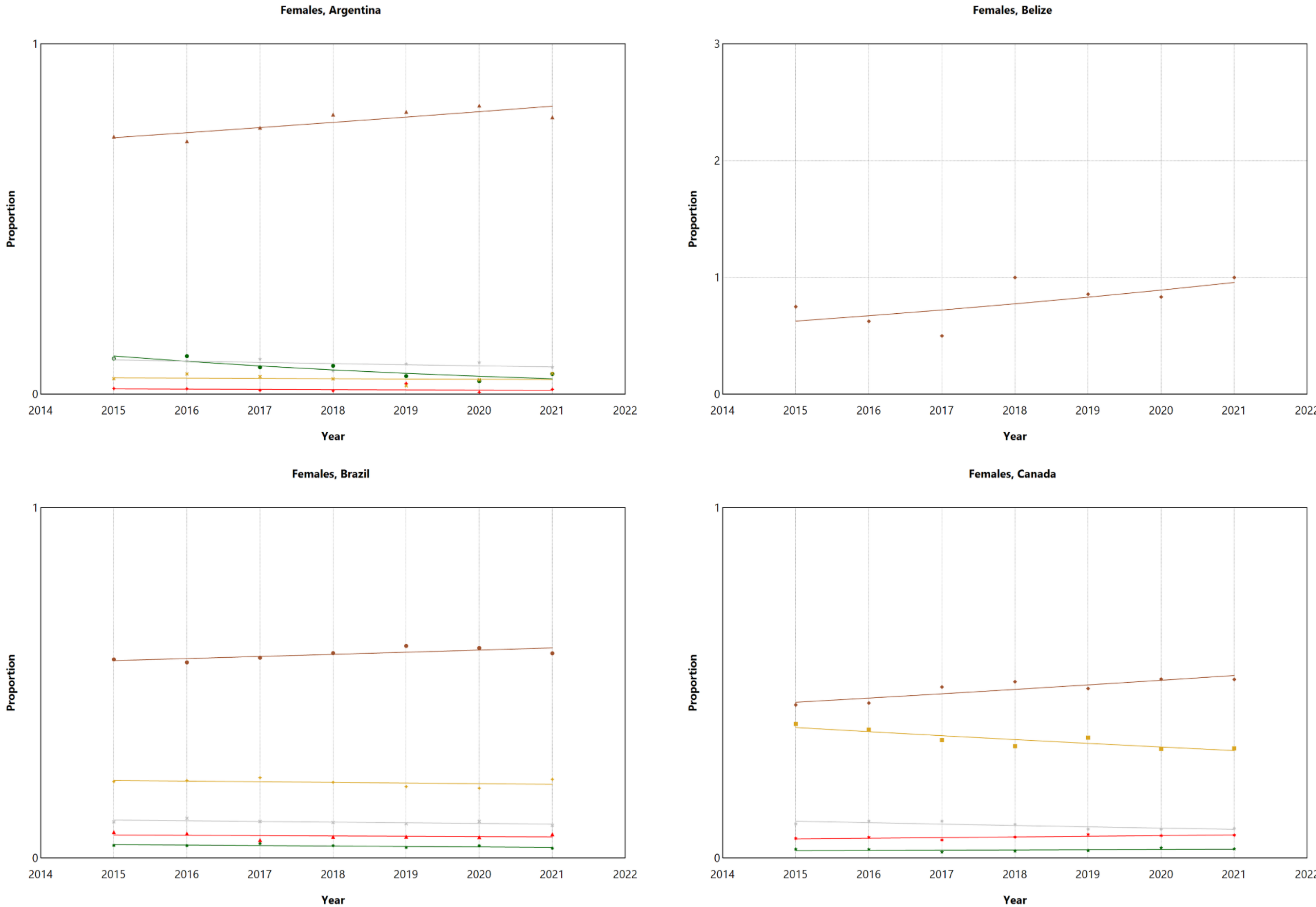

Females, Argentina
Proportion
Year
0
1
2014
2015
2016
2017
2018
2019
2020
2021
2022
Females, Belize
Proportion
Year
0
1
2
3
2014
2015
2016
2017
2018
2019
2020
2021
2022
Females, Brazil
Proportion
Year
0
1
2014
2015
2016
2017
2018
2019
2020
2021
2022
Females, Canada
Proportion
Year
0
1
2014
2015
2016
2017
2018
2019
2020
2021
2022

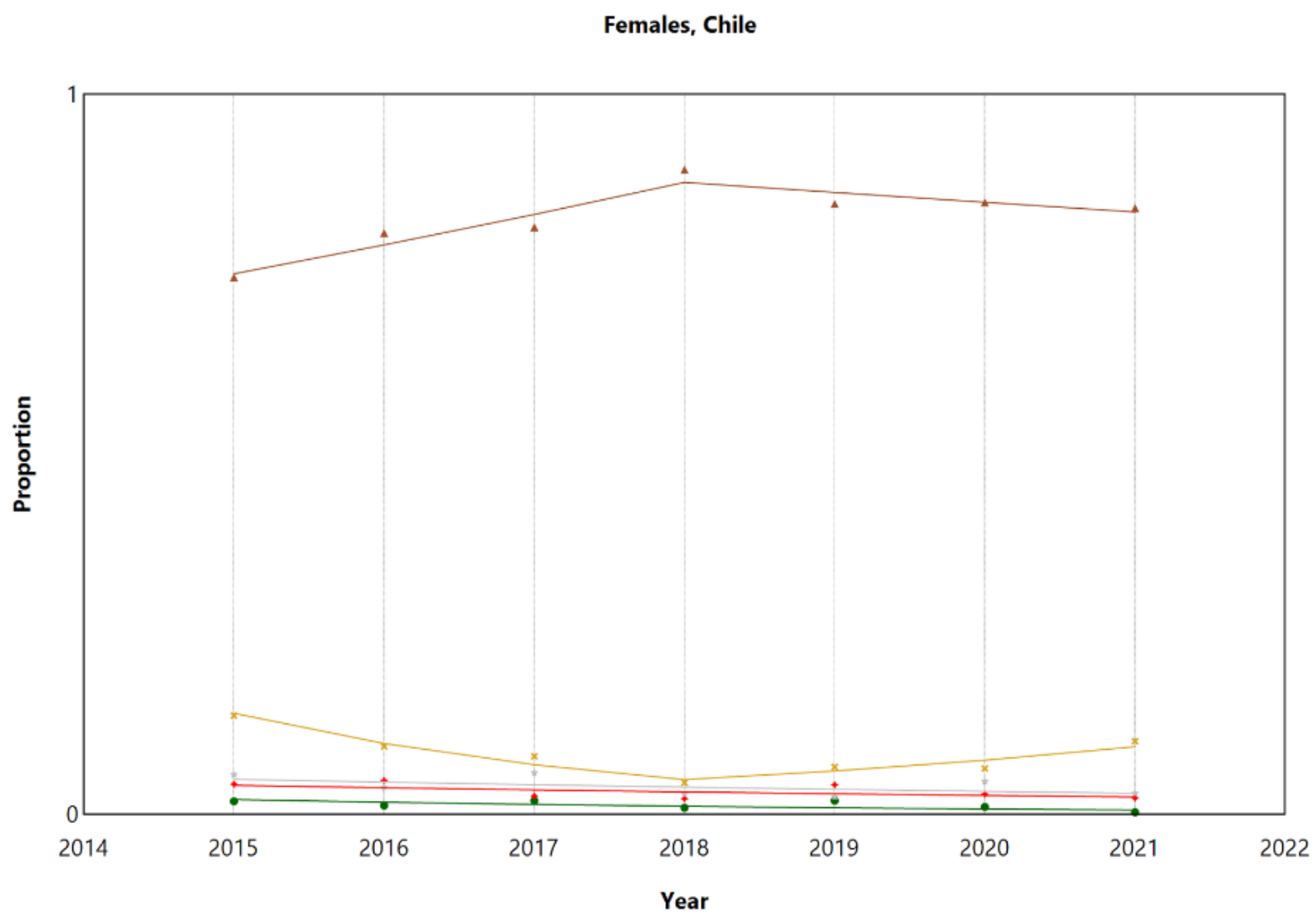
Females, Chile
Proportion
Year
1
0
2014
2015
2016
2017
2018
2019
2020
2021
2022

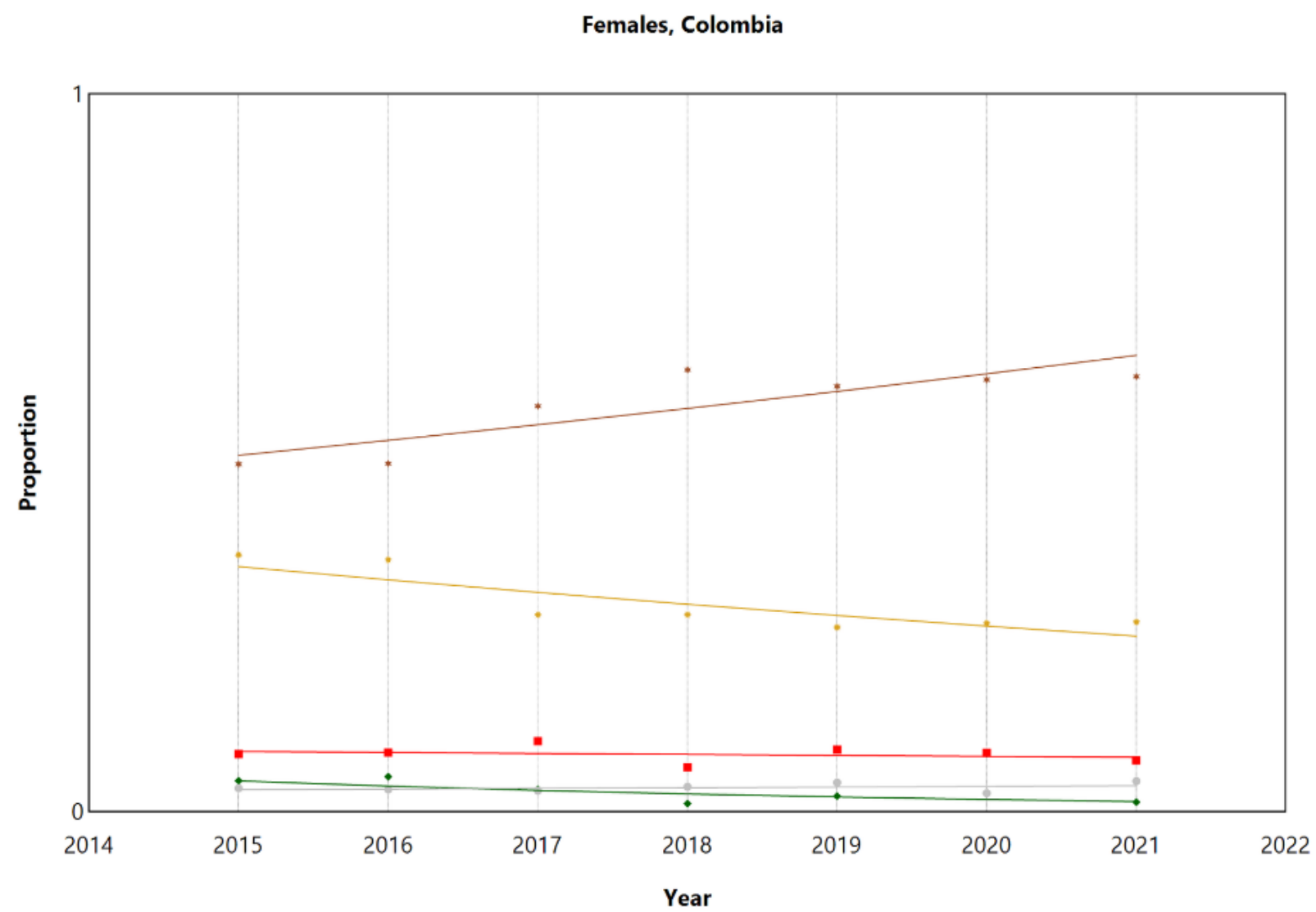
Females, Colombia
Proportion
Year
1
0
2014
2015
2016
2017
2018
2019
2020
2021
2022

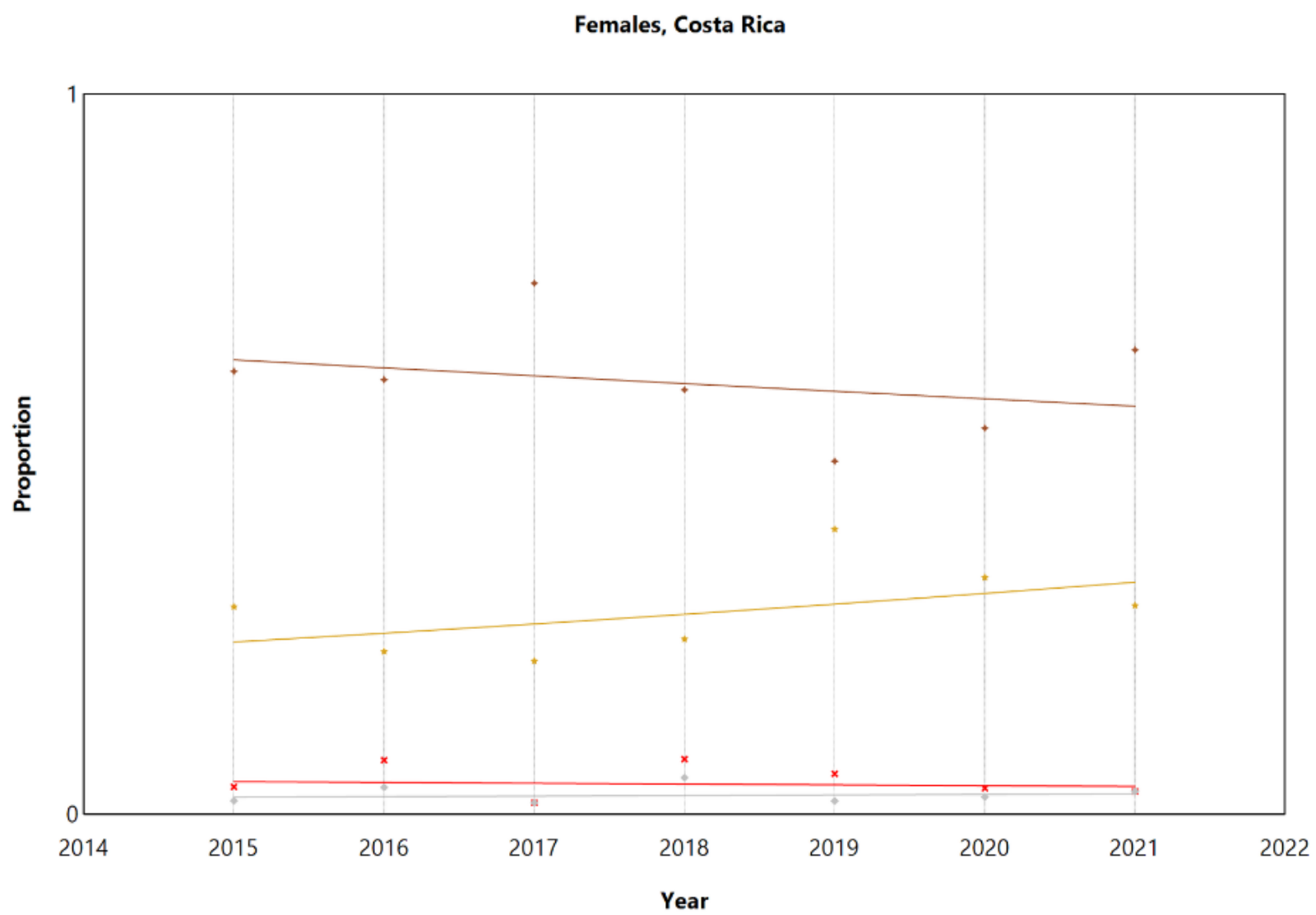
Females, Costa Rica
Proportion
Year
1
0
2014
2015
2016
2017
2018
2019
2020
2021
2022

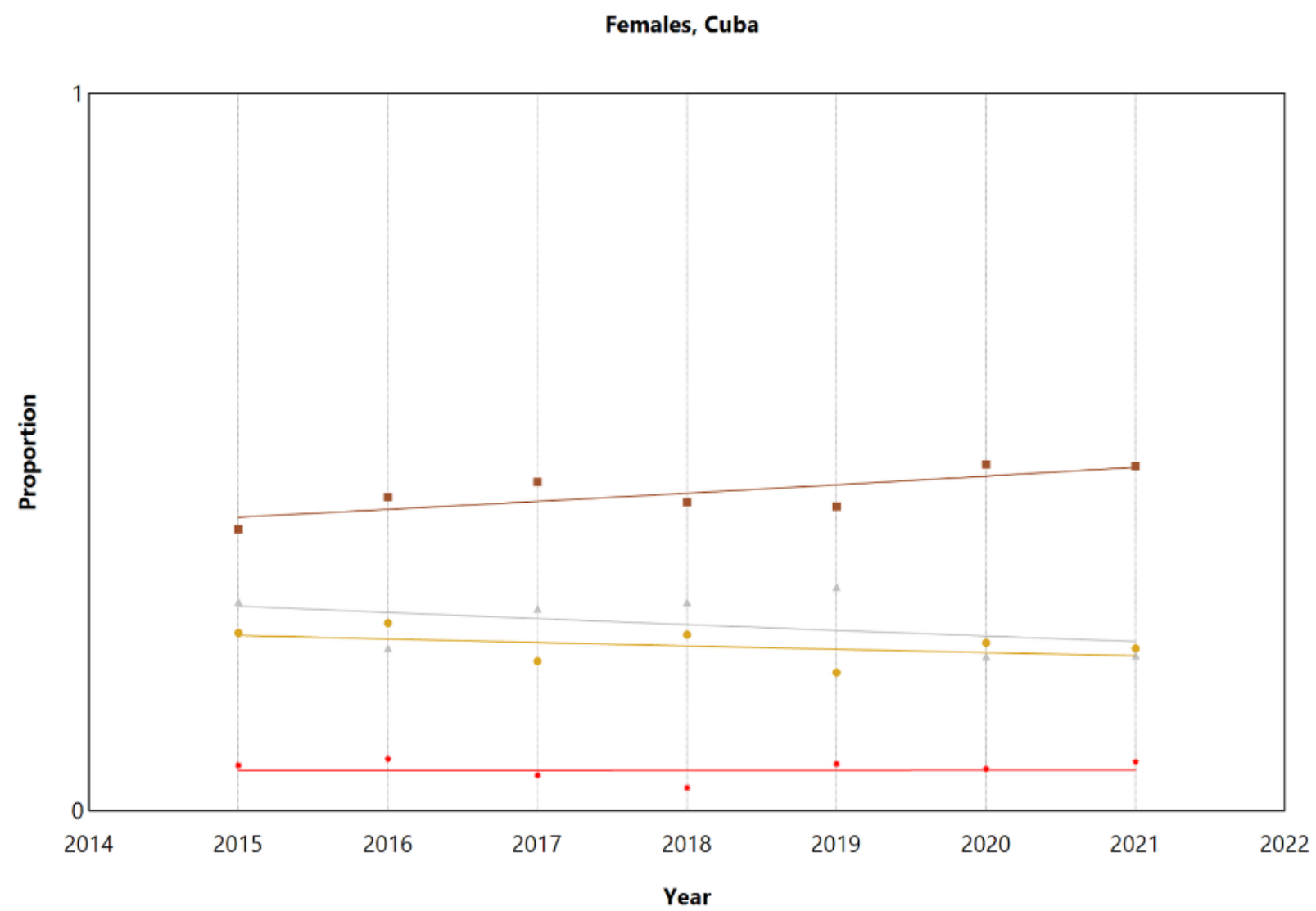
Females, Cuba
Proportion
Year
1
0
2014
2015
2016
2017
2018
2019
2020
2021
2022

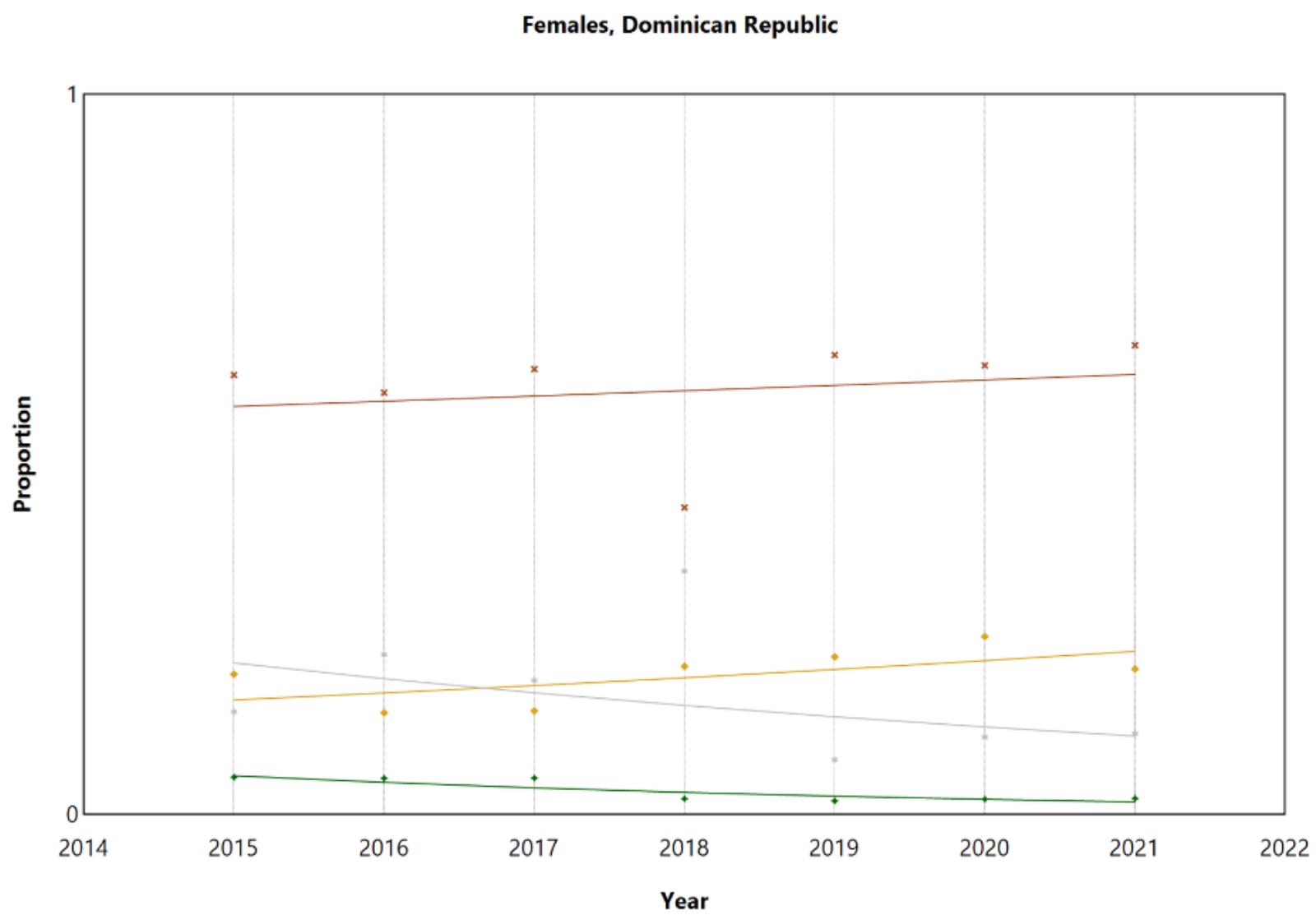
Females, Dominican Republic
Proportion
Year
1
0
2014
2015
2016
2017
2018
2019
2020
2021
2022

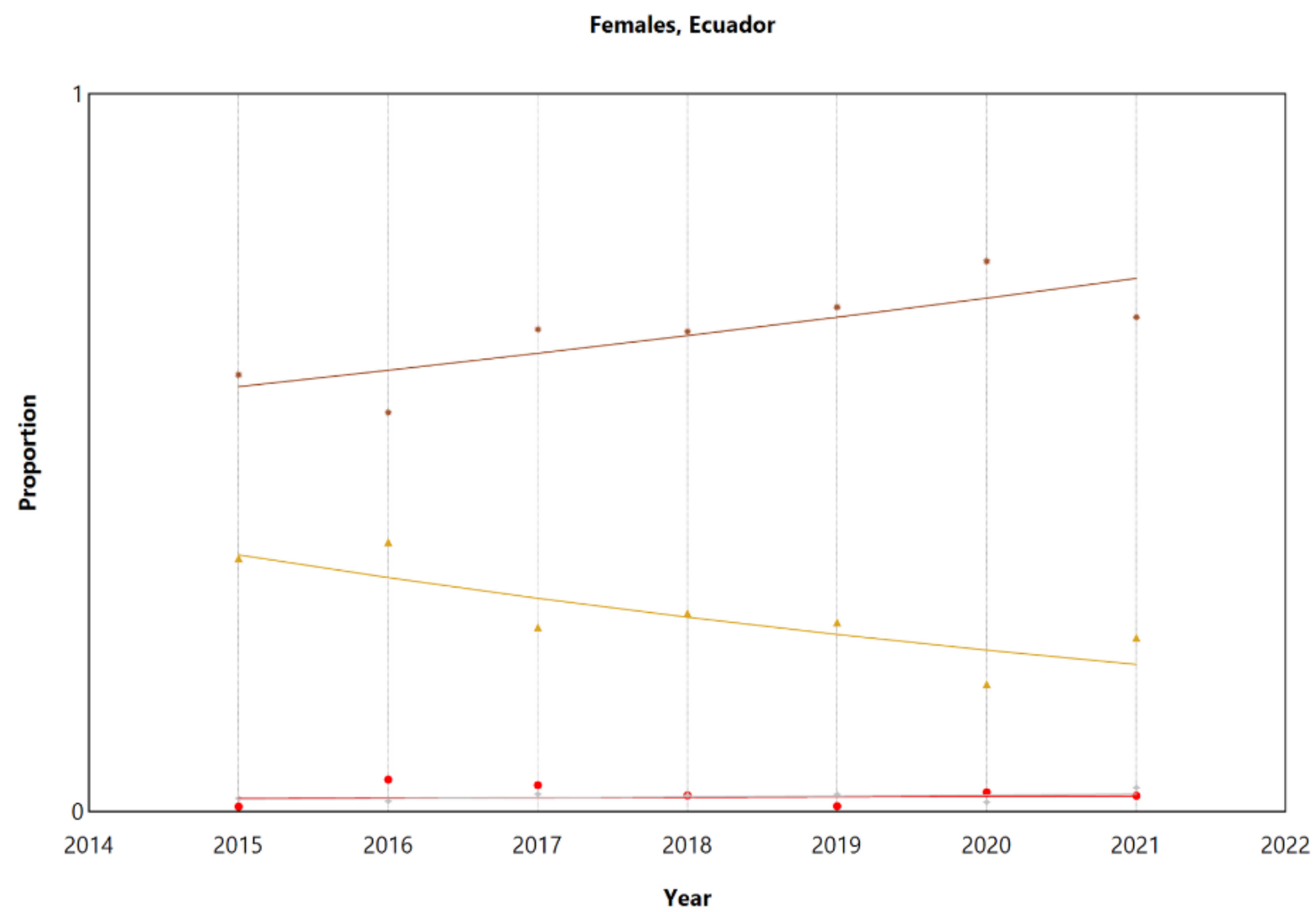
Females, Ecuador
Proportion
Year
1
0
2014
2015
2016
2017
2018
2019
2020
2021
2022

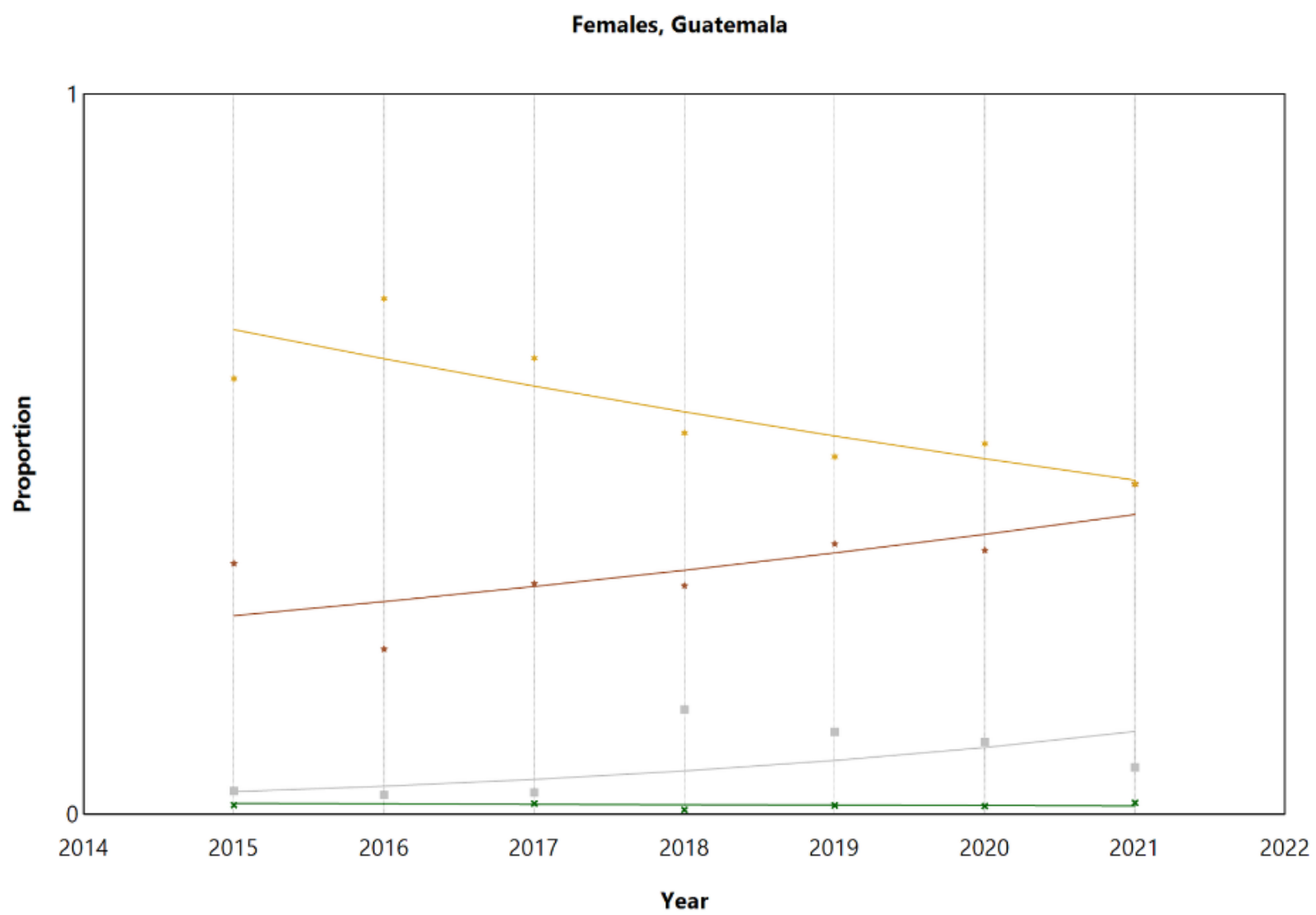
Females, Guatemala
Proportion
Year
1
0
2014
2015
2016
2017
2018
2019
2020
2021
2022

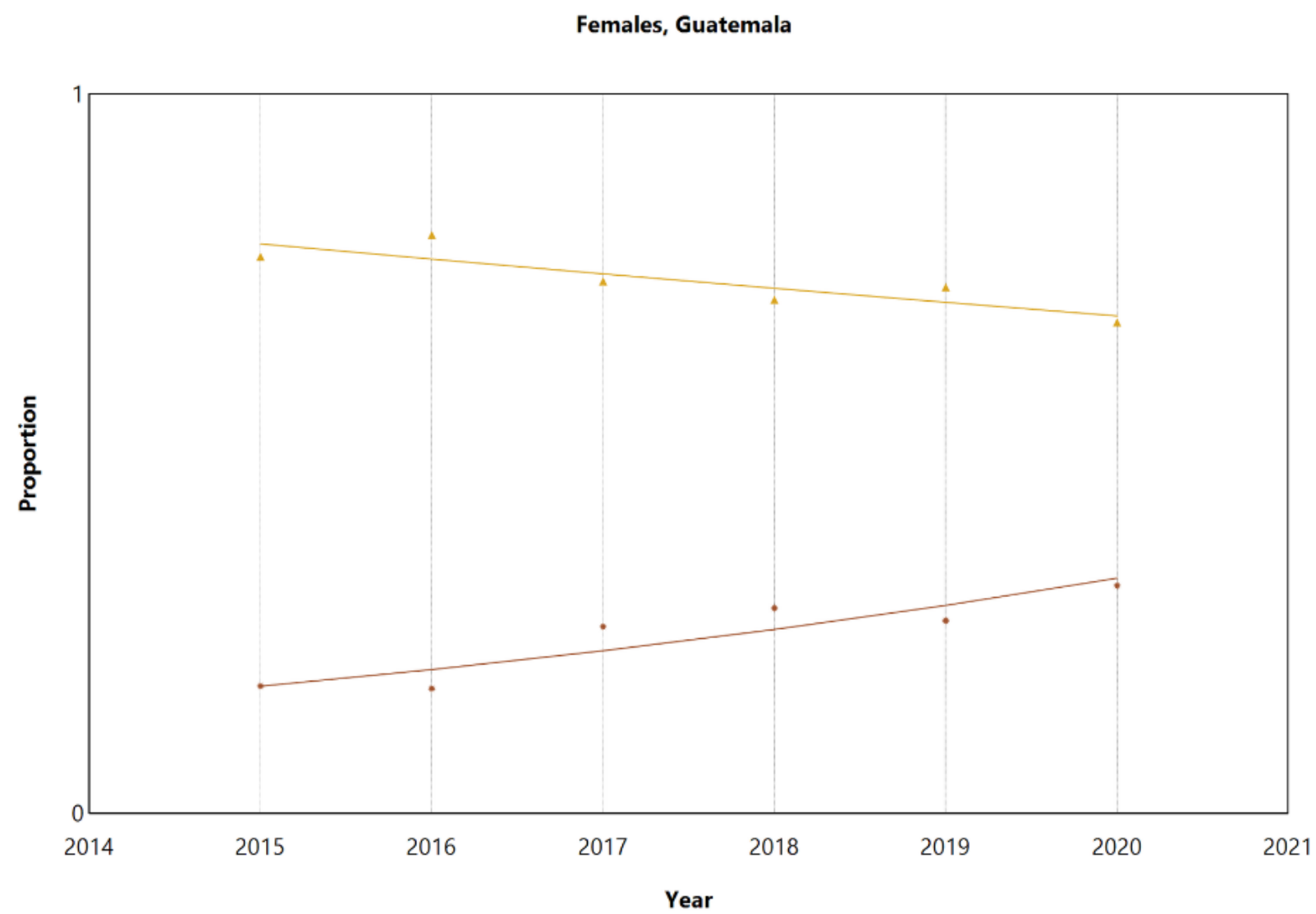
Females, Guatemala
Proportion
Year
1
0
2014
2015
2016
2017
2018
2019
2020
2021

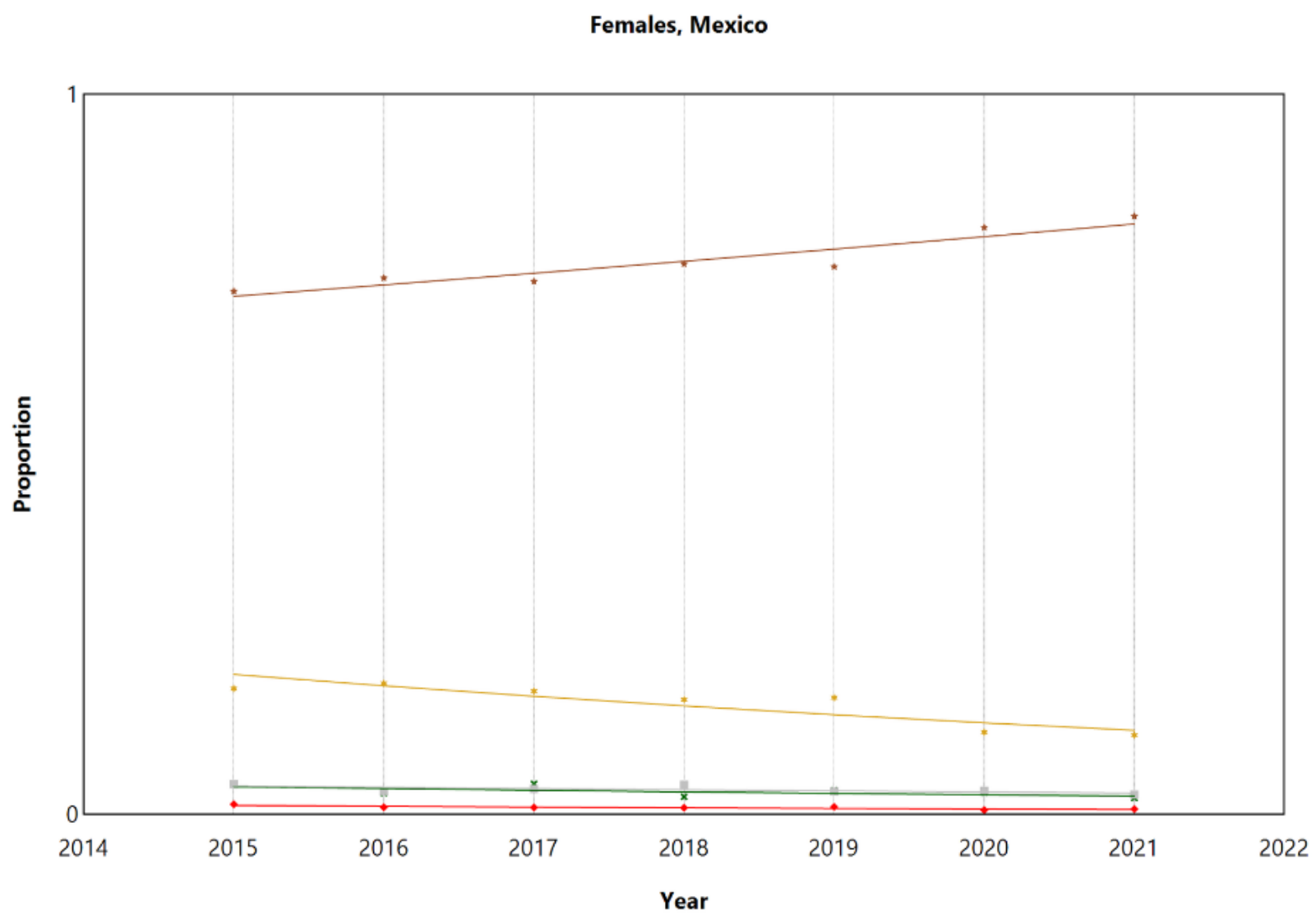

Females, Mexico
Proportion
Year
1
0
2014
2015
2016
2017
2018
2019
2020
2021
2022

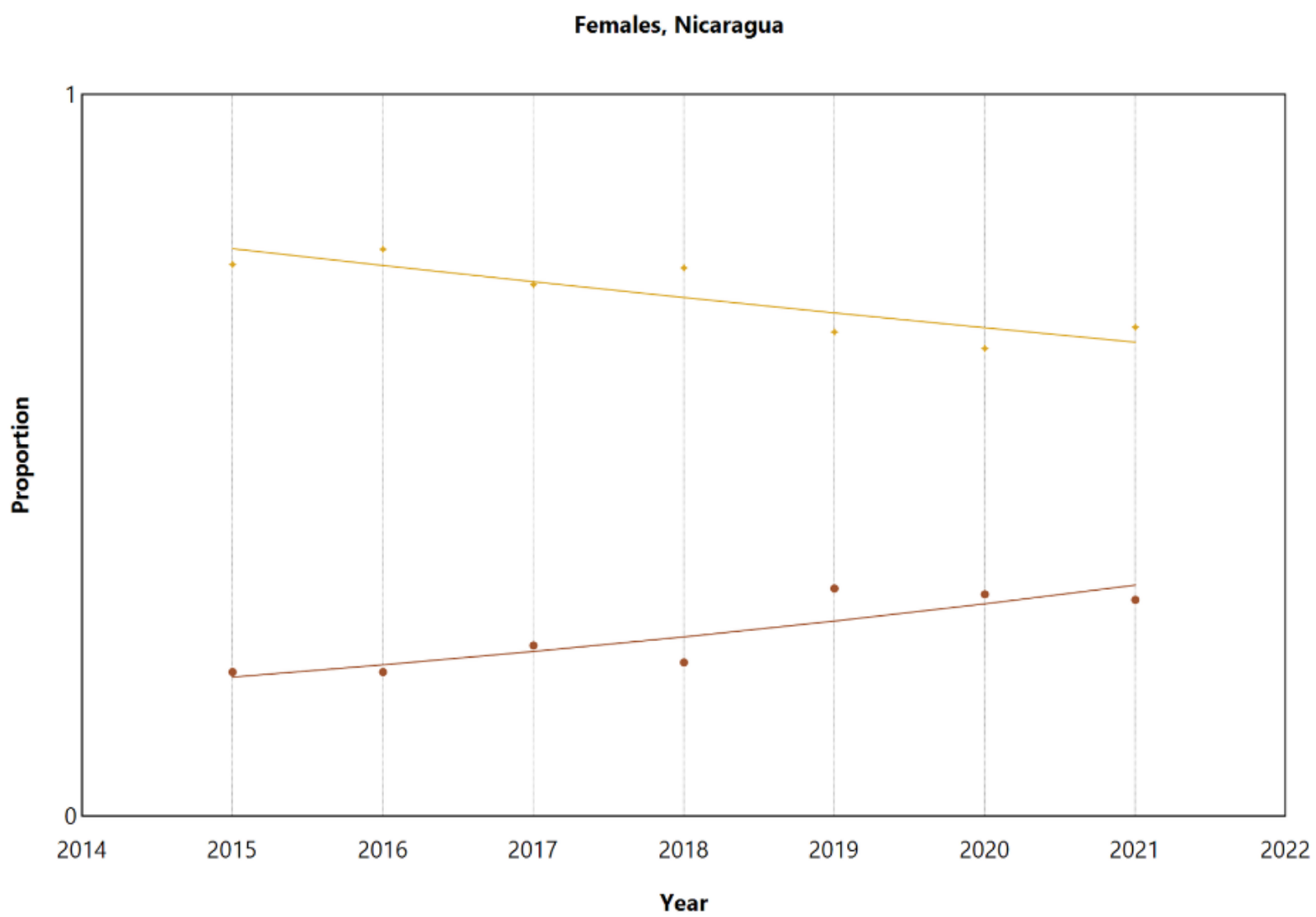

Females, Nicaragua
Proportion
Year
1
0
2014
2015
2016
2017
2018
2019
2020
2021
2022

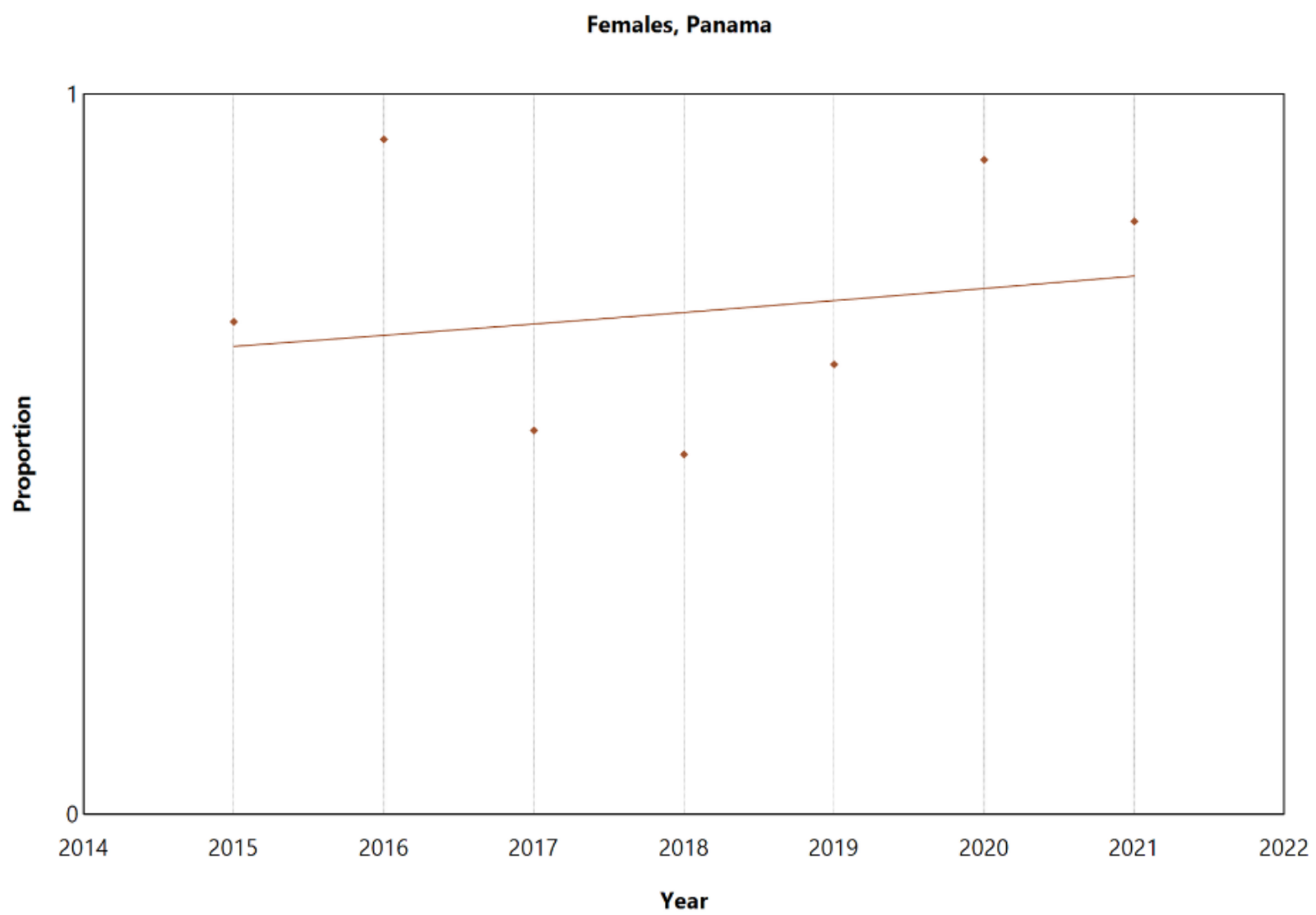

Females, Panama
Proportion
Year
1
0
2014
2015
2016
2017
2018
2019
2020
2021
2022

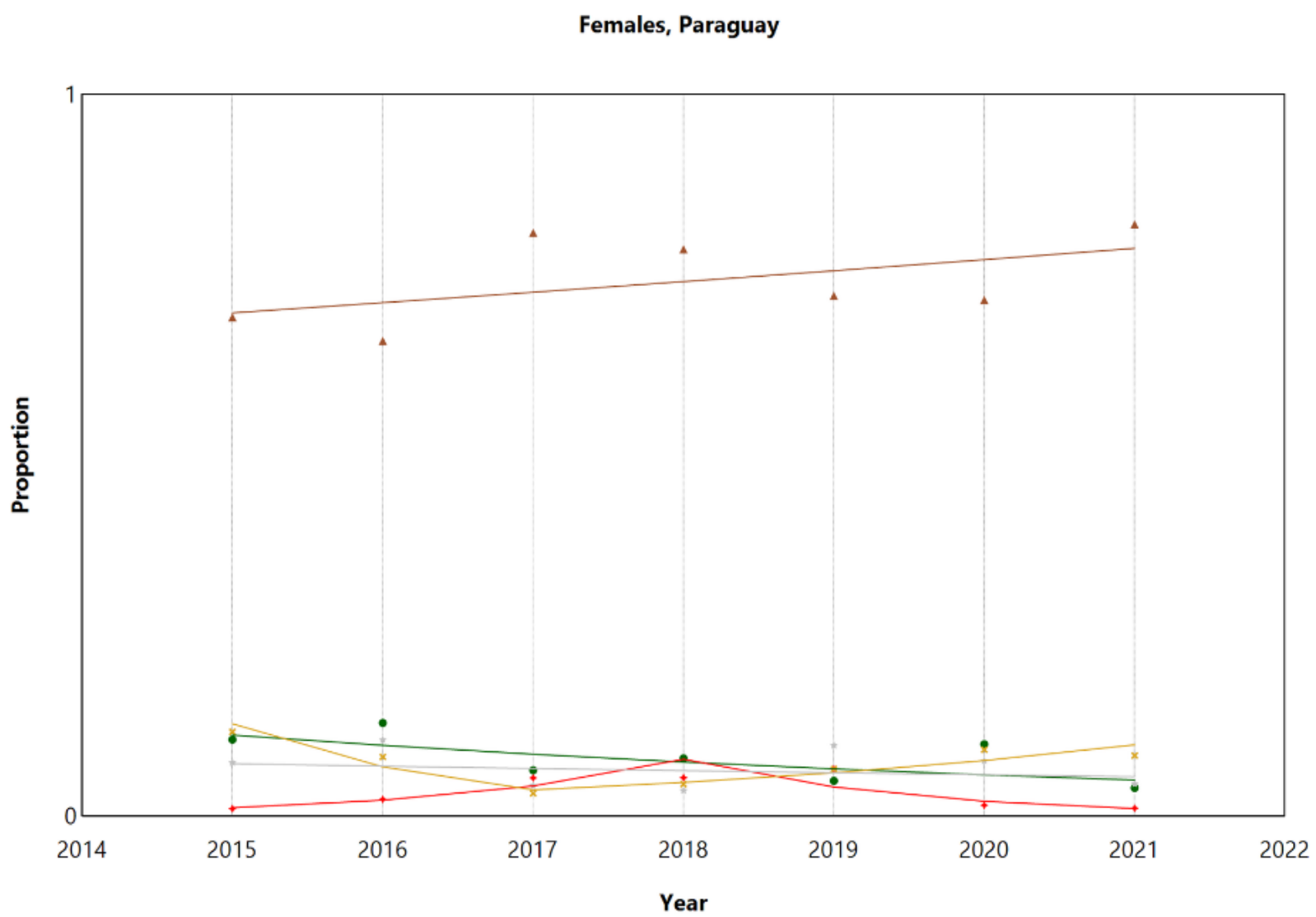

Females, Paraguay
Proportion
Year
1
0
2014
2015
2016
2017
2018
2019
2020
2021
2022

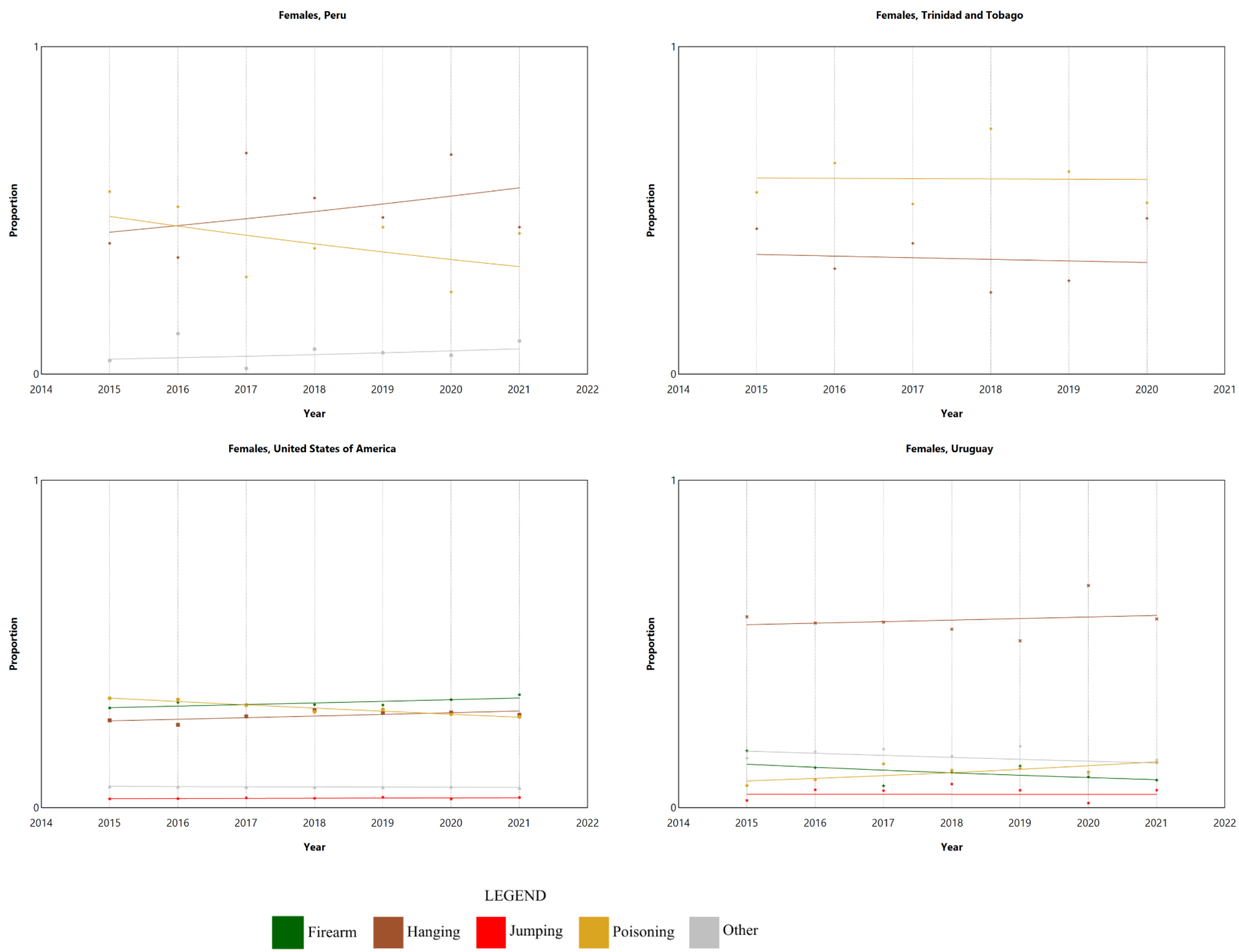

Females, Peru
Females, Trinidad and Tobago
Females, United States of America
Females, Uruguay
Proportion
Year
0
1
2014
2015
2016
2017
2018
2019
2020
2021
2022
LEGEND
Firearm
Hanging
Jumping
Poisoning
Other
